# Metal-free introduction of primary sulfonamide into electron-rich aromatics†

**DOI:** 10.1039/d4sc03075c

**Published:** 2024-07-05

**Authors:** Ming-Ming Wang, Kai Johnsson

**Affiliations:** a Department of Chemical Biology, Max Planck Institute for Medical Research Jahnstrasse 29 69120 Heidelberg Germany mingming.wang@mr.mpg.de johnsson@mr.mpg.de; b Institute of Chemical Sciences and Engineering, École Polytechnique Fédérale de Lausanne (EPFL) 1015 Lausanne Switzerland

## Abstract

We report herein a direct and practical synthesis of arylsulfonamides from electron-rich aromatic compounds by using *in situ* generated *N*-sulfonylamine as the active electrophile. Substrates include derivatives of aniline, indole, pyrrole, furan, styrene and so on. The reaction proceeds under mild conditions and tolerates many sensitive functional groups such as alkyne, acetate, the trifluoromethoxy group or acetoxymethyl ester. Applications of this method for the construction of metal ion sensors and fluorogenic dye have been demonstrated, thus highlighting the potential of this method for probe development.

## Abstract 2

Inspired by the Burgess reagent, we report a mild and practical synthesis of primary sulfonamides from electron-rich aromatic compounds by using *in situ* formed *N*-sulfonylamine as the electrophile.

## Graphical abstract

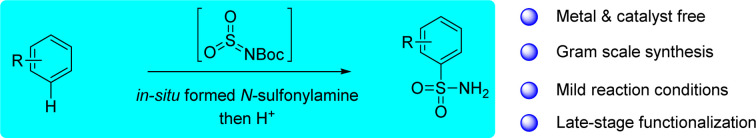

## Introduction

**Scheme 1:**
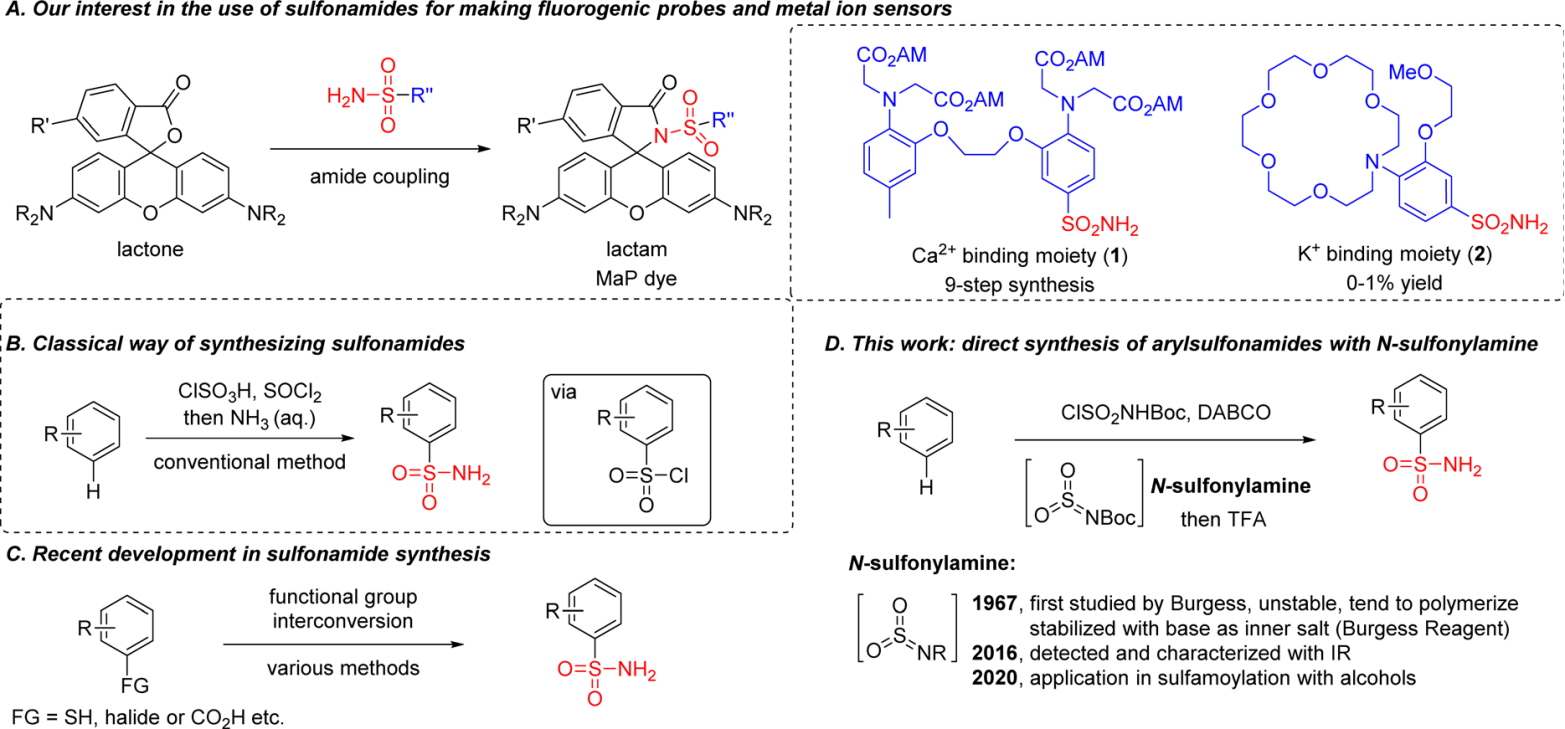
Our previous work using sulfonamides for fine-tuning rhodamine properties and further applications in creating metal ion sensors (A). Traditional methods for sulfonamide synthesis (B). Synthesis of sulfonamides from pre-functionalized substrates (C). This work using *N*-sulfonylamine for direct access to sulfonamides from electron-rich aromatic compounds (D).

Primary sulfonamides R–SO_2_NH_2_ are abundant in pharmaceutical compounds,^1^ dyes,^2^ catalysts,^3,4^ polymers^5^ and other structures.^6–9^ For example, by incorporating sulfonamide into classical rhodamine dyes, our group reported a general strategy in 2020 for making cell-permeable and fluorogenic probes (so-called ‘MaP’-dyes, Scheme 1A).^10,11^ Based on this design, we further reported a semi-synthetic calcium sensor in 2022 by combining the known calcium chelator BAPTA [1,2-bis(*o*-aminophenoxy)ethane-*N*,*N*,*N*′*N*′-tetraacetic acid] with rhodamine dyes through a sulfonamide linkage.^12^

However, a conventional method for sulfonamide synthesis was performed under strong acidic/basic conditions using a chlorosulfonic acid – ammonia sequence (Scheme 1B), which forced us to take a nine-step synthetic route with multiple protection/deprotection steps for the synthesis of 1. Meanwhile we were also interested in developing a potassium sensor using the MaP dye strategy, but the synthetic efficiency for accessing sulfonamide 2 with the conventional method was too low (0–1% yield). Considering the importance of the sulfonamide group in medicinal chemistry, novel SO_2_ surrogates have been developed for accessing sulfonamides, including: DABSO [DABCO·(SO_2_)_2_] from the Willis group (2011);^13^ potassium metabisulfite from the Wu group (2012);^14^ tetrabromothiophene *S*,*S*-dioxide from the Lian group (2021).^15–17^ More recently, sulfonamides were also prepared from carboxylic acids by using a photocatalytic decarboxylative process, as reported by the Larionov group, the MacMillan group, the Willis group and the Zhang/Yang group independently.^18–21^ Despite these advances, pre-functionalized substrates were required in most cases (Scheme 1C).^22–34^ An electrochemical process for direct sulfonamide synthesis was reported in 2021 by the Waldvogel group, but primary sulfonamides were not accessed.^35^ These limitations motivated us to develop a direct method for the introduction of primary sulfonamide group into aromatic compounds.

While searching for an alternative route, we were intrigued by the structure of the Burgess reagent,^36^ a mild dehydrating reagent as well as a sulfamoylation reagent for the synthesis of sulfamate^37,38^ or sulfamide.^39–41^ Recently it has also been used for preparing sulfonamide, but functional group tolerance was limited since an organometallic reagent or Lewis acid/heating was needed for activation.^42–44^ In fact, *N*-sulfonylamine (or aza-sulfene) was proposed in Burgess's early report as an elusive intermediate for the preparation of the Burgess reagent, and trapping it with nucleophiles such as amine or alkene to form sulfamide or a cycloadduct was successfully demonstrated.^45,46^ However, *N*-sulfonylamine was only detected and characterized by matrix-isolation IR spectroscopy in 2016, largely due to its high instability.^47^ Although its applications in synthetic chemistry were still limited, we envisioned that *N*-sulfonylamine could be a better electrophile than the Burgess reagent for making sulfonamides, in a similar way to sulfur trioxide for accessing sulfonic acids (Scheme 1D).

## Results and discussion

We therefore explored the possibility of using *in situ* generated *N*-sulfonylamine for direct sulfonamide synthesis. *N*,*N*-dimethylaniline 3a was selected as a model substrate and ClSO_2_NHBoc as an *N*-sulfonylamine precursor (Table 1). To our pleasure, formation of Boc-protected sulfonamide 4a at the *para*/*ortho* position was observed when treating a solution of 3a and a base with ClSO_2_NHBoc at room temperature for the *in situ* formation of *N*-sulfonylamine. Among the tested organic bases, DABCO gave the highest conversion as well as *para*/*ortho* selectivity (Table 1, entries 1–4). An inorganic base like Na_2_CO_3_ gave products in poor yield and selectivity (Table 1, entry 5). In comparison to diethyl ether, dichloromethane as a solvent can further improve the conversion, albeit with lower *para*/*ortho* selectivity (Table 1, entry 6). In contrast, only 7% of 3a was consumed in the absence of a base while the rest was found to be protonated, which was then restored after a basic aqueous workup (Table 1, entry 7). Lewis acid additives such as AlCl_3_ or TMSOTf were unable to catalyze this Friedel–Crafts type reaction (Table 1, entry 8), which was not surprising since it has always been a challenge for aniline derivatives to participate in other electrophilic aromatic substitution reactions like alkylation^48–53^ or acylation.^54^ Switching from *N*-sulfonylamine to the Burgess reagent led to a lower conversion of 3a, while the DABCO based inner salt showed no reactivity (see ESI, Scheme S1†).

**Table 1:** Optimization of the reaction conditions^a^

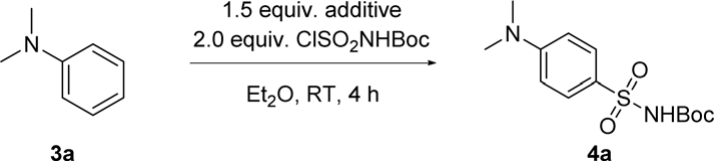				
Entry	Additive	Solvent	Conversion^b^ (%)	*p*/*o* ratio
1	DABCO	Et_2_O	92	17 : 1
2	Et_3_N	Et_2_O	80	3.6 : 1
3	Pyridine	Et_2_O	20	3.8 : 1
4	DBU	Et_2_O	25	5 : 1
5	Na_2_CO_3_	Et_2_O	20	2.5 : 1
6	DABCO	CH_2_Cl_2_	98	2.6 : 1
7	—	Et_2_O	7	—
8	AlCl_3_/TMSOTf^c^	CH_2_Cl_2_	<5	—

a Reaction conditions: reactions were run at a 0.10 mmol scale in 1.0 mL of solvent at room temperature for 4 h. Boc = CO_2_*t*-Bu.

b The conversion was determined by crude ^1^H NMR after an aqueous workup.

c Only 0.1 equivalent of Lewis acid was used.

**Scheme 2:**
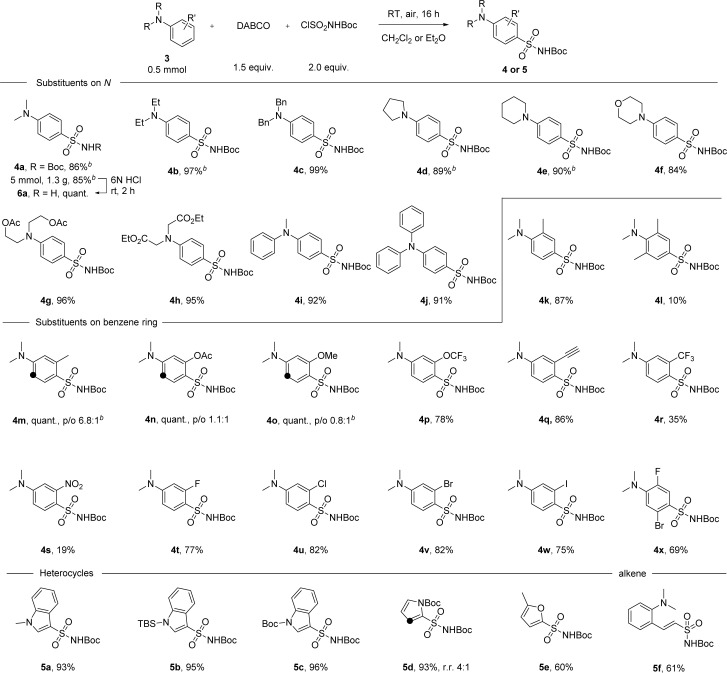
Scope of aromatic compounds in the direct sulfonamide synthesis. ^[a]^Reaction conditions: 3 (0.50 mmol), DABCO (0.75 mmol), ClSO_2_NHBoc (1.0 mmol), in CH_2_Cl_2_ (5.0 mL) at room temperature for 16 hours. Black dots indicated other reactive sites where regioisomers were formed. In all cases, regioisomers were easily separated from each other by flash column chromatography, except in the case of 5d. ^[b]^ Et_2_O (5.0 mL) was used as solvent.

With the optimal conditions in hand, we then examined the scope of the reaction with either diethyl ether or dichloromethane as the solvent (Scheme 2). *Para*-substituted sulfonamide 4a was obtained in 86% yield and the synthesis can be easily scaled up, delivering 1.3 grams of the product without losing efficiency. Facile removal of the Boc group was achieved by treating 4a with 6 N HCl solution at room temperature for 2 hours, and the primary sulfonamide 6a was obtained quantitatively by filtration after neutralization. Anilines with cyclic or acyclic alkyl substituents on the nitrogen atom underwent the reaction well to give products 4b–h in yields ranging between 84 and 99%. This reaction was extended successfully to diphenylmethylamine and triphenylamine, with products 4i-j isolated in 91–92% yield. No erosion of the reactivity was observed with just one methyl group at the *ortho* position, but the conversion rate dropped to 10% when *ortho* positions were both occupied (products 4k-l). Substituents at the *meta* position, especially electron-donating groups, were found to have a strong impact on *para*/*ortho* selectivity. On switching from a methyl group to acetate or to a methoxy group, though the yield was not affected, the ratio of the *ortho*-substituted product gradually increased even when Et_2_O was used as the solvent (products 4m–o). With electron-withdrawing substituents at the *meta* position, the *para*-substituted product was obtained as the major product. For example, in the case of the trifluoromethoxy group, *para*-substituted 4p was isolated in 78% yield. A series of functional groups including alkynyl, trifluoromethyl and the strongly electron-withdrawing nitro group were well tolerated, giving the corresponding products in 19–86% yield (products 4q–s). Substrates with one or more halogen atoms can also be tolerated and products 4t–x were obtained in 69–82% yield. Apart from aniline derivatives, other electron-rich aromatic compounds like indole or pyrrole with various substituents on the *N* atom were also tested under standard conditions and excellent yields were obtained in these cases (products 5a–d). Furan can participate in the reaction as well, with product 5e isolated in 60% yield. When a vinyl group was present in the substrate, the reaction took place first at the vinyl position before the aryl substitution happened (product 5f). However, efforts to further extend the reaction scope to include less electron-rich substrates, such as anisole, chlorobenzene, or azobenzene, were not successful (see ESI, Scheme S3†).

**Scheme 3:**
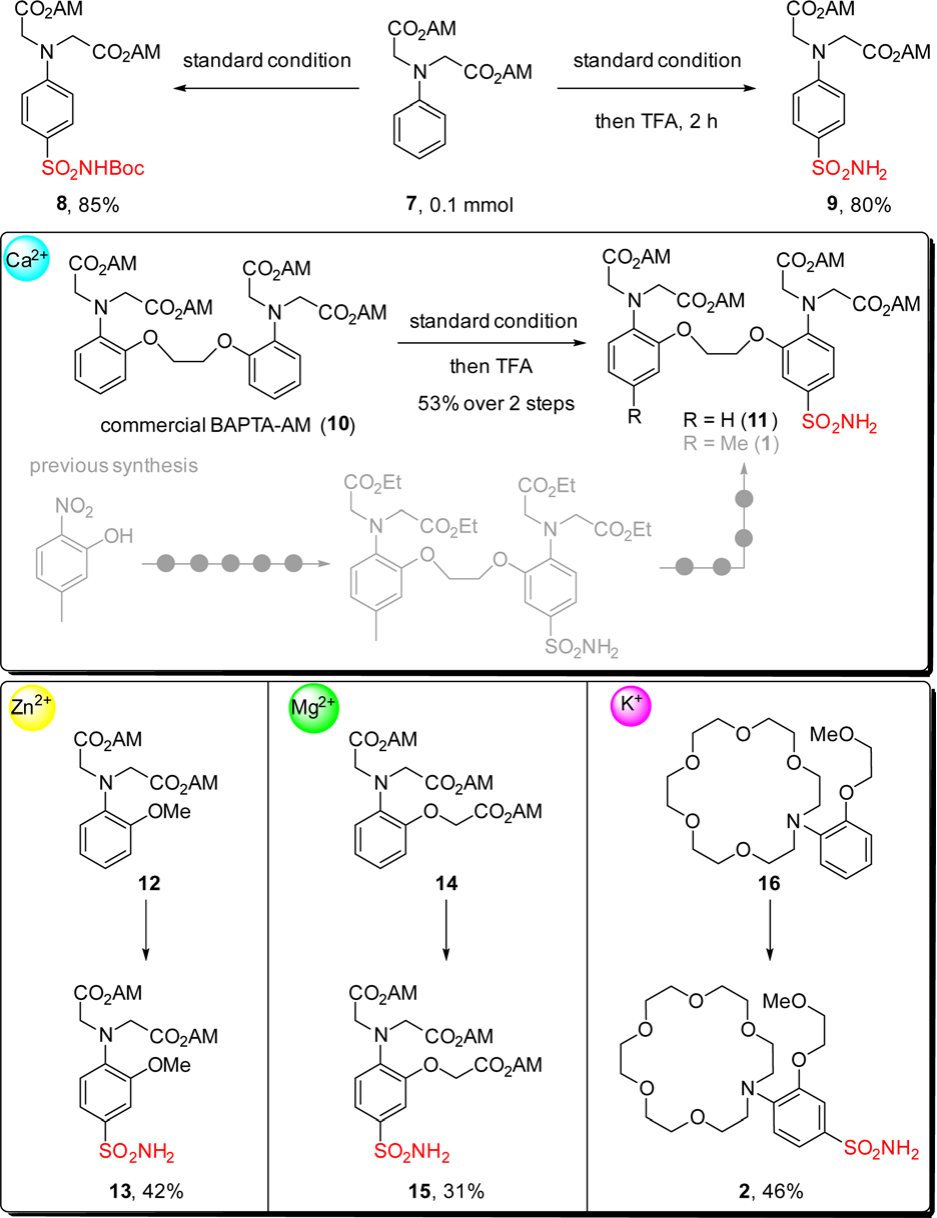
Applications of this method for the introduction of sulfonamide groups into metal ion chelators.

With our interest in developing semi-synthetic fluorescent sensors, we then applied this reaction for introducing sulfonamide group into metal-ion binding moieties (Scheme 3). A simple substrate containing acetoxymethyl ester (AM ester) 7 was tested first, giving the desired product 8 in 85% yield. Combining this step with Boc deprotection in a one-pot fashion by adding TFA to the reaction crude was also successful, and primary sulfonamide 9 was isolated in 80% yield. We then moved forward to test this reaction on the commercially available calcium chelator BAPTA-AM ester 10. Gratifyingly sulfonamide 11 was obtained in 53% yield with this two-step sequence, which greatly reduced synthetic steps as compared to the previously reported nine-step route for accessing sulfonamide 1.^12^ Encouraged by this result, we further synthesized sulfonamides 13 and 15, which can be used for making zinc ion and magnesium ion fluorescent sensors respectively.^55–57^ Performing this reaction on the aza-crown ether 16, a chelator favors coordination to potassium ions over other metal ions,^58–61^ led to the formation of sulfonamide 2 in 46% yield which was hard to access by other methods.

**Scheme 4:**
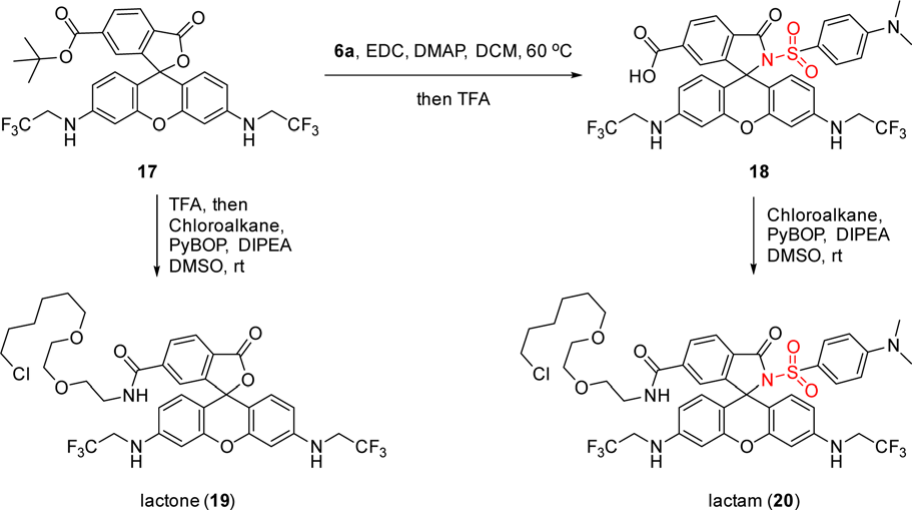
The use of sulfonamide 6a in accessing fluorogenic MaP dye 18 and further synthesis of rhodamine 500R-chloroalkane conjugates 19 and 20 for the evaluation of fluorogenicity.

Furthermore, a fluorogenic MaP dye 18 was synthesized *via* amide coupling of 17 with 6a, which could be seen as the parent structure of these metal ion sensors but was not included in our previous studies (Scheme 4).^11^ We then installed chloroalkane, a ligand to label the HaloTag protein covalently, to 17 and 18 for accessing lactone 19 and lactam 20 as their HaloTag conjugates. Live-cell, no-wash fluorescence imaging confirmed the high fluorogenicity of 20, as compared to 19 (see ESI, Scheme S4†).

**Scheme 5:**
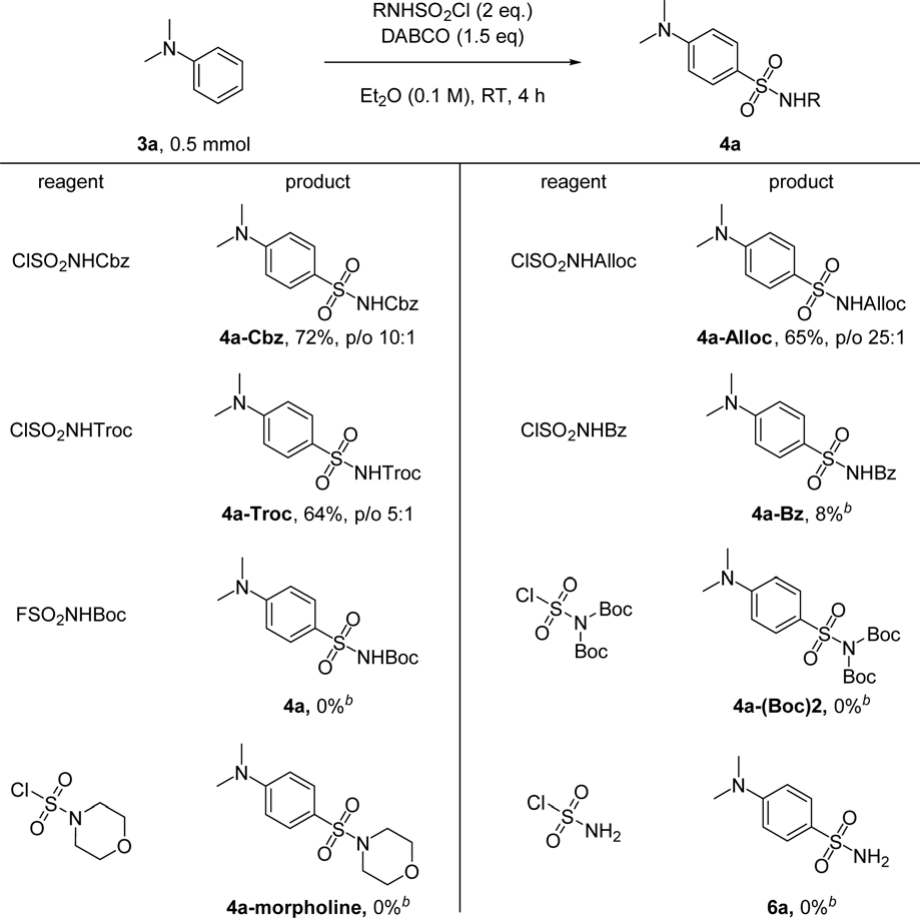
Reaction of 3a with other *N*-sulfonylamine precursors. Cbz = CO_2_CH_2_Ph, Alloc = CO_2_CH_2_CH=CH_2_, Troc = CO_2_CH_2_CCl_3_, and Bz = COPh. ^[b]^ 0.1 mmol scale.

In the end, we compared the reactivities of several different *N*-sulfonylamine precursors (Scheme 5). Besides ClSO_2_NHBoc, other carbamate-based reagents like ClSO_2_NHCbz, ClSO_2_NHAlloc and ClSO_2_NHTroc can also be used, yielding 4a with different protecting groups which can be readily removed by hydrogenation, palladium chemistry, or Zn, respectively. ClSO_2_NHBz can also be used as an *N*-sulfonylamine precursor, but the desired 4a-Bz was obtained in only 8% yield, likely due to the competing rearrangement reaction of *N*-sulfonylbenzamide at room temperature.^62^ Although FSO_2_NHBoc shows improved stability, it failed to deliver any product under standard conditions. Additionally, reagents without free N–H, such as ClSO_2_N(Boc)_2_ and the commercial reagent morpholine-4-sulfonyl chloride, cannot be used for transferring the sulfonamide group to 3a. Sulfamoyl chloride (ClSO_2_NH_2_) also failed to give any product, probably due to the high p*K*_a_ of its N–H bond compared to that of ClSO_2_NHBoc.

## Conclusions

In summary, we have developed a practical and general strategy for introducing primary sulfonamide group into aniline derivatives as well as other electron-rich aromatic compounds such as indole, pyrrole, furan and alkenes. Owing to the mild reaction conditions, a series of functional groups were tolerated, especially the labile AM ester. With this method, several sulfonamides containing a metal ion chelator (Ca^2+^, Zn^2+^, Mg^2+^, and K^+^) were rapidly accessed, underlining the potential of the method to rapidly generate complex chemical probes in the late stage. Efforts for further constructing and characterizing semi-synthetic fluorescent probes through the MaP dye strategy are currently underway in our lab.

## Data availability

Additional synthetic, analytical, and biological data are available in the ESI† of this article. Raw data for NMR, MS and fluorescent images are available at http://zenodo.org, DOI: http://doi.org/10.5281/zenodo.10683729.

## Author contributions

K. J. directed the research. M.-M. W. designed and performed the experiments. K. J. and M.-M. W. wrote the manuscript together.

## Conflicts of interest

The authors declare no conflict of interest.

## Supplementary Material

SC-015-D4SC03075C-s001
