# Supplementary material for: Metal-free introduction of primary sulfonamide into electron-rich aromatics
Source: Chem Sci. 2024 Jul 5;15(31):12310–5. doi: 10.1039/d4sc03075c (PMC11304520; doi:10.1039/d4sc03075c)

Electronic Supplementary Material (ESI) for Chemical Science.
This journal is © The Royal Society of Chemistry 2024

Supporting Information for

Metal-Free Introduction of Primary Sulfonamide into Electron-Rich Aromatics

Ming-Ming Wang*, Kai Johnsson*

Department of Chemical Biology, Max-Planck Institute for Medical Research, Heidelberg, Germany

corresponding author: mingming.wang@mr.mpg.de, johnsson@mr.mpg.de

Table of Contents

1. General Methods S2
2. Cell culture and microscopy S3
3. Reaction development and challenging substrates S4
4. Synthesis and characterization S6
5. Spectra for New Compounds S30

1. General Methods

For quantitative flash chromatography, distilled technical grade solvents were used. THF, Et_2O, toluene, hexane and CH_2Cl_2 were dried by passage over activated alumina under nitrogen atmosphere (H_2O content < 7 ppm, Karl-Fischer titration). All chemicals were purchased and used as received unless stated otherwise. Chromatographic purification was performed as flash chromatography using Macherey-Nagel silica 40-63, 60 Å, using the solvents indicated as eluent with 0.1-0.5 bar pressure. TLC was performed on Merck silica gel 60 F254 TLC plastic or aluminium plates and visualized with UV light, permanganate stain. Melting points were measured on a calibrated Büchi B-540 melting point apparatus using open glass capillaries. ^{1}H-NMR spectra were recorded at room temperature on a Brucker DPX-400 400 MHz spectrometer in $CDCl_3$, Acetone-d_6, CD_3CN or CD_3OD, all signals are reported in ppm with the internal chloroform signal at 7.26 ppm, the internal acetone signal at 2.09 ppm, the internal acetonitrile signal at 1.94 ppm and the internal methanol signal at 3.34 ppm as standard. The data is being reported as (s = singlet, d = doublet, t = triplet, q = quadruplet, p = quintet, m = multiplet or unresolved, br = broad signal, integration, coupling constant(s) in Hz, interpretation). ^{13}C-NMR spectra were recorded with 1H-decoupling on a Brucker DPX-400 101 MHz spectrometer in $CDCl_3$, Acetone-d_6, CD_3CN or CD_3OD, all signals are reported in ppm with the internal chloroform signal at 77.0 ppm, Acetone-d_6 signal at 29.8 ppm, CD_3CN signal at 1.3 ppm or CD_3OD signal at 49.0 ppm as standard. High resolution mass spectrometric measurements were performed by the mass spectrometry service of MPIMR on a MICROMASS (ESI) Q-TOF Ultima API.

2. Cell culture and Microscopy

Cell culture and labeling

U-2 OS cells (wild-type), U-2 OS FlpIn Halo-SNAP-NLS[1] expressing cells were cultured in Dulbecco's Modified Eagle Medium (DMEM, 4.5 g/L glucose) supplemented with 10 % (v/v) fetal bovine serum (FBS), GlutaMAX and sodium pyruvate (all Life Technologies) in a humidified 5 % CO_2 incubator at 37 °C. Cells were split every 2 – 4 days or at confluency and regularly tested for mycoplasma contamination. Cells were seeded on 10-well glass bottom plates (Greiner Bio-One) 2 days before imaging. Prior to imaging, cells were labeled with SiR-BG, **19** or **20** in imaging medium (phenolred free DMEM supplemented with GlutaMAX, sodium pyruvate and 10 % (v/v) FBS (all Life Technologies)).

Confocal Microscopy

Confocal microscopy was performed on a Leica DMi8 microscope (Leica Microsystems) equipped with a Leica TCS SP8 X scanhead, a SuperK white light laser, a HC PL APO CS2 20.0 x/0.75 objective and an incubator with CO_2 as well as temperature control (Life Imaging Services, 5 %, 37 °C).

Evaluation of Fluorogenicity - Live-Cell, No-Wash Confocal Microscopy

For rhodamine 500R derived HaloTag probes (**19** and **20**), co-cultured U-2 OS FlpIn Halo-SNAP-NLS expressing cells and wild-type U-2 OS cells (1:1) were pre-labeled with 500 nM of SiR-BG (overnight) and washed with imaging medium. After labeling with 200 nM **19** and **20** for 2.5 hours, confocal images were recorded without washing steps. Microscopy conditions: λ_{ex}: 510 nm and 645 nm, detection range: 520 – 600 nm and 655 – 720 nm, image size: 446.02 µm x 446.02 µm, pixel size: 436 nm, pixel dwell time: 0.86 µs. The summed stacks were analyzed to measure the ratios between nuclear signal (F_{nuc}) and cytosolic background signal (F_{cyt}) of **19** and **20**. F_{nuc}: Mean values of rhodamine 500R fluorescence of ROIs within the nuclei of U-2 OS FlpIn Halo-SNAP-NLS expressing cells normalized to the SiR fluorescence. F_{cyt}: Mean values of rhodamine 500R fluorescence of ROIs within the cytosol of wild-type U-2 OS cells. Bright field images were used to locate wild-type U-2 OS cells represented by dotted lines. In total, 60 cells (30 U-2 OS FlpIn Halo-SNAP-NLS expressing cells and 30 wild-type U-2 OS cells) were examined from 5 images for each probe.

1 Wang, L.; Tran, M.; D'Este, E.; Roberti, J.; Koch, B.; Xue, L.; Johnsson, K. *Nat. Chem*. **2020**, *12*, 165-172.

3. Reaction development

3.1 Comparison of reactivity with common sulfamoylation reagents

Scheme S1. Reactions with other sulfamoylation reagents

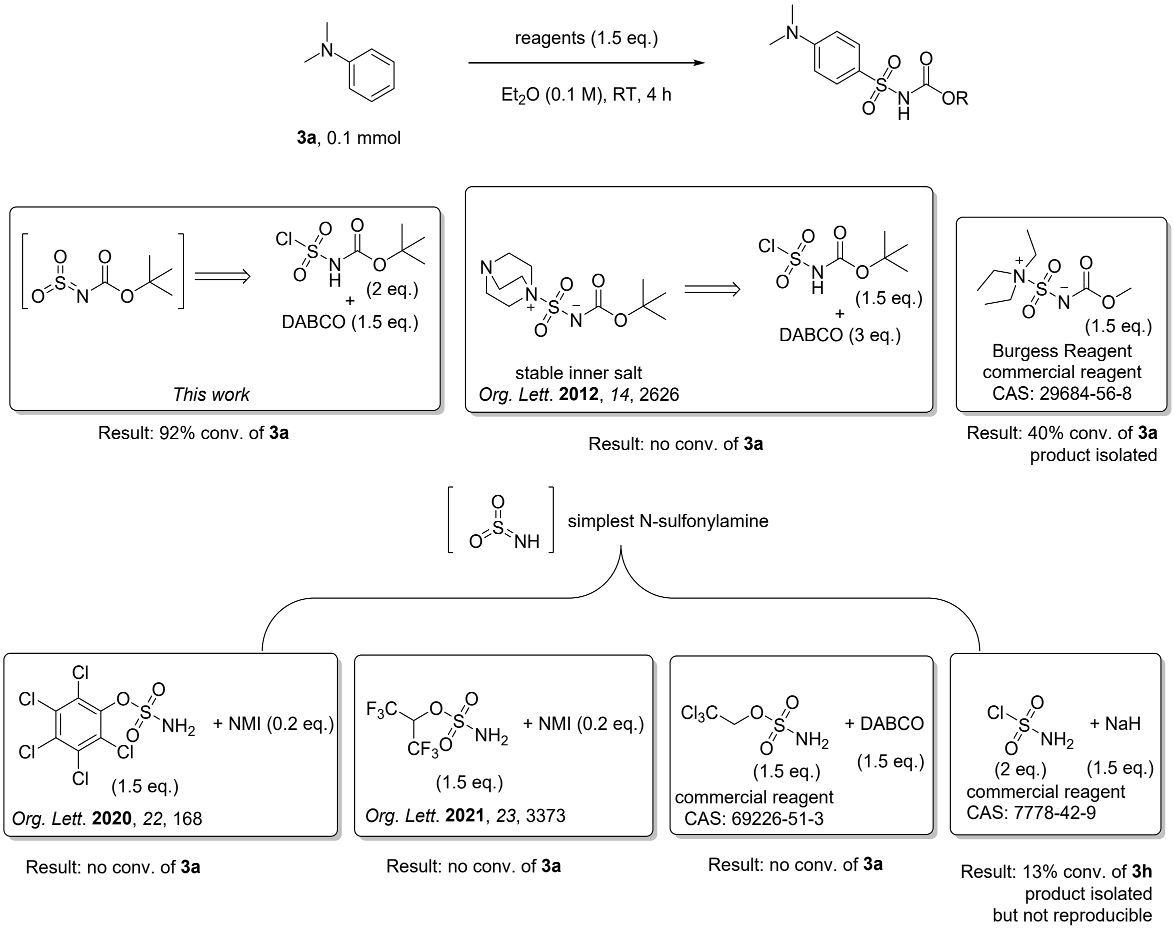

Scheme S2. Reactions with other sulfamoyl chlorides or tosyl chloride

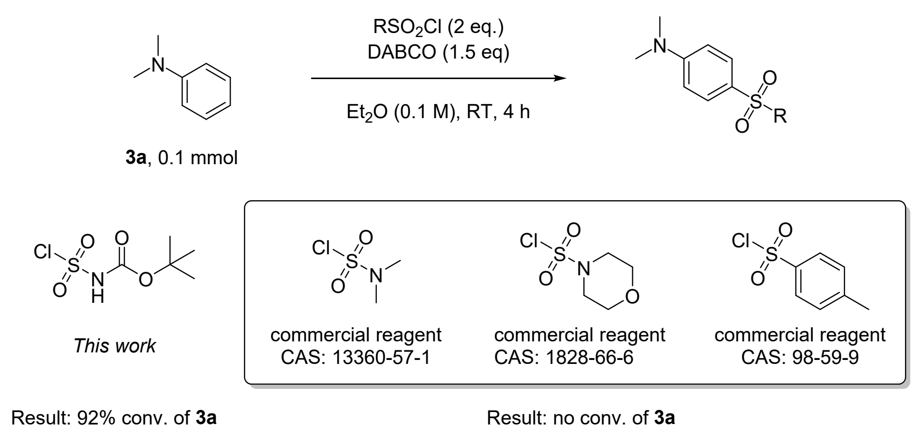

3.2 Challenging substrates for this reaction

Scheme S3. Substrates with no reactivity or undesired site-selectivity under standard conditions

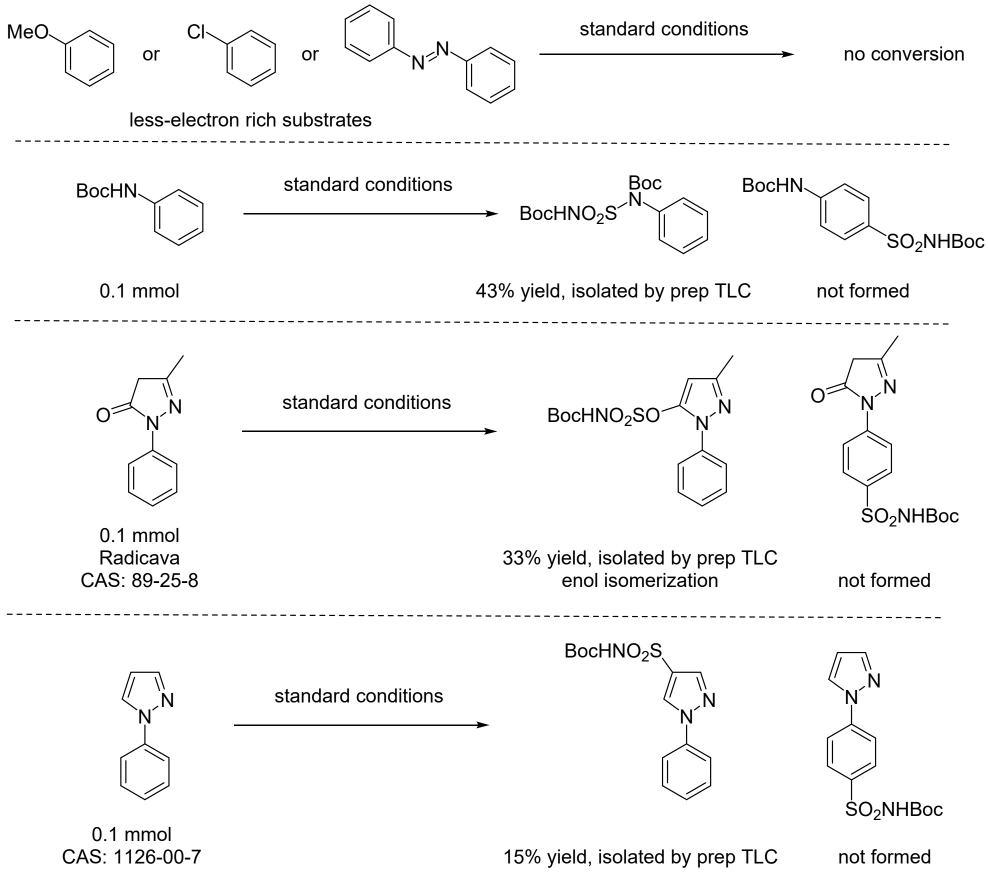

4. Synthesis and characterization

4.1 Synthesis of reagents

N-(*tert*-butoxycarbonyl)sulfamoyl chloride

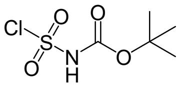

Following a modified version of a reported procedure,[2] to a solution of *tert*-butanol (15.7 mL, 165 mmol, 1.1 equiv.) in 10 mL toluene at 0 °C was added a solution of chlorosulfonyl isocyanate (13.1 mL, 150 mmol, 1.0 equiv.) in 30 mL toluene dropwise under argon. The reaction mixture was allowed to warm to room temperature and was stirred vigorously for 1 hour. 140 mL pentane was then added and the solid was resuspended by using a spatule (This step is very important in order to get the product in the powder form). The resulting suspension was stirred for another 30 min before being filtered. The filter cake was washed with pentane 3 x 100 mL and dried under atmospheric pressure in fume hood for 30 min. Then it was transferred to a flask and connected to a rotavap for further rotation until constant weight was reached (no heating, no vacuum, just rotation). *N*-(*tert*-butoxycarbonyl)sulfamoyl chloride (26.3 g, 122 mmol, 81%) was obtained as a white solid, which was stored at -18 °C and used without further purification (no obvious loss of its reactivity was observed even after storage for 1 year).

^{1}H NMR (400 MHz, $CDCl_3$): δ = ^{1}H NMR (400 MHz, $CDCl_3$) δ 8.43 (s, 1H, N*H*), 1.56 (s, 1H, C*H3*).

^{1}H NMR data correspond to the reported values.[1]

Benzyl (chlorosulfonyl)carbamate

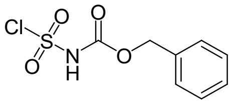

Following a modified version of a reported procedure,[2] to a solution of benzoic acid (1.00 g, 8.19 mmol, 1.0 equiv.) in 10 mL toluene was added chlorosulfonyl isocyanate (1.16 g, 8.19 mmol, 1.0 equiv.) dropwise under argon. The reaction mixture was stirred at room temperature for 1 hour. 40 mL pentane was then added and the formed white precipitate was filtered, washed with pentane 3 x 10 mL and dried under atmospheric pressure in fume hood for 30 min. Then it was transferred to a flask and connected to a rotavap for further rotation until constant weight was reached (no heating, no vacuum, just rotation). Benzyl (chlorosulfonyl)carbamate (1.90 g, 7.61 mmol, 93%) was obtained as a white solid, which was stored at -18 °C and used without further purification.

^{1}H NMR (400 MHz, $CDCl_3$): δ = 8.61 (s, 1H, N*H*), 7.40 (s, 5H, Ar*H*), 5.32 (s, 2H, C*H2*).

Allyl (chlorosulfonyl)carbamate

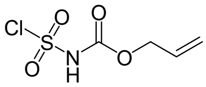

[2] Gorelik, D. J.; Turner, J. A.; Taylor, M. S. *Org. Lett.* **2022**, *24*, 5249-5253.

Following a modified version of a reported procedure,[3] to a solution of allyl alcohol (476 mg, 8.19 mmol, 1.0 equiv.) in 10 mL DCM was added chlorosulfonyl isocyanate (1.16 g, 8.19 mmol, 1.0 equiv.) dropwise. The reaction mixture was stirred at room temperature for 1 hour and then solvent was removed *in vacuo*. Allyl (chlorosulfonyl)carbamate (1.61 g, 8.07 mmol, 98%) was obtained as a pale yellow oil, which was stored at -18 °C and used without further purification.

^{1}H NMR (400 MHz, $CDCl_3$): δ = 8.86 (s, 1H, N*H*), 5.94 (ddt, J = 16.5, 10.8, 5.8 Hz, 1H, vinyl*H*), 5.44 (dt, J = 17.1, 1.4 Hz, 1H, vinyl*H*), 5.36 (d, J = 10.4 Hz, 1H, vinyl*H*), 4.80 (dd, J = 5.8, 1.4 Hz, 2H, C*H2*).

2,2,2-Trichloroethyl (chlorosulfonyl)carbamate

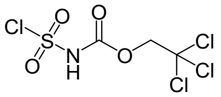

Following a modified version of a reported procedure,[3] to a solution of 2,2,2-trichloroethanol (1.22 g, 8.19 mmol, 1.0 equiv.) in 10 mL DCM was added chlorosulfonyl isocyanate (1.16 g, 8.19 mmol, 1.0 equiv.) dropwise. The reaction mixture was stirred at room temperature for 1 hour and then solvent was removed *in vacuo*. 2,2,2-Trichloroethyl (chlorosulfonyl)carbamate (2.29 g, 7.87 mmol, 96%) was obtained as a white solid, which was stored at -18 °C and used without further purification.

^{1}H NMR (400 MHz, $CDCl_3$): δ = 8.78 (s, 1H, N*H*), 4.90 (d, J = 1.0 Hz, 2H, C*H2*).

Benzoylsulfamoyl chloride

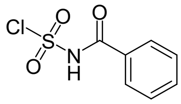

Following a modified version of a reported procedure,[4] benzoic acid (1.00 g, 8.19 mmol, 1.0 equiv.) and chlorosulfonyl isocyanate (1.16 g, 8.19 mmol, 1.0 equiv.) was used. Benzoylsulfamoyl chloride (1.70 g, 7.74 mmol, 95%) was obtained as a white solid, which was used without further purification.

***tert*-Butyl (fluorosulfonyl)carbamate**

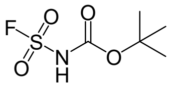

Following a reported procedure,[5] to a solution of *tert*-butyl (chlorosulfonyl)carbamate (539 mg, 2.50 mmol, 1.0 equiv.) in 5 mL MeCN was added potassium hydrogenfluoride (234 mg, 3.00 mmol, 1.2 equiv.). The reaction mixture was stirred at room temperature for 2 hours before being quenched by water and extracted with Et2O (20 mL x 3). The organic phases were combined, dried over Na_2SO_4 and evaporated *in vacuo*. *tert*-Butyl (fluorosulfonyl)carbamate (448 mg, 2.25 mmol, 90%) was obtained as a white solid, which was stored at -18 °C and used without further purification.

^{1}H NMR (400 MHz, $CDCl_3$): δ = 8.78 (s, 1H, N*H*), 4.90 (d, J = 1.0 Hz, 2H, C*H2*);

3 Nicolaou, K. C.; Snyder, S. A.; Longbottom, D. A.; Nalbandian, A. Z.; Huang, X. *Chem. Eur. J.* **2004**, *10*, 5581-5606.

4 A. Dorlars, in Methoden der Organischen Chemie (Huuben- Weyl), Georg Thieme Verlag, Stuttgart, 1958, vol. 11/2, p. 693-700.

5 Wata, C.; Hashimoto, T. *J. Am. Chem. Soc.* **2021**, *143*, 1745-1751.

^{19}F NMR (376 MHz, $CDCl_3$): δ = 52.9.

^{1}H and ^{19}F NMR data correspond to the reported values.[5]

***tert*-Butyl (*tert*-butoxycarbonyl)(chlorosulfonyl)carbamate**

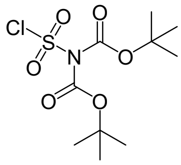

To a suspension of NaH (60% dispersion in mineral oil, 200 mg, 5.00 mmol, 1.0 equiv.) in 5 mL Et_2O was added a solution of di-*tert*-butyl iminodicarbonate (1.08 g, 5.00 mmol, 1.0 equiv.) in 5 mL Et_2O. After stirring at room temperature for 30 min, sulfuryl chloride (675 mg, 5.00 mmol, 1.0 equiv.) was added dropwise. The reaction mixture was further stirred at room temperature overnight before being filtered. The filtrate was concentrated *in vacuo* to give *tert*-Butyl (fluorosulfonyl)carbamate (1.58 g, 5.00 mmol, >99%) as a yellow oil, which was used without further purification.

^{1}H NMR (400 MHz, $CDCl_3$): δ = 1.48 (s, 9H, C(C*H*3)3), 1.45 (s, 9H, C(C*H*3)3);
^{13}C NMR (101 MHz, $CDCl_3$): δ = 156.2, 149.7, 82.0, 79.7, 28.3, 28.2, 28.0 (one extra signal due to rotamers).

4.2 Synthesis of sulfonamides

General Procedure A (GP A):

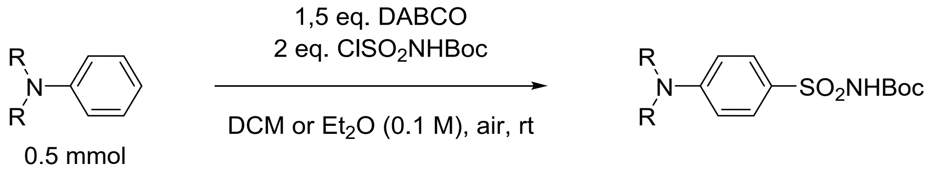

To a vial containing a solution of aniline derivative (0.500 mmol, 1.0 equiv.) and 1,4-diazabicyclo[2.2.2]octane (DABCO) (84.1 mg, 0.750 mmol, 1.5 equiv.) in dichloromethane (2.5 mL) was added a solution of *N*-(*tert*-butoxycarbonyl)sulfamoyl chloride ($ClSO_2NHBoc$) (216 mg, 1.00 mmol, 2.0 equiv.) in dichloromethane (2.5 mL) at room temperature under air. Upon addition of $ClSO_2NHBoc$, white precipitate was formed immediately. The suspension was kept stirring for 16 hours before being quenched by the addition of saturated $NaHCO_3$ (2 mL). The aqueous layer was then extracted with dichloromethane (3 x 10 mL). The organic phases were combined, dried over Na_2SO_4, filtered and concentrated *in vacuo*. The pure product was obtained after column chromatography on Biotage (SiO_2 12 g, eluent with 0 - 35% ethyl acetate in n-Hexane, linear gradient).

Note: the reactions for preparing **4a**, **4b**, **4d**, **4e**, **4m** and **4o** was performed in Et_2O, in order to achieve a better regioselectivity.

***tert*-Butyl ((4-(dimethylamino)phenyl)sulfonyl)carbamate** (**4a**)

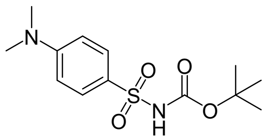

Following GP A, starting from *N*,*N*-dimethylaniline **3a** (60.6 mg, 0.500 mmol) and using Et_2O as solvent, *tert*-butyl ((4-(dimethylamino)phenyl)sulfonyl)carbamate **4a** (129 mg, 0.431 mmol, 86%) and *tert*-butyl ((2-(dimethylamino)phenyl)sulfonyl)carbamate **4a'** (7.6 mg, 0.025 mmol, 5%) were both obtained as a white solid.

R_f: 0.27 (silica, pentanes:ethyl acetate 2:1);
^{1}H NMR (400 MHz, $CDCl_3$): δ = 7.86 – 7.75 (m, 2H, Ar*H*), 6.71 – 6.62 (m, 2H, Ar*H*), 3.06 (s, 6H, NCH_3), 1.39 (s, 9H, CH_3);
^{13}C NMR (101 MHz, $CDCl_3$): δ = 153.4, 149.4, 130.1, 123.7, 110.5, 83.4, 40.1, 27.9;
HRMS (ESI) calcd. for $C_{13}H_{21}N_2O_4S$ $[M+H]^+$ 301.1218; Found 301.1217.
***tert*-Butyl ((2-(dimethylamino)phenyl)sulfonyl)carbamate** (**4a'**)

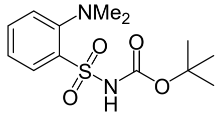

R_f: 0.45 (silica, pentanes:ethyl acetate 2:1);
^{1}H NMR (400 MHz, $CDCl_3$): δ = 8.08 (dd, *J* = 8.0, 1.6 Hz, 1H, ArH), 7.62 (ddd, *J* = 8.0, 7.3, 1.6 Hz, 1H, ArH), 7.45 (dd, *J* = 8.1, 1.2 Hz, 1H, ArH), 7.32 (ddd, *J* = 7.9, 7.4, 1.2 Hz, 1H, ArH), 2.76 (s, 6H, NCH3), 1.28 (s, 9H, CH3);
^{13}C NMR (101 MHz, $CDCl_3$): δ = 153.2, 149.6, 135.5, 134.7, 131.0, 125.3, 123.8, 83.5, 46.4, 27.7;
HRMS (ESI) calcd. for $C_{13}H_{21}N_2O_4S$ $[M+H]^+$ 301.1221; Found 301.1217.

Gram-scale synthesis of 4a: To a 100 mL round-bottom flask charged with **3a** (606 mg, 5.00 mmol), DABCO (841 mg, 7.50 mmol, 1.5 equiv.) and Et_2O (25 mL) was added a solution of $ClSO_2NHBoc$ (2.16 g, 10.0 mmol, 2.0 equiv.) in Et_2O (25 mL) at room temperature under air. After stirring for 16 hours, the solvent was evaporated before the addition of saturated $NaHCO_3$ (20 mL). The aqueous layer was then extracted with dichloromethane (3 x 50 mL). The organic phases were combined, dried over Na_2SO_4, filtered and concentrated *in vacuo*. **4a** (1.27 g, 4.23 mmol, 85%) was obtained after column chromatography on Biotage (SiO_2 25 g, eluent with 0 - 35% ethyl acetate in n-Hexane, linear gradient).

***tert*-Butyl ((4-(diethylamino)phenyl)sulfonyl)carbamate** (**4b**)

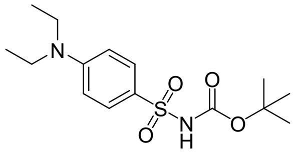

Following GP A, starting from *N*,*N*-diethylaniline (74.6 mg, 0.500 mmol) and using Et_2O as solvent, *tert*-butyl ((4-(diethylamino)phenyl)sulfonyl)carbamate **4b** (160 mg, 0.487 mmol, 97%) was obtained as a white solid.

R_f: 0.50 (silica, pentanes:ethyl acetate 2:1);
^{1}H NMR (400 MHz, $CDCl_3$): δ = 7.82 – 7.70 (m, 2H, Ar*H*), 7.23 (s, 1H, N*H*), 6.70 – 6.51 (m, 2H, Ar*H*), 3.41 (q, J = 7.1 Hz, 4H, NC*H*2), 1.40 (s, 9H, C*H*3), 1.20 (t, J = 7.1 Hz, 6H, NCH2C*H*3);
^{13}C NMR (101 MHz, $CDCl_3$): δ = 151.2, 149.5, 130.4, 122.8, 110.1, 83.4, 44.7, 28.0, 12.3;

HRMS (ESI) calcd. for $C_{15}H_{25}N_2O_4S$ $[M+H]^+$ 329.1530; Found 329.1525.

***tert*-Butyl ((4-(dibenzylamino)phenyl)sulfonyl)carbamate** (**4c**)

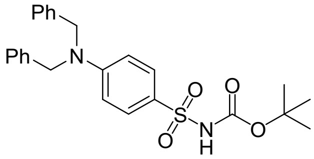

Following GP A, starting from *N*,*N*-bis(phenylmethyl)aniline (137 mg, 0.500 mmol) and using DCM as solvent, *tert*-butyl ((4-(dibenzylamino)phenyl)sulfonyl)carbamate **4c** (225 mg, 0.497 mmol, 99%) was obtained as a colorless oil.

R_f: 0.55 (silica, pentanes:ethyl acetate 2:1);
^{1}H NMR (400 MHz, $CDCl_3$): δ = 7.80 – 7.71 (m, 2H, Ar*H*), 7.39 – 7.31 (m, 4H, Ar*H*), 7.31 – 7.26 (m, 2H, Ar*H*), 7.23 – 7.17 (m, 4H, Ar*H*), 6.80 – 6.71 (m, 2H, Ar*H*), 4.73 (s, 4H, NC*H*2), 1.38 (s, 9H, C*H*3);
^{13}C NMR (101 MHz, $CDCl_3$): δ = 152.8, 149.3, 136.7, 136.7, 130.3, 128.9, 127.4, 126.4, 124.9, 111.3, 83.6, 54.2, 27.9;
HRMS (ESI) calcd. for $C_{26}H_{29}NO_4S$ $[M+H]^+$ 453.1840; Found 453.1843.

***tert*-Butyl ((4-(pyrrolidin-1-yl)phenyl)sulfonyl)carbamate** (**4d**)

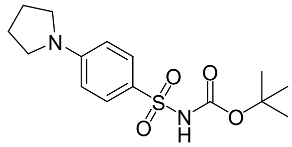

Following GP A, starting from 1-phenylpyrrolidine (73.6 mg, 0.500 mmol) and using Et_2O as solvent, *tert*-butyl ((4-(pyrrolidin-1-yl)phenyl)sulfonyl)carbamate **4d** (146 mg, 0.447 mmol, 89%) was obtained as a white solid.

R_f: 0.43 (silica, pentanes:ethyl acetate 2:1);
^{1}H NMR (400 MHz, $CDCl_3$): δ = 7.86 – 7.72 (m, 2H, Ar*H*), 7.28 (s, 1H, N*H*), 6.60 – 6.45 (m, 2H, Ar*H*), 3.43 – 3.27 (m, 4H, NC*H*2), 2.10 – 1.99 (m, 4H, C*H*2), 1.39 (s, 9H, C*H*3);
^{13}C NMR (101 MHz, $CDCl_3$): δ = 151.0, 149.5, 130.2, 122.8, 110.5, 83.3, 47.6, 27.9, 25.4;
HRMS (ESI) calcd. for $C_{15}H_{23}N_2O_4S$ $[M+H]^+$ 327.1370; Found 327.1373.

***tert*-Butyl ((4-(piperidin-1-yl)phenyl)sulfonyl)carbamate** (**4e**)

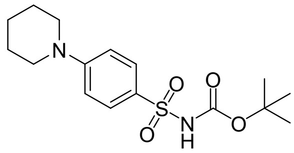

Following GP A, starting from 1-phenylpiperidine (80.6 mg, 0.500 mmol) and using Et_2O as solvent, *tert*-butyl ((4-(piperidin-1-yl)phenyl)sulfonyl)carbamate **4e** (153 mg, 0.448 mmol, 90%) was obtained as a white solid.

R_f: 0.53 (silica, pentanes:ethyl acetate 2:1);
^{1}H NMR (400 MHz, $CDCl_3$): δ = 7.85 – 7.74 (m, 2H, Ar*H*), 7.16 (s, 1H, N*H*), 6.93 – 6.84 (m, 2H, Ar*H*), 3.37 (t, J = 4.3 Hz, 4H, NC*H*2), 1.72 – 1.62 (m, 6H, C*H*2), 1.40 (s, 9H, C*H*3);

^{13}**C NMR** (101 MHz, $CDCl_3$): δ = 154.3, 149.3, 130.1, 125.6, 113.3, 83.6, 48.7, 27.9, 25.2, 24.2;
HRMS (ESI) calcd. for $C_{16}H_{25}N_2O_4S$ $[M+H]^+$ 341.1530; Found 341.1531.

***tert*-Butyl ((4-morpholinophenyl)sulfonyl)carbamate** (**4f**)

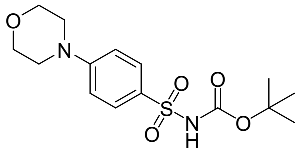

Following GP A, starting from 4-phenylmorpholine (81.6 mg, 0.500 mmol) and using DCM as solvent, *tert*-butyl ((4-morpholinophenyl)sulfonyl)carbamate **4f** (144 mg, 0.420 mmol, 84%) was obtained as a white solid.

R$_f$: 0.41 (silica, pentanes:ethyl acetate 1:1);
^{1}**H NMR** (400 MHz, $CDCl_3$): δ = 7.91 – 7.82 (m, 2H, Ar*H*), 7.14 (s, 1H, N*H*), 6.95 – 6.86 (m, 2H, Ar*H*), 3.90 – 3.82 (m, 4H, OC*H*2), 3.37 – 3.28 (m, 4H, NC*H*2), 1.40 (s, 9H, C*H*3);
^{13}**C NMR** (101 MHz, $CDCl_3$): δ = 154.4, 149.2, 130.1, 127.3, 113.3, 83.7, 66.4, 47.4, 27.9;
HRMS (ESI) calcd. for $C_{15}H_{23}N_2O_5S$ $[M+H]^+$ 343.1322; Found 343.1326.

((4-(N-(*tert*-Butoxycarbonyl)sulfamoyl)phenyl)azanediyl)bis(ethane-2,1-diyl) diacetate (**4g**)

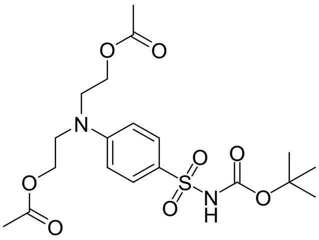

Following GP A, starting from (phenylazanediyl)bis(ethane-2,1-diyl) diacetate (133 mg, 0.500 mmol) and using DCM as solvent, ((4-(N-(*tert*-Butoxycarbonyl)sulfamoyl)phenyl)azanediyl)bis(ethane-2,1-diyl) diacetate **4g** (214 mg, 0.481 mmol, 96%) was obtained as a colorless oil.

R$_f$: 0.31 (silica, pentanes:ethyl acetate 1:1);
^{1}**H NMR** (400 MHz, $CDCl_3$): δ = 7.90 – 7.76 (m, 2H, Ar*H*), 7.17 (s, 1H, N*H*), 6.82 – 6.73 (m, 2H, Ar*H*), 4.26 (t, J = 6.2 Hz, 4H, OC*H*2), 3.69 (t, J = 6.2 Hz, 4H, NC*H*2), 2.04 (s, 6H, C*H*3), 1.40 (s, 9H, C*H*3);
^{13}**C NMR** (101 MHz, $CDCl_3$): δ = 170.8, 151.3, 149.2, 130.4, 125.2, 110.8, 83.6, 60.8, 49.5, 27.9, 20.8;
HRMS (ESI) calcd. for $C_{19}H_{29}N_2O_8S$ $[M+H]^+$ 445.1639; Found 445.1643.

Diethyl 2,2'-((4-(*N*-(*tert*-butoxycarbonyl)sulfamoyl)phenyl)azanediyl)diacetate (**4h**)

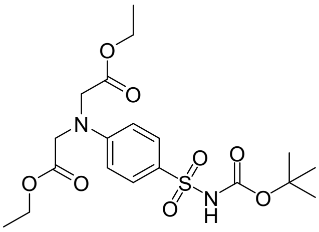

Following GP A, starting from diethyl 2,2'-(phenylazanediyl)diacetate (133 mg, 0.500 mmol) and using DCM as solvent, diethyl 2,2'-((4-(*N*-(*tert*-butoxycarbonyl)sulfamoyl)phenyl)azanediyl)diacetate **4h** (211

mg, 0.475 mmol, 95%) was obtained as a colorless oil.

R$_f$: 0.58 (silica, pentanes:ethyl acetate 2:3);
^{1}H NMR (400 MHz, $CDCl_3$): δ = 7.87 – 7.79 (m, 2H, Ar*H*), 6.67 – 6.59 (m, 2H, Ar*H*), 4.23 (q, J = 7.1 Hz, 4H, OC*H*2), 4.18 (s, 4H, NC*H*2), 1.39 (s, 9H, C*H*3), 1.28 (t, J = 7.2 Hz, 6H, OCH2C*H*3);
^{13}C NMR (101 MHz, $CDCl_3$): δ = 169.7, 151.7, 149.2, 130.3, 126.8, 111.4, 83.6, 61.6, 53.3, 27.9, 14.2;
HRMS (ESI) calcd. for $C_{19}H_{28}N_2O_8SNa$ $[M+Na]^+$ 467.1459; Found 467.1468.

***tert*-Butyl ((4-(methyl(phenyl)amino)phenyl)sulfonyl)carbamate** (**4i**)

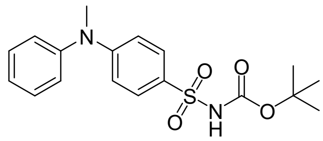

Following GP A, starting from *N*-methyldiphenylamine (91.6 mg, 0.500 mmol) and using Et_2O as solvent, *tert*-butyl ((4-(methyl(phenyl)amino)phenyl)sulfonyl)carbamate **4i** (168 mg, 0.462 mmol, 92%) was obtained as a colorless oil.

R$_f$: 0.50 (silica, pentanes:ethyl acetate 3:2);
^{1}H NMR (400 MHz, $CDCl_3$): δ = 7.80 – 7.73 (m, 2H, Ar*H*), 7.62 (s, 1H, N*H*), 7.46 – 7.39 (m, 2H, Ar*H*), 7.28 – 7.23 (m, 1H, Ar*H*), 7.23 – 7.17 (m, 2H, Ar*H*), 6.78 – 6.71 (m, 2H, Ar*H*), 3.36 (s, 3H, NC*H*3), 1.40 (s, 9H, C*H*3);
^{13}C NMR (101 MHz, $CDCl_3$): δ = 152.8, 149.5, 146.8, 130.0, 129.8, 126.4, 126.2, 125.7, 112.9, 83.4, 40.2, 27.9;
HRMS (ESI) calcd. for $C_{18}H_{23}N_2O_4S$ $[M+H]^+$ 363.1373; Found 363.1377.

***tert*-Butyl ((4-(diphenylamino)phenyl)sulfonyl)carbamate** (**4j**)

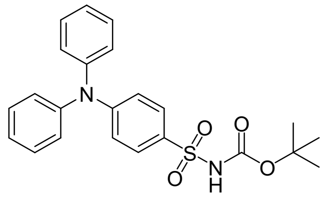

Following GP A, starting from triphenylamine (123 mg, 0.500 mmol) and using DCM as solvent, *tert*-butyl ((4-(diphenylamino)phenyl)sulfonyl)carbamate **4j** (193 mg, 0.455 mmol, 91%) was obtained as a colorless oil.

R$_f$: 0.48 (silica, pentanes:ethyl acetate 3:1);
^{1}H NMR (400 MHz, $CDCl_3$): δ = 7.79 – 7.72 (m, 2H, Ar*H*), 7.34 (dd, J = 8.9, 6.9 Hz, 4H, Ar*H*), 7.21 – 7.13 (m, 7H, Ar*H* + N*H*), 7.02 – 6.96 (m, 2H, Ar*H*), 1.42 (s, 9H, C*H*3);
^{13}C NMR (101 MHz, $CDCl_3$): δ = 152.6, 149.2, 149.1, 146.0, 129.8, 129.7, 128.7, 126.2, 125.2, 118.8, 83.8, 27.9;
HRMS (ESI) calcd. for $C_{23}H_{24}N_2O_4SNa$ $[M+Na]^+$ 447.1349; Found 447.1357.

***tert*-Butyl ((4-(dimethylamino)-3-methylphenyl)sulfonyl)carbamate** (**4k**)

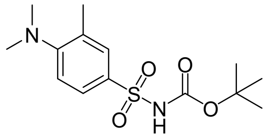

Following GP A, starting from *N*,*N*,2-trimethylaniline (67.6 mg, 0.500 mmol) and using DCM as solvent, *tert*-butyl ((4-(dimethylamino)-3-methylphenyl)sulfonyl)carbamate **4k** (136 mg, 0.433 mmol, 87%) was obtained as a white solid.

R_f: 0.50 (silica, pentanes:ethyl acetate 3:2);
^{1}H NMR (400 MHz, $CDCl_3$): δ = 7.80 – 7.70 (m, 2H, Ar*H*), 7.21 (s, 1H, N*H*), 7.01 (d, J = 8.5 Hz, 1H, Ar*H*), 2.81 (s, 6H, NC*H*3), 2.37 (s, 3H, C*H*3), 1.39 (s, 9H, C*H*3);
^{13}C NMR (101 MHz, $CDCl_3$): δ = 157.3, 149.2, 131.2, 130.8, 130.4, 127.2, 117.3, 83.8, 43.3, 27.9, 19.5;
HRMS (ESI) calcd. for $C_{14}H_{23}N_2O_4S$ $[M+H]^+$ 315.1373; Found 315.1374.

***tert*-Butyl ((4-(dimethylamino)-3,5-dimethylphenyl)sulfonyl)carbamate** (**4l**)

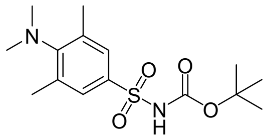

Following GP A, starting from *N,N,*2,6-tetramethylaniline (74.6 mg, 0.500 mmol) and using DCM as solvent, *tert*-butyl ((4-(dimethylamino)-3,5-dimethylphenyl)sulfonyl)carbamate **4l** (16.0 mg, 0.049 mmol, 10%) was obtained as a white solid.

R_f: 0.50 (silica, pentanes:ethyl acetate 3:1);
^{1}H NMR (400 MHz, $CDCl_3$): δ = 7.60 (s, 2H, Ar*H*), 7.18 (s, 1H, N*H*), 2.85 (s, 6H, NC*H*3), 2.33 (s, 6H, C*H*3), 1.40 (s, 9H, C*H*3);
^{13}C NMR (101 MHz, $CDCl_3$): δ = 155.0, 149.1, 136.8, 133.3, 128.6, 83.9, 42.3, 27.9, 19.6;
HRMS (ESI) calcd. for $C_{15}H_{25}N_2O_4S$ $[M+H]^+$ 329.1530; Found 329.1534.

***tert*-Butyl ((4-(dimethylamino)-2-methylphenyl)sulfonyl)carbamate** (**4m**)

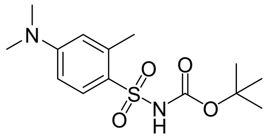

Following GP A, starting from *N*,*N*,3-trimethylaniline (67.6 mg, 0.500 mmol) and using Et_2O as solvent, *tert*-butyl ((4-(dimethylamino)-2-methylphenyl)sulfonyl)carbamate **4m** (144 mg, 0.457 mmol, 91%) was obtained as a white solid.

R_f: 0.31 (silica, pentanes:ethyl acetate 3:2);
^{1}H NMR (400 MHz, $CDCl_3$): δ = 7.92 (d, *J* = 9.0 Hz, 1H, Ar*H*), 7.19 (s, 1H, N*H*), 6.54 (dd, *J* = 9.0, 2.7 Hz, 1H, Ar*H*), 6.49 (dd, *J* = 2.6, 0.9 Hz, 1H, Ar*H*), 3.05 (s, 6H, NC*H*3), 2.58 (s, 3H, C*H*3), 1.36 (s, 9H, C*H*3);
^{13}C NMR (101 MHz, $CDCl_3$): δ = 153.3, 149.3, 139.0, 133.4, 122.3, 114.1, 108.2, 83.4, 40.1, 27.9, 20.9;
HRMS (ESI) calcd. for $C_{14}H_{23}N_2O_4S$ $[M+H]^+$ 315.1373; Found 315.1371.

2-(*N*-(*tert*-butoxycarbonyl)sulfamoyl)-5-(dimethylamino)phenyl acetate (**4n**)

Following GP A, starting from 3-(dimethylamino)phenyl acetate (89.6 mg, 0.500 mmol) and using DCM as solvent, 2-(*N*-(*tert*-butoxycarbonyl)sulfamoyl)-5-(dimethylamino)phenyl acetate **4n** (95.0 mg, 0.265

mmol, 53%) was obtained as a white solid, together with 4-(*N*-(*tert*-butoxycarbonyl)sulfamoyl)-3-(dimethylamino)phenyl acetate **4n'** (85.0 mg, 0.237 mmol, 47%) as a white solid.

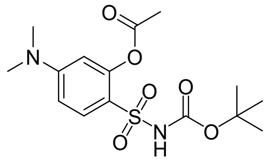

R$_f$: 0.33 (silica, pentanes:ethyl acetate 1:1);
^{1}H NMR (400 MHz, $CDCl_3$): δ = 7.88 (d, J = 9.0 Hz, 1H, Ar*H*), 7.42 (s, 1H, N*H*), 6.54 (dd, J = 9.1, 2.6 Hz, 1H, Ar*H*), 6.35 (d, J = 2.6 Hz, 1H, Ar*H*), 3.05 (s, 6H, NC*H*3), 2.34 (s, 3H, COC*H*3), 1.38 (s, 9H, C*H*3);
^{13}C NMR (101 MHz, $CDCl_3$): δ = 168.9, 154.7, 149.5, 149.3, 133.2, 115.6, 108.0, 106.3, 83.6, 40.1, 27.9, 21.1;
HRMS (ESI) calcd. for $C_{15}H_{23}N_2O_6S$ $[M+H]^+$ 359.1271; Found 359.1266.

4-(*N*-(*tert*-Butoxycarbonyl)sulfamoyl)-3-(dimethylamino)phenyl acetate (4n')

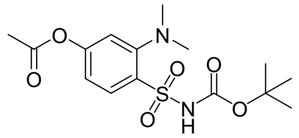

R$_f$: 0.53 (silica, pentanes:ethyl acetate 3:2);
^{1}H NMR (400 MHz, $CDCl_3$): δ = 8.10 (d, J = 8.7 Hz, 1H, Ar*H*), 7.97 – 7.61 (br, 1H, N*H*), 7.18 (d, J = 2.2 Hz, 1H, Ar*H*), 7.06 (dd, J = 8.7, 2.2 Hz, 1H, Ar*H*), 2.75 (s, 6H, NC*H*3), 2.32 (s, 3H, COC*H*3), 1.29 (s, 9H, C*H*3);
^{13}C NMR (101 MHz, $CDCl_3$): δ = 168.4, 155.4, 154.7, 149.4, 132.6, 132.4, 118.3, 117.1, 83.8, 46.3, 27.7, 21.1;
HRMS (ESI) calcd. for $C_{15}H_{23}N_2O_6S$ $[M+H]^+$ 359.1271; Found 359.1267.

***tert*-Butyl ((4-(dimethylamino)-2-methoxyphenyl)sulfonyl)carbamate** (**4o**)

Following GP A, starting from 3-methoxy-*N,N*-dimethylaniline (75.6 mg, 0.500 mmol) and using Et_2O as solvent, *tert*-butyl ((4-(dimethylamino)-2-methoxyphenyl)sulfonyl)carbamate **4o** (73.0 mg, 0.221 mmol, 44%) was obtained as a white solid, together with *tert*-butyl ((2-(dimethylamino)-4-methoxyphenyl)sulfonyl)carbamate **4o'** (92.0 mg, 0.278 mmol, 56%) as a white solid.

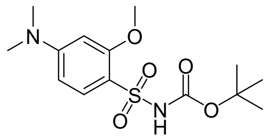

R$_f$: 0.33 (silica, pentanes:ethyl acetate 1:1);
^{1}H NMR (400 MHz, $CDCl_3$): δ = 7.78 (d, J = 9.0 Hz, 1H, Ar*H*), 7.31 (s, 1H, N*H*), 6.28 (dd, J = 9.0, 2.3 Hz, 1H, Ar*H*), 6.12 (d, J = 2.3 Hz, 1H, Ar*H*), 3.93 (s, 3H, OC*H*3), 3.06 (s, 6H, NC*H*3), 1.33 (s, 9H, C*H*3);
^{13}C NMR (101 MHz, $CDCl_3$): δ = 158.4, 155.4, 149.6, 133.1, 112.4, 103.2, 94.3, 83.0, 56.0, 40.2, 27.8;
HRMS (ESI) calcd. for $C_{14}H_{23}N_2O_5S$ $[M+H]^+$ 331.1322; Found 331.1322.

***tert*-Butyl ((2-(dimethylamino)-4-methoxyphenyl)sulfonyl)carbamate (4o')**

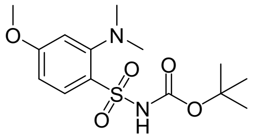

R$_f$: 0.53 (silica, pentanes:ethyl acetate 3:2);
^{1}H NMR (400 MHz, $CDCl_3$): δ = 8.02 (d, J = 8.9 Hz, 1H, Ar*H*), 7.96 – 7.66 (br, 1H, N*H*), 6.89 (d, J = 2.5 Hz, 1H, Ar*H*), 6.78 (dd, J = 8.9, 2.5 Hz, 1H, Ar*H*), 3.87 (s, 3H, OC*H*3), 2.74 (s, 6H, NC*H*3), 1.31 (s, 9H, C*H*3);
^{13}C NMR (101 MHz, $CDCl_3$): δ = 164.6, 155.1, 149.6, 133.2, 127.1, 110.0, 109.3, 83.2, 55.7, 46.3, 27.8;

HRMS (ESI) calcd. for $C_{14}H_{23}N_2O_5S$ $[M+H]^+$ 331.1322; Found 331.1327.

***tert*-Butyl ((4-(dimethylamino)-2-(trifluoromethoxy)phenyl)sulfonyl)carbamate** (**4p**)

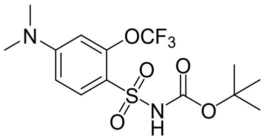

Following GP A, starting from *N*,*N*-dimethyl-3-(trifluoromethoxy)aniline (103 mg, 0.500 mmol) and using DCM as solvent, *tert*-butyl ((4-(dimethylamino)-2-(trifluoromethoxy)phenyl)sulfonyl)carbamate **4p** (151 mg, 0.392 mmol, 78%) was obtained as a beige solid.

R_f: 0.30 (silica, pentanes:ethyl acetate 3:1);
^{1}H NMR (400 MHz, $CDCl_3$): δ = 7.90 (d, J = 9.1 Hz, 1H, Ar*H*), 7.31 (s, 1H, N*H*), 6.56 (dd, J = 9.1, 2.5 Hz, 1H, Ar*H*), 6.52 (dd, J = 2.6, 1.4 Hz, 1H, Ar*H*), 3.07 (s, 6H, NC*H*3), 1.37 (s, 9H, C*H*3);
^{13}C NMR (101 MHz, $CDCl_3$): δ = 154.7, 149.0, 147.7 (q, J = 1.7 Hz), 133.6, 120.3 (q, J = 260.1 Hz), 115.6, 108.1, 102.2 (q, *J* = 1.9 Hz), 83.6, 40.1, 27.8;
^{19}F NMR (376 MHz, $CDCl_3$): δ = -55.8;
HRMS (ESI) calcd. for $C_{14}H_{19}N_2O_5F_3S$ $[M+H]^+$ 385.1040; Found 385.1040.

***tert*-Butyl ((4-(dimethylamino)-2-ethynylphenyl)sulfonyl)carbamate** (**4q**)

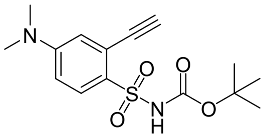

Following GP A, starting from 2-ethynyl-*N*,*N*-dimethylaniline (72.6 mg, 0.500 mmol) and using DCM as solvent, *tert*-butyl ((4-(dimethylamino)-2-ethynylphenyl)sulfonyl)carbamate **4q** (139 mg, 0.429 mmol, 86%) was obtained as a beige solid.

R_f: 0.34 (silica, pentanes:ethyl acetate 3:2);
^{1}H NMR (400 MHz, $CDCl_3$): δ = 7.93 (d, J = 9.1 Hz, 1H, Ar*H*), 7.40 (s, 1H, N*H*), 6.88 (d, J = 2.8 Hz, 1H, Ar*H*), 6.65 (dd, J = 9.1, 2.7 Hz, 1H, Ar*H*), 3.49 (s, 1H, C≡C*H*), 3.06 (s, 6H, NC*H*3), 1.35 (s, 9H, C*H*3);
^{13}C NMR (101 MHz, $CDCl_3$): δ = 152.5, 149.2, 132.6, 125.4, 121.2, 117.2, 110.3, 84.0, 83.5, 80.3, 40.0, 27.8;
HRMS (ESI) calcd. for $C_{15}H_{21}N_2O_4S$ $[M+H]^+$ 325.1219; Found 325.1217.

***tert*-Butyl ((4-(dimethylamino)-2-(trifluoromethyl)phenyl)sulfonyl)carbamate** (**4r**)

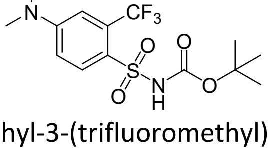

Following GP A, starting from *N*,*N*-dimethyl-3-(trifluoromethyl)aniline (94.6 mg, 0.500 mmol) and using DCM as solvent, *tert*-butyl ((4-(dimethylamino)-2-(trifluoromethyl)phenyl)sulfonyl)carbamate **4r** (65.3 mg, 0.177 mmol, 35%) was obtained as a white solid.

R_f: 0.27 (silica, pentanes:ethyl acetate 3:1);
^{1}H NMR (400 MHz, $CDCl_3$): δ = 8.18 (d, J = 9.1 Hz, 1H, Ar*H*), 7.28 (s, 1H, N*H*), 7.00 (d, J = 2.7 Hz, 1H, Ar*H*), 6.76 (dd, J = 9.2, 2.8 Hz, 1H, Ar*H*), 3.11 (s, 6H, NC*H*3), 1.38 (s, 9H, C*H*3);

^{13}C NMR (101 MHz, $CDCl_3$): δ = 152.7, 149.0, 135.9, 128.9 (t, J = 32.4 Hz), 120.9, 122.9 (q, J = 274.0 Hz), 111.8, 110.4 (q, J = 6.7 Hz), 83.6, 40.0, 27.9;
^{19}F NMR (376 MHz, $CDCl_3$): δ = -58.42;
HRMS (ESI) calcd. for $C_{14}H_{19}N_2O_4F_3S$ $[M+H]^+$ 369.1090; Found 369.1092.

***tert*-Butyl ((4-(dimethylamino)-2-nitrophenyl)sulfonyl)carbamate** (**4s**)

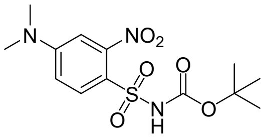

Following GP A, starting from *N,N*-dimethyl-3-nitroaniline (83.1 mg, 0.500 mmol) and using DCM as solvent, *tert*-butyl ((4-(dimethylamino)-2-nitrophenyl)sulfonyl)carbamate **4s** (33.2 mg, 96.1 umol, 19%) was obtained as a bright yellow solid.

R_f: 0.41 (silica, pentanes:ethyl acetate 3:2);
^{1}H NMR (400 MHz, $CDCl_3$): δ = 8.06 (d, J = 9.1 Hz, 1H, Ar*H*), 6.96 (d, J = 2.7 Hz, 1H, Ar*H*), 6.76 (dd, J = 9.1, 2.7 Hz, 1H, Ar*H*), 3.12 (s, 6H, NC*H*3), 1.43 (s, 9H, C*H*3);
^{13}C NMR (101 MHz, $CDCl_3$): δ = 153.4, 149.9, 149.1, 134.7, 116.1, 112.2, 106.9, 83.9, 40.2, 27.9;
HRMS (ESI) calcd. for $C_{13}H_{19}N_3O_6SNa$ $[M+Na]^+$ 368.0887; Found 368.0883.

***tert*-Butyl ((4-(dimethylamino)-2-fluorophenyl)sulfonyl)carbamate** (**4t**)

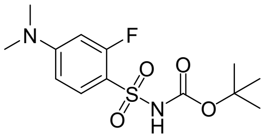

Following GP A, starting from 3-fluoro-*N,N*-dimethylaniline (69.6 mg, 0.500 mmol) and using DCM as solvent, *tert*-butyl ((4-(dimethylamino)-2-fluorophenyl)sulfonyl)carbamate **4t** (123 mg, 0.386 mmol, 77%) was obtained as a white solid.

R_f: 0.42 (silica, pentanes:ethyl acetate 3:2);
^{1}H NMR (400 MHz, $CDCl_3$): δ = 7.76 (t, J = 8.8 Hz, 1H, Ar*H*), 7.35 (s, 1H, N*H*), 6.44 (dd, J = 9.1, 2.5 Hz, 1H, Ar*H*), 6.34 (dd, J = 14.2, 2.5 Hz, 1H, Ar*H*), 3.05 (s, 6H, NC*H*3), 1.38 (s, 9H, C*H*3);
^{13}C NMR (101 MHz, $CDCl_3$): δ = 160.6 (d, J = 252.6 Hz), 155.6 (d, J = 11.7 Hz), 149.1, 132.8 (d, J = 1.7 Hz), 111.4 (d, J = 13.5 Hz), 106.3 (d, J = 1.8 Hz), 98.3 (d, J = 25.5 Hz), 83.6, 40.1, 27.8;
^{19}F NMR (376 MHz, $CDCl_3$): δ = -108.7 (dd, J = 14.2, 8.6 Hz);
HRMS (ESI) calcd. for C13H19N2O4FS $[M+H]^+$ 319.1122; Found 319.1125.

***tert*-Butyl ((4-(dimethylamino)-2-chlorophenyl)sulfonyl)carbamate** (**4u**)

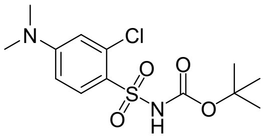

Following GP A, starting from 3-chloro-*N,N*-dimethylaniline (77.8 mg, 0.500 mmol) and using DCM as solvent, *tert*-butyl ((4-(dimethylamino)-2-chlorophenyl)sulfonyl)carbamate **4u** (137 mg, 0.409 mmol, 82%) was obtained as a white solid.

R_f: 0.34 (silica, pentanes:ethyl acetate 3:2);

^{1}**H NMR** (400 MHz, $CDCl_3$): δ = 7.96 (d, J = 9.1 Hz, 1H, Ar*H*), 7.38 (s, 1H, N*H*), 6.69 (d, J = 2.6 Hz, 1H, Ar*H*), 6.57 (dd, J = 9.1, 2.6 Hz, 1H, Ar*H*), 3.06 (s, 6H, NC*H*3), 1.36 (s, 9H, C*H*3);
^{13}**C NMR** (101 MHz, $CDCl_3$): δ = 153.8, 149.0, 134.1, 133.3, 120.9, 113.0, 108.7, 83.6, 40.0, 27.8;
HRMS (ESI) calcd. for $C_{13}H_{19}N_2O_4ClS$ $[M+H]^+$ 335.0827; Found 335.0815.

***tert*-Butyl ((4-(dimethylamino)-2-bromophenyl)sulfonyl)carbamate** (**4v**)

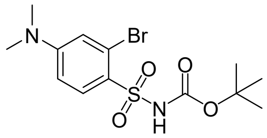

Following GP A, starting from 3-bromo-*N,N*-dimethylaniline (100 mg, 0.500 mmol) and using DCM as solvent, *tert*-butyl ((4-(dimethylamino)-2-bromophenyl)sulfonyl)carbamate **4v** (155 mg, 0.409 mmol, 82%) was obtained as a white solid.

R_f: 0.31 (silica, pentanes:ethyl acetate 3:2);
^{1}**H NMR** (400 MHz, $CDCl_3$): δ = 7.99 (d, *J* = 9.1 Hz, 1H, Ar*H*), 6.89 (d, *J* = 2.6 Hz, 1H, Ar*H*), 6.59 (dd, *J* = 9.1, 2.6 Hz, 1H, Ar*H*), 3.04 (s, 6H, NC*H*3), 1.35 (s, 9H, C*H*3);
^{13}**C NMR** (101 MHz, $CDCl_3$): δ = 153.5, 149.3, 134.2, 122.8, 121.4, 116.5, 109.1, 83.3, 40.0, 27.9;
HRMS (ESI) calcd. for $C_{13}H_{19}N_2O_4BrS$ $[M+H]^+$ 379.0322; Found 379.0328.

***tert*-Butyl ((4-(dimethylamino)-2-iodophenyl)sulfonyl)carbamate** (**4w**)

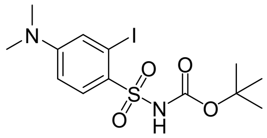

Following GP A, starting from 3-iodo-*N,N*-dimethylaniline (124 mg, 0.500 mmol) and using DCM as solvent, *tert*-butyl ((4-(dimethylamino)-2-iodophenyl)sulfonyl)carbamate **4w** (160 mg, 0.376 mmol, 75%) was obtained as a white solid.

R_f: 0.48 (silica, pentanes:ethyl acetate 3:2);
^{1}**H NMR** (400 MHz, $CDCl_3$): δ = 8.03 (d, J = 9.1 Hz, 1H, Ar*H*), 7.42 (s, 1H, N*H*), 7.24 (d, J = 2.6 Hz, 1H, Ar*H*), 6.65 (dd, J = 9.1, 2.6 Hz, 1H, Ar*H*), 3.04 (s, 6H, NC*H*3), 1.37 (s, 9H, C*H*3);
^{13}**C NMR** (101 MHz, $CDCl_3$): δ = 153.1, 148.8, 133.9, 125.7, 124.0, 109.7, 93.9, 83.6, 40.0, 27.9;
HRMS (ESI) calcd. for $C_{13}H_{19}N_2O_4IS$ $[M+H]^+$ 427.0183; Found 427.0187.

***tert*-Butyl ((2-bromo-4-(dimethylamino)-5-fluorophenyl)sulfonyl)carbamate** (**4x**)

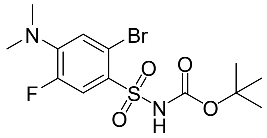

Following GP A, starting from 5-bromo-2-fluoro-*N*,*N*-dimethylaniline (109 mg, 0.500 mmol) and using DCM as solvent, *tert*-butyl ((4-(dimethylamino)-2-iodophenyl)sulfonyl)carbamate **4x** (137 mg, 0.346 mmol, 69%) was obtained as a white solid.

R_f: 0.32 (silica, pentanes:ethyl acetate 3:1);
^{1}**H NMR** (400 MHz, $CDCl_3$): δ = 7.80 (d, *J* = 13.9 Hz, 1H, Ar*H*), 7.53 (s, 1H, N*H*), 6.97 (d, *J* = 8.0 Hz, 1H, Ar*H*), 3.05 (d, *J* = 2.0 Hz, 6H, NC*H*3), 1.38 (s, 9H, C*H*3) (some peaks were split into a doublet due to

rotamers);
^{13}C NMR (101 MHz, $CDCl_3$): δ = 150.0 (d, *J* = 246.9 Hz), 148.7, 144.4 (d, *J* = 8.2 Hz), 125.1 (d, *J* = 6.9 Hz), 121.3, 121.2 (d, *J* = 31.7 Hz), 115.6 (d, *J* = 2.8 Hz), 84.1, 42.0, 41.9, 27.8;
^{19}F NMR (376 MHz, $CDCl_3$): δ = -124.2 – -124.4 (m);
HRMS (ESI) calcd. for $C_{13}H_{19}N_2O_4BrFS$ $[M+H]^+$ 397.0227; Found 397.0228.

***tert*-Butyl ((1-methyl-1*H*-indol-3-yl)sulfonyl)carbamate** (**5a**)

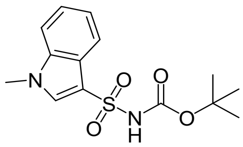

Following GP A, starting from 1-methylindole (65.6 mg, 0.500 mmol) and using DCM as solvent, *tert*-butyl ((1-methyl-1*H*-indol-3-yl)sulfonyl)carbamate **5a** (145 mg, 0.466 mmol, 93%) was obtained as a white solid.

R_f: 0.55 (silica, pentanes:ethyl acetate 1:1);
^{1}H NMR (400 MHz, $CDCl_3$): δ = 8.01 – 7.91 (m, 1H, Ar*H*), 7.87 (s, 1H, Ar*H*), 7.51 (s, 1H, N*H*), 7.44 – 7.28 (m, 3H, Ar*H*), 3.86 (s, 3H, NC*H*3), 1.36 (s, 9H, C*H*3);
^{13}C NMR (101 MHz, $CDCl_3$): δ = 149.3, 136.9, 135.8, 123.9, 123.6, 122.5, 119.8, 111.4, 110.3, 83.3, 33.7, 27.9;
HRMS (ESI) calcd. for $C_{14}H_{18}N_2O_4SNa$ $[M+Na]^+$ 333.0879; Found 333.0878.

***tert*-Butyl ((1-(*tert*-butyldimethylsilyl)-1*H*-indol-3-yl)sulfonyl)carbamate** (**5b**)

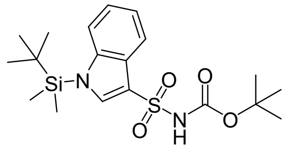

Following GP A, starting from 1-(*tert*-butyldimethylsilyl)-1*H*-indole (133 mg, 0.500 mmol) and using DCM as solvent, *tert*-butyl ((1-(*tert*-butyldimethylsilyl)-1*H*-indol-3-yl)sulfonyl)carbamate **5b** (194 mg, 0.473 mmol, 95%) was obtained as a colorless oil.

R_f: 0.53 (silica, pentanes:ethyl acetate 2:1);
^{1}H NMR (400 MHz, $CDCl_3$): δ = 7.99 – 7.89 (m, 2H, Ar*H*), 7.58 – 7.52 (m, 1H, Ar*H*), 7.41 – 7.26 (m, 3H, Ar*H* + N*H*), 1.35 (s, 9H, C*H*3), 0.94 (s, 9H, C*H*3), 0.66 (s, 6H, SiC*H*3);
^{13}C NMR (101 MHz, $CDCl_3$): δ = 149.1, 141.0, 139.0, 126.0, 123.4, 122.4, 119.7, 115.1, 114.7, 83.3, 27.9, 26.1, 20.2, -4.0;
HRMS (ESI) calcd. for $C_{19}H_{30}N_2O_4SSiNa$ $[M+Na]^+$ 433,1588; Found 433,1588.

***tert*-Butyl 3-(*N*-(*tert*-butoxycarbonyl)sulfamoyl)-1*H*-indole-1-carboxylate** (**5c**)

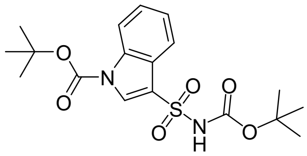

Following GP A, starting from *tert*-butyl 1*H*-indole-1-carboxylate (109 mg, 0.500 mmol) and using DCM as solvent, *tert*-butyl 3-(*N*-(*tert*-butoxycarbonyl)sulfamoyl)-1*H*-indole-1-carboxylate **5c** (189 mg, 0.478

mmol, 96%) was obtained as a colorless oil.

R$_f$: 0.44 (silica, pentanes:ethyl acetate 3:1);
^{1}H NMR (400 MHz, $CDCl_3$): δ = 8.33 (s, 1H, Ar*H*), 8.25 (d, *J* = 8.2 Hz, 1H, Ar*H*), 7.89 (d, *J* = 8.9 Hz, 1H, Ar*H*), 7.73 – 7.55 (br, 1H, N*H*), 7.44 (ddt, *J* = 8.5, 7.2, 1.4 Hz, 1H, Ar*H*), 7.37 (dddd, *J* = 8.3, 7.3, 2.4, 1.2 Hz, 1H, Ar*H*), 1.68 (s, 9H, C*H*3), 1.39 (s, 9H, C*H*3);
^{13}C NMR (101 MHz, $CDCl_3$): δ = 149.0, 148.3, 135.4, 132.7, 126.0, 124.5, 124.4, 119.8, 118.2, 115.6, 86.0, 84.1, 28.0, 27.9;
HRMS (ESI) calcd. for $C_{18}H_{24}N_2O_6SNa$ $[M+Na]^+$ 419,1247; Found 419,1247.

***tert*-Butyl 2-(*N*-(tert-butoxycarbonyl)sulfamoyl)-1*H*-pyrrole-1-carboxylate** (**5d**)

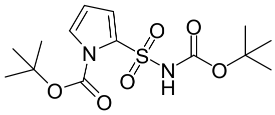

Following GP A, starting from *tert*-butyl 1*H*-pyrrole-1-carboxylate (83.6 mg, 0.500 mmol) and using DCM as solvent, an inseparable mixture of *tert*-Butyl 2-(*N*-(tert-butoxycarbonyl)sulfamoyl)-1*H*-pyrrole-1-carboxylate **5d** containing 20% *tert*-Butyl 2-(*N*-(tert-butoxycarbonyl)sulfamoyl)-1*H*-pyrrole-1-carboxylate (161 mg, 0.465 mmol, 93%) was obtained as a beige oil.

R$_f$: 0.39 (silica, pentanes:ethyl acetate 3:1);
^{1}H NMR (400 MHz, $CDCl_3$): δ = 7.88 (t, J = 2.0 Hz, 1H, Ar*H*, minor), 7.68 (s, 1H, N*H*, major), 7.39 (dd, J = 3.3, 1.9 Hz, 1H, Ar*H*, major), 7.28 (dd, J = 3.5, 2.3 Hz, 1H, Ar*H*, minor), 7.24 (dd, J = 3.7, 1.9 Hz, 1H, Ar*H*, major), 7.19 (s, 1H, N*H*, minor), 6.60 (dd, J = 3.4, 1.7 Hz, 1H, Ar*H*, minor), 6.25 (t, J = 3.5 Hz, 1H, Ar*H*, major), 1.63 (s, 9H, C*H*3, major), 1.61 (s, 3H, C*H*3, minor), 1.45 (s, 3H, C*H*3, minor), 1.41 (s, 9H, C*H*3, major) (mixture of 2 regioisolaers with a ration of 4 :1);
^{13}C NMR (101 MHz, $CDCl_3$): δ = 149.0 (minor), 148.9 (major), 147.4 (minor), 147.0 (major), 128.4 (major), 127.6 (major), 125.9 (major), 125.5 (minor), 124.9 (minor), 121.3 (minor), 110.4 (minor), 110.3 (major), 86.5 (major), 86.1 (minor), 84.0 (minor), 83.7 (major), 27.9 (minor), 27.8 (major), 27.8 (minor), 27.8 (major);
HRMS (ESI) calcd. for $C_{14}H_{22}N_2O_6SNa$ $[M+Na]^+$ 369,1091; Found 369,1089.

***tert*-Butyl ((5-methylfuran-2-yl)sulfonyl)carbamate** (**5e**)

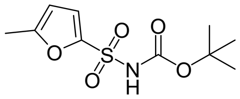

Following GP A, starting from 2-methylfuran (41.1 mg, 0.500 mmol) and using DCM as solvent, *tert*-butyl ((5-methylfuran-2-yl)sulfonyl)carbamate **5e** (78.5 mg, 0.300 mmol, 60%) was obtained as a colorless oil.

R$_f$: 0.38 (silica, pentanes:ethyl acetate 3:1);
^{1}H NMR (400 MHz, $CDCl_3$): δ = 7.32 (s, 1H, N*H*), 7.16 (d, J = 3.5 Hz, 1H, Ar*H*), 6.16 (dd, J = 3.4, 1.0 Hz, 1H, Ar*H*), 2.40 (s, 3H, C*H*3), 1.43 (s, 9H, C*H*3);
^{13}C NMR (101 MHz, $CDCl_3$): δ = 158.2, 148.5, 144.2, 121.0, 108.1, 84.4, 27.8, 13.9;
HRMS (ESI) calcd. for $C_{10}H_{15}NO_5SNa$ $[M+Na]^+$ 284,0563; Found 284,0564.

***tert*-Butyl (*E*)-((2-(dimethylamino)styryl)sulfonyl)carbamate** (**5f**)

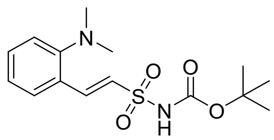

Following GP A, starting from N,N-dimethyl-2-vinylaniline (73.6 mg, 0.500 mmol) and using Et_2O as solvent, *tert*-butyl (*E*)-((2-(dimethylamino)styryl)sulfonyl)carbamate **5f** (75.8 mg, 0.232 mmol, 46%) was obtained as a yellow oil.

R_f: 0.40 (silica, pentanes:ethyl acetate 3:1);
^{1}H NMR (400 MHz, $CDCl_3$): δ = 8.00 (d, J = 15.5 Hz, 1H, vinyl*H*), 7.44 (dd, J = 7.7, 1.6 Hz, 1H, Ar*H*), 7.37 (ddd, J = 8.6, 7.3, 1.7 Hz, 2H, Ar*H* + N*H*), 7.11 – 6.98 (m, 3H, Ar*H* + vinyl*H*), 2.76 (s, 6H, NC*H*3), 1.46 (s, 9H, C*H*3);
^{13}C NMR (101 MHz, $CDCl_3$): δ = 154.3, 149.4, 143.0, 131.8, 129.2, 125. 6, 123.1, 122.2, 118.7, 83.8, 44.9, 27.9;
HRMS (ESI) calcd. for $C_{15}H_{23}N_2O_4S$ $[M+H]^+$ 327,1373; Found 327,1376.

4-(Dimethylamino)benzenesulfonamide (**6a**)

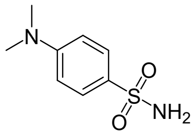

To **4a** (1.27 g, 4.23 mmol) in a 20 mL glass vial was added 6N HCl (5 mL) and the resulting suspension was kept stirring at room temperature for 2 hours. 2M NaOH aqueous solution was then carefully added to the reaction crude to adjust the pH value to 7. The white precipitate was collected by filtration and washed with deionized water (2 x 10 mL). 4-(Dimethylamino)benzenesulfonamide **6a** (0.85 g, 4.25 mmol, >99% yield) was obtained after drying.

R_f: 0.47 (silica, pentanes:ethyl acetate 1:1);
^{1}H NMR (400 MHz, DMSO-*d*$_6$): δ = 7.63 – 7.54 (m, 2H, Ar*H*), 6.96 (s, 2H, NH_2), 6.79 – 6.68 (m, 2H, Ar*H*), 2.97 (s, 6H, C*H*$_3$).

^{1}H NMR data correspond to the reported values.[6]

Bis(acetoxymethyl) 2,2'-(phenylazanediyl)diacetate (**7**)

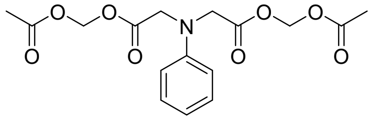

Following a reported procedure,[7] to a solution of 2,2'-(phenylazanediyl)diacetic acid (628 mg, 3.00 mmol, 1 eq.) in 10 mL MeCN was added DIPEA (2.48 mL, 15.0 mmol, 5 eq.) and bromomethyl acetate (882 µL, 9.00 mmol, 3 eq.). The reaction was stirred at rt for 16 hours before the solvent was evaporated. The residue was purified by column chromatography on Biotage (SiO_2 12 g, eluent with 0 - 50% ethyl acetate in n-Hexane, linear gradient). Bis(acetoxymethyl) 2,2'-(phenylazanediyl)diacetate **7** (530 mg, 1.50 mmol, 50%) was obtained as a colorless oil.

6 Nocentini, A.; Vullo, D.; Bartolucci, G.; Supuran, C. T. *Bioorg. Med. Chem.* **2016**, *24*, 3612-3617.
7 Heilporn, S.; Broeders, F.; Daloze, D.; Braekman, J. C. *Bull. Soc. Chim. Belg.* **1994**, *103*, 309-319.

R$_f$: 0.50 (silica, pentanes:ethyl acetate 1:1);
^{1}H NMR (400 MHz, $CDCl_3$): δ = 7.26 – 7.18 (m, 2H, Ar*H*), 6.82 (tt, J = 7.4, 1.0 Hz, 1H, Ar*H*), 6.65 – 6.56 (m, 2H, Ar*H*), 5.80 (s, 4H, OC*H*2O), 4.20 (s, 4H, NC*H*2), 2.10 (s, 6H, C*H*3).

^{1}H NMR data correspond to the reported values.[4

Bis(acetoxymethyl) 2,2'-((4-(*N*-(*tert*-butoxycarbonyl)sulfamoyl)phenyl)azanediyl)diacetate (8)

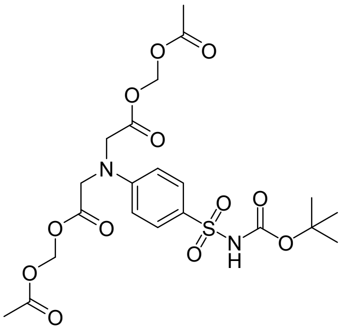

Following GP A, starting from bis(acetoxymethyl) 2,2'-(phenylazanediyl)diacetate **7** (43.1 mg, 0.100 mmol) and using DCM as solvent, bis(acetoxymethyl) 2,2'-((4-(*N*-(*tert*-butoxycarbonyl)sulfamoyl)phenyl)azanediyl)diacetate **8** (45.3 mg, 0.085 mmol, 85%) was obtained as a colorless oil.

R$_f$: 0.27 (silica, pentanes:ethyl acetate 1:1);
^{1}H NMR (400 MHz, $CDCl_3$): δ = 7.95 – 7.75 (m, 2H, Ar*H*), 6.73 – 6.56 (m, 2H, Ar*H*), 5.80 (s, 4H, OC*H*2), 4.24 (s, 4H, NC*H*2), 2.11 (s, 6H, COC*H*3), 1.39 (s, 9H, C*H*3);
^{13}C NMR (101 MHz, $CDCl_3$): δ = 169.5, 168.4, 151.2, 149.2, 130.3, 127.7, 111.6, 83.8, 79.7, 52.9, 27.9, 20.6;
HRMS (ESI) calcd. for $C_{21}H_{32}N_3O_{12}S$ $[M+NH_4]^+$ 550.1701; Found 550.1702.

Bis(acetoxymethyl) 2,2'-((4-sulfamoylphenyl)azanediyl)diacetate (9)

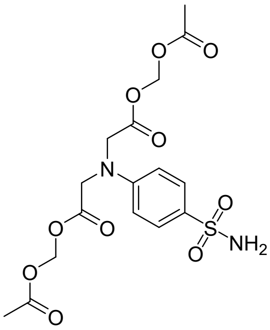

Following GP A, starting from bis(acetoxymethyl) 2,2'-(phenylazanediyl)diacetate **7** (43.1 mg, 0.100 mmol) and using DCM as solvent, after the first step was complete, 0.15 mL TFA was added to the reaction crude and the resulting mixture was kept stirring for 2 hours. It was then quenched by the addition of saturated $NaHCO_3$ (2 mL) and the aqueous layer was then extracted with dichloromethane (3 x 10 mL). The organic phases were combined, dried over Na_2SO_4, filtered and concentrated *in vacuo*. Bis(acetoxymethyl) 2,2'-((4-sulfamoylphenyl)azanediyl)diacetate **9** (34.4 mg, 0.080 mmol, 80%) was obtained as a colorless oil after column chromatography on Biotage (SiO_2 4 g, eluent with 0 - 80% ethyl

acetate in n-Hexane, linear gradient).

R_f: 0.57 (silica, 100% ethyl acetate);
^{1}H NMR (400 MHz, $CDCl_3$): δ = 7.80 – 7.69 (m, 2H, Ar*H*), 6.69 – 6.57 (m, 2H, Ar*H*), 5.78 (s, 4H, OC*H*2), 4.94 (s, 2H, N*H*2), 4.23 (s, 4H, NC*H*2), 2.10 (s, 6H, COC*H*3);
^{13}C NMR (101 MHz, $CDCl_3$): δ = 169.5, 168.6, 150.4, 150.4, 131.2, 128.4, 111.9, 79.7, 52.9, 20.6;
HRMS (ESI) calcd. for $C_{16}H_{21}N_2O_{10}S$ $[M+H]^+$ 433.0911; Found 433.0898.

Bis(acetoxymethyl) 2,2'-((2-(2-(2-(bis(2-(acetoxymethoxy)-2-oxoethyl)amino)-5-sulfamoylphenoxy)ethoxy)phenyl)azanediyl)diacetate (**11**)

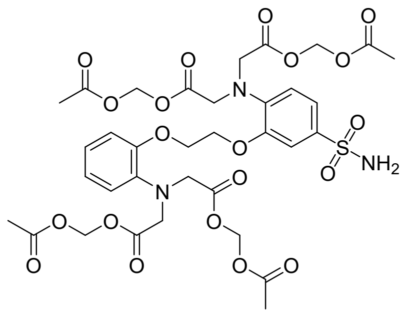

To a vial containing a solution of BAPTA-AM **10** (38.2 mg, 50.0 µmol) and DABCO (8.4 mg, 75.0 µmol, 1.5 equiv.) in dichloromethane (0.25 mL) was added a solution of $ClSO_2NHBoc$ (21.6 mg, 100 µmol, 2.0 equiv.) in dichloromethane (0.25 mL) at room temperature under air. The crude was purified by column chromatography on Biotage (SiO_2 4 g, eluent with 0 - 35% ethyl acetate in n-Hexane, linear gradient) and fractions containing the corresponding Boc-protected product was combined. A colorless oil (25.1 mg) was obtained after evaporation, to which 0.15 mL TFA was added. The resulting mixture was kept stirring for 2 hours, before the volatiles were removed under vacuum. Bis(acetoxymethyl) 2,2'-((2-(2-(2-(bis(2-(acetoxymethoxy)-2-oxoethyl)amino)-5-sulfamoylphenoxy)ethoxy)phenyl)azanediyl)diacetate **11** (22.4 mg, 26.6 µmol, 53%) was obtained as a brown oil, which was used as such without further purification.

R_f: 0.35 (silica, 100% ethyl acetate);
^{1}H NMR (400 MHz, $CDCl_3$): δ = 7.46 – 7.41 (m, 2H, Ar*H*), 7.09 (td, *J* = 7.7, 1.6 Hz, 1H, Ar*H*), 7.03 (dd, *J* = 8.0, 1.6 Hz, 1H, Ar*H*), 6.99 – 6.92 (m, 2H, Ar*H*), 6.81 (d, *J* = 8.9 Hz, 1H, Ar*H*), 5.62 (s, 4H, OC*H*2O), 5.60 (s, 4H, OC*H*2O), 4.41 – 4.36 (m, 2H, OC*H*2), 4.36 – 4.31 (m, 2H, OC*H*2), 4.28 (s, 4H, NC*H*2), 4.21 (s, 4H, NC*H*2), 2.09 (s, 6H, C*H*3), 2.07 (s, 6H, C*H*3);
^{13}C NMR (101 MHz, $CDCl_3$): δ = 170.6, 170.5, 170.0, 169.4, 159.1 (q, *J* = 41.6 Hz), 150.3, 149.5, 142.5, 136.2, 134.4, 125.1, 122.0, 120.6, 120.5, 118.0, 114.7 (q, *J* = 285.8 Hz), 113.3, 111.1, 79.8, 79.7, 67.5, 66.9, 54.1, 53.5, 20.6, 20.5;
HRMS (ESI) calcd. for $C_{34}H_{41}N_3O_{20}S$ $[M+H]^+$ 844.2077; Found 844.2080.

Bis(acetoxymethyl) 2,2'-((2-methoxyphenyl)azanediyl)diacetate (**12**)

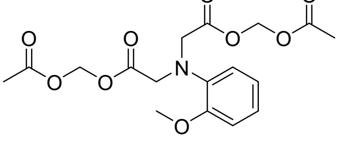

Following a reported procedure,[4] to a solution of 2,2'-((2-methoxyphenyl)azanediyl)diacetic acid (880

mg, 3.68 mmol, 1 eq.) in 10 mL MeCN was added DIPEA (3.04 mL, 18.4 mmol, 5 eq.) and bromomethyl acetate (1.08 mL, 11.0 mmol, 3 eq.). The reaction was stirred at rt for 16 hours before the solvent was evaporated. The residue was purified by column chromatography on Biotage (SiO_2 12 g, eluent with 0 - 55% ethyl acetate in n-Hexane, linear gradient). Bis(acetoxymethyl) 2,2'-((2-methoxyphenyl)azanediyl)diacetate **12** (740 mg, 1.93 mmol, 52%) was obtained as a colorless oil.

R$_f$: 0.28 (silica, pentanes:ethyl acetate 3:2);
^{1}H NMR (400 MHz, $CDCl_3$): δ = 6.98 – 6.92 (m, 1H, Ar*H*), 6.90 – 6.80 (m, 3H, Ar*H*), 5.78 (s, 4H, OC*H*2O), 4.18 (s, 4H, NC*H*2), 3.81 (s, 3H, OC*H*3), 2.10 (s, 6H, C*H*3);
^{13}C NMR (101 MHz, $CDCl_3$): δ = 170.2, 169.5, 151.5, 138.1, 123.0, 121.0, 119.4, 112.1, 79.2, 55.5, 53.7, 20.7;
HRMS (ESI) calcd. for $C_{17}H_{22}NO_9$ [M+H]+ 384.1289; Found 384.1290.

Bis(acetoxymethyl) 2,2'-((2-methoxy-4-sulfamoylphenyl)azanediyl)diacetate (**13**)

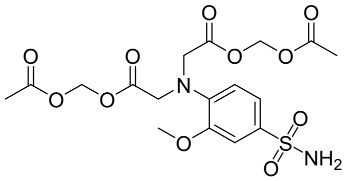

To a vial containing a solution of **12** (38.3 mg, 100 µmol) and DABCO (16.8 mg, 150 µmol, 1.5 equiv.) in dichloromethane (0.5 mL) was added a solution of $ClSO_2NHBoc$ (43.2 mg, 200 µmol, 2.0 equiv.) in dichloromethane (0.5 mL) at room temperature under air. The crude was purified by column chromatography on Biotage (SiO_2 4 g, eluent with 0 - 35% ethyl acetate in n-Hexane, linear gradient) and fractions containing the corresponding Boc-protected product was combined. A colorless oil (23.0 mg) was obtained after evaporation, to which 0.15 mL TFA was added. The resulting mixture was kept stirring for 2 hours, before the volatiles were removed under vacuum. Bis(acetoxymethyl) 2,2'-((2-methoxy-4-sulfamoylphenyl)azanediyl)diacetate **13** (18.9 mg, 40.9 µmol, 41%) was obtained as a colorless oil, which was used as such without further purification.

R$_f$: 0.50 (silica, 100% ethyl acetate);
^{1}H NMR (400 MHz, $CDCl_3$): δ = 7.40 (dt, J = 8.4, 1.8 Hz, 1H, Ar*H*), 7.34 (t, J = 2.0 Hz, 1H, Ar*H*), 6.75 (dd, J = 8.4, 5.8 Hz, 1H, Ar*H*), 5.79 (s, 4H, OC*H*2O), 4.18 (s, 4H, OC*H*2), 3.81 (s, 4H, NC*H*2), 2.12 (s, 6H, C*H*3);
^{13}C NMR (101 MHz, $CDCl_3$): δ = 169.8, 169.7, 150.4, 142.2, 134.2, 120.7, 120.0, 117.4, 110.0, 79.6, 56.0, 53.8, 20.6;
HRMS (ESI) calcd. for $C_{17}H_{23}N_2O_{11}S$ $[M+H]^+$ 463.1017; Found 463.1014.

Bis(acetoxymethyl) 2,2'-((2-(2-(acetoxymethoxy)-2-oxoethoxy)phenyl)azanediyl)diacetate (**14**)

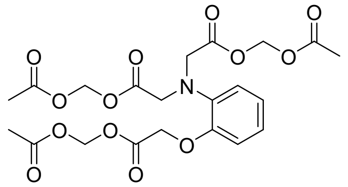

Following a reported procedure,[4] to a solution of 2,2'-((2-(carboxymethoxy)phenyl)azanediyl)diacetic acid (567 mg, 2.00 mmol, 1 eq.) in 10 mL MeCN was added DIPEA (2.00 mL, 12.1 mmol, 6 eq.) and bromomethyl acetate (883 µL, 9.010 mmol, 4.5 eq.). The reaction was stirred at rt for 16 hours before

the solvent was evaporated. The residue was purified by column chromatography on Biotage (SiO_2 12 g, eluent with 0 - 60% ethyl acetate in n-Hexane, linear gradient). Bis(acetoxymethyl) 2,2'-((2-(2-(acetoxymethoxy)-2-oxoethoxy)phenyl)azanediyl)diacetate **14** (450 mg, 901 µmol, 45%) was obtained as a colorless oil.

R_f: 0.32 (silica, pentanes:ethyl acetate 1:1);
^{1}H NMR (400 MHz, $CDCl_3$): δ = 7.00 – 6.85 (m, 3H, Ar*H*), 6.82 (ddt, J = 7.6, 5.9, 2.4 Hz, 1H, Ar*H*), 5.84 – 5.73 (m, 4H, OC*H*2O), 5.31 (dd, J = 5.2, 3.7 Hz, 2H, OC*H*2O), 4.70 (s, 2H, OC*H*2), 4.23 (d, J = 2.1 Hz, 4H, NC*H*2), 2.13 – 2.04 (m, 9H, C*H*3);
^{13}C NMR (101 MHz, $CDCl_3$, signals from the rotamers was omitted): δ = 170.0, 169.5, 167.7, 149.6, 139.1, 123.0, 122.9, 120.2, 115.0, 79.3, 79.2, 65.8, 53.5, 20.9, 20.7;
HRMS (ESI) calcd. for $C_{21}H_{26}NO_{13}$ $[M+H]^+$ 500.1399; Found 500.1403.

Bis(acetoxymethyl) 2,2'-((2-(2-(acetoxymethoxy)-2-oxoethoxy)-4-sulfamoylphenyl)azanediyl)diacetate (**15**)

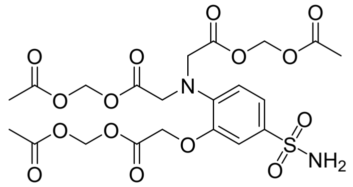

To a vial containing a solution of **14** (49.9 mg, 100 µmol) and DABCO (16.8 mg, 150 µmol, 1.5 equiv.) in dichloromethane (0.5 mL) was added a solution of $ClSO_2NHBoc$ (43.2 mg, 200 µmol, 2.0 equiv.) in dichloromethane (0.5 mL) at room temperature under air. The crude was purified by column chromatography on Biotage (SiO_2 4 g, eluent with 0 - 35% ethyl acetate in n-Hexane, linear gradient) and fractions containing the corresponding Boc-protected product was combined. A colorless oil (17.8 mg) was obtained after evaporation, to which 0.15 mL TFA was added. The resulting mixture was kept stirring for 2 hours, before the volatiles were removed under vacuum. Bis(acetoxymethyl) 2,2'-((2-(2-(acetoxymethoxy)-2-oxoethoxy)-4-sulfamoylphenyl)azanediyl)diacetate **15** (14.7 mg, 30.8 µmol, 31%) was obtained as a colorless oil, which was used as such without further purification.

R_f: 0.45 (silica, 100% ethyl acetate);
^{1}H NMR (400 MHz, $CDCl_3$, mixture of two rotamers: the signals corresponding to the two rotamers are partially resolved): δ = 7.46 (dt, J = 8.5, 1.7 Hz, 1H, Ar*H*), 7.30 – 7.18 (m, 1H, Ar*H*), 6.82 (dd, J = 8.5, 4.4 Hz, 1H, Ar*H*), 5.83 – 5.74 (m, 4H, OC*H*2O), 5.46 – 5.42 (m, 1H, OC*H*2O), 5.32 (d, J = 5.9 Hz, 1H, OC*H*2O), 4.70 (s, 2H, OC*H*2), 4.25 (s, 4H, NC*H*2), 2.20 – 2.01 (m, 9H, COC*H*3);
^{13}C NMR (101 MHz, $CDCl_3$, mixture of two rotamers): δ = 170.7, 170.5, 170.2, 170.0, 169.5, 169.5, 167.5, 166.7, 148.3, 148.3, 148.2, 148.2, 142.6, 142.5, 134.9, 134.8, 134.8, 134.8, 121.2, 121.1, 118.2, 118.2, 111.5, 111.4, 87.5, 87.3, 86.8, 86.6, 79.6, 79.5, 65.3, 65.1, 53.7, 53.6, 20.9, 20.9, 20.6, 20.6;
HRMS (ESI) calcd. for $C_{21}H_{26}N_2O_{15}SNa$ $[M+Na]^+$ 601.0946; Found 601.0939.

16-(2-(2-Methoxyethoxy)phenyl)-1,4,7,10,13-pentaoxa-16-azacyclooctadecane (**16**)

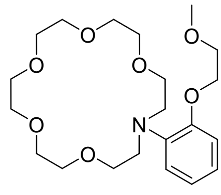

Following a reported procedure,[8] a solution of ((oxybis(ethane-2,1-diyl))bis(oxy))bis(ethane-2,1-diyl) bis(4-methylbenzenesulfonate) (8.86 g, 17.6 mmol, 1 eq.) and 2,2'-((2-(2-methoxyethoxy)phenyl)azanediyl)bis(ethan-1-ol) (4.5 g, 17.6 mmol, 1 eq.) in 50 mL anhydrous THF was added to s suspension of NaH (1.41 g, 35.2 mmol, 2 eq.) in 50 mL anhydrous THF at 60 °C. The reaction was stirred at this temperature for 16 hours before passing through a pad of celite and washed with THF (3 x 20 mL). The solvent was evaporated and the residue was purified by recrystallization in ethyl acetate. 16-(2-(2-Methoxyethoxy)phenyl)-1,4,7,10,13-pentaoxa-16-azacyclooctadecane **16** (6.50 g, 15.7 mmol, 89%) was obtained as a brown oil.

^{1}H NMR (400 MHz, $CDCl_3$): δ = 7.21 – 6.77 (m, 4H, Ar*H*), 4.14 (dt, J = 44.5, 4.8 Hz, 2H, C*H*2), 3.81 – 3.35 (m, 27H, C*H*2 + C*H*3), 3.24 – 3.08 (m, 2H, C*H*2).

^{1}H NMR data correspond to the reported values.[5]

4-(1,4,7,10,13-Pentaoxa-16-azacyclooctadecan-16-yl)-3-(2-methoxyethoxy)benzenesulfonamide (**2**)

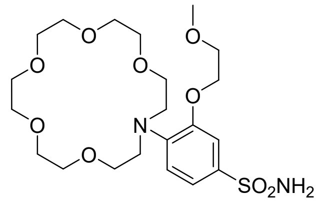

To a vial containing a solution of **16** (41.4 mg, 100 µmol) and DABCO (16.8 mg, 150 µmol, 1.5 equiv.) in dichloromethane (0.5 mL) was added a solution of $ClSO_2NHBoc$ (43.2 mg, 200 µmol, 2.0 equiv.) in dichloromethane (0.5 mL) at room temperature under air. After the first step was complete, 0.15 mL TFA was added to the reaction crude and the mixture was kept stirring for 2 hours. The volatiles were removed under vacuum and the crude was dissolved in DMSO, filtered, purified by Thermo preparative HPLC (8 mL/min; 10 - 90 % MeCN/H_2O (0.1 % TFA) in 60 min). 4-(1,4,7,10,13-Pentaoxa-16-azacyclooctadecan-16-yl)-3-(2-methoxyethoxy)benzenesulfonamide **2** (22.8 mg, 46.2 µmol, 46%) was obtained as a beige oil after lyophilization.

^{1}H NMR (400 MHz, $CDCl_3$): δ = 7.88 (d, J = 1.8 Hz, 1H, ArH), 7.80 (d, J = 8.3 Hz, 1H, ArH), 7.68 (dd, J = 8.4, 1.7 Hz, 1H, ArH), 4.42 (t, J = 4.1 Hz, 2H, OC*H*2), 3.87 (s, 4H, OC*H*2), 3.78 – 3.57 (m, 15H, OC*H*2), 3.47 – 3.38 (m, 7H, OC*H*2 + NC*H*2), 3.36 (s, 3H, OC*H*3);
^{13}C NMR (101 MHz, $CDCl_3$): δ = 152.4, 127.3, 119.8, 117.3, 114.4, 113.7, 70.3, 70.2, 70.1, 69.9, 69.0, 68.9, 64.2, 58.6, 56.3;
HRMS (ESI) calcd. for $C_{21}H_{37}N_2O_9S$ $[M+H]^+$ 493.2214; Found 493.2219.

2-((4-(dimethylamino)phenyl)sulfonyl)-3-oxo-3',6'-bis((2,2,2-trifluoroethyl)amino)spiro[isoindoline-1,9'-xanthene]-6-carboxylic acid (**18**)

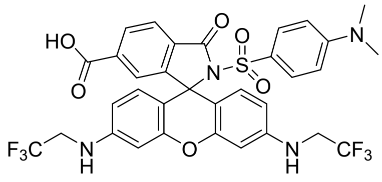

[8] Ast, S.; Schwarze, T.; Müller, H.; Sukhanov, A.; Michaelis, S.; Wegener, J.; Wolfbeis, O. S.; Körzdörfer, T.; Dürkop, A.; Holdt, H.-J. *Chem. Eur. J.* **2013**, *19*, 14911-14917.

A solution of (*E*)-4-*tert*-butoxycarbonyl)-2-(6-((2,2,2-trifluoroethyl)amino)-3-((2,2,2-trifluoro-ethyl)iminio)-3*H*-xanthen-9-yl)benzoate **17** (4.00 mg, 6.73 µmol, 1.0 eq.), **6a** (6.74 mg, 33.6 µmol, 5.0 eq.), 1-(3-dimethylaminopropyl)-3-ethylcarbodiimide hydrochloride (5.16 mg, 26.9 µmol, 4 eq.) and 4-dimethylaminopyridine (3.29 mg, 26.9 µmol, 4 eq.) in DCM (0.6 mL) was heated to 60°C and stirred at this temperature for 12 h in a sealed tube. H_2O (1 mL) was added and the aqueous layer was extracted with DCM (3x). The combined organic layers were dried over Na_2SO_4, filtered and concentrated. The residue was dissolved in TFA/DCM (1:4, 0.6 mL) and stirred at rt for 2 h. After the solvent was evaporated, the crude product was dissolved in DMSO (0.8 mL) and purified by preparative HPLC (8 mL/min, 30 - 90 % MeCN/H_2O (0.1 % TFA) in 60 min) to give **18** (1.4 mg, 1.94 µmol, 29 %) as a red solid.

^{1}H NMR (400 MHz, DMSO-*d*$_6$): δ = 8.08 (d, J = 8.0 Hz, 1H, Ar*H*), 7.92 (d, J = 8.0 Hz, 1H, Ar*H*), 7.41 (s, 1H, Ar*H*), 7.07 (d, J = 8.9 Hz, 2H, Ar*H*), 6.65 (s, 2H, N*H*), 6.62 (d, J = 2.4 Hz, 2H, Ar*H*), 6.51 (d, J = 9.0 Hz, 2H, Ar*H*), 6.36 (dd, J = 8.7, 2.4 Hz, 2H, Ar*H*), 6.24 (d, J = 8.6 Hz, 2H, Ar*H*), 4.06 – 3.94 (m, 4H, NC*H2*), 2.97 (s, 6H, NC*H3*);
^{13}C NMR (101 MHz, DMSO-*d*$_6$, the signal of methyl carbon overlaps with that of DMSO): δ = 166.0, 164.4, 153.2, 152.8, 152.4, 149.2, 136.8, 131.2, 130.0, 129.8, 128.5, 125.6 (q, J = 326.6 Hz), 124.7, 124.4, 123.2, 109.7, 109.4, 107.3, 98.3, 68.0, 43.8 (q, J = 33.2 Hz);
HRMS (ESI) calcd. for $C_{33}H_{27}F_6N_4O_6S$ $[M+H]^+$ 721.1550; Found 721.1555.

N-(2-(2-((6-Chlorohexyl)oxy)ethoxy)ethyl)-2-((4-(dimethylamino)phenyl)sulfonyl)-3-oxo-3',6'-bis((2,2,2-trifluoroethyl)amino)spiro[isoindoline-1,9'-xanthene]-6-carboxamide (20)

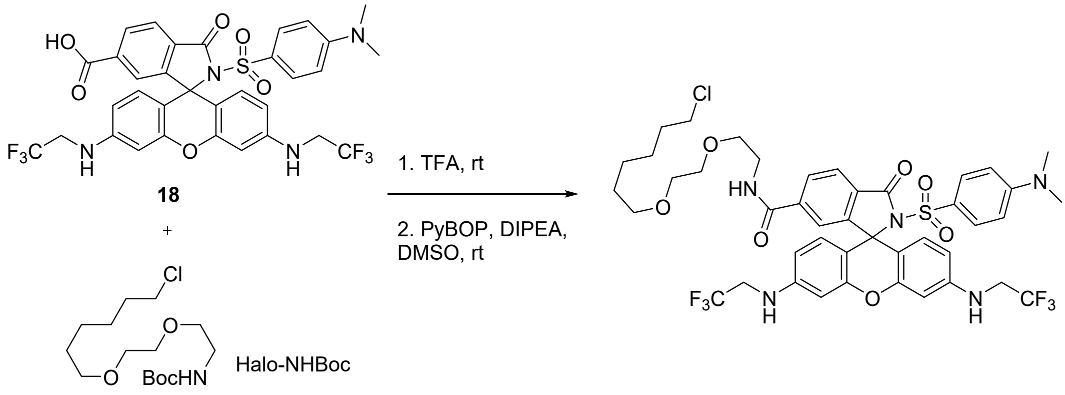

A solution of Halo-NHBoc (0.58 mg, 2.16 µmol, 1.3 eq) in TFA (0.3 mL) was stirred at rt for 5 min. The solvent was evaporated, the residue dissolved in DMSO (0.2 mL) and transferred to a solution of **18** (1.20 mg, 1.67 µmol, 1.0 eq), PyBOP (1.3 mg, 2.50 µmol, 1.5 eq), and DIPEA (2.76 µL, 16.7 µmol, 10 eq) in DMSO (0.2 mL). The reaction mixture was stirred at rt for 40 min followed by purification using preparative HPLC (8 mL/min, 30 - 90 % MeCN/H_2O (0.1 % TFA) in 60 min) to give **20** (0.90 mg, 0.97 µmol, 58 %) as a red solid.

^{1}H NMR (400 MHz, CD_3OD): δ = 8.02 (d, J = 8.1 Hz, 1H, Ar*H*), 7.94 (d, J = 8.1 Hz, 1H, Ar*H*), 7.50 (s, 1H, Ar*H*), 7.20 (d, J = 8.8 Hz, 2H, Ar*H*), 6.59 (s, 2H, Ar*H*), 6.52 (d, J = 9.2 Hz, 2H, Ar*H*), 6.34 – 6.23 (m, 4H, Ar*H*), 3.89 (q, J = 9.3 Hz, 4H, NC*H2*CF3), 3.57 – 3.51 (m, 5H, OC*H2* + N*H*), 3.48 (dd, J = 10.0, 6.0 Hz, 6H, OC*H2*), 3.37 (t, J = 6.5 Hz, 4H, NC*H2* + ClC*H2*), 3.01 (s, 6H, NC*H3*), 1.69 (p, J = 6.8 Hz, 2H, C*H2*), 1.47 (p, J = 6.7 Hz, 2H, C*H2*), 1.42 – 1.36 (m, 2H, C*H2*), 0.90 (t, J = 6.8 Hz, 2H, C*H2*);
HRMS (ESI) calcd. for $C_{43}H_{46}N_5O_7ClF_6S$ $[M+H]^+$ 926.2783; Found 926.2778.

Benzyl ((4-(dimethylamino)phenyl)sulfonyl)carbamate (**4a-Cbz**)

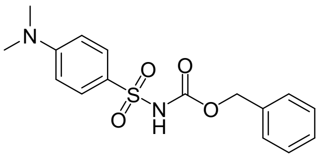

Following GP A, starting from *N*,*N*-dimethylaniline **3a** (60.6 mg, 0.500 mmol), benzyl (chlorosulfonyl)carbamate (250 mg, 1.00 mmol, 2.0 eq) and using Et_2O as solvent, benzyl ((4-(dimethylamino)phenyl)sulfonyl)carbamate **4a-Cbz** (121 mg, 0.362 mmol, 72%) was obtained as a white solid.

$\mathbf{R_f}$: 0.44 (silica, pentanes:ethyl acetate 1:1);
^{1}**H NMR** (400 MHz, $CDCl_3$): δ = 7.88 – 7.65 (m, 2H, Ar*H*), 7.29 – 7.22 (m, 3H, Ar*H*), 7.22 – 7.18 (m, 2H, Ar*H*), 6.66 – 6.49 (m, 2H, Ar*H*), 5.03 (s, 2H, OC*H*2), 3.00 (s, 6H, NC*H*3);
^{13}**C NMR** (101 MHz, $CDCl_3$): δ = 153.6, 150.6, 134.7, 130.3, 128.5, 128.5, 128.3, 122.9, 110.5, 68.2, 40.0 ();
HRMS (ESI) calcd. for $C_{16}H_{19}N_2O_4S$ $[M+H]^+$ 335.1060; Found 335.1061.

Allyl ((4-(dimethylamino)phenyl)sulfonyl)carbamate (**4a-Alloc**)

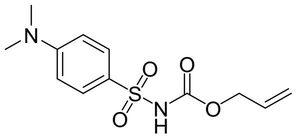

Following GP A, starting from *N*,*N*-dimethylaniline **3a** (60.6 mg, 0.500 mmol), allyl (chlorosulfonyl)carbamate (200 mg, 1.00 mmol, 2.0 eq) and using Et_2O as solvent, allyl ((4-(dimethylamino)phenyl)sulfonyl)carbamate **4a-Alloc** (92.4 mg, 0.325 mmol, 65%) was obtained as a white solid.

$\mathbf{R_f}$: 0.44 (silica, pentanes:ethyl acetate 1:1);
^{1}**H NMR** (400 MHz, $CDCl_3$): δ = 7.92 – 7.73 (m, 2H, Ar*H*), 6.73 – 6.57 (m, 2H, Ar*H*), 5.83 (ddt, J = 16.5, 11.0, 5.8 Hz, 1H, vinyl*H*), 5.34 – 5.14 (m, 2H, vinyl), 4.64 – 4.47 (m, 2H, OC*H*2), 3.06 (s, 6H, NC*H*3);
^{13}**C NMR** (101 MHz, $CDCl_3$): δ = 153.6, 150.4, 131.1, 130.3, 122.9, 119.1, 110.5, 67.0, 40.0;
HRMS (ESI) calcd. for $C_{12}H_{17}N_2O_4S$ $[M+H]^+$ 285.0904; Found 285.0905.

2,2,2-Trichloroethyl ((4-(dimethylamino)phenyl)sulfonyl)carbamate (**4a-Troc**)

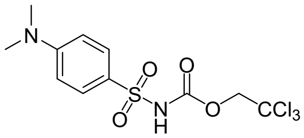

Following GP A, starting from *N*,*N*-dimethylaniline **3a** (60.6 mg, 0.500 mmol), 2,2,2-trichloroethyl (chlorosulfonyl)carbamate (291 mg, 1.00 mmol, 2.0 eq) and using Et_2O as solvent, 2,2,2-trichloroethyl ((4-(dimethylamino)phenyl)sulfonyl)carbamate **4a-Troc** (119 mg, 0.317 mmol, 64%) was obtained as a white solid.

$\mathbf{R_f}$: 0.50 (silica, pentanes:ethyl acetate 1:1);
^{1}**H NMR** (400 MHz, $CDCl_3$): δ = 7.89 – 7.81 (m, 2H, Ar*H*), 6.70 – 6.61 (m, 2H, Ar*H*), 4.67 (s, 2H, OC*H*2), 3.06 (s, 6H, NC*H*3);
^{13}**C NMR** (101 MHz, $CDCl_3$): δ = 153.7, 149.3, 130.4, 122.2, 110.5, 94.2, 75.0, 40.0;

HRMS (ESI) calcd. for $C_{11}H_{13}Cl_3N_2O_4S$ $[M+H]^+$ 374.9734; Found 374.9742.

***N*-((4-(Dimethylamino)phenyl)sulfonyl)benzamide** (**4a-Bz**)

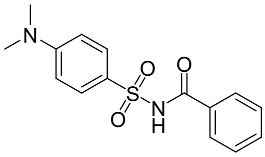

Following GP A, starting from *N*,*N*-dimethylaniline **3a** (12.1 mg, 0.100 mmol), benzoylsulfamoyl chloride (44.0 mg, 0.200 mmol, 2.0 eq) and using Et_2O as solvent, *N*-((4-(dimethylamino)phenyl)sulfonyl)benzamide **4a-Bz** (2.4 mg, 0.078 mmol, 8%) was obtained as a white solid.

R_f: 0.40 (silica, pentanes:ethyl acetate 1:1);
^{1}H NMR (400 MHz, $CDCl_3$): δ = 8.66 (s, 1H, N*H*), 8.06 – 7.89 (m, 2H, Ar*H*), 7.84 – 7.68 (m, 2H, Ar*H*), 7.60 – 7.51 (m, 1H, Ar*H*), 7.44 (t, J = 7.7 Hz, 2H, Ar*H*), 6.78 – 6.60 (m, 2H, Ar*H*), 3.06 (s, 6H, NC*H*3);
^{13}C NMR (101 MHz, $CDCl_3$): δ = 164.0, 153.7, 133.2, 131.7, 130.7, 128.9, 127.6, 122.8, 110.5, 40.0;
HRMS (ESI) calcd. for $C_{15}H_{17}N_2O_3S$ $[M+H]^+$ 305.0954; Found 305.0959.

Scheme S4. Comparison of fluorogenicity between lacton **19** and lactam **20**

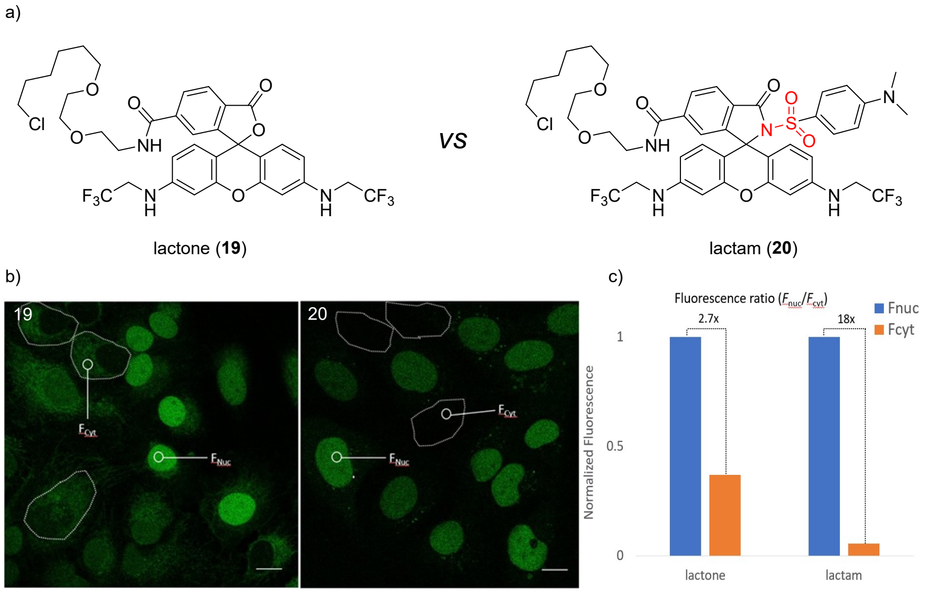

5. NMR spectra of new compounds

tert-Butyl (_tert_-butoxycarbonyl)(chlorosulfonyl)carbamate

^{1}H-NMR (400 MHz, $CDCl_3$)

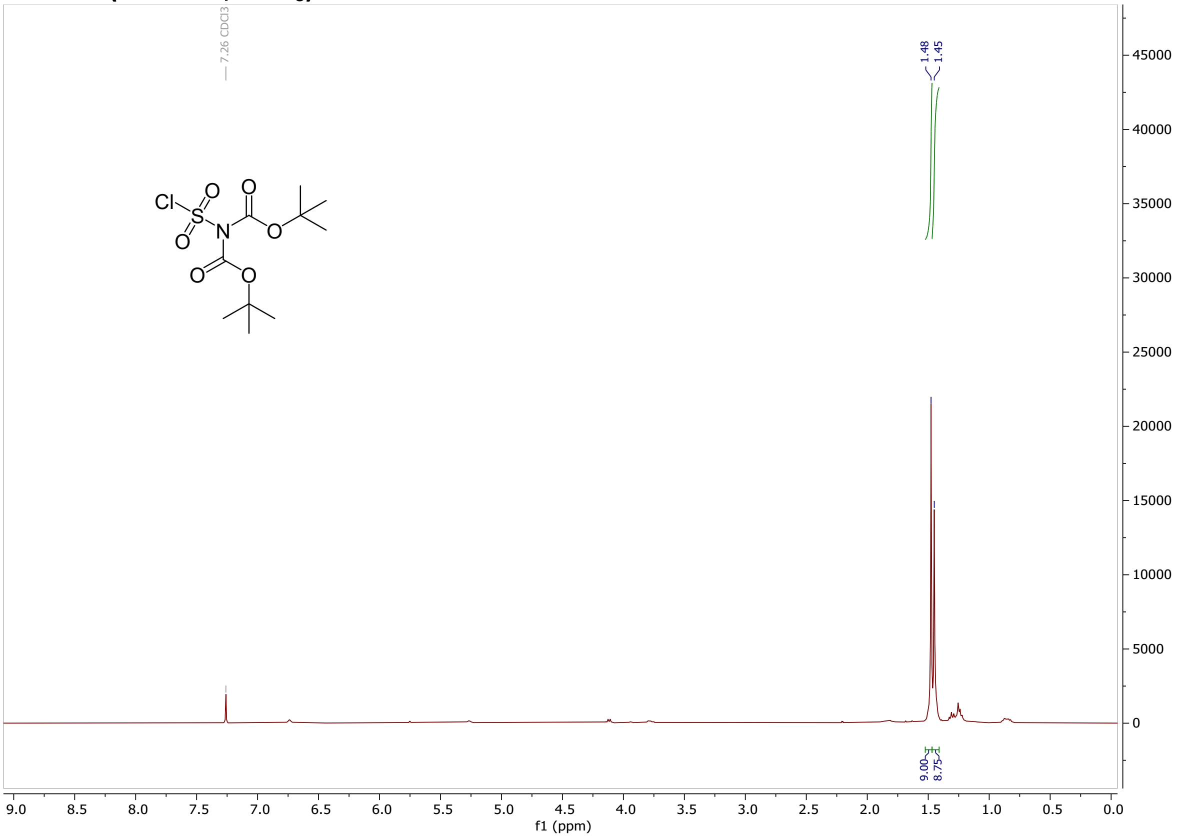

^{13}C-NMR (101 MHz, $CDCl_3$)

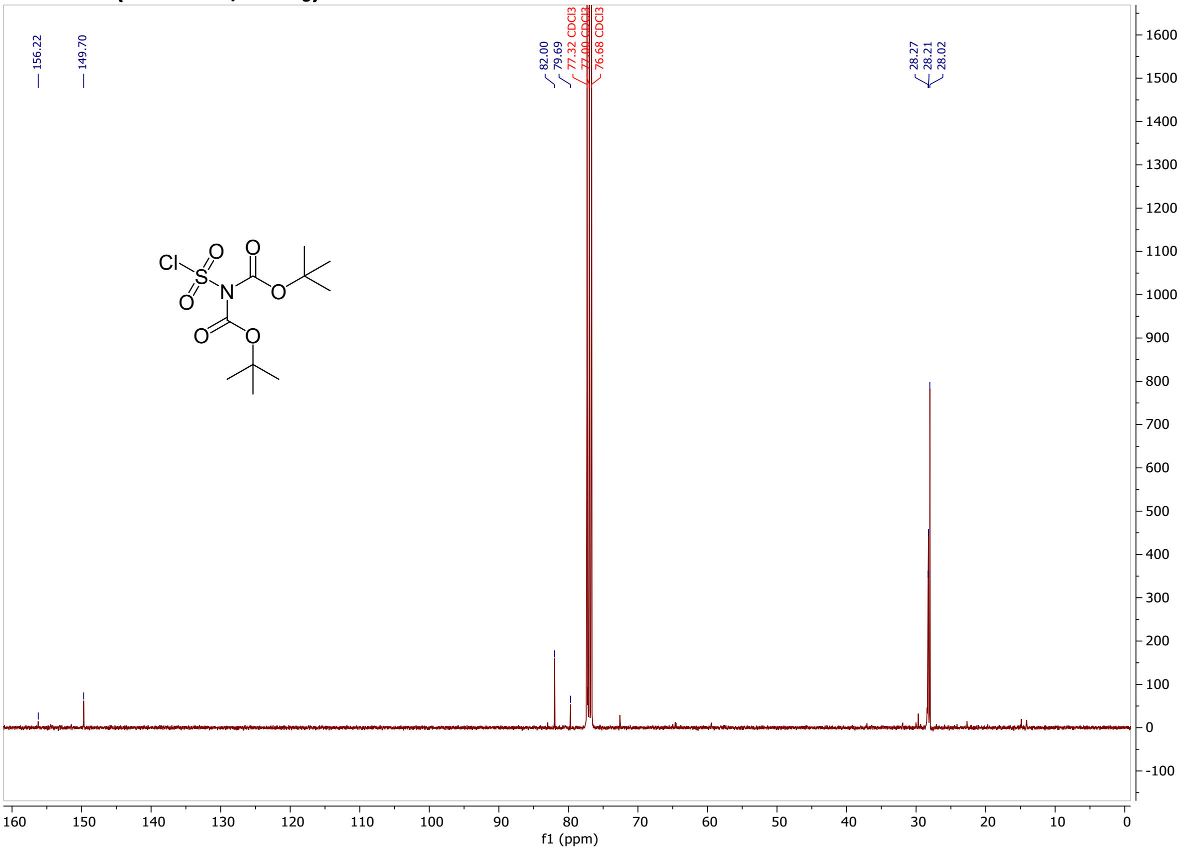

***tert*-Butyl ((4-(dimethylamino)phenyl)sulfonyl)carbamate (4a)**

^{1}H-NMR (400 MHz, $CDCl_3$)

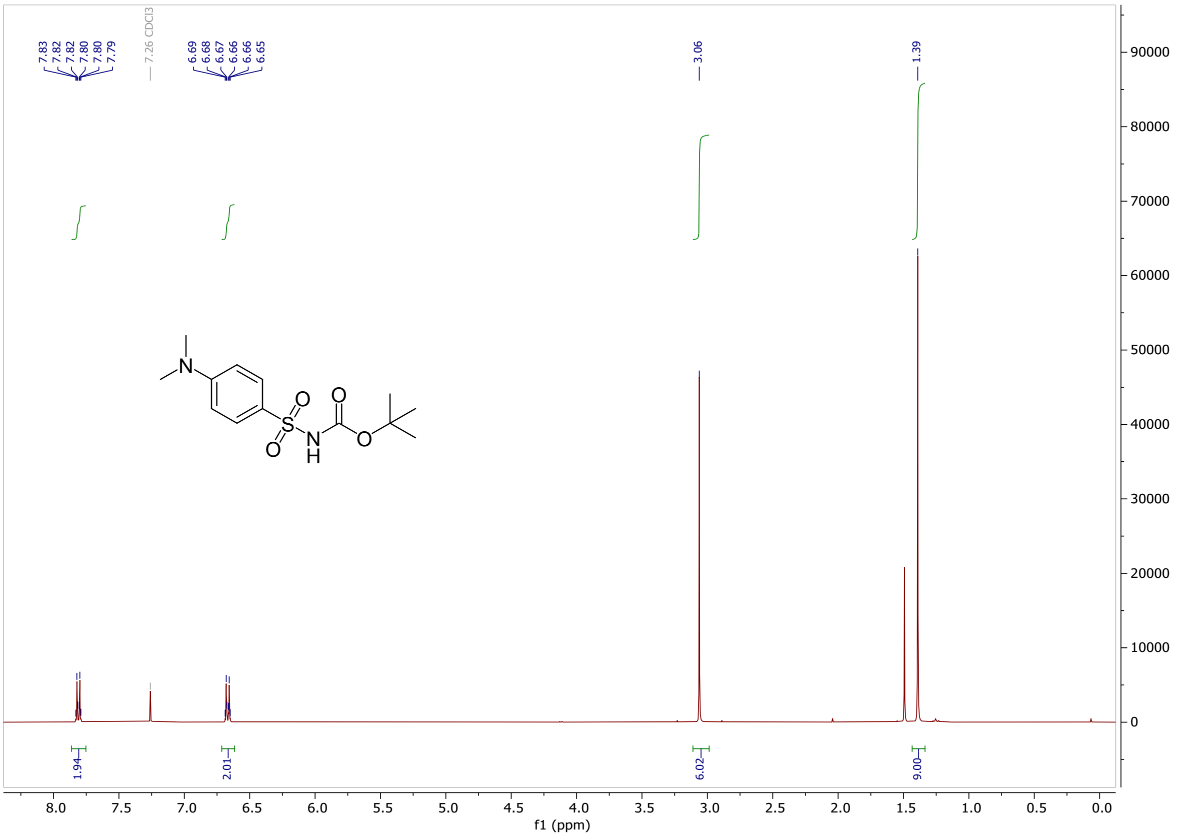

^{13}C-NMR (101 MHz, $CDCl_3$)

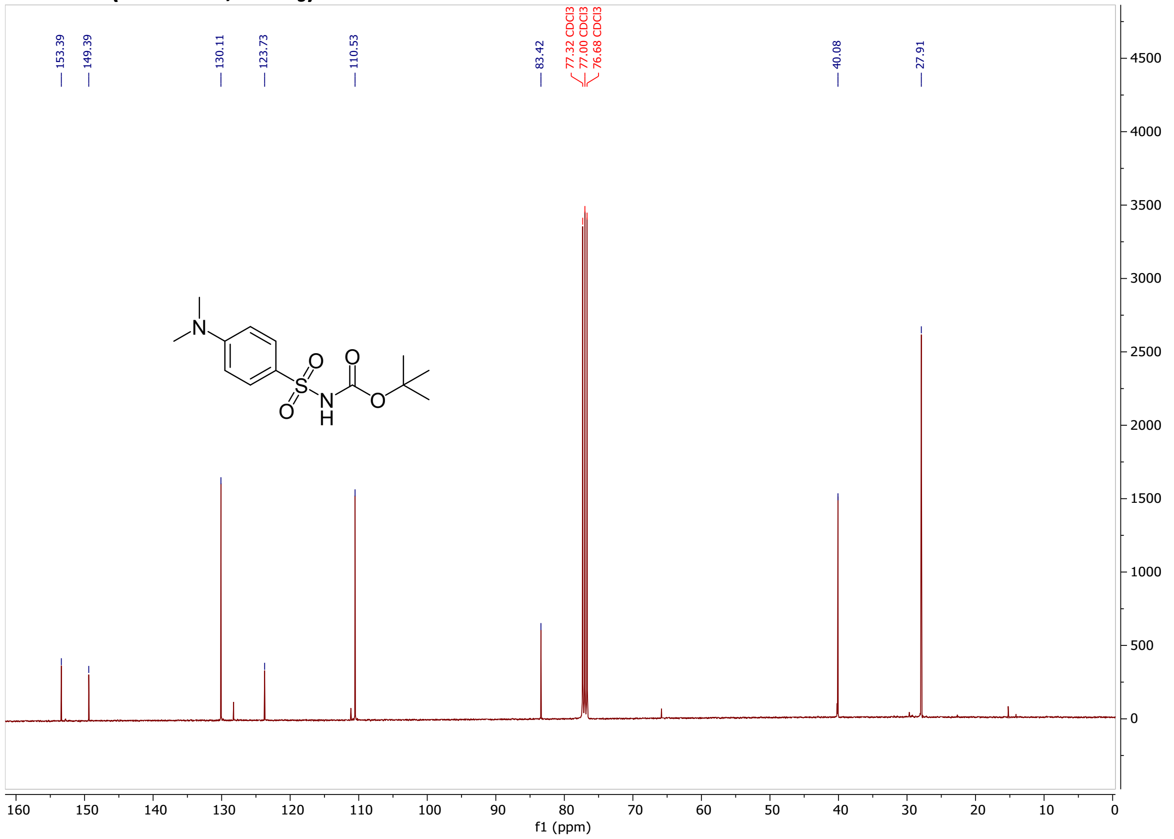

***tert*-Butyl ((2-(dimethylamino)phenyl)sulfonyl)carbamate (4a')**

^{1}H-NMR (400 MHz, $CDCl_3$)

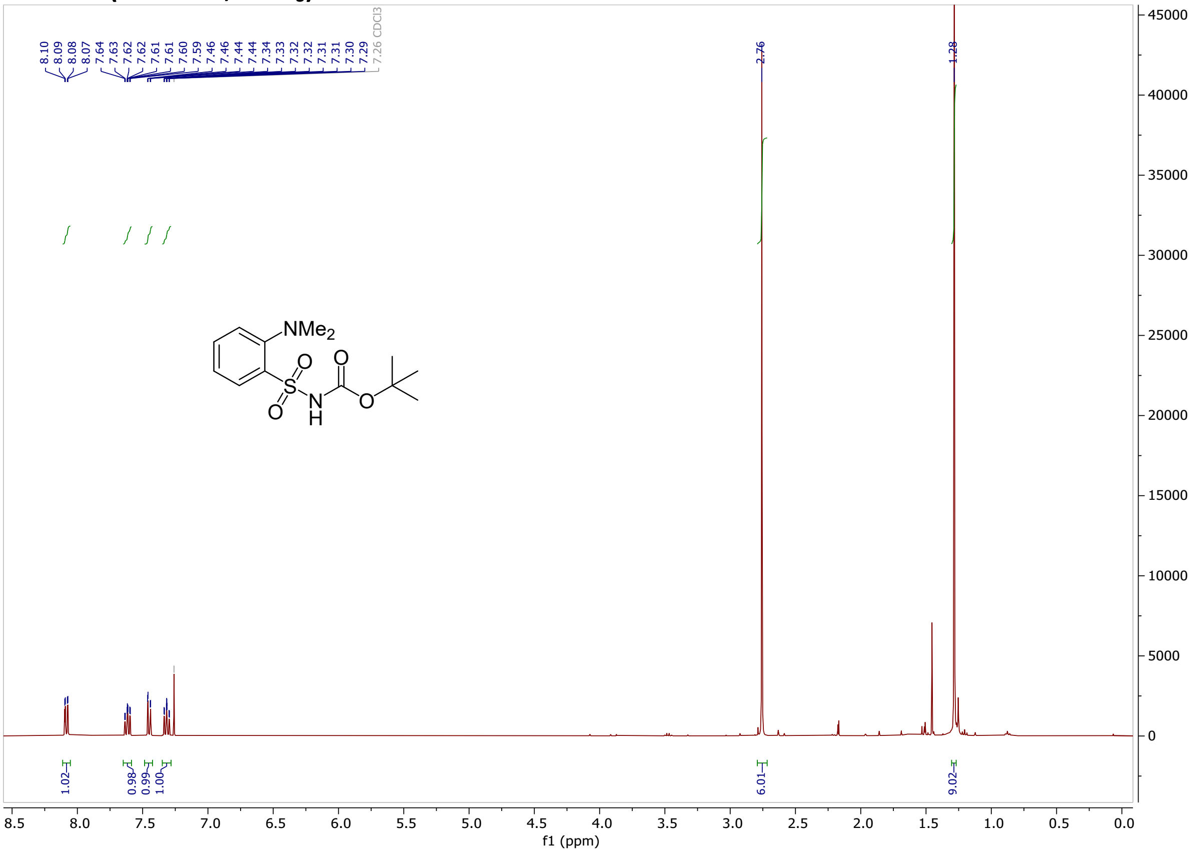

^{13}C-NMR (101 MHz, $CDCl_3$)

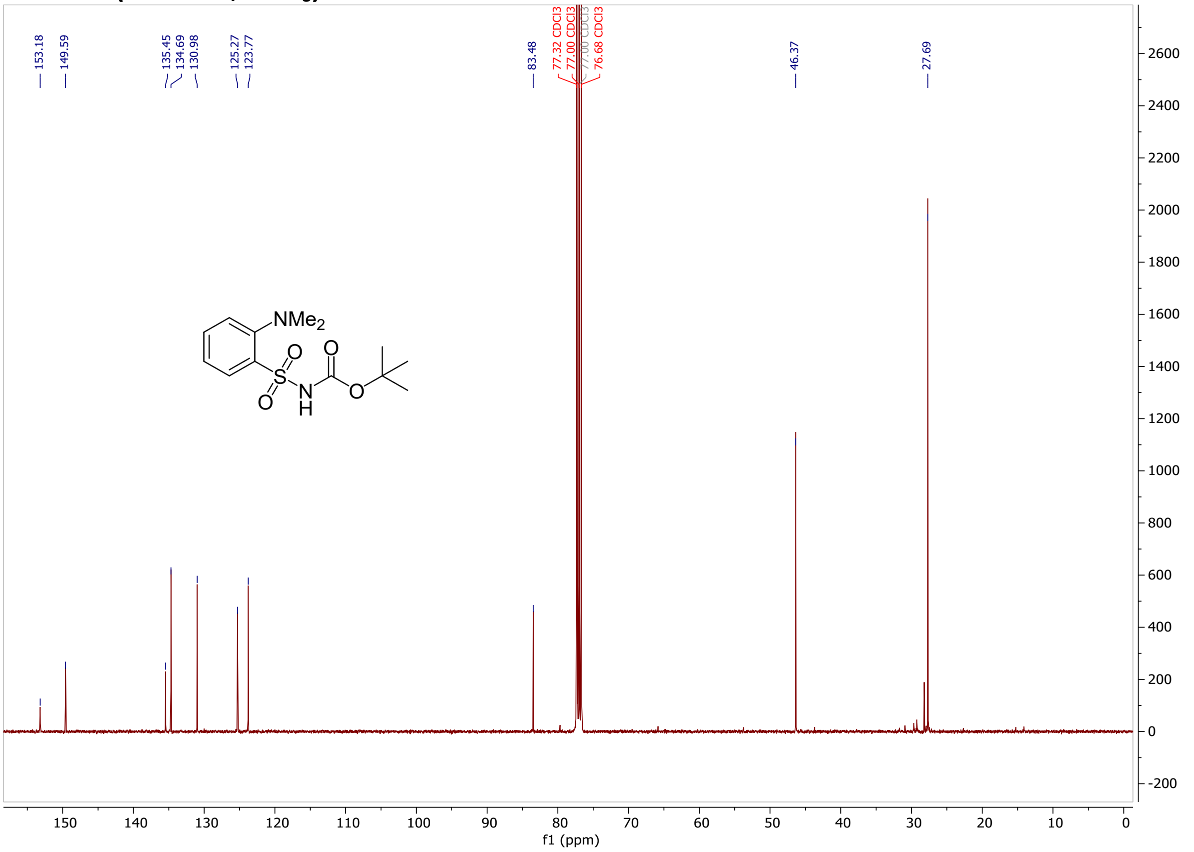

***tert*-Butyl ((4-(diethylamino)phenyl)sulfonyl)carbamate (4b)**

^{1}H-NMR (400 MHz, $CDCl_3$)

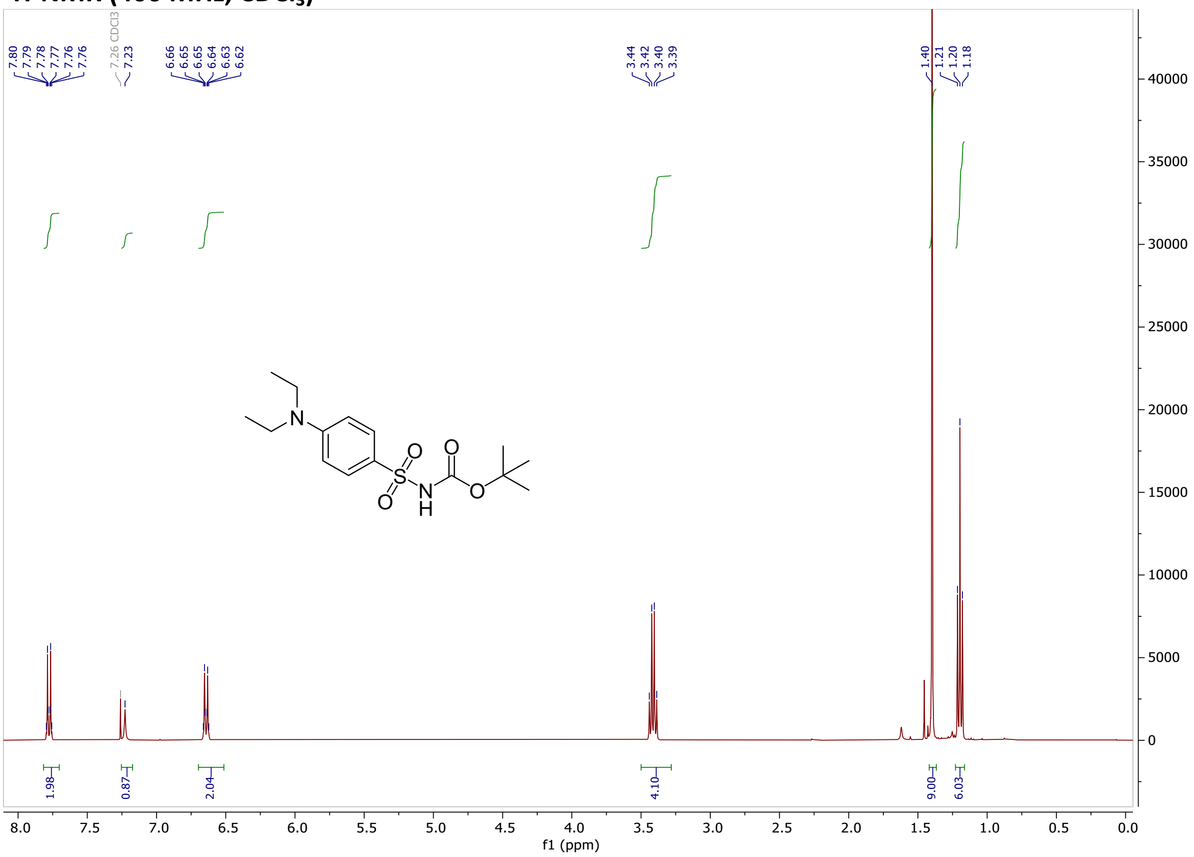

^{13}C-NMR (101 MHz, $CDCl_3$)

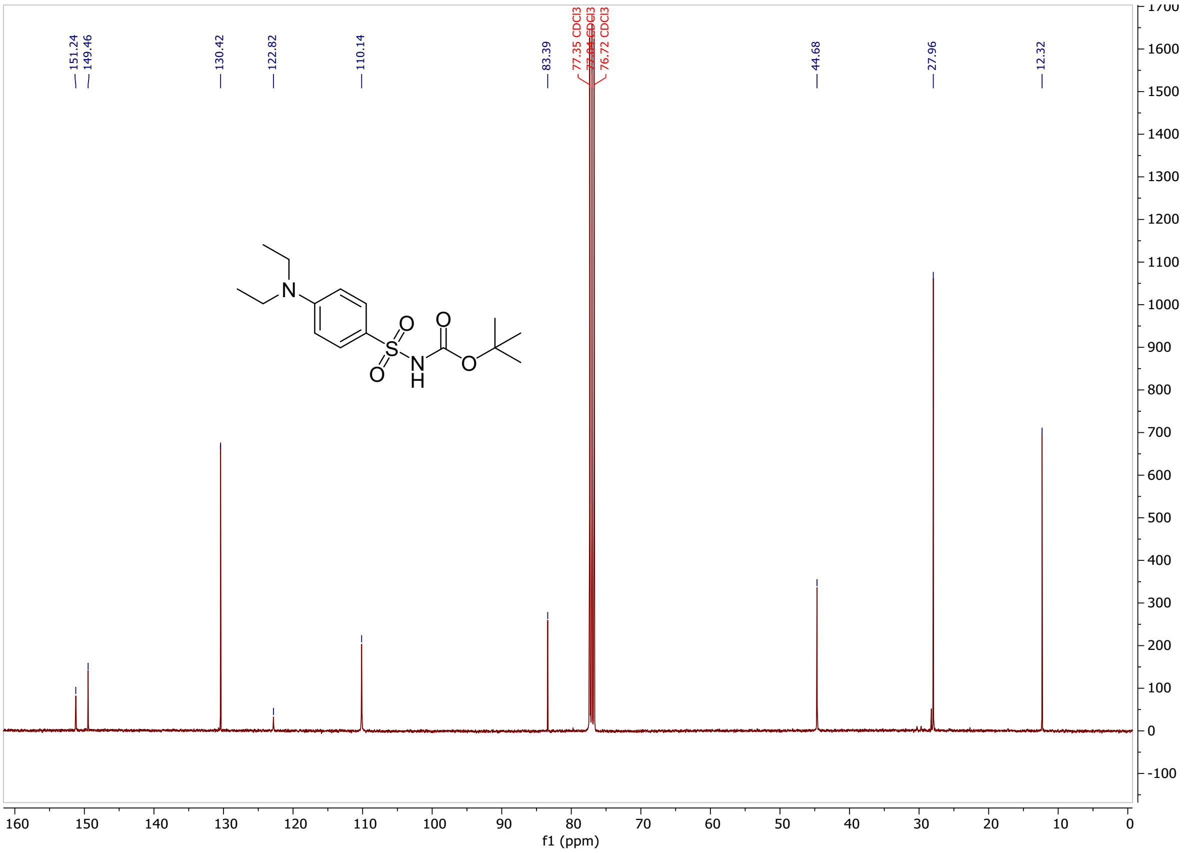

***tert*-Butyl ((4-(dibenzylamino)phenyl)sulfonyl)carbamate (4c)**

^{1}H-NMR (400 MHz, $CDCl_3$)

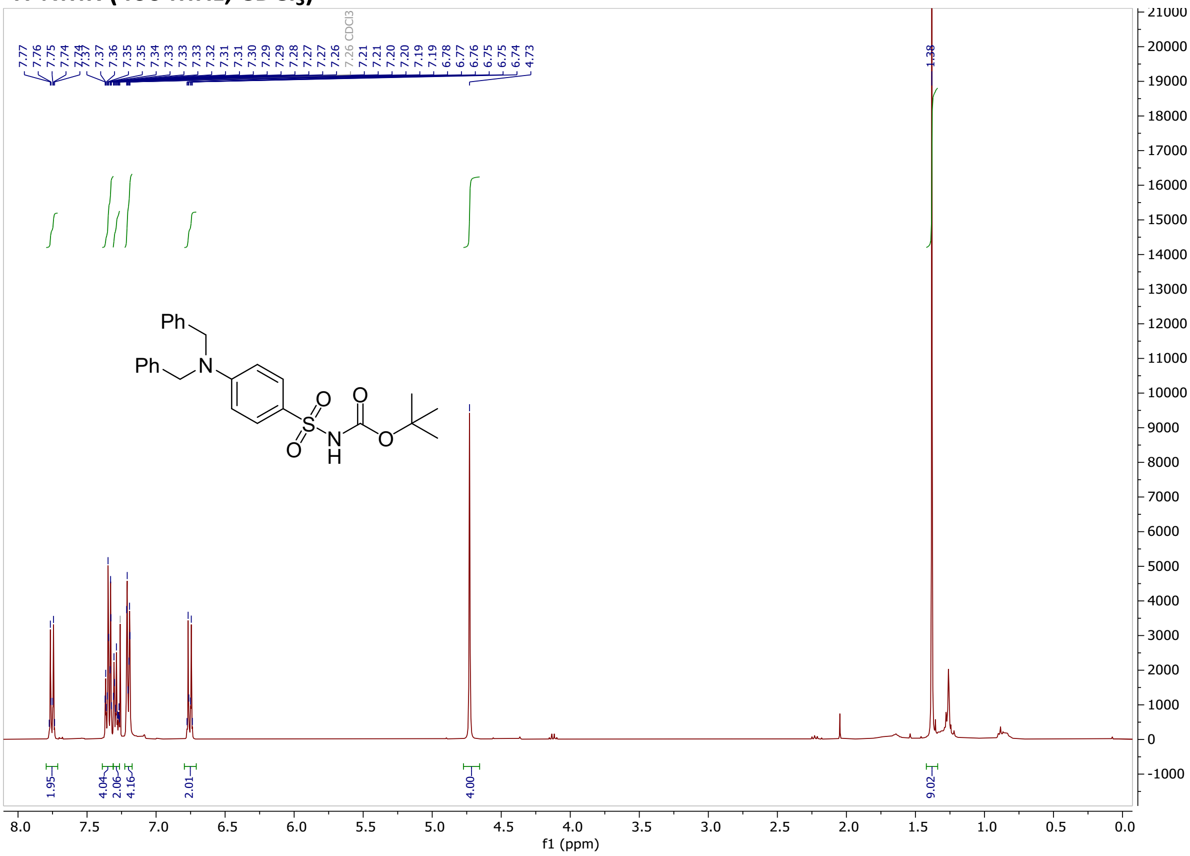

^{13}C-NMR (101 MHz, $CDCl_3$)

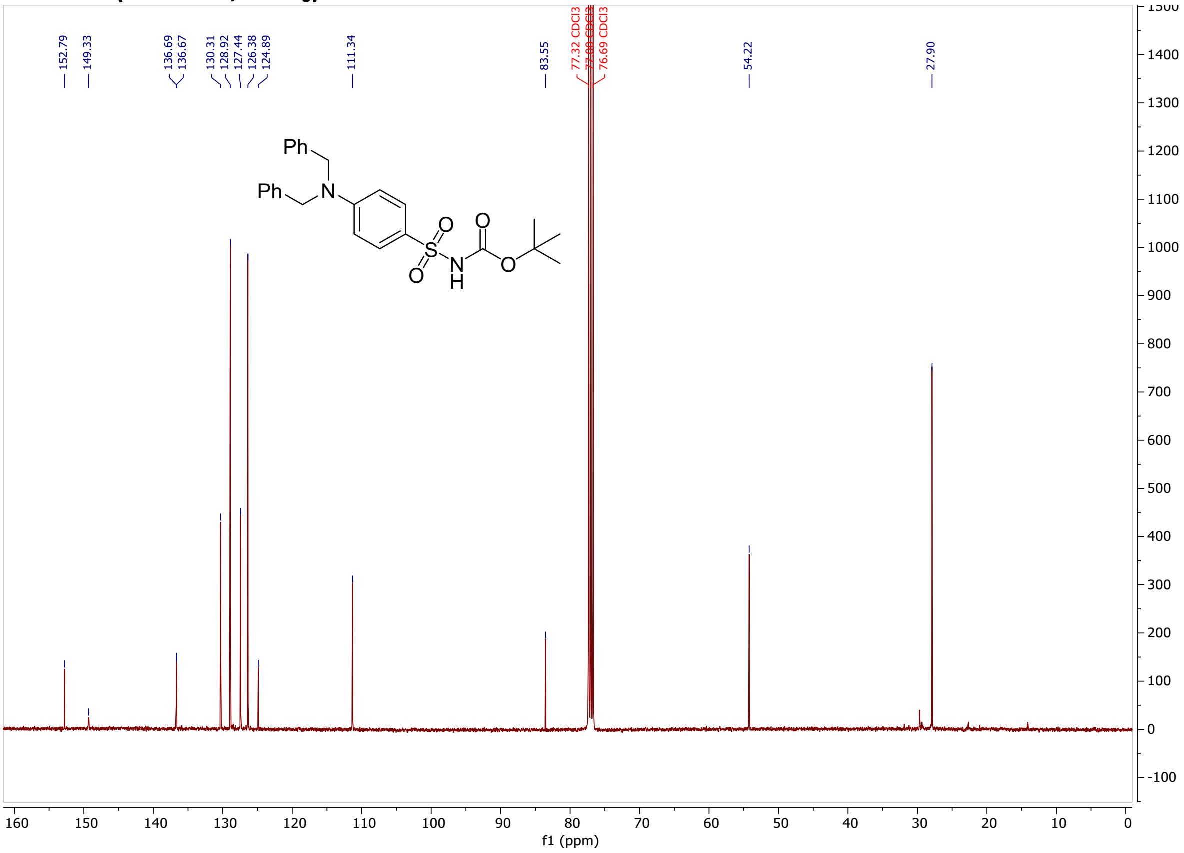

***tert*-Butyl ((4-(pyrrolidin-1-yl)phenyl)sulfonyl)carbamate (4d)**

^{1}H-NMR (400 MHz, $CDCl_3$)

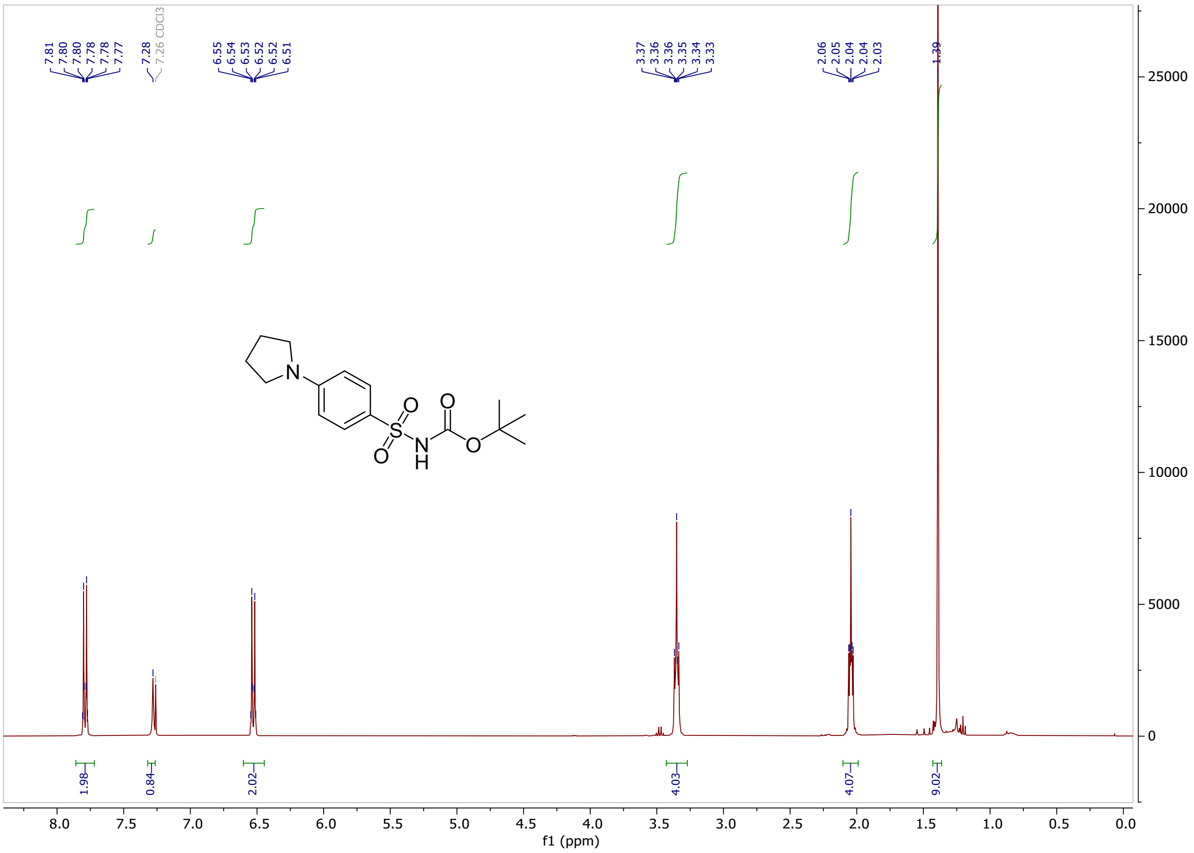

^{13}C-NMR (101 MHz, $CDCl_3$)

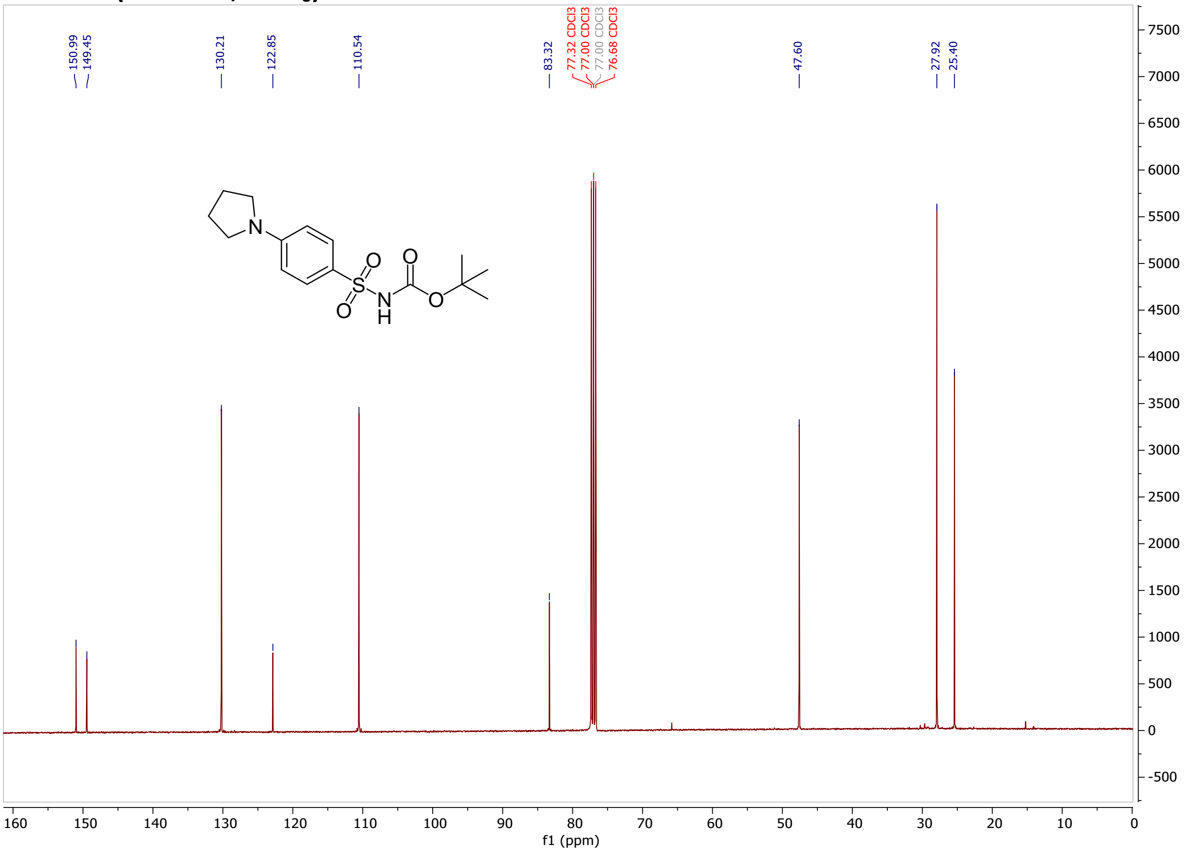

***tert*-Butyl ((4-(piperidin-1-yl)phenyl)sulfonyl)carbamate (4e)**

^{1}H-NMR (400 MHz, $CDCl_3$)

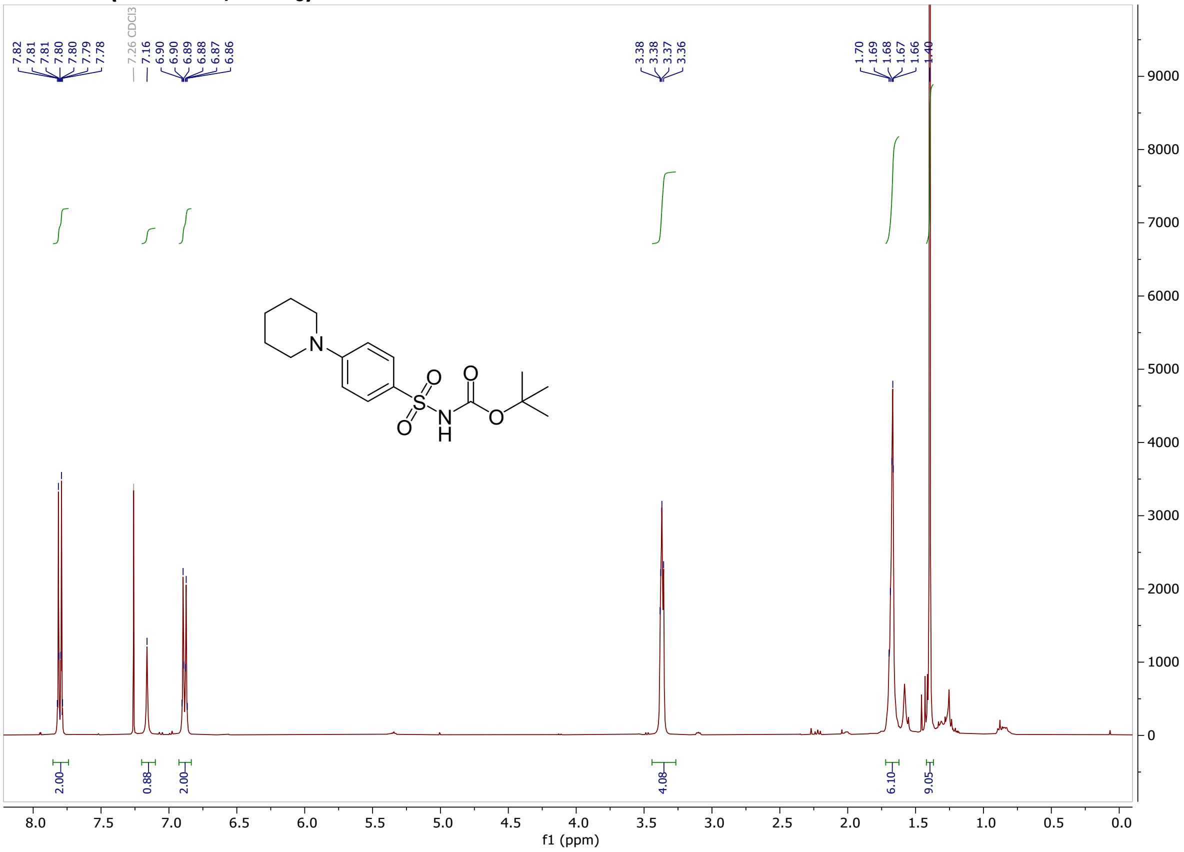

^{13}C-NMR (101 MHz, $CDCl_3$)

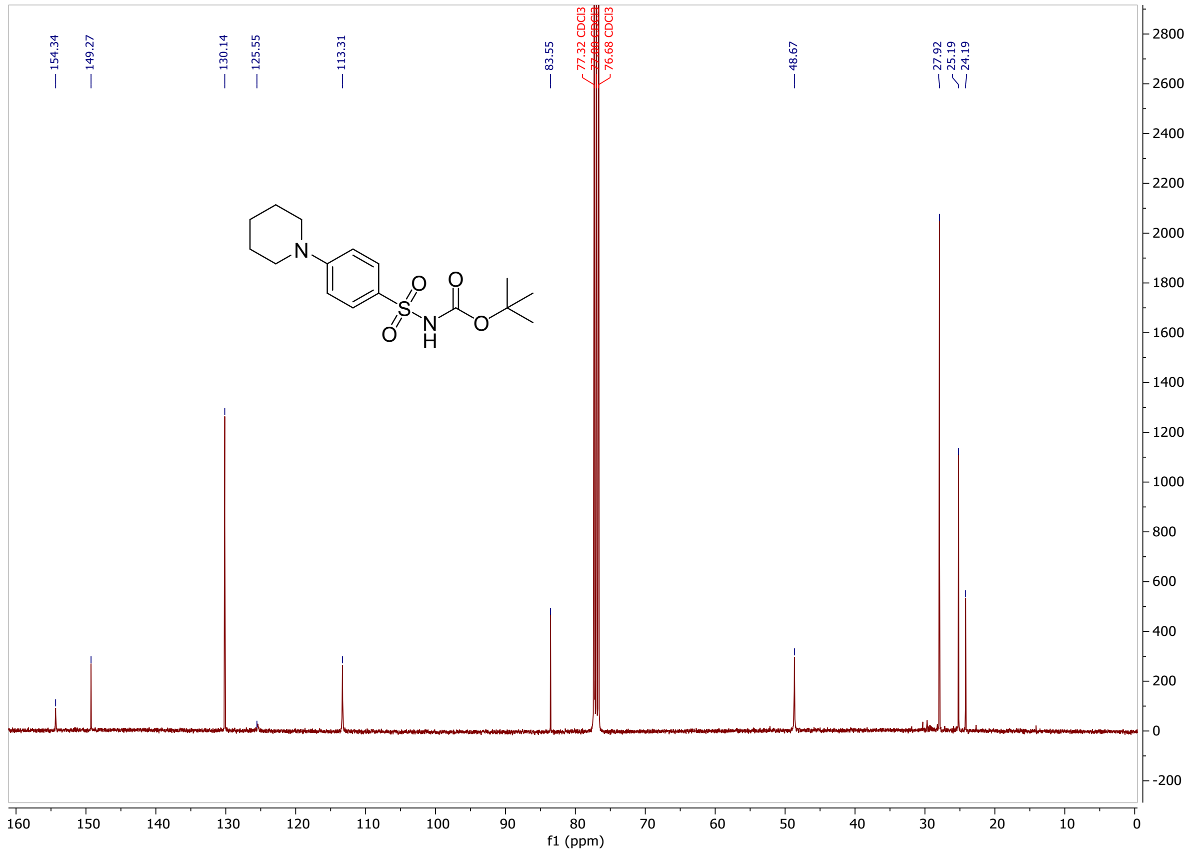

***tert*-Butyl ((4-morpholinophenyl)sulfonyl)carbamate (4f)**
^{1}H-NMR (400 MHz, $CDCl_3$)

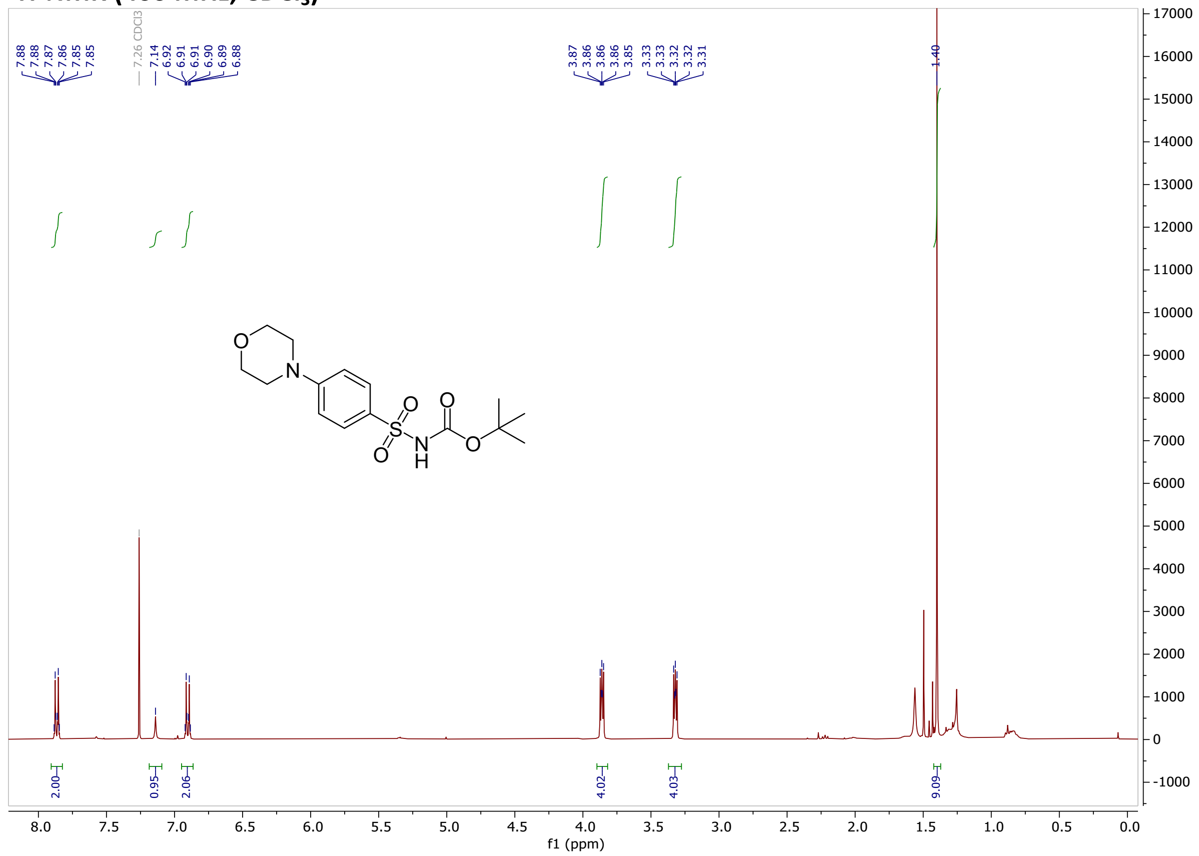

^{13}C-NMR (101 MHz, $CDCl_3$)

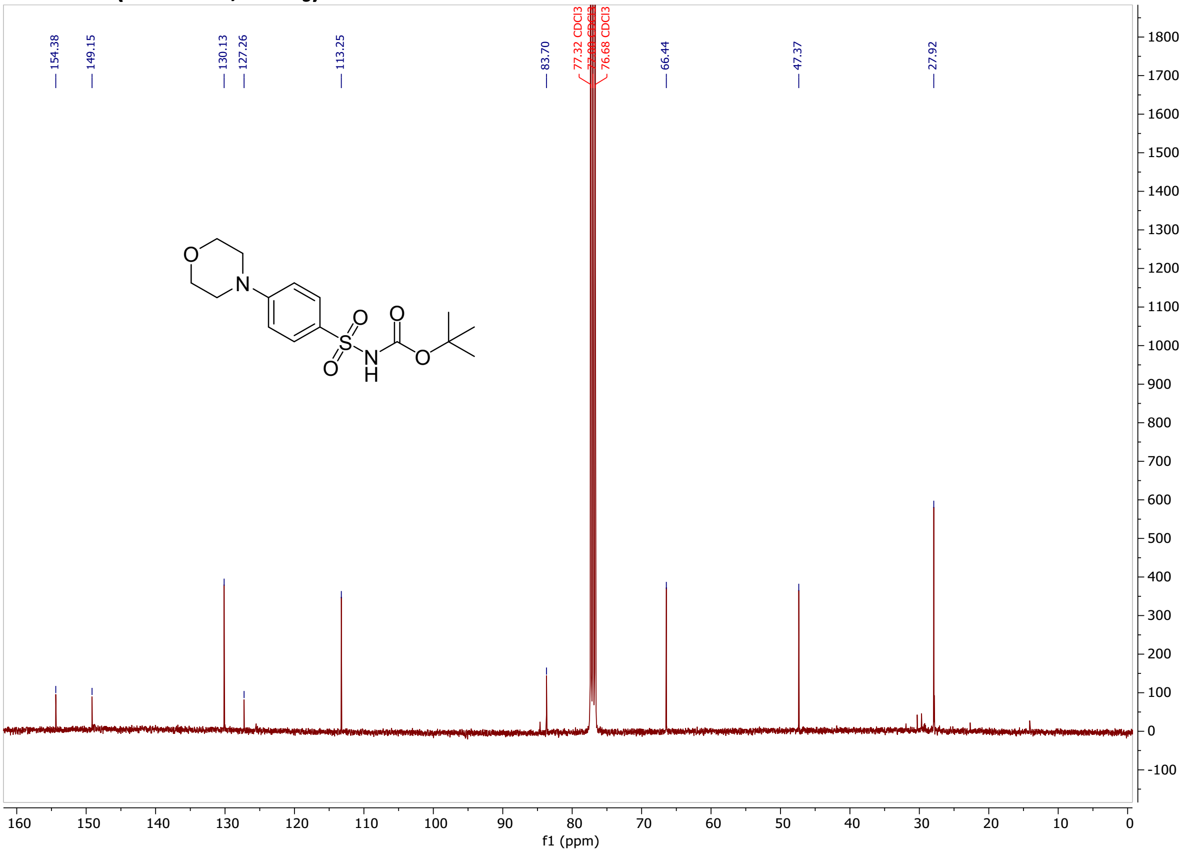

((4-(N-(*tert*-Butoxycarbonyl)sulfamoyl)phenyl)azanediyl)bis(ethane-2,1-diyl) diacetate (4g)

^{1}H-NMR (400 MHz, $CDCl_3$)

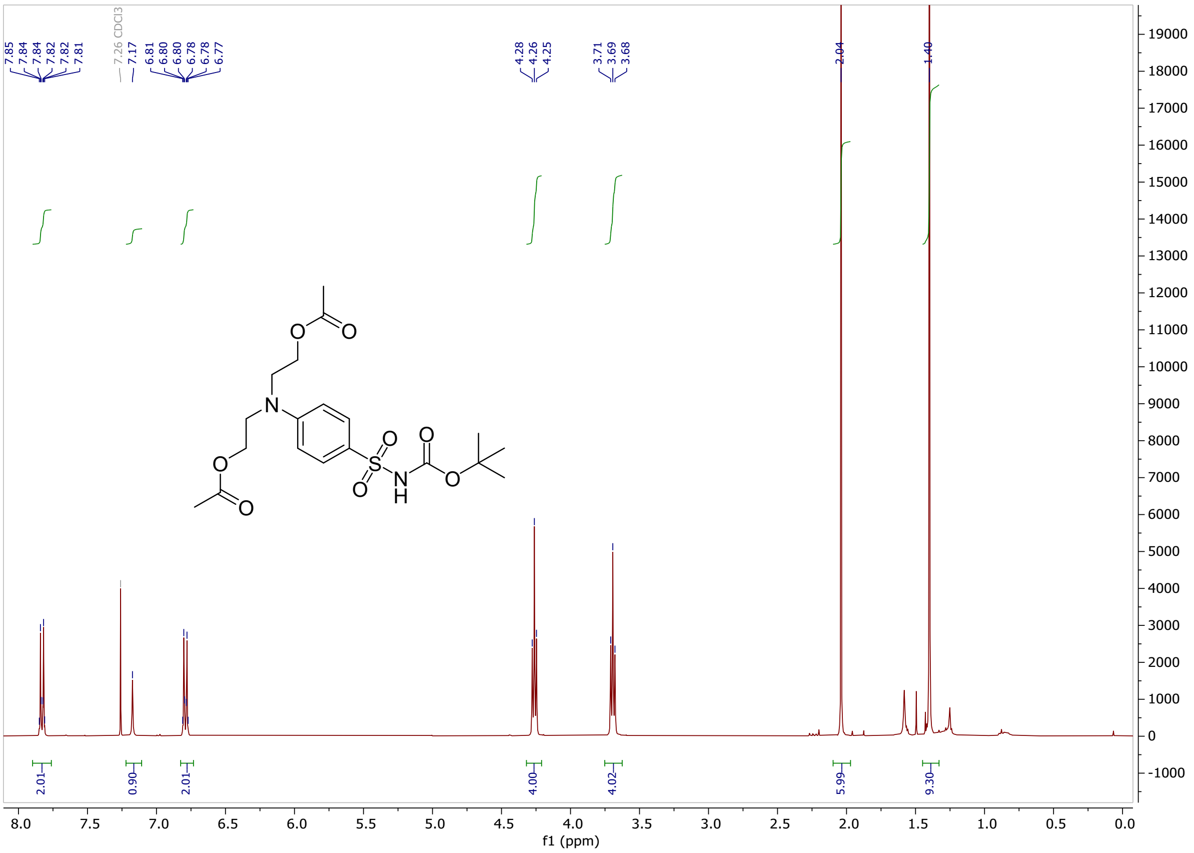

^{13}C-NMR (101 MHz, $CDCl_3$)

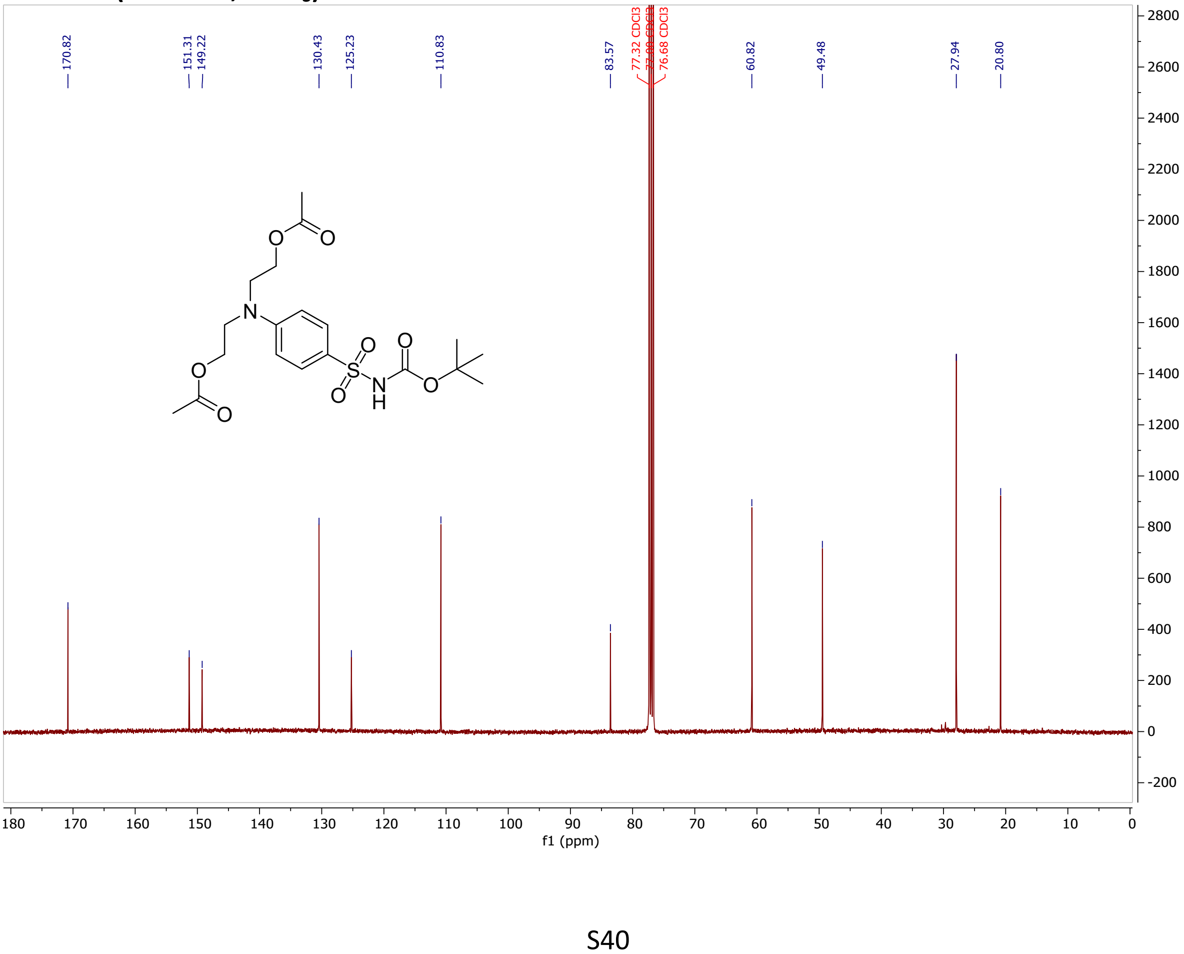

Diethyl 2,2'-((4-(*N*-(*tert*-butoxycarbonyl)sulfamoyl)phenyl)azanediyl)diacetate (4h)

^{1}H-NMR (400 MHz, $CDCl_3$)

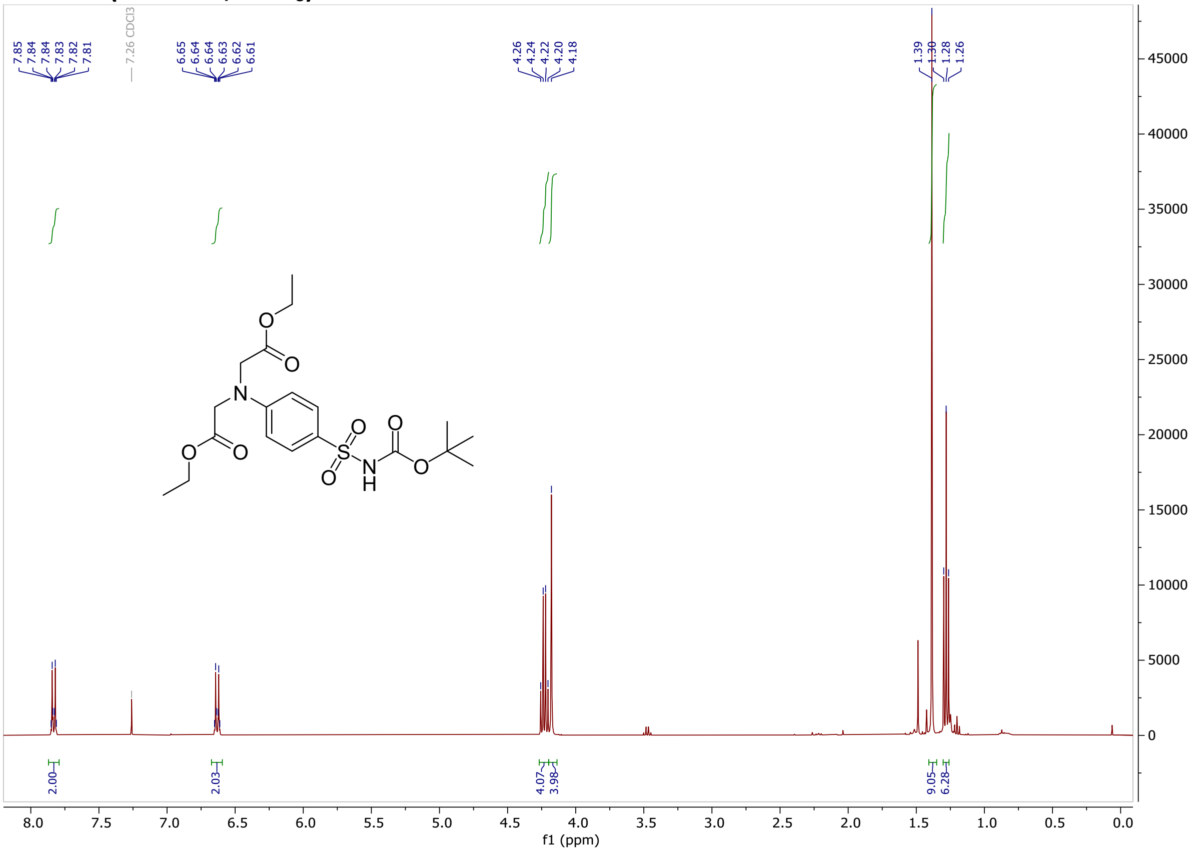

^{13}C-NMR (101 MHz, $CDCl_3$)

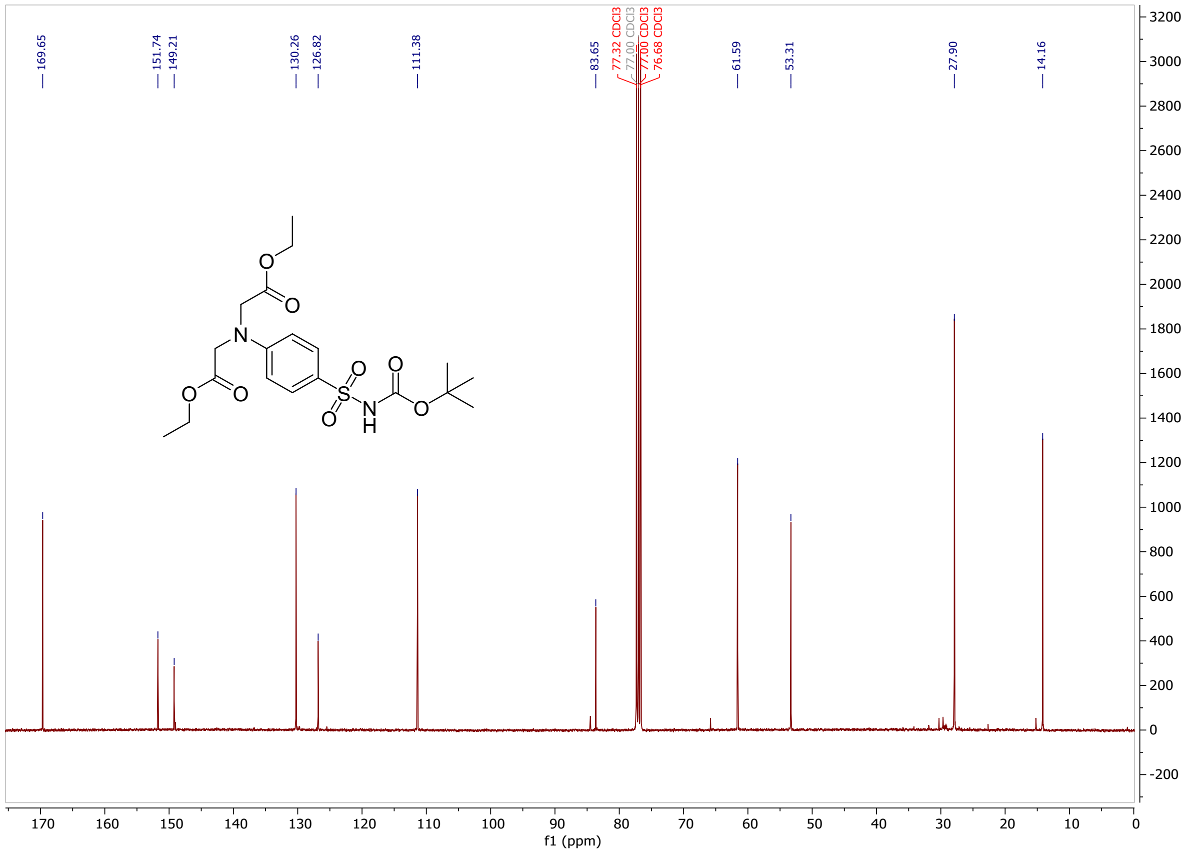

***tert*-Butyl ((4-(diphenylamino)phenyl)sulfonyl)carbamate (4i)**
^{1}H-NMR (400 MHz, $CDCl_3$)

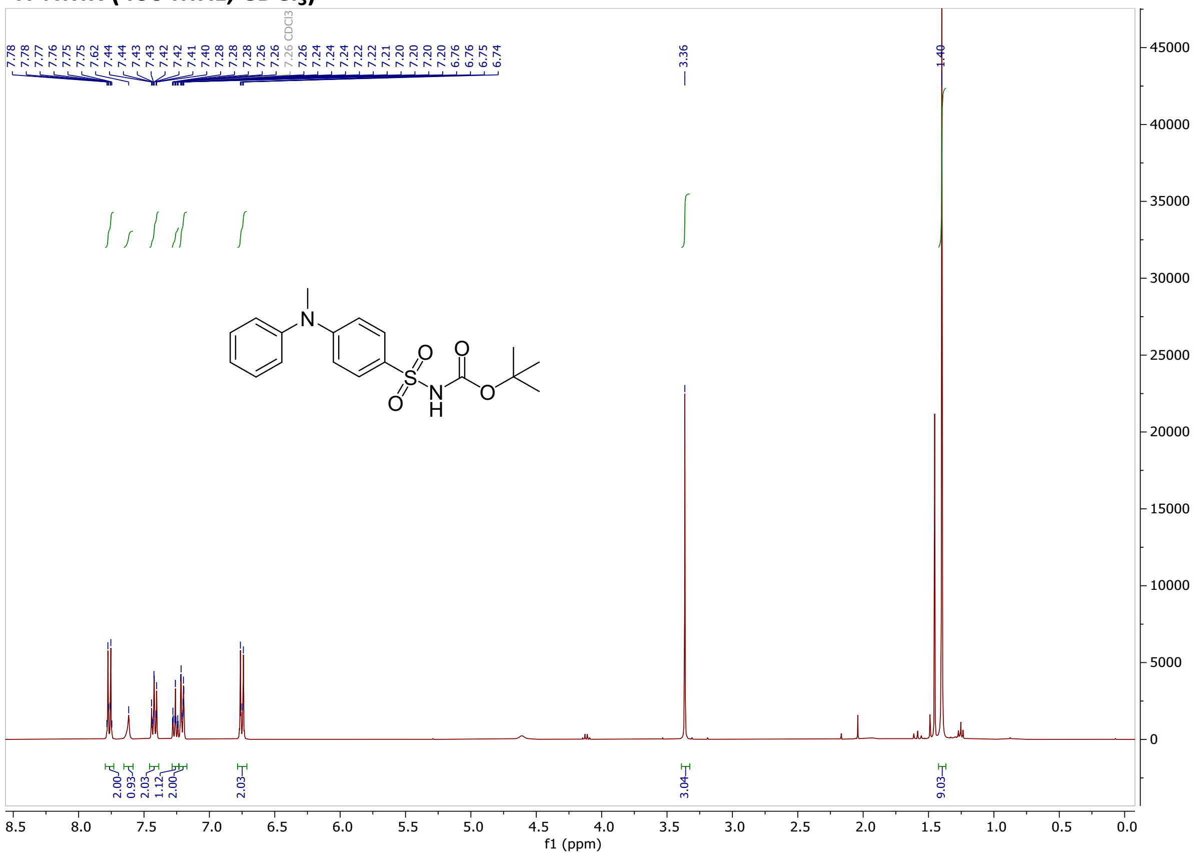

^{13}C-NMR (101 MHz, CDCl$_3$)

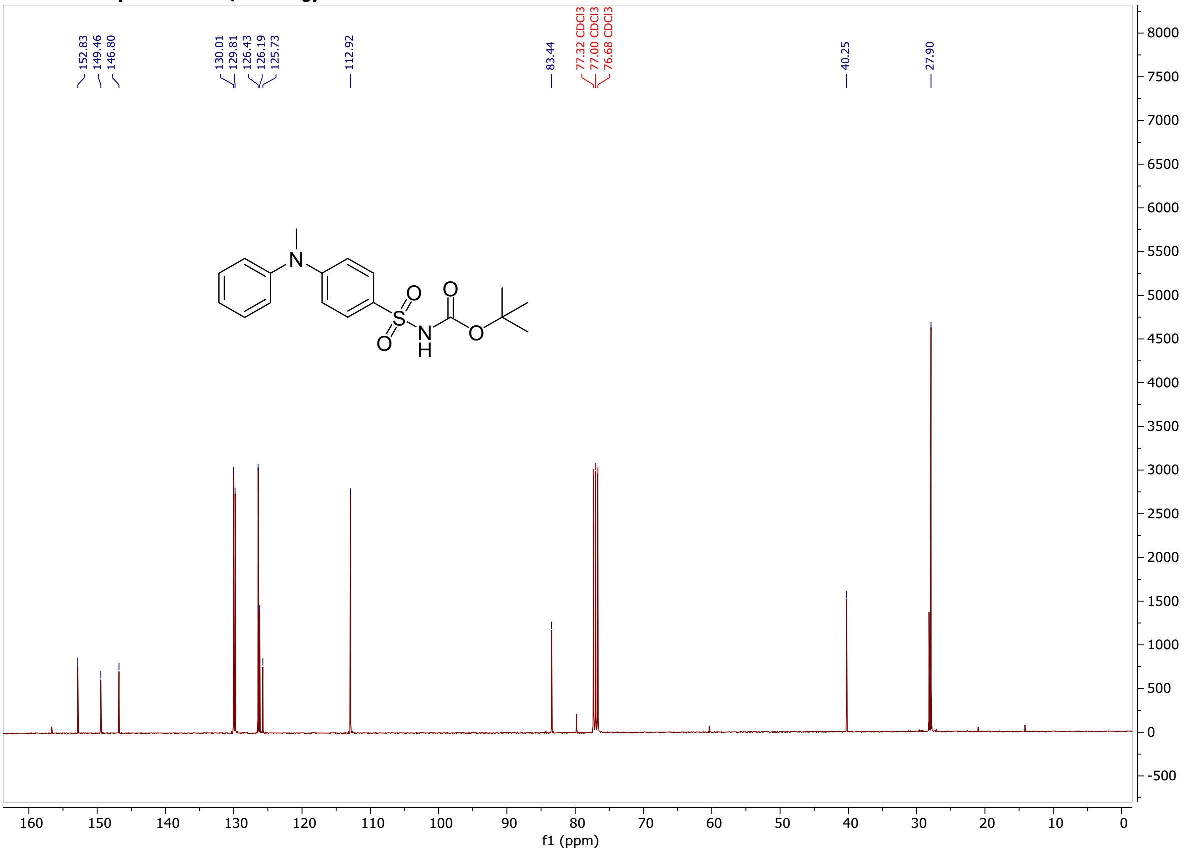

***tert*-Butyl ((4-(diphenylamino)phenyl)sulfonyl)carbamate (4j)**

^{1}H-NMR (400 MHz, CDCl$_3$)

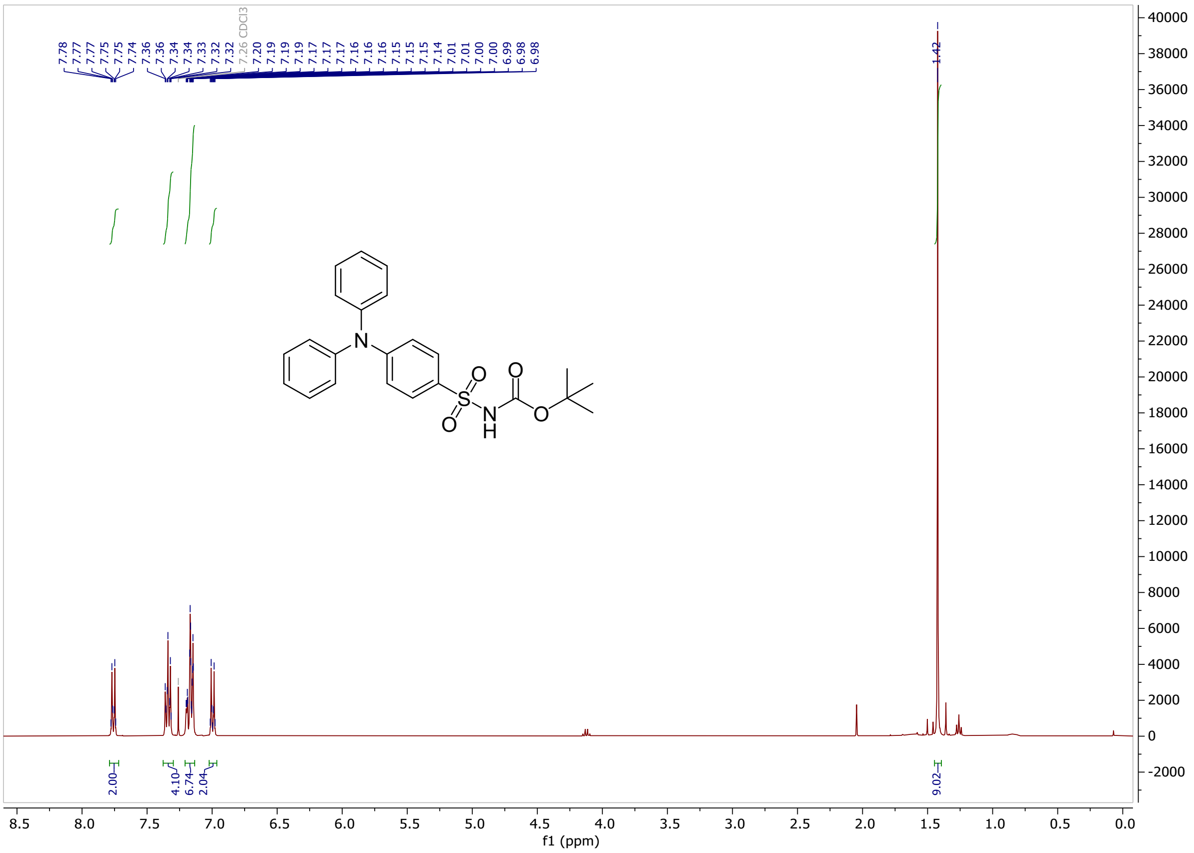

^{13}C-NMR (101 MHz, $CDCl_3$)

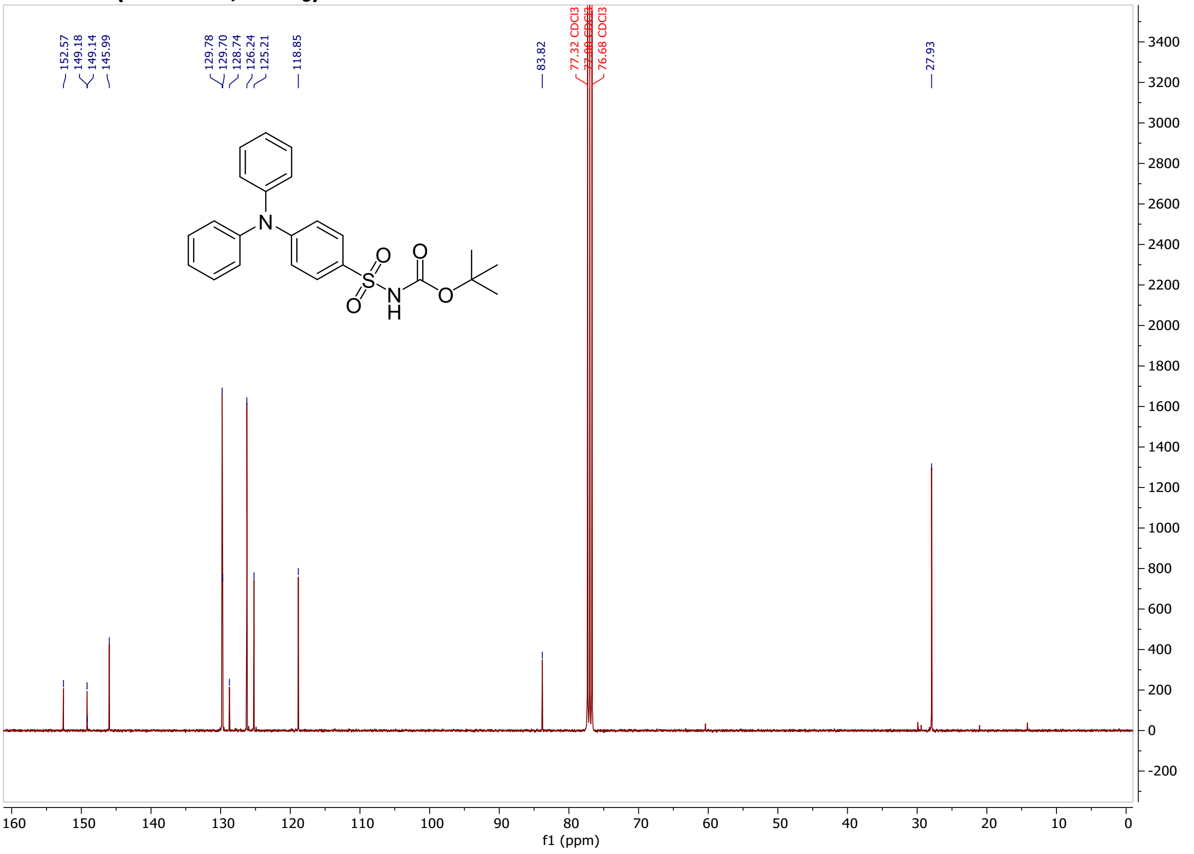

***tert*-Butyl ((4-(dimethylamino)-3-methylphenyl)sulfonyl)carbamate (4k)**

^{1}H-NMR (400 MHz, CDCl$_3$)

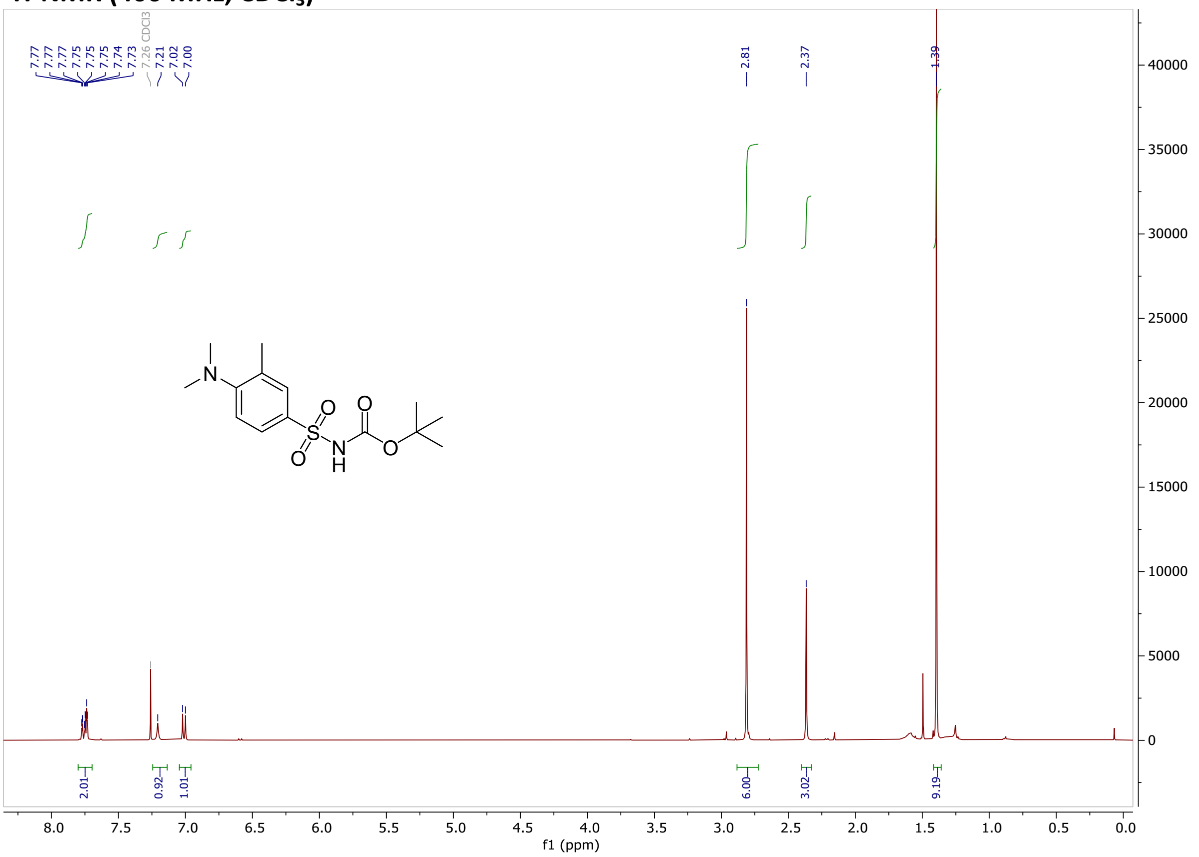

^{13}C-NMR (101 MHz, CDCl$_3$)

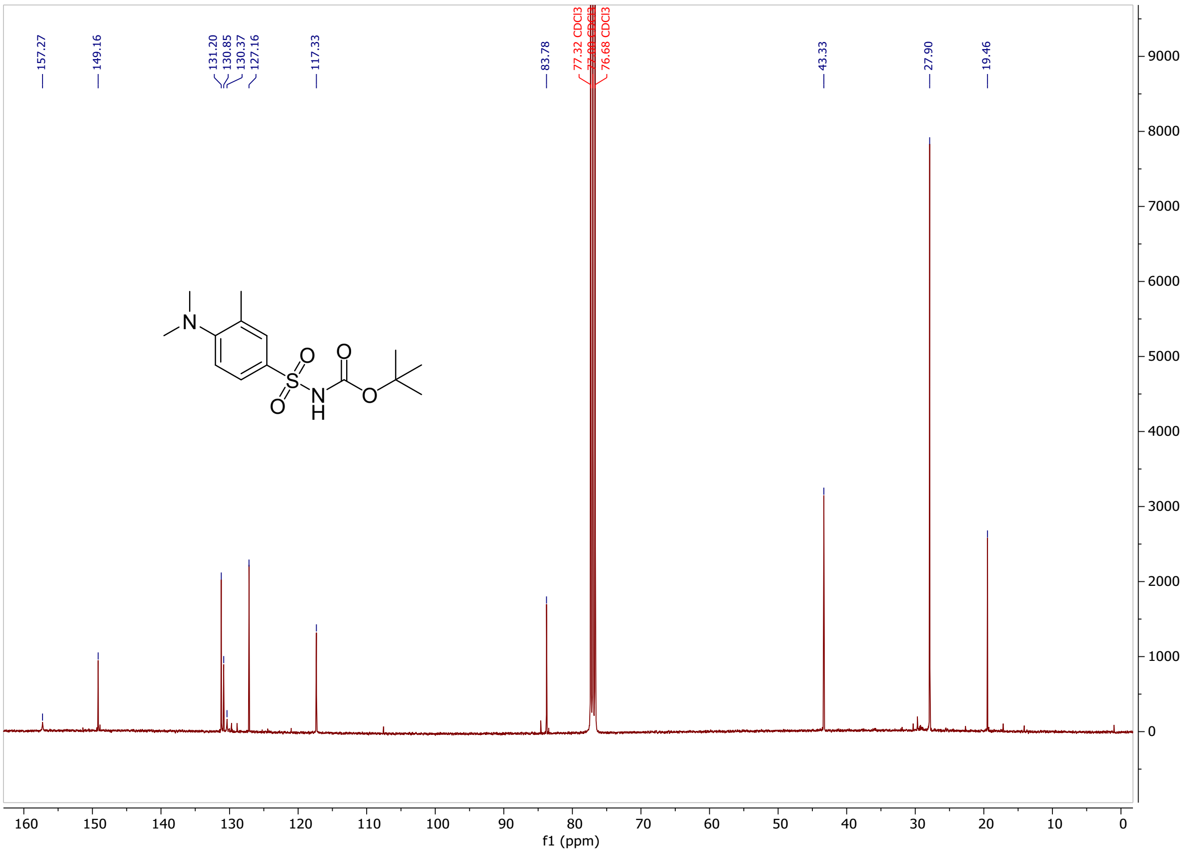

***tert*-Butyl ((4-(dimethylamino)-3,5-dimethylphenyl)sulfonyl)carbamate (4l)**

^{1}H-NMR (400 MHz, $CDCl_3$)

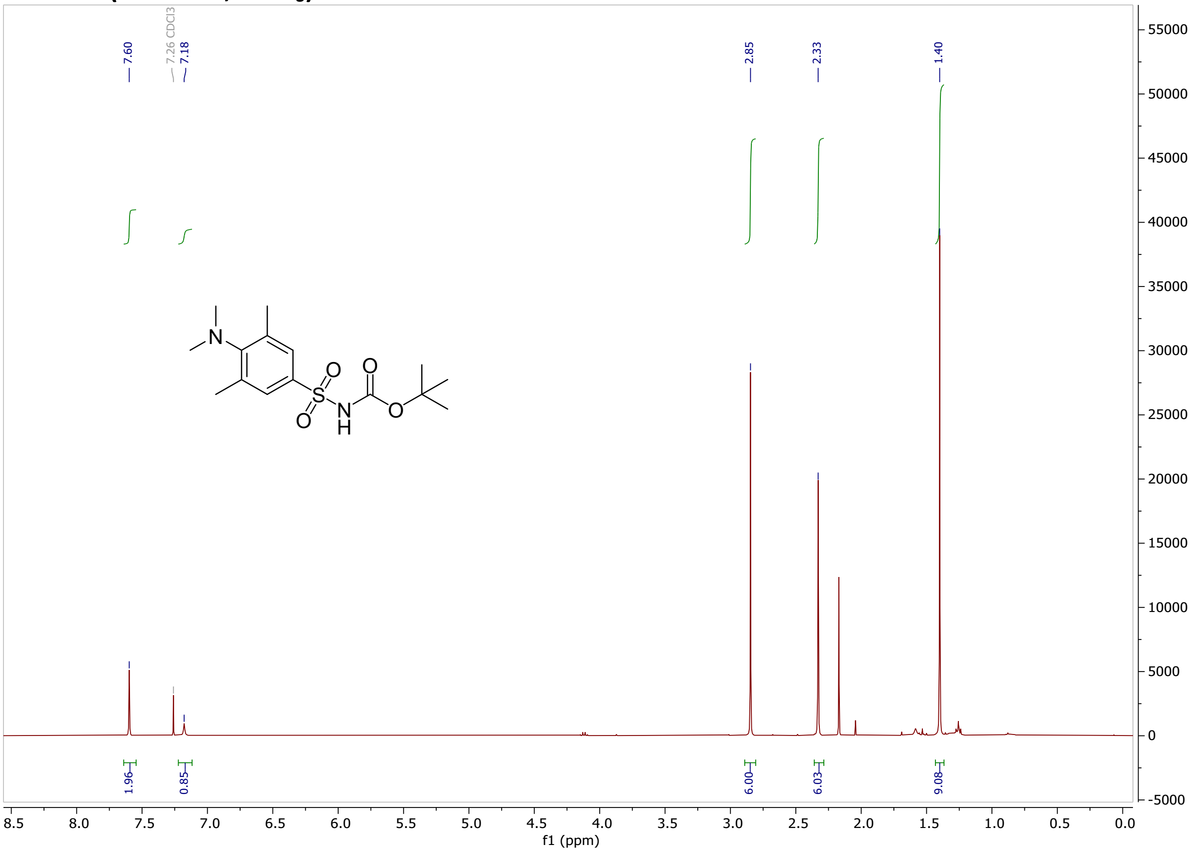

^{13}C-NMR (101 MHz, CDCl$_3$)

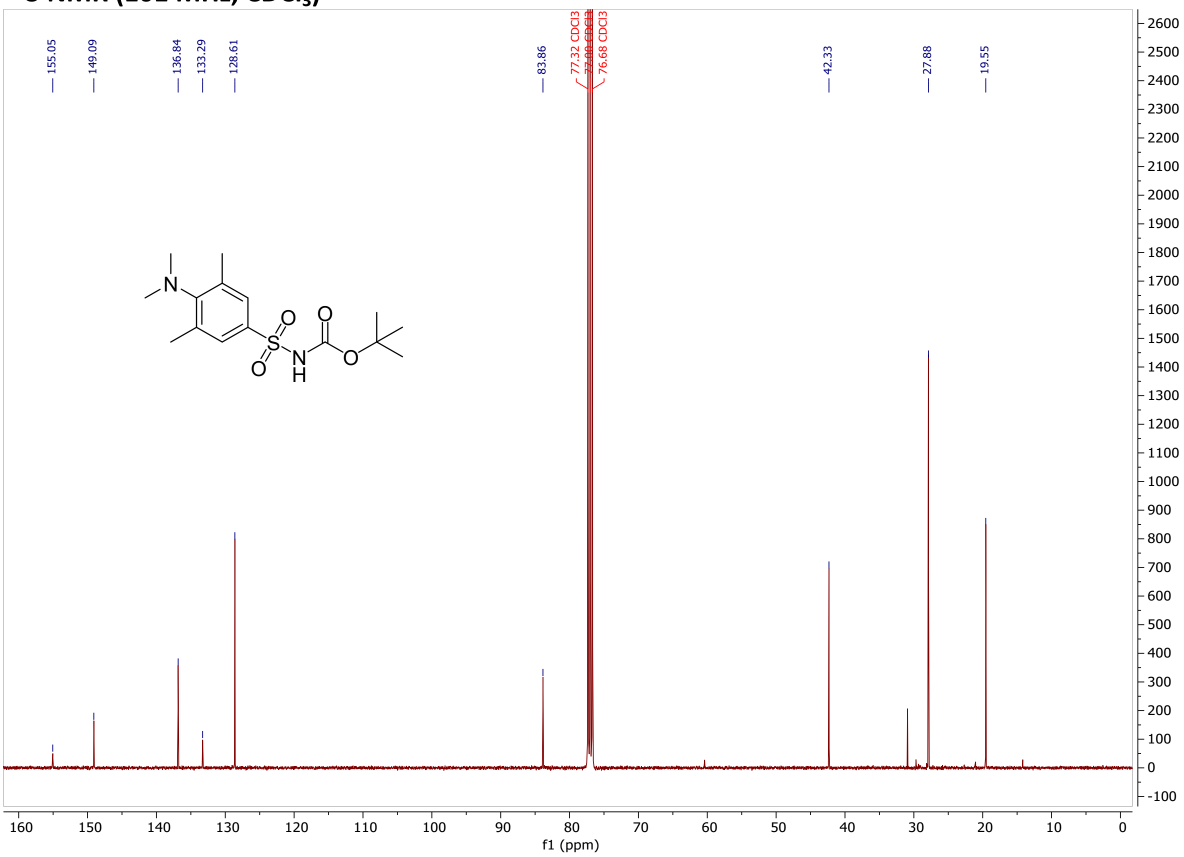

***tert*-Butyl ((4-(dimethylamino)-2-methylphenyl)sulfonyl)carbamate (4m)**

^{1}H-NMR (400 MHz, CDCl$_3$)

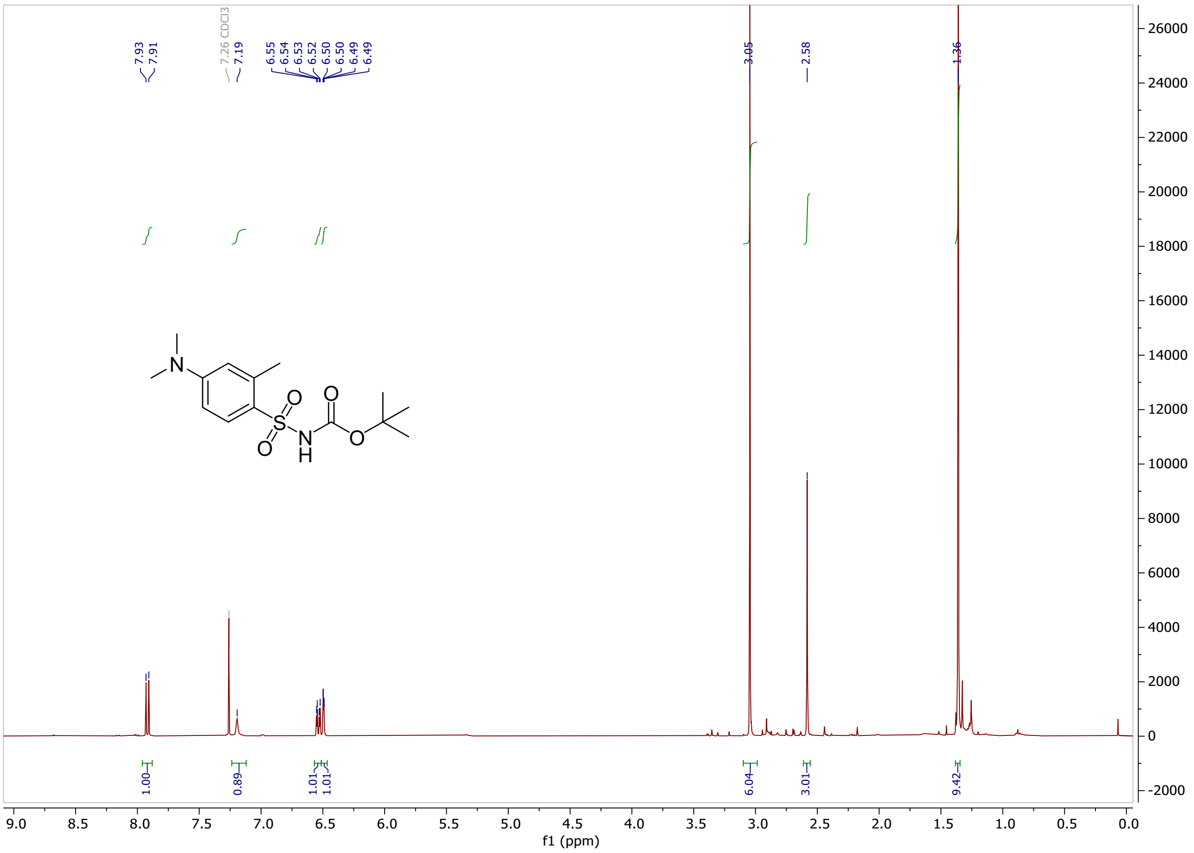

^{13}C-NMR (101 MHz, $CDCl_3$)

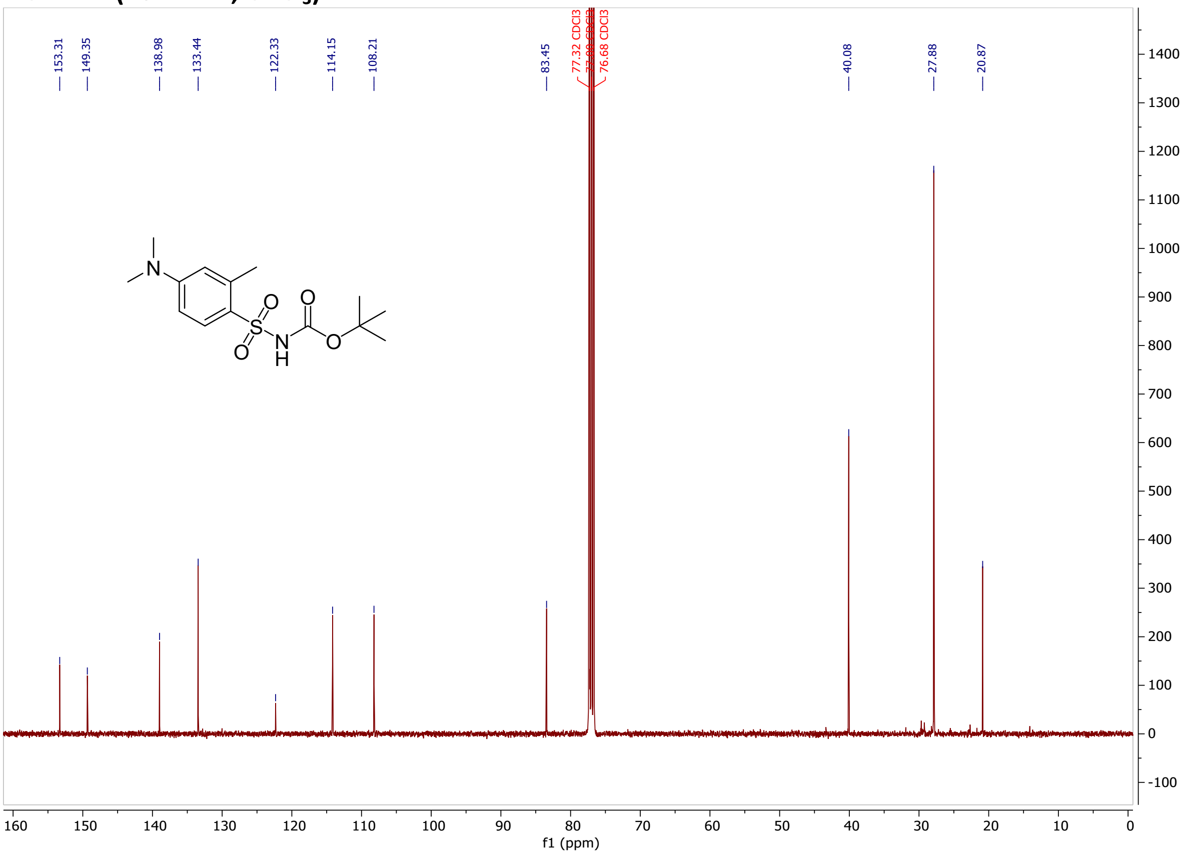

2-(*N*-(*tert*-Butoxycarbonyl)sulfamoyl)-5-(dimethylamino)phenyl acetate (4n)

^{1}H-NMR (400 MHz, CDCl$_3$)

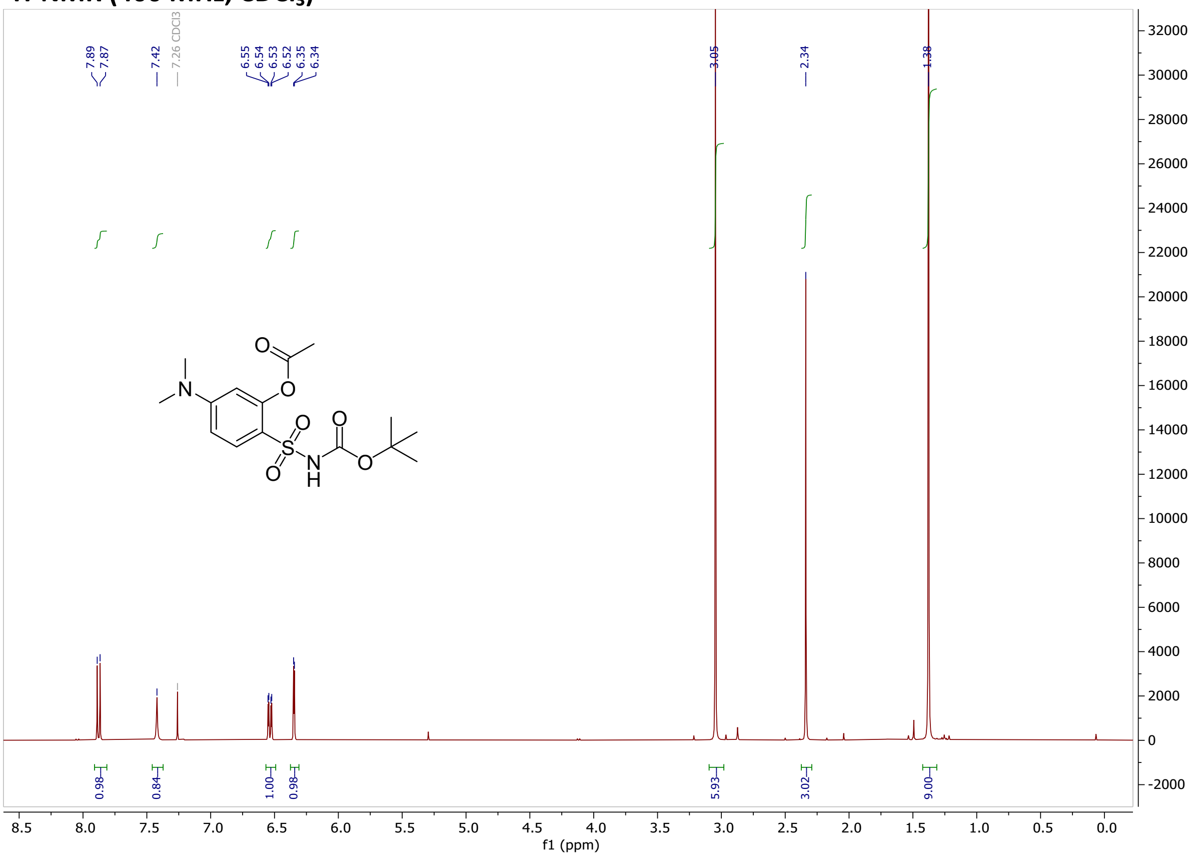

^{13}C-NMR (101 MHz, CDCl$_3$)

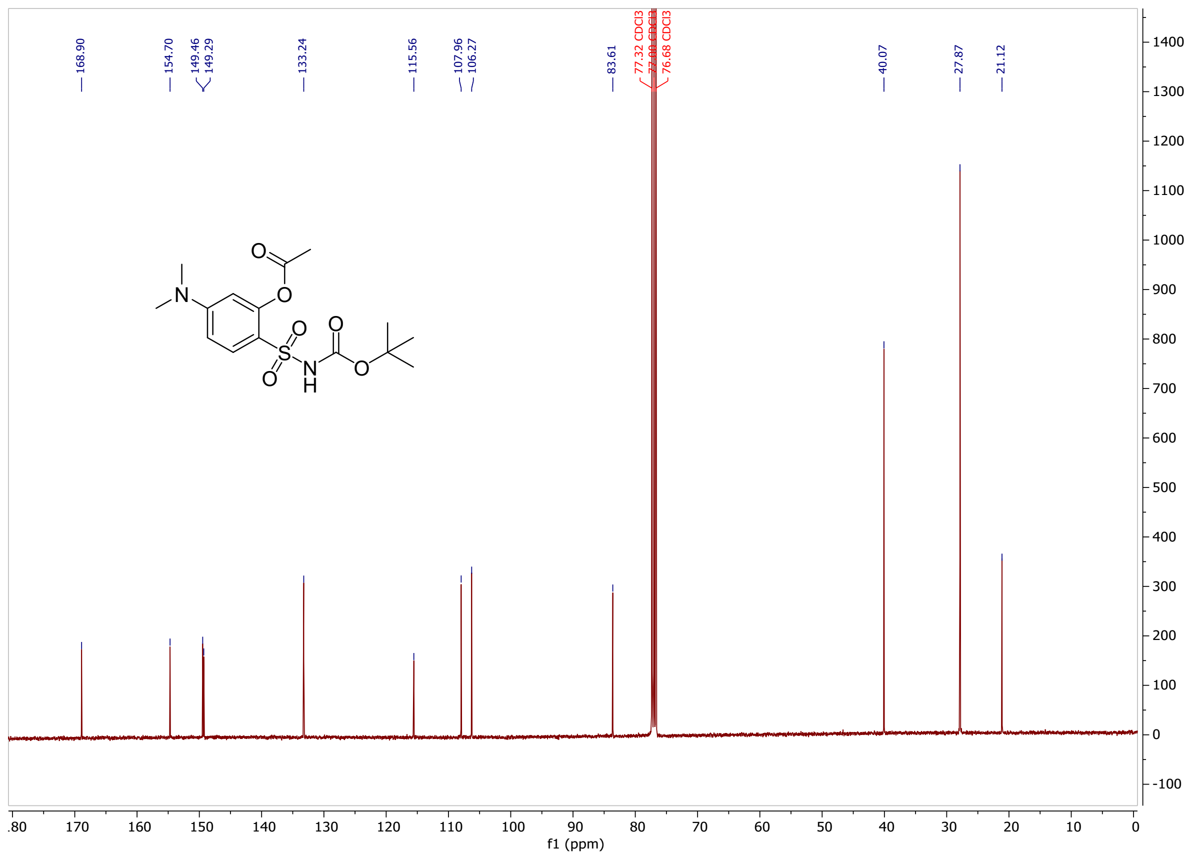

4-(*N*-(*tert*-Butoxycarbonyl)sulfamoyl)-3-(dimethylamino)phenyl acetate (4n')
^{1}H-NMR (400 MHz, $CDCl_3$)

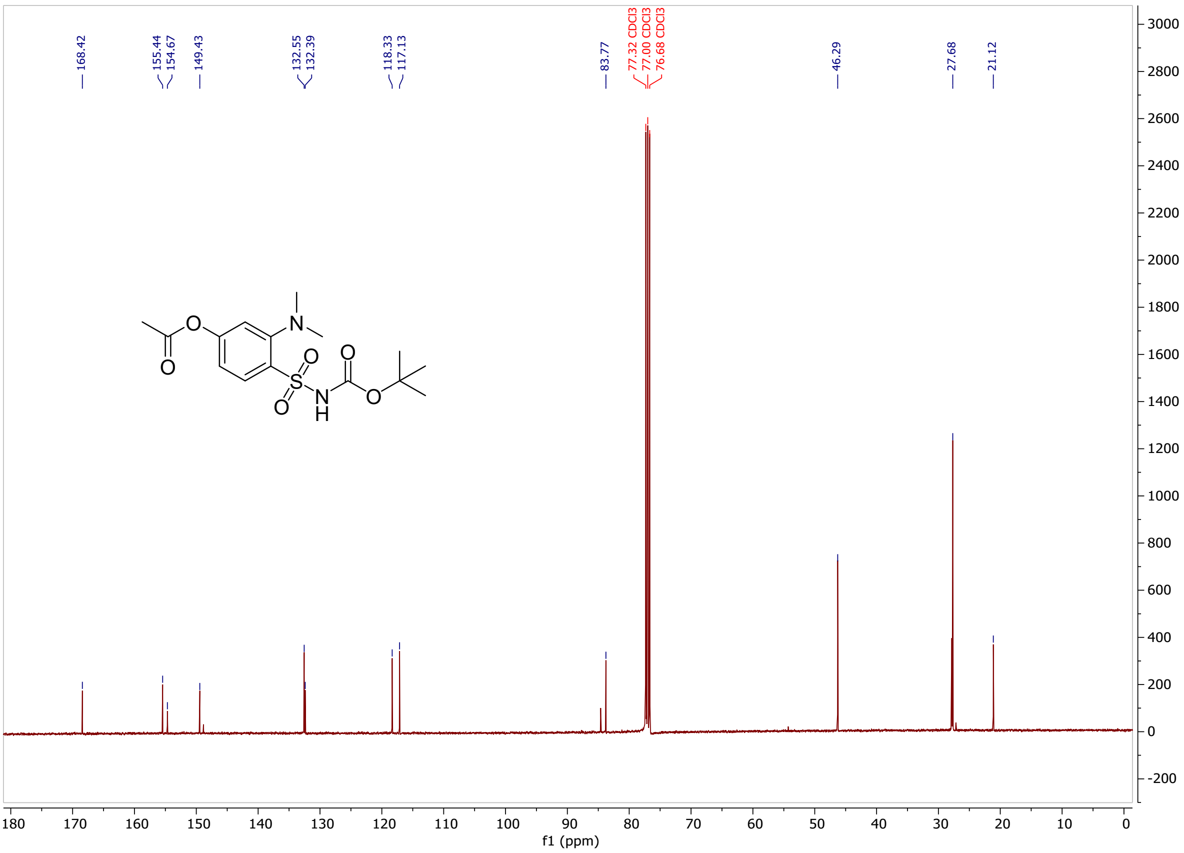

^{13}C-NMR (101 MHz, CDCl$_3$)

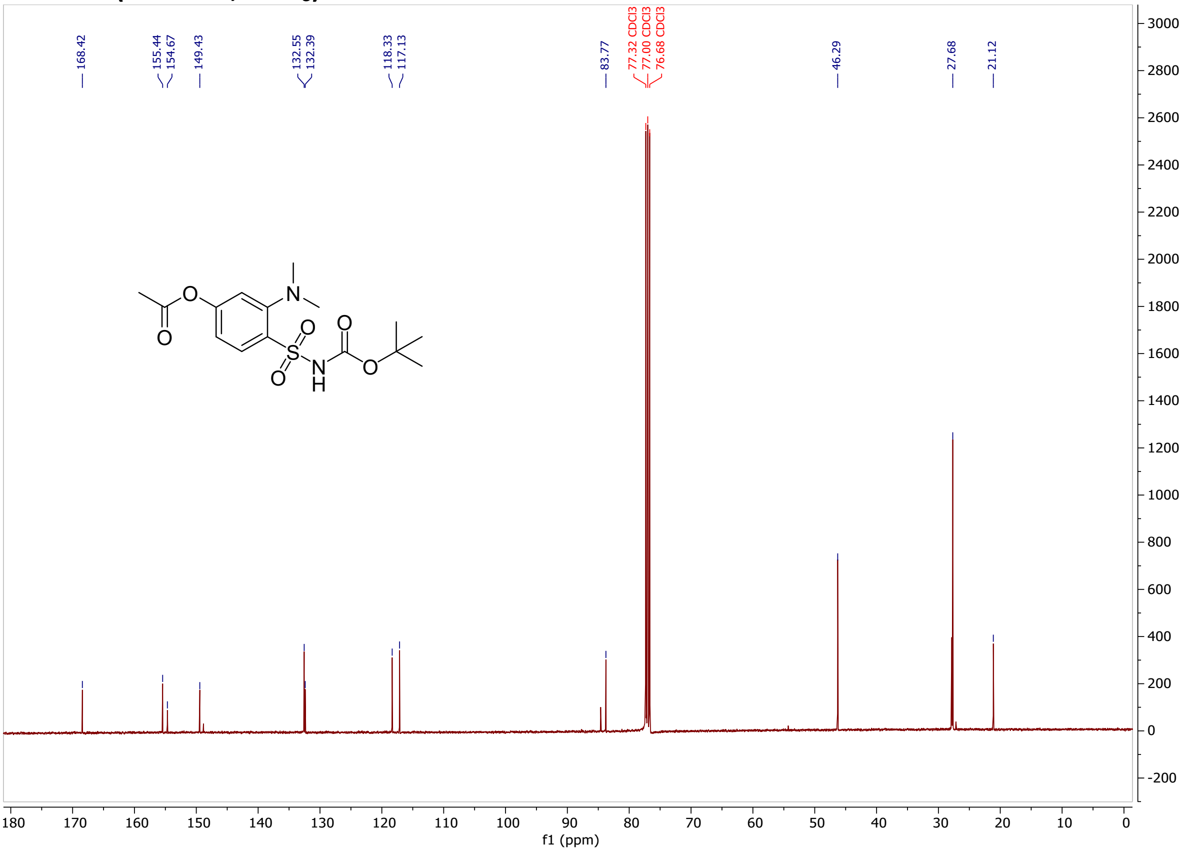
168.42
155.44
154.67
149.43
132.55
132.39
118.33
117.13
83.77
77.32 CDCl3
77.00 CDCl3
76.68 CDCl3
46.29
27.68
21.12
f1 (ppm)

***tert*-Butyl ((4-(dimethylamino)-2-methoxyphenyl)sulfonyl)carbamate (4o)**

^{1}H-NMR (400 MHz, $CDCl_3$)

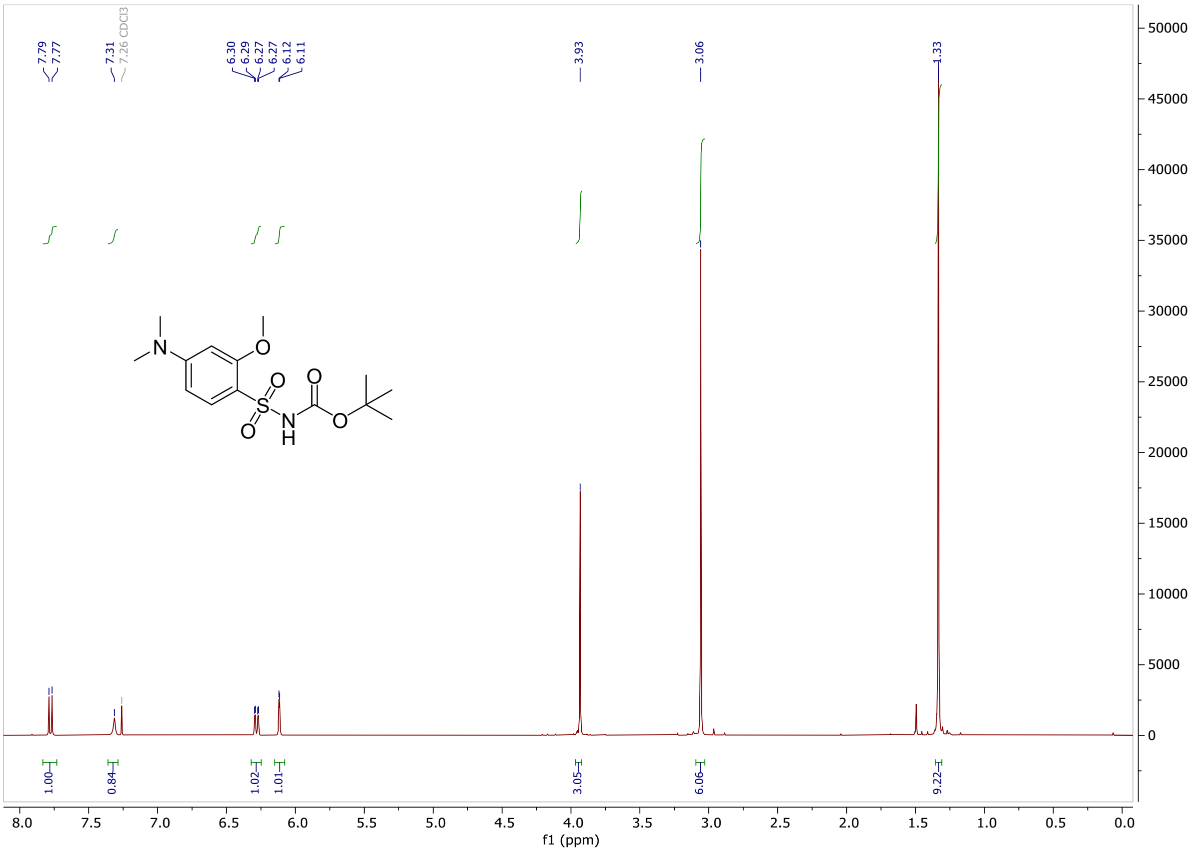

^{13}C-NMR (101 MHz, $CDCl_3$)

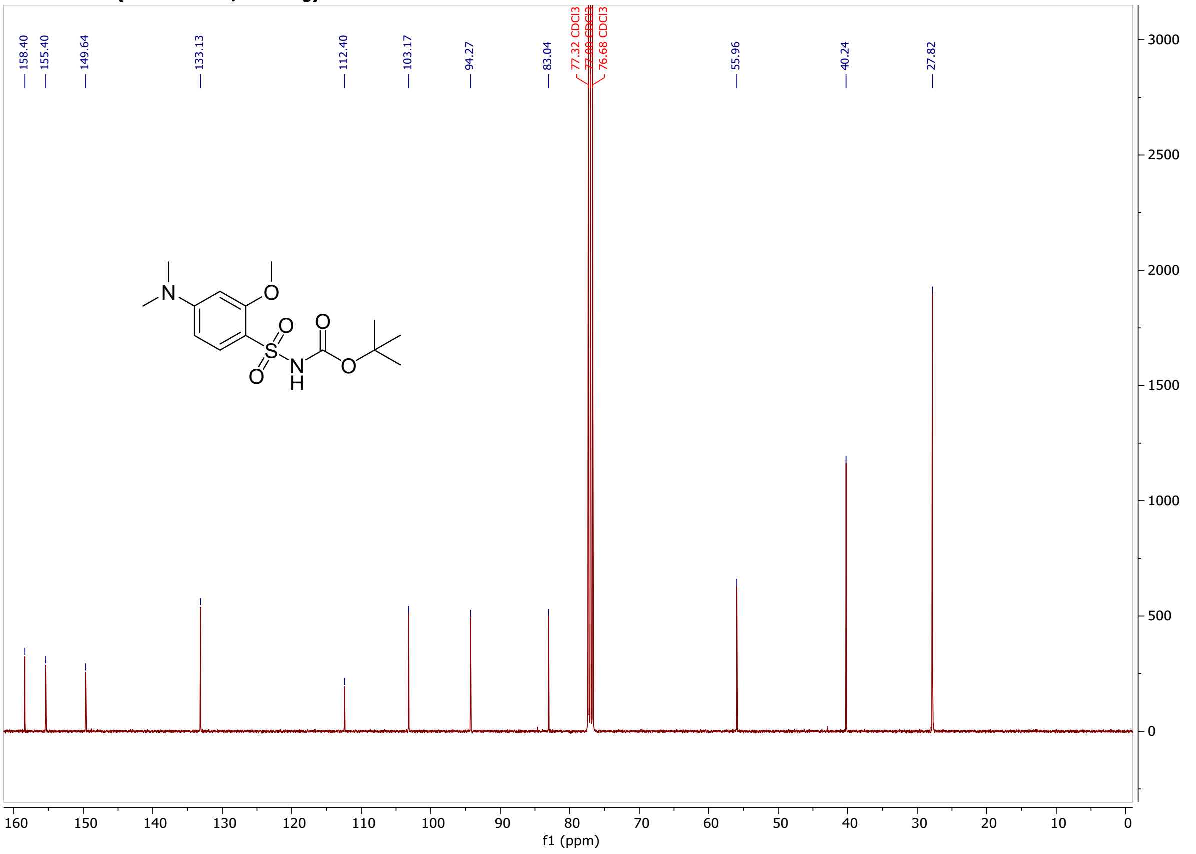

***tert*-Butyl ((2-(dimethylamino)-4-methoxyphenyl)sulfonyl)carbamate (4o')**

^{1}H-NMR (400 MHz, $CDCl_3$)

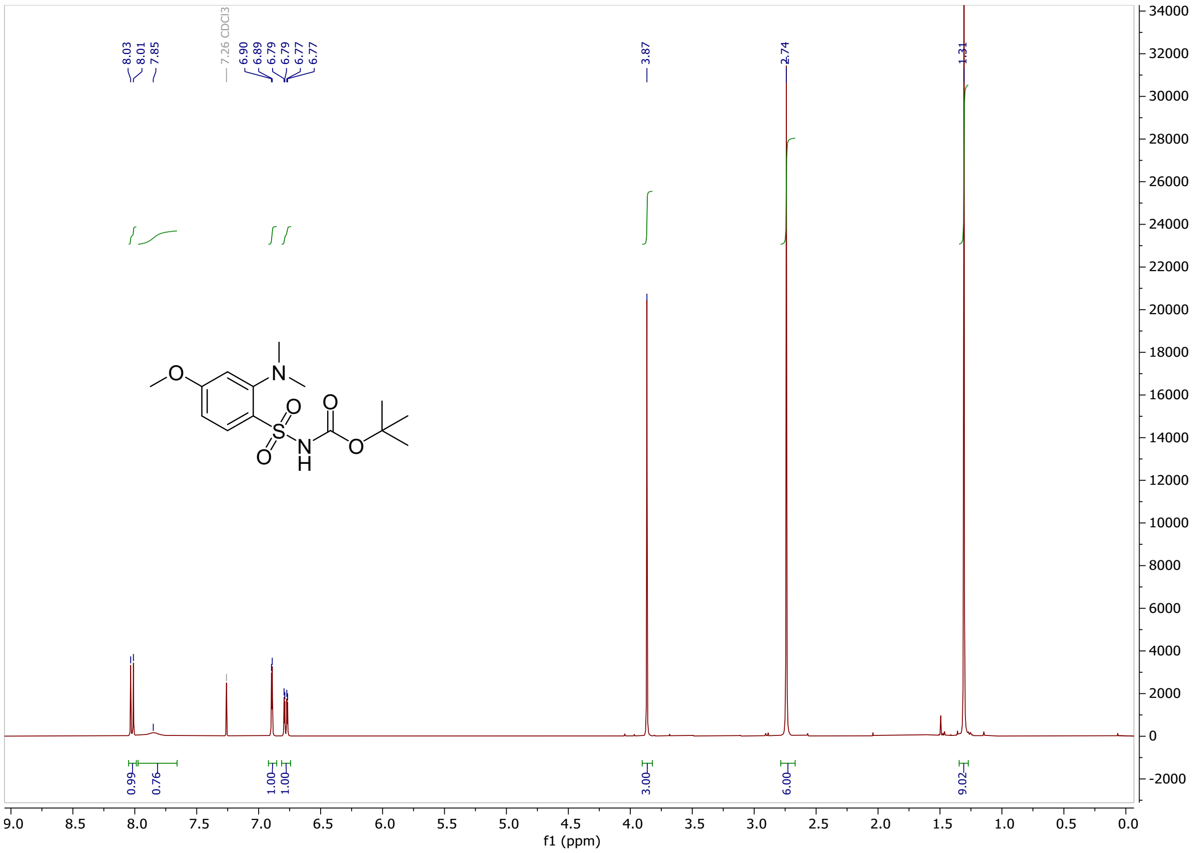

^{13}C-NMR (101 MHz, $CDCl_3$)

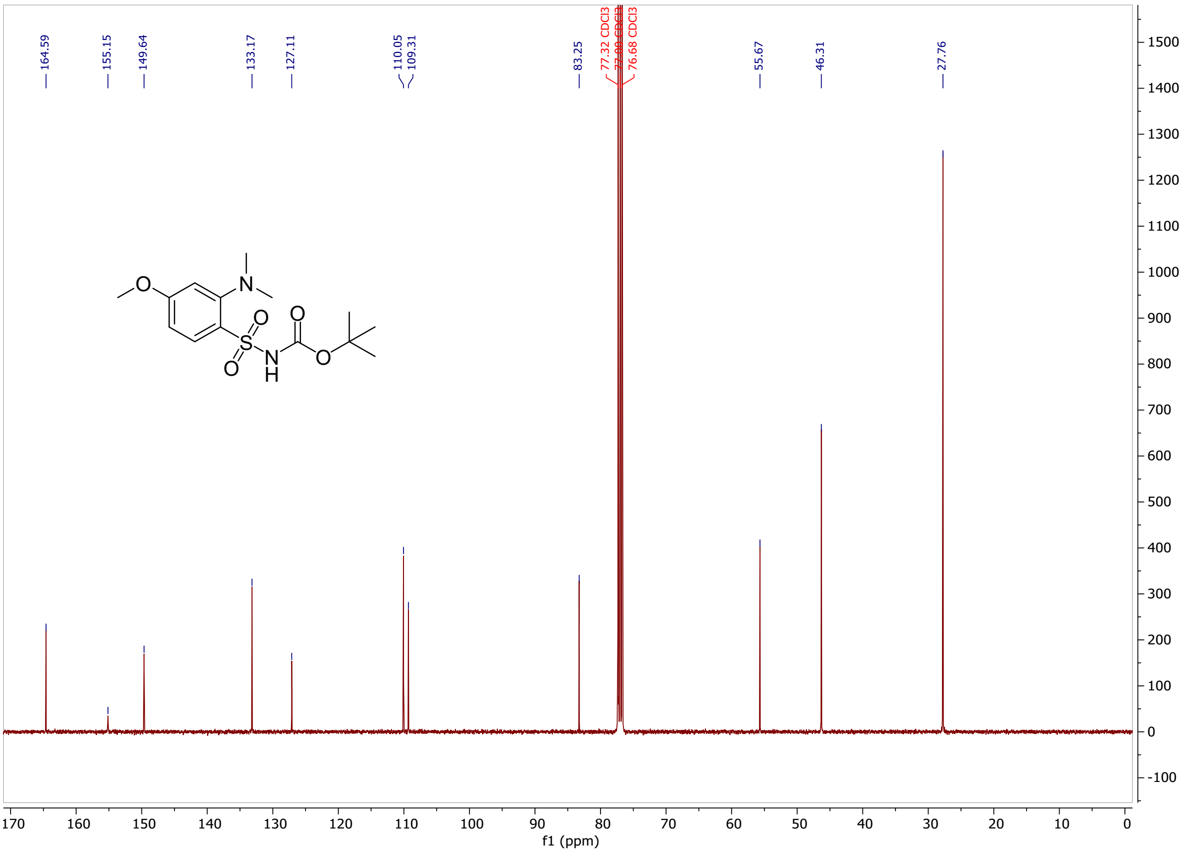

***tert*-Butyl ((4-(dimethylamino)-2-(trifluoromethoxy)phenyl)sulfonyl)carbamate (4p)**

^{1}H-NMR (400 MHz, $CDCl_3$)

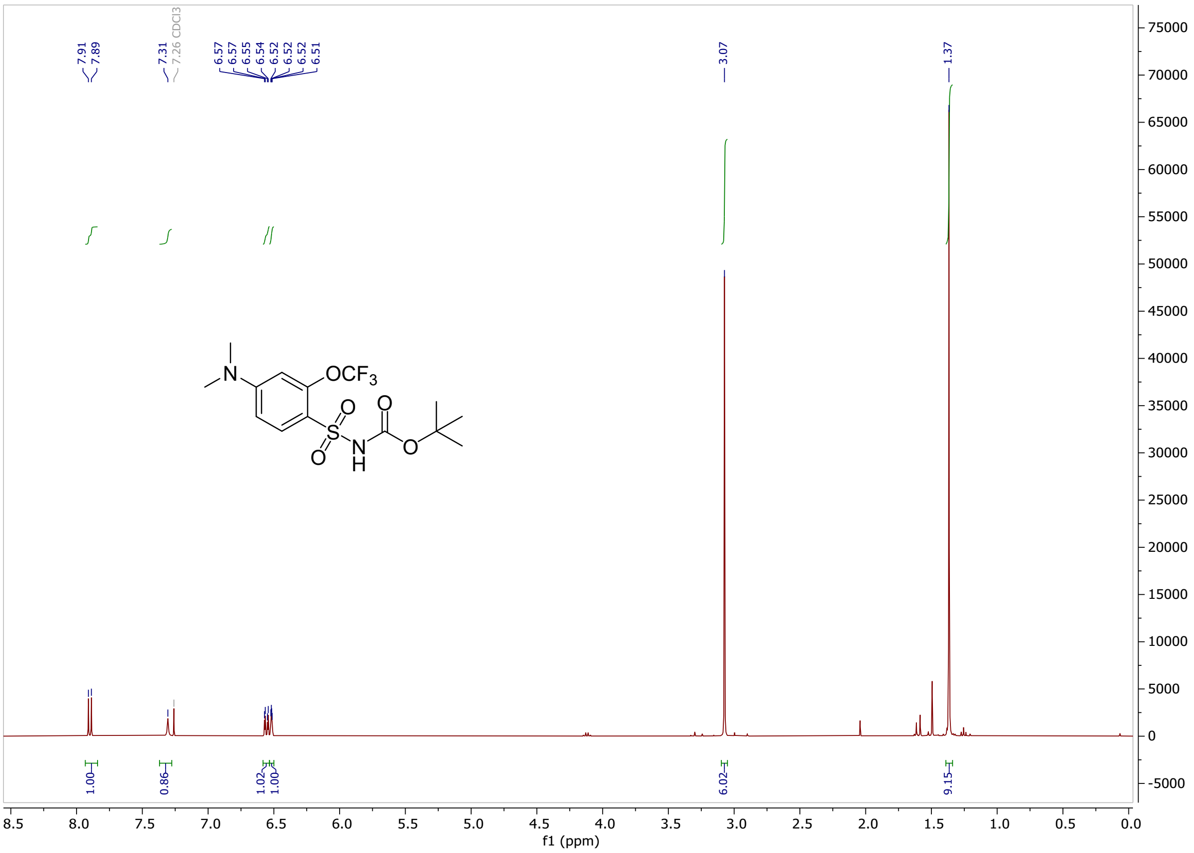

^{13}C-NMR (101 MHz, CDCl$_3$)

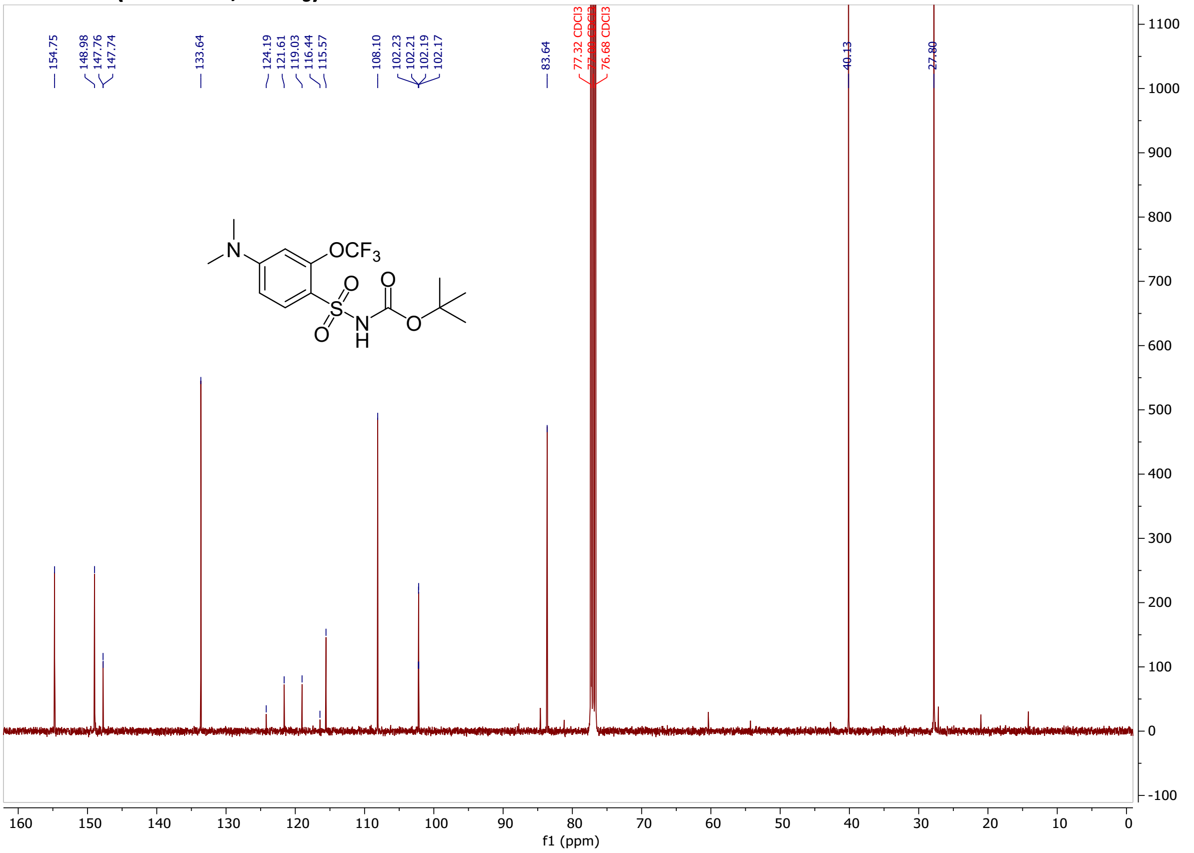

^{19}F-NMR (376 MHz, $CDCl_3$)

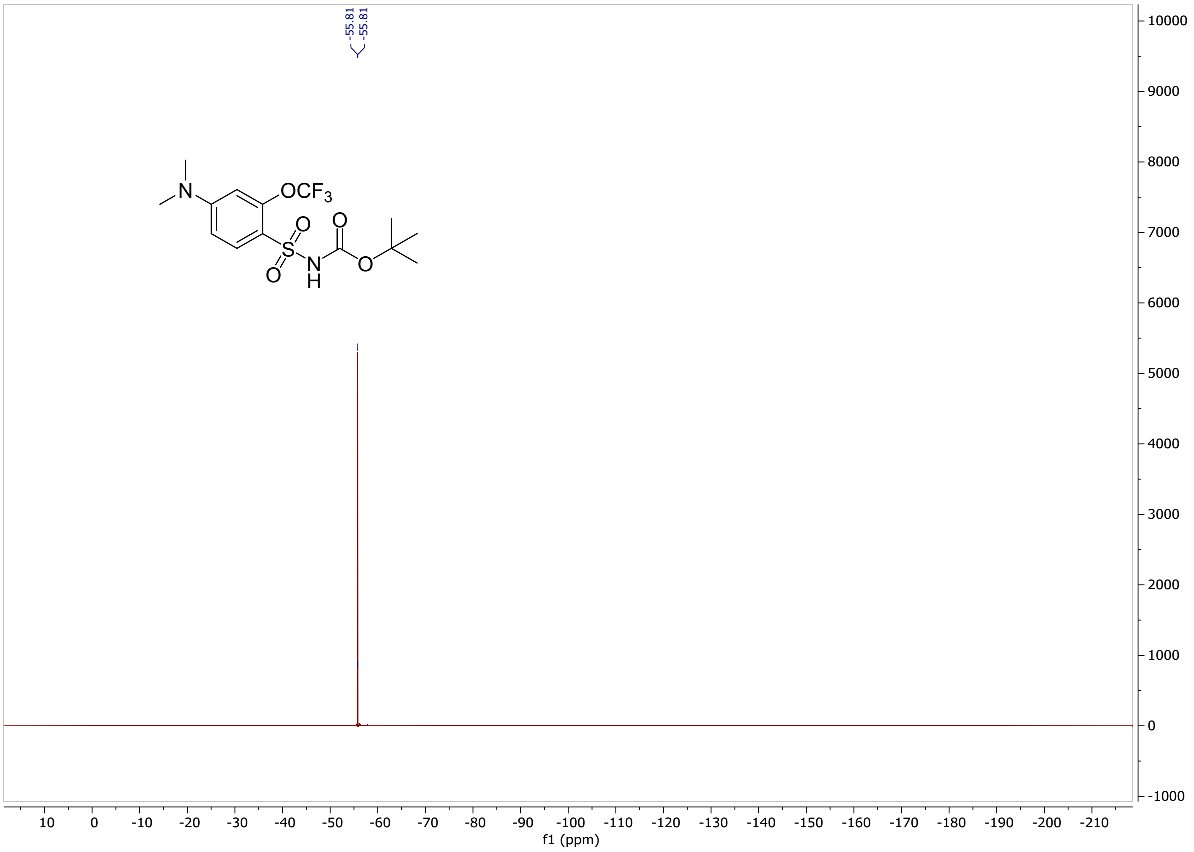

***tert*-Butyl ((4-(dimethylamino)-2-ethynylphenyl)sulfonyl)carbamate (4q)**

^{1}H-NMR (400 MHz, $CDCl_3$)

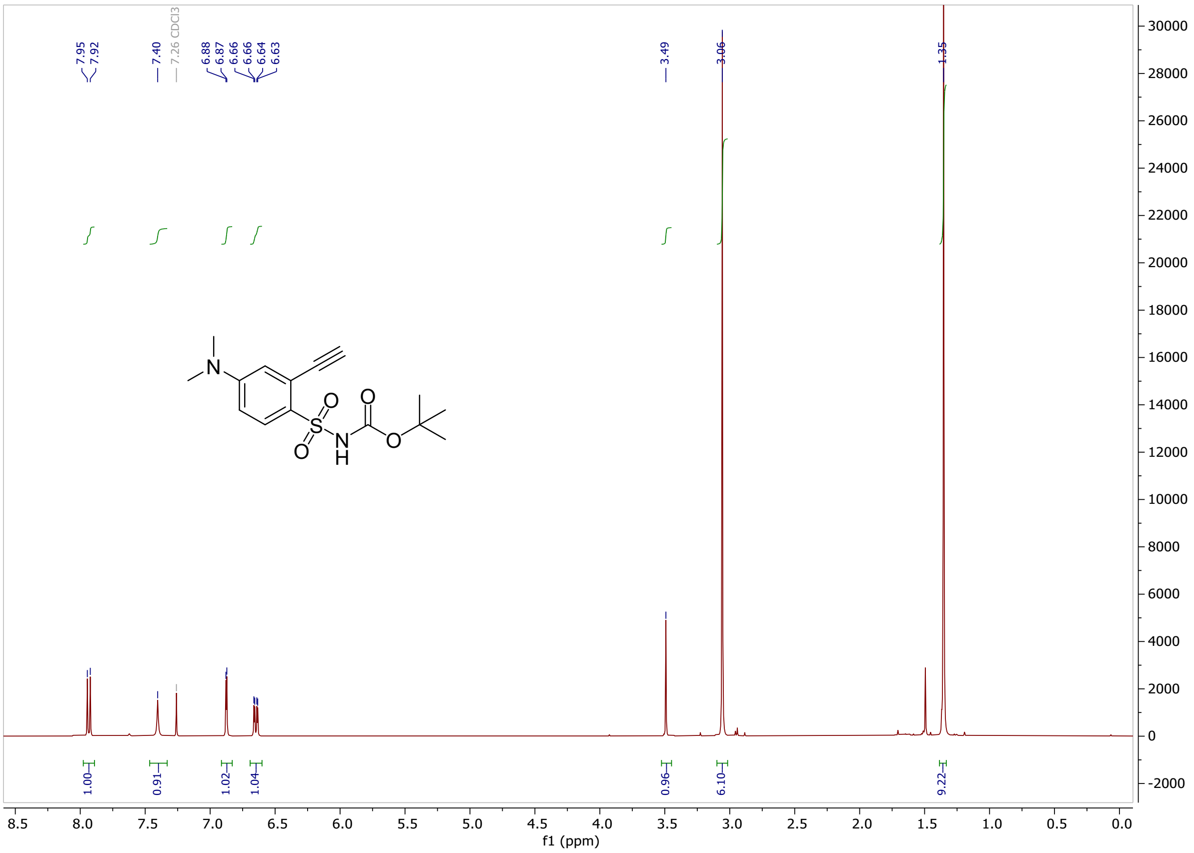

^{13}C-NMR (101 MHz, $CDCl_3$)

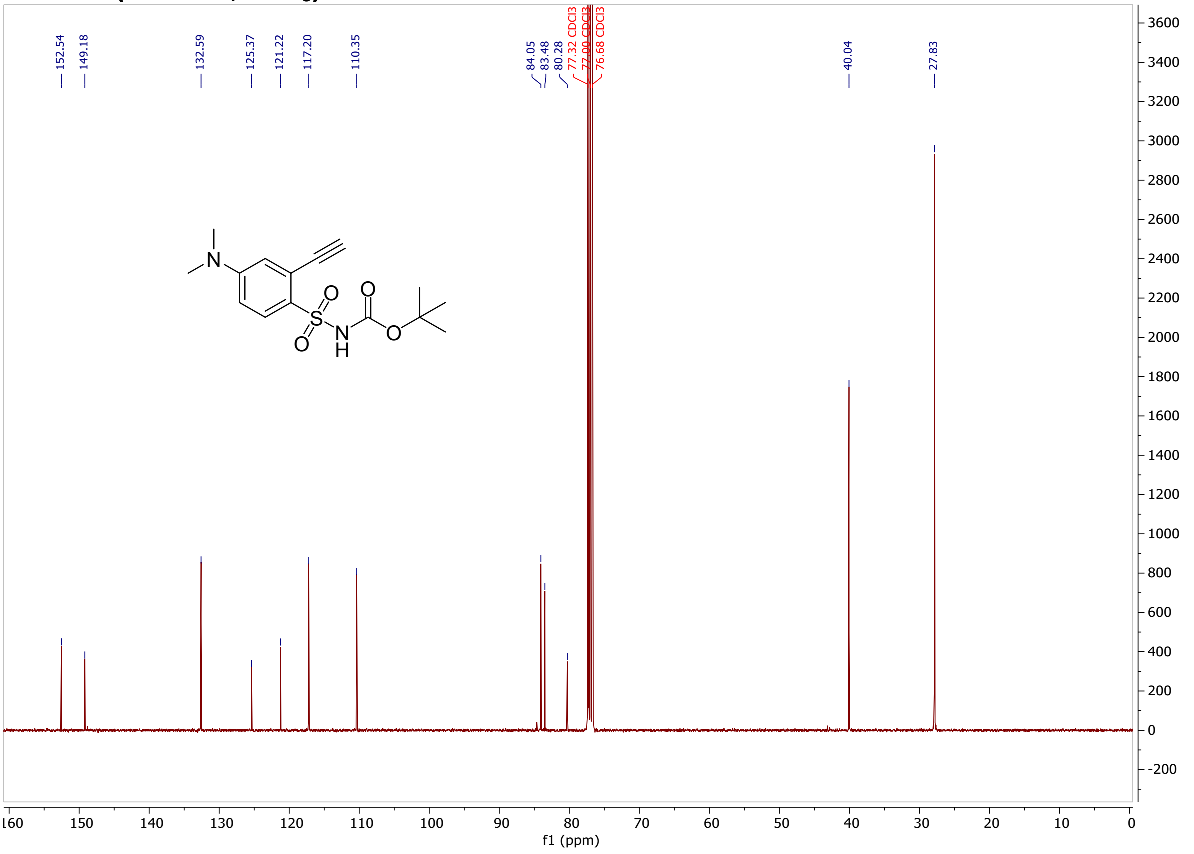

***tert*-Butyl ((4-(dimethylamino)-2-(trifluoromethyl)phenyl)sulfonyl)carbamate (4r)**

^{1}H-NMR (400 MHz, $CDCl_3$)

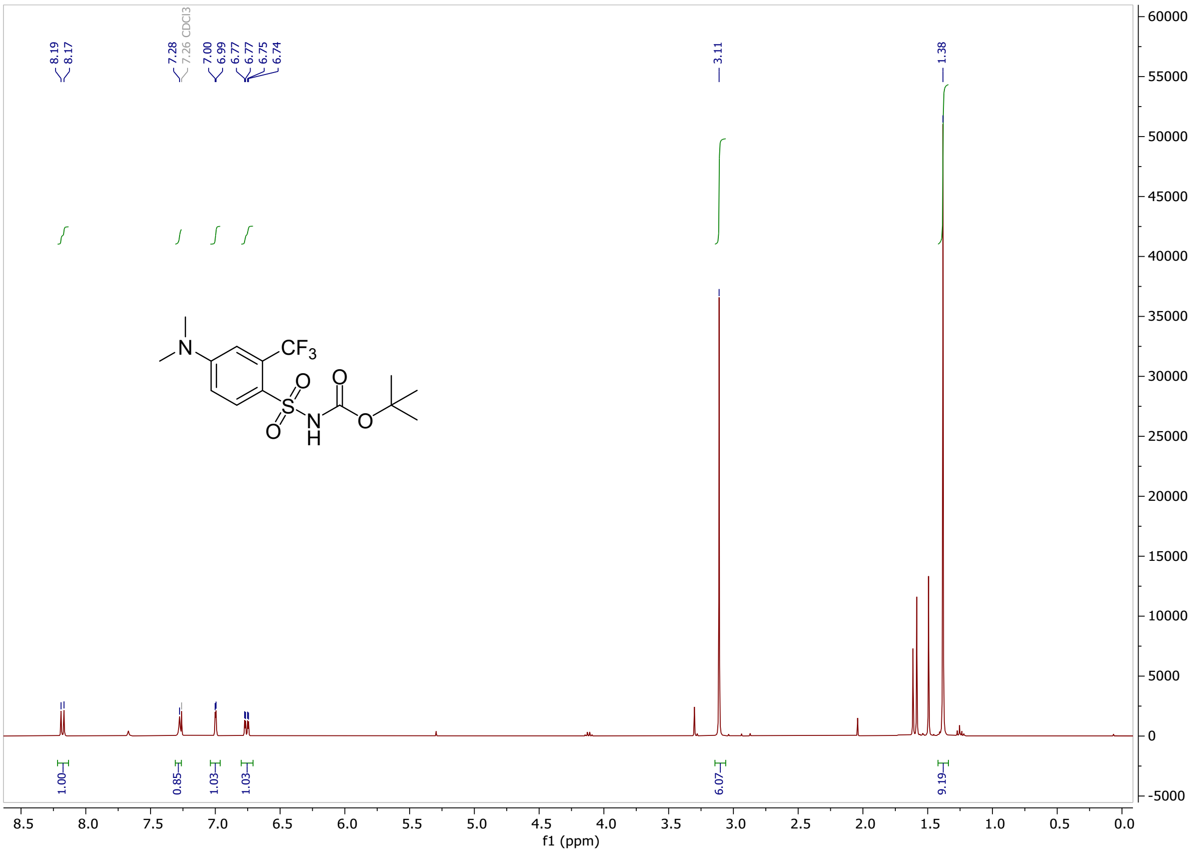

^{13}C-NMR (101 MHz, $CDCl_3$)

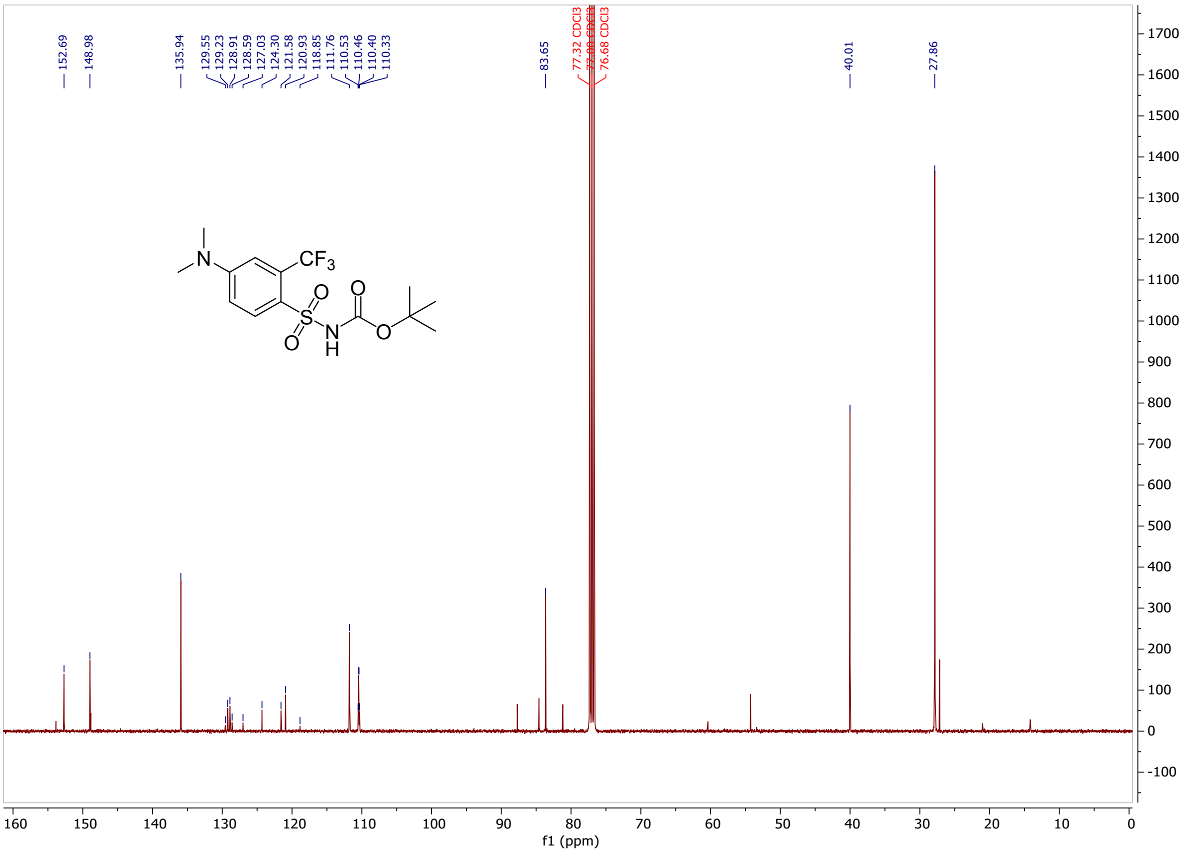

^{19}F-NMR (376 MHz, $CDCl_3$)

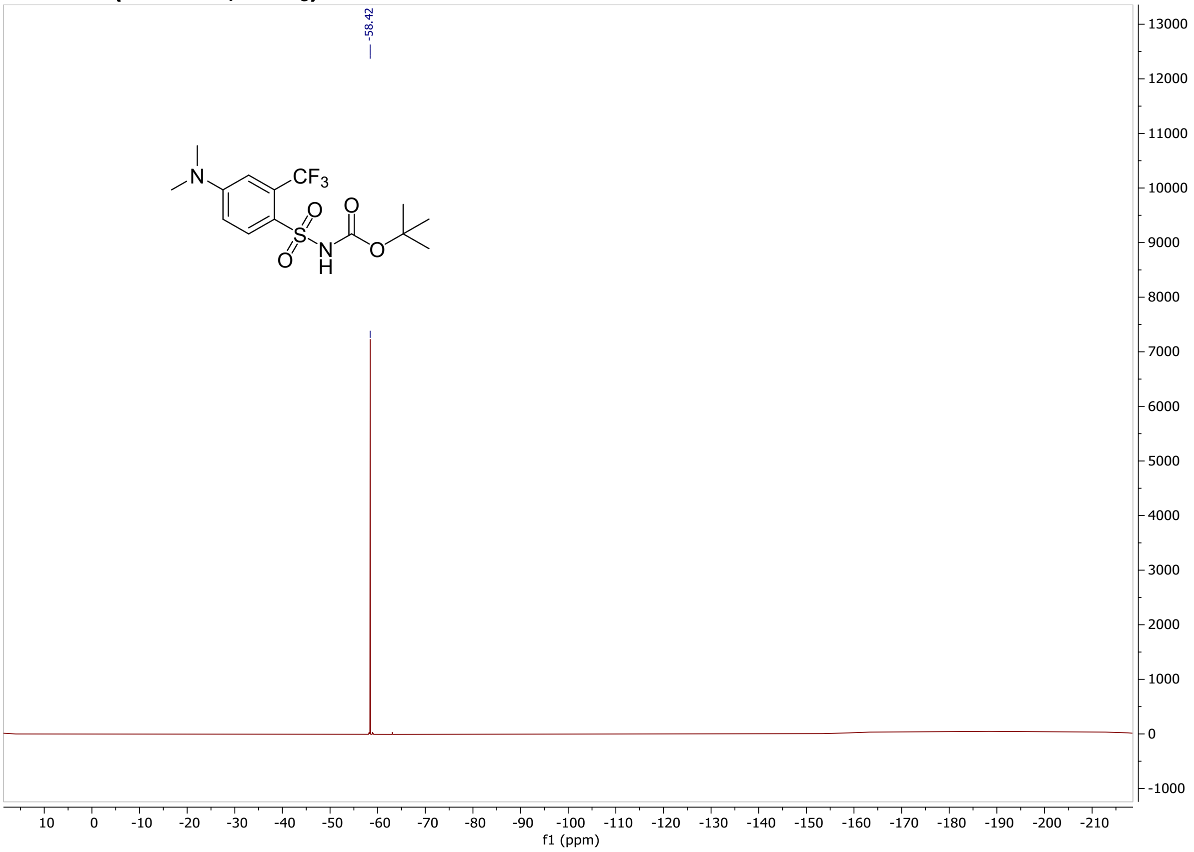

***tert*-Butyl ((4-(dimethylamino)-2-nitrophenyl)sulfonyl)carbamate (4s)**

^{1}H-NMR (400 MHz, $CDCl_3$)

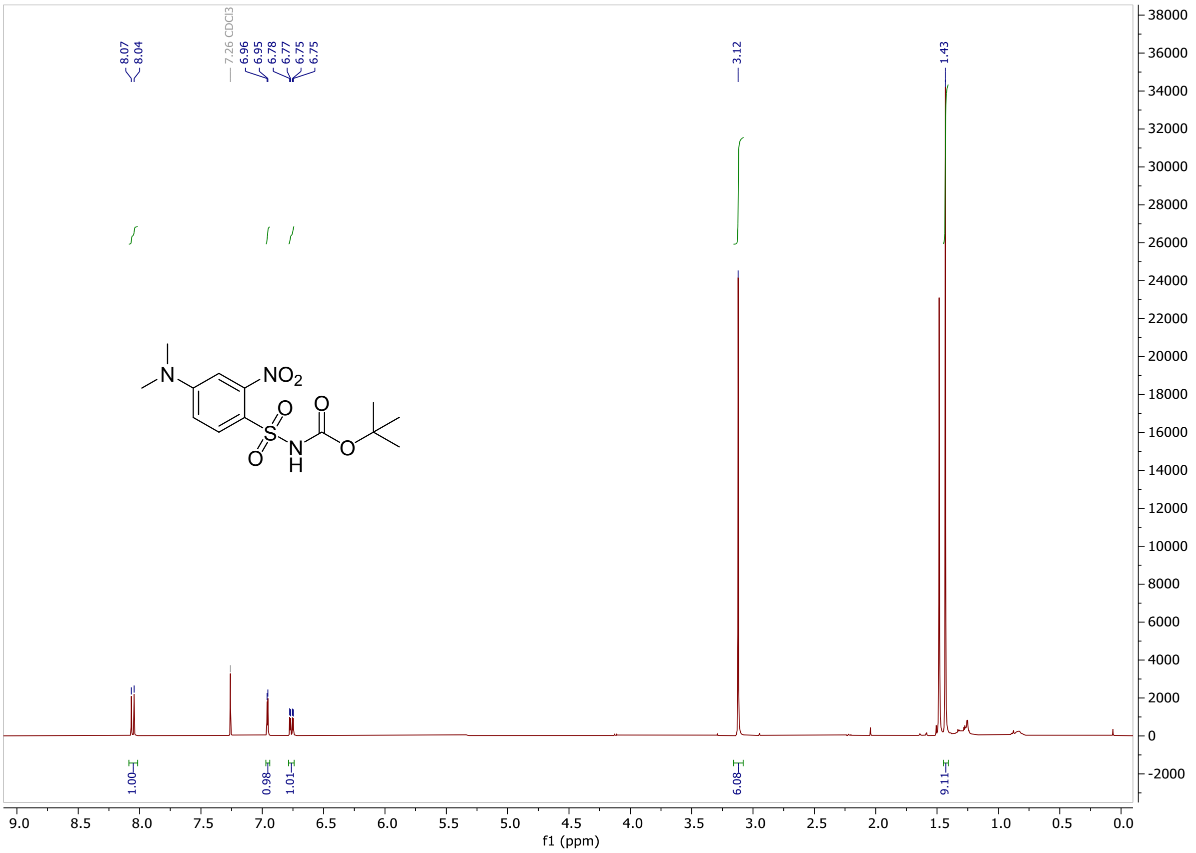

^{13}C-NMR (101 MHz, $CDCl_3$)

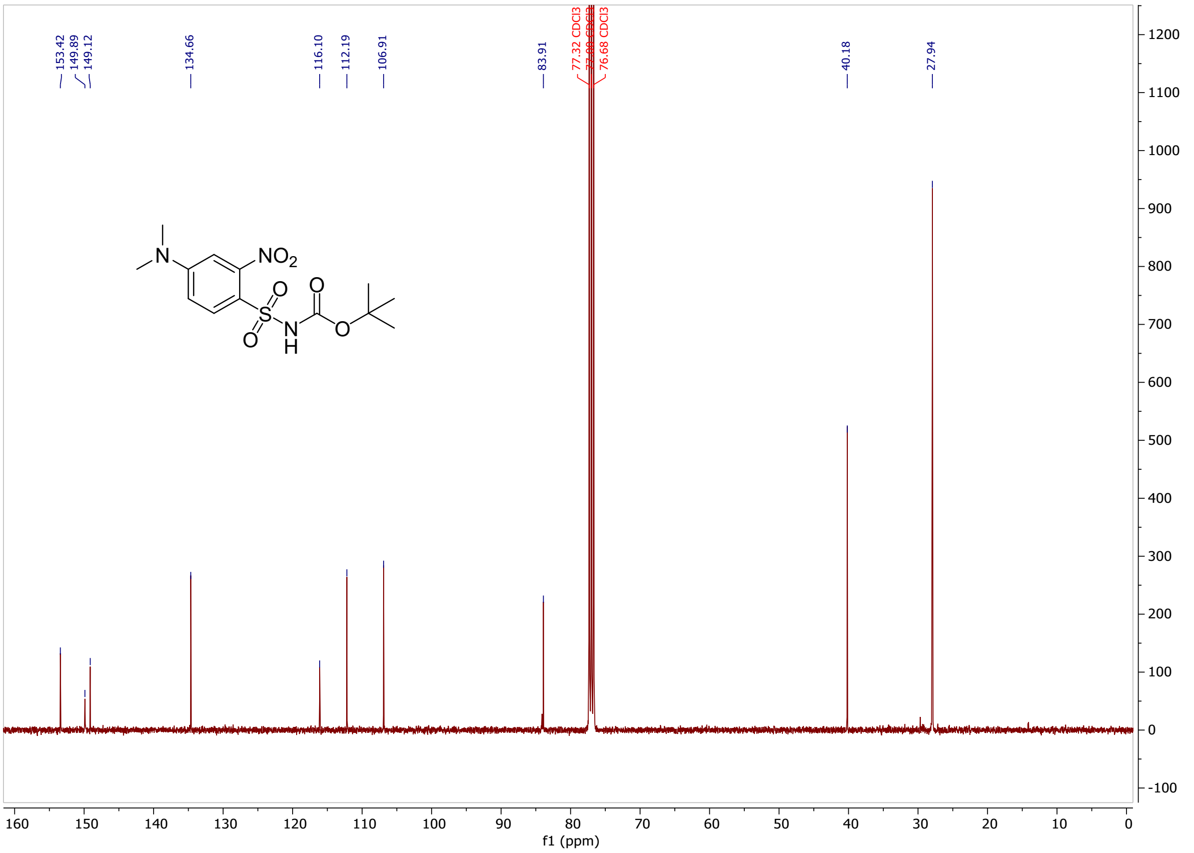

tert-Butyl ((4-(dimethylamino)-2-fluorophenyl)sulfonyl)carbamate (4t)

^{1}H-NMR (400 MHz, $CDCl_3$)

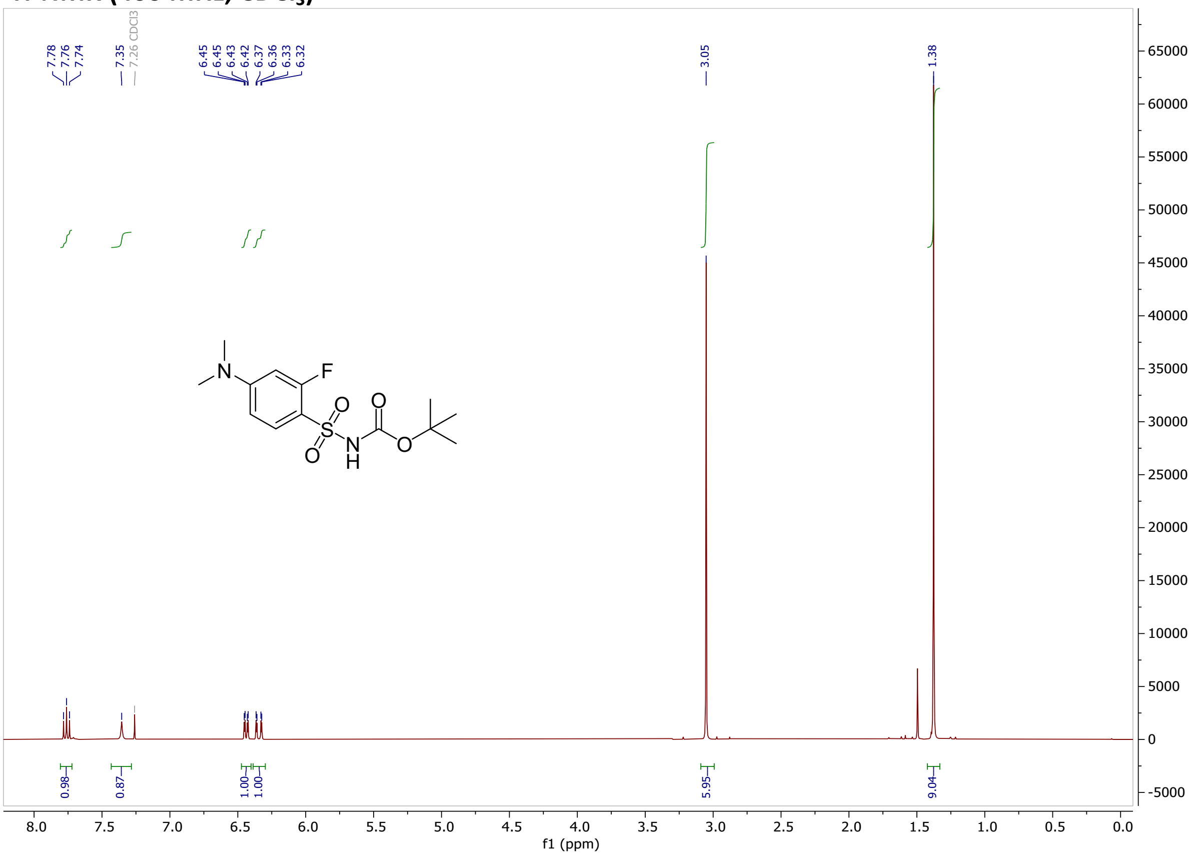

^{13}C-NMR (101 MHz, $CDCl_3$)

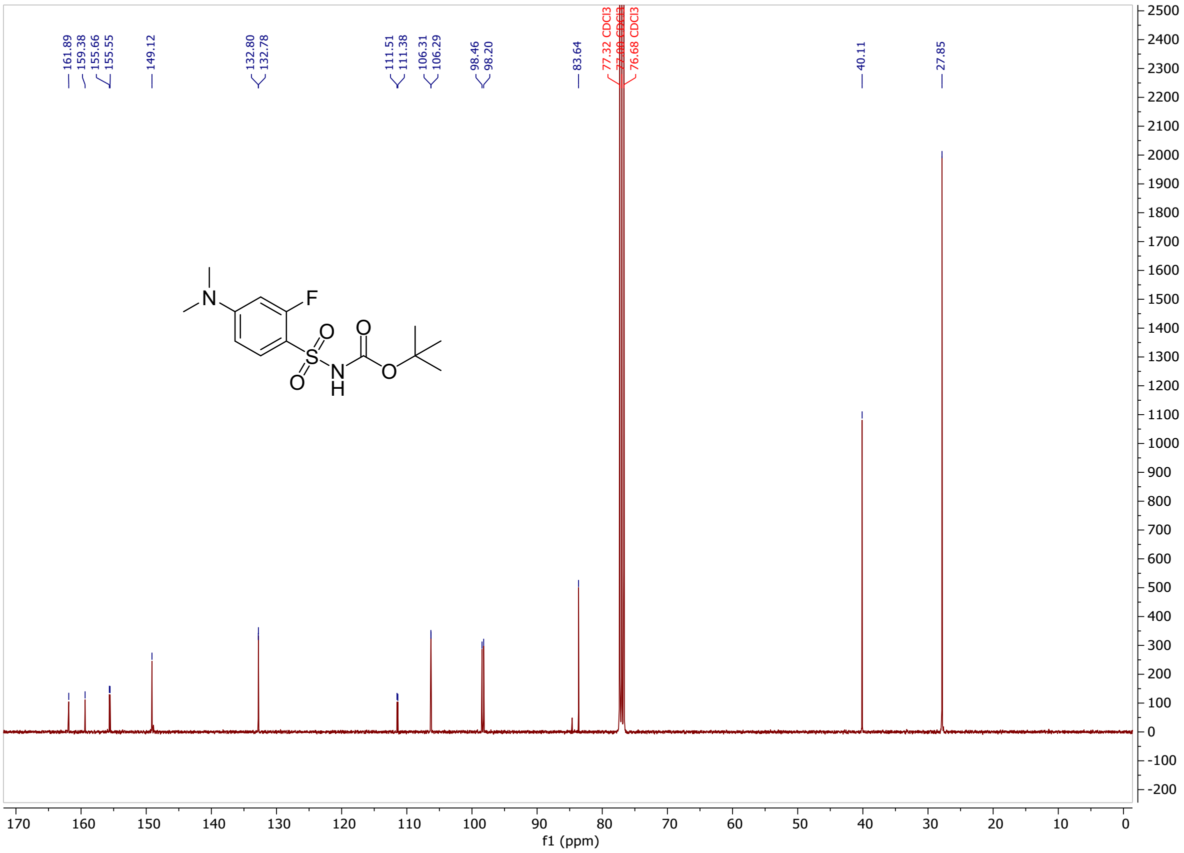

^{19}F-NMR (376 MHz, $CDCl_3$)

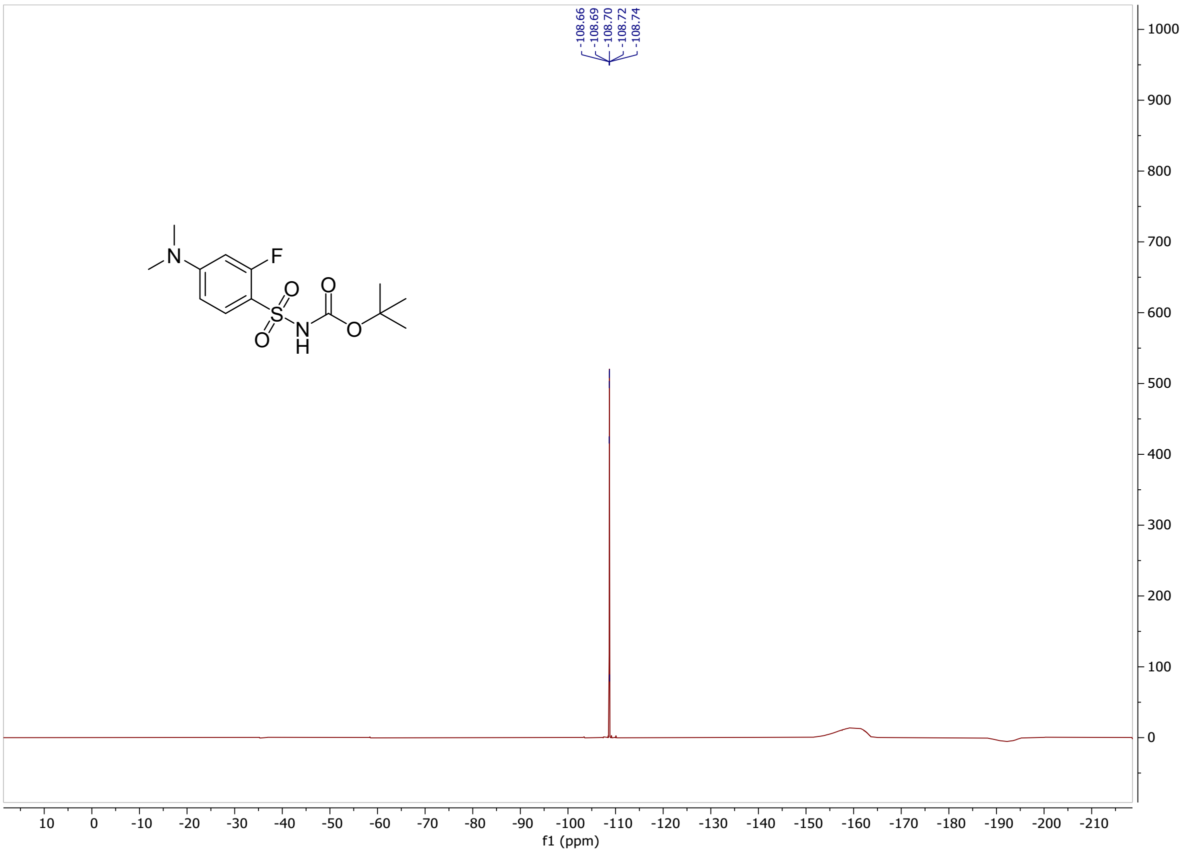

***tert*-Butyl ((4-(dimethylamino)-2-chlorophenyl)sulfonyl)carbamate (4u)**

^{1}H-NMR (400 MHz, $CDCl_3$)

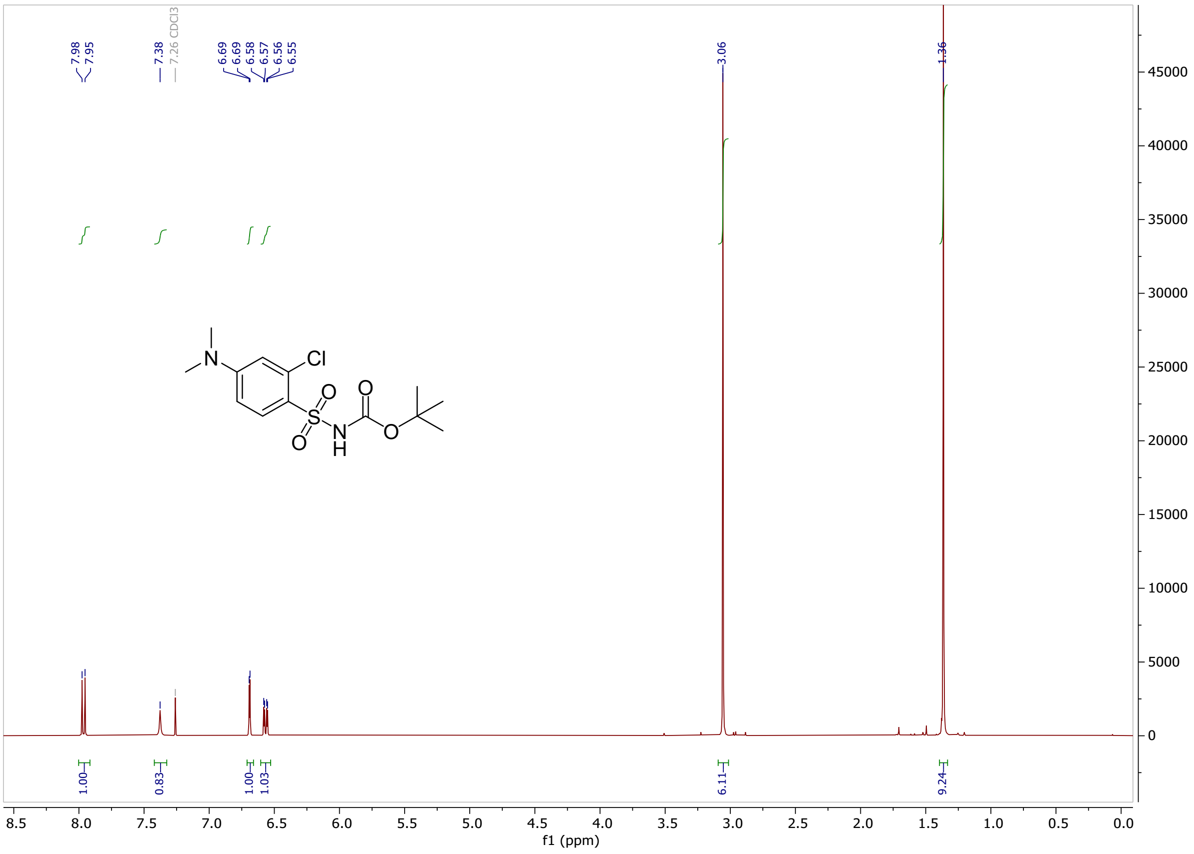

^{13}C-NMR (101 MHz, $CDCl_3$)

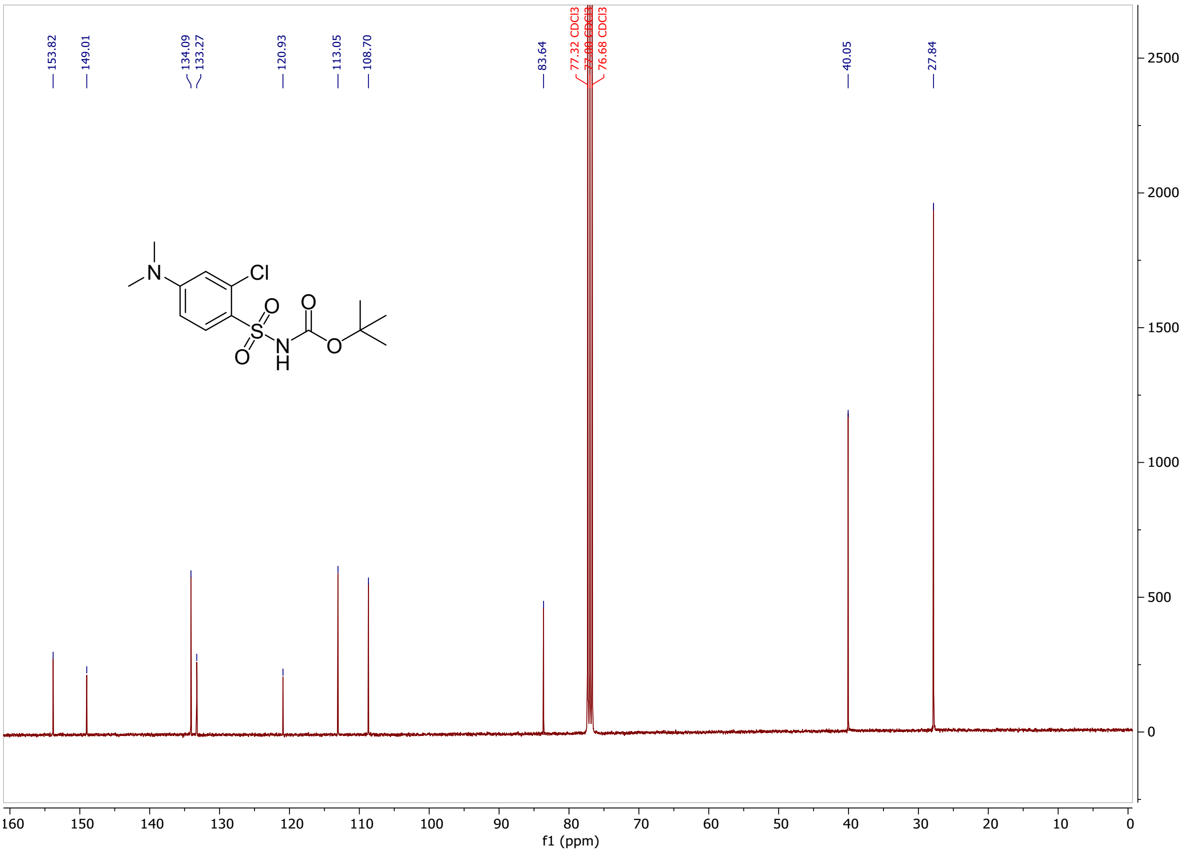

tert-Butyl ((2-bromo-4-(dimethylamino)phenyl)sulfonyl)carbamate (4v)
^{1}H-NMR (400 MHz, $CDCl_3$)

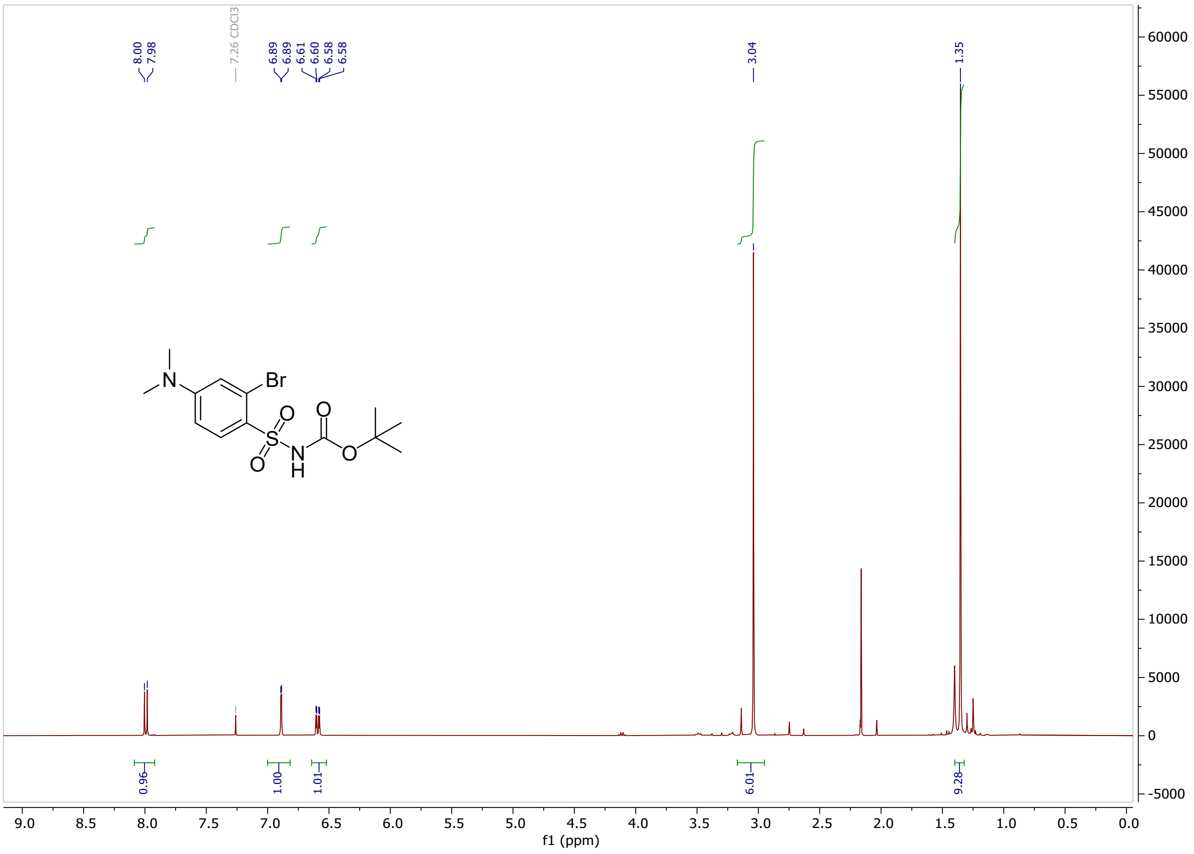

^{13}C-NMR (101 MHz, $CDCl_3$)

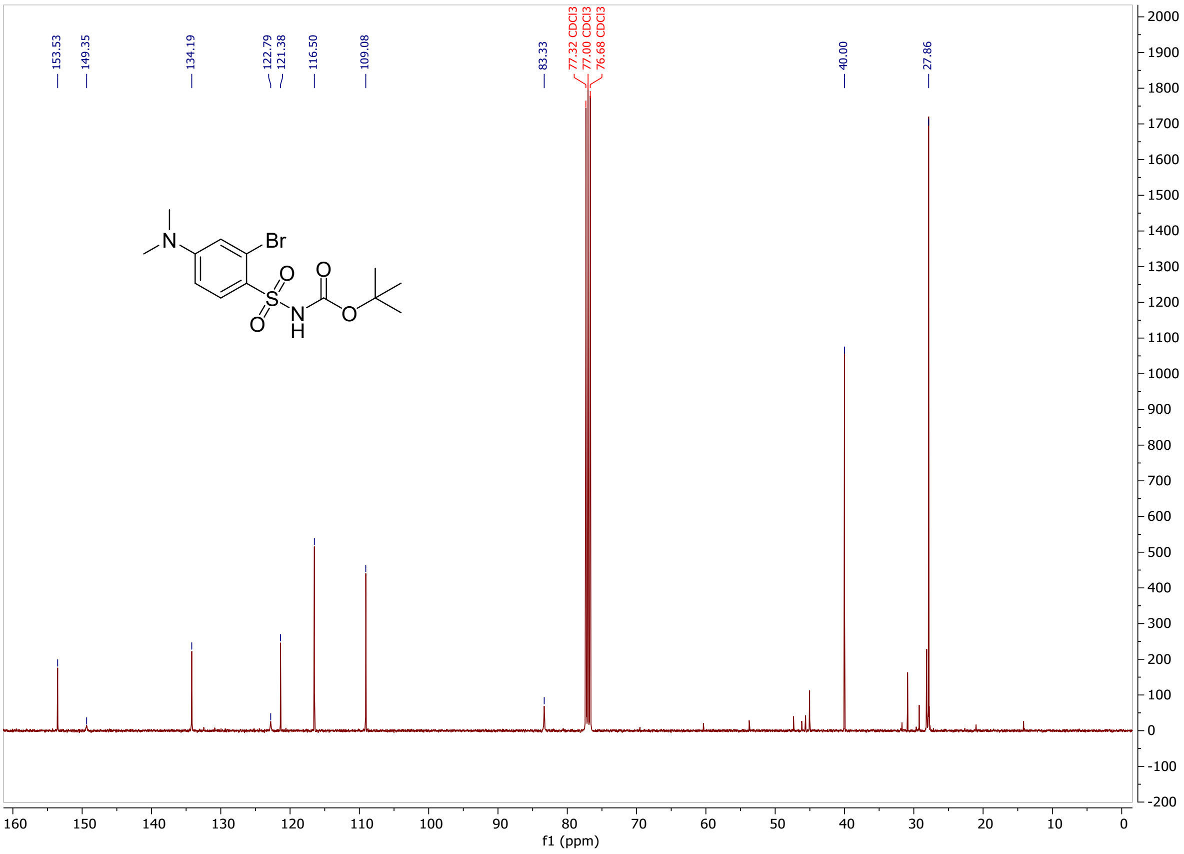

***tert*-Butyl ((4-(dimethylamino)-2-iodophenyl)sulfonyl)carbamate (4w)**

^{1}H-NMR (400 MHz, $CDCl_3$)

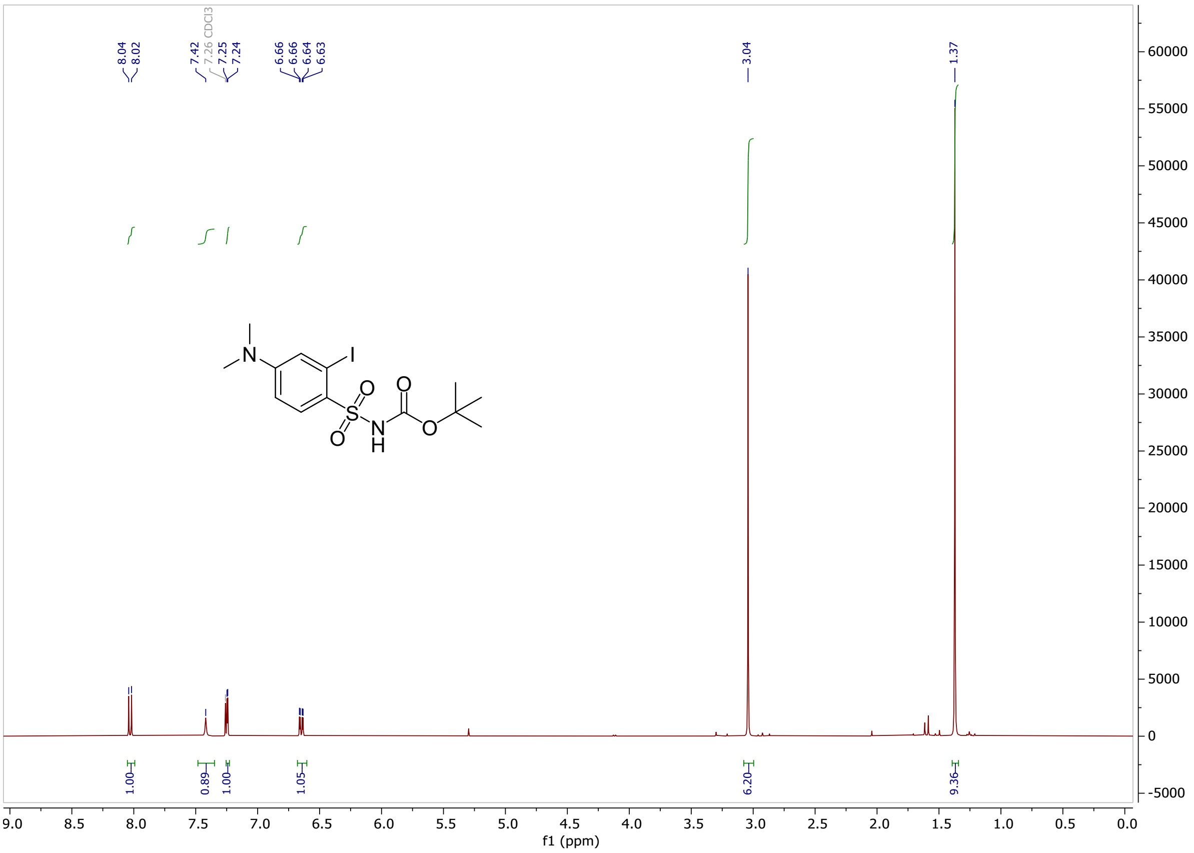

^{13}C-NMR (101 MHz, $CDCl_3$)

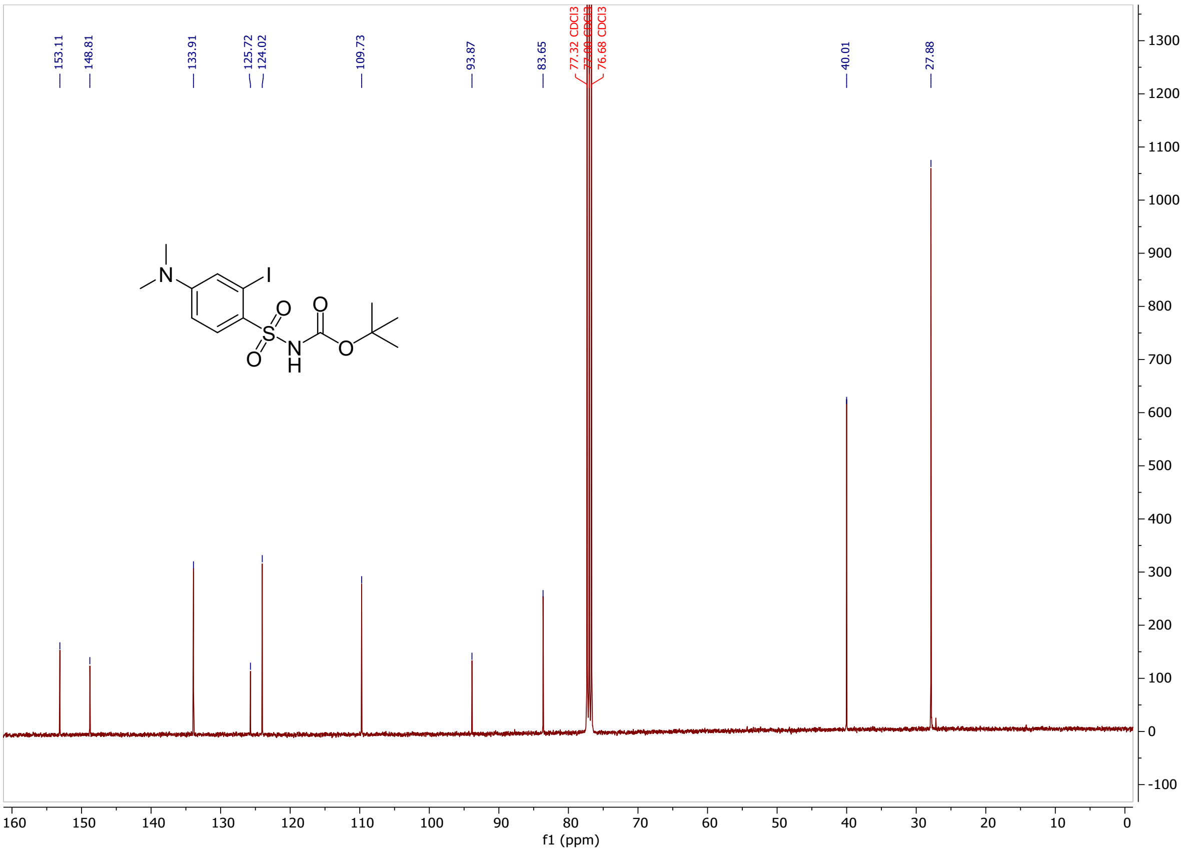

***tert*-Butyl ((2-bromo-4-(dimethylamino)-5-fluorophenyl)sulfonyl)carbamate (4x)**

^{1}H-NMR (400 MHz, $CDCl_3$)

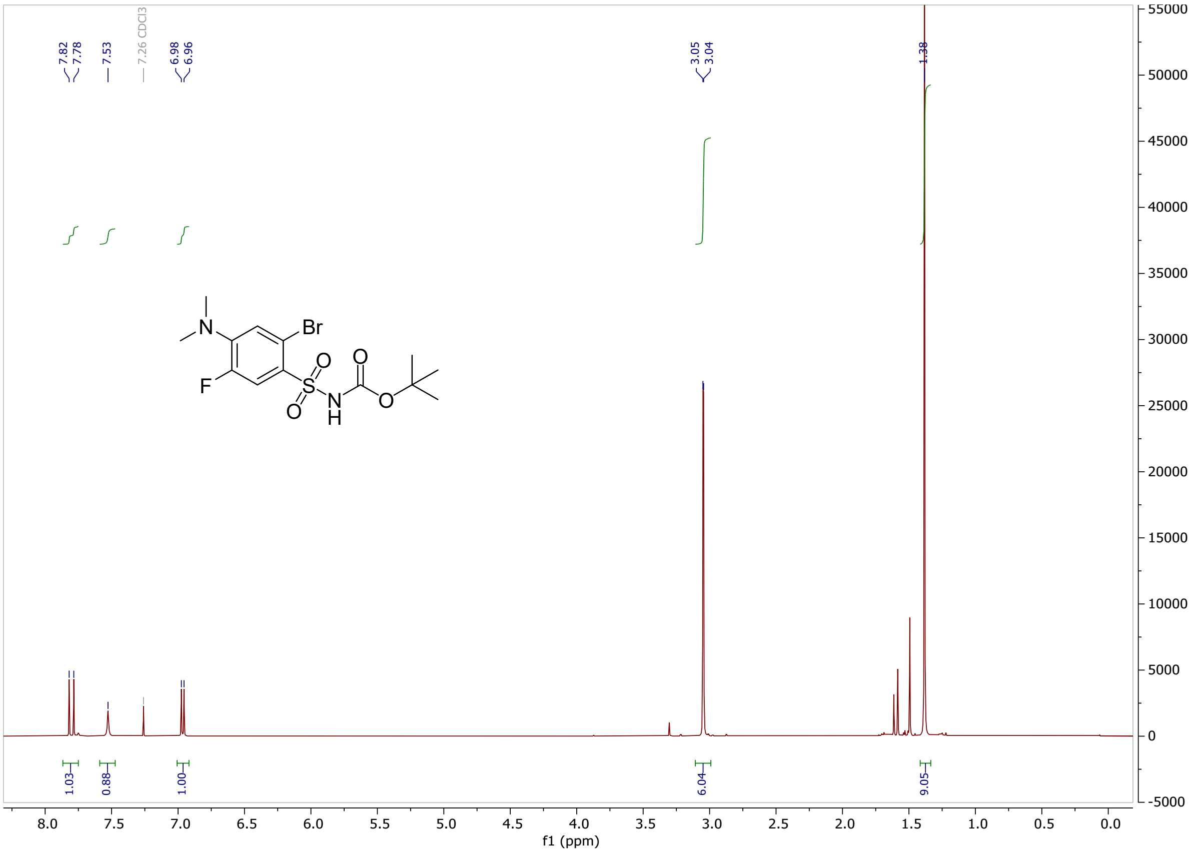

^{13}C-NMR (101 MHz, $CDCl_3$)

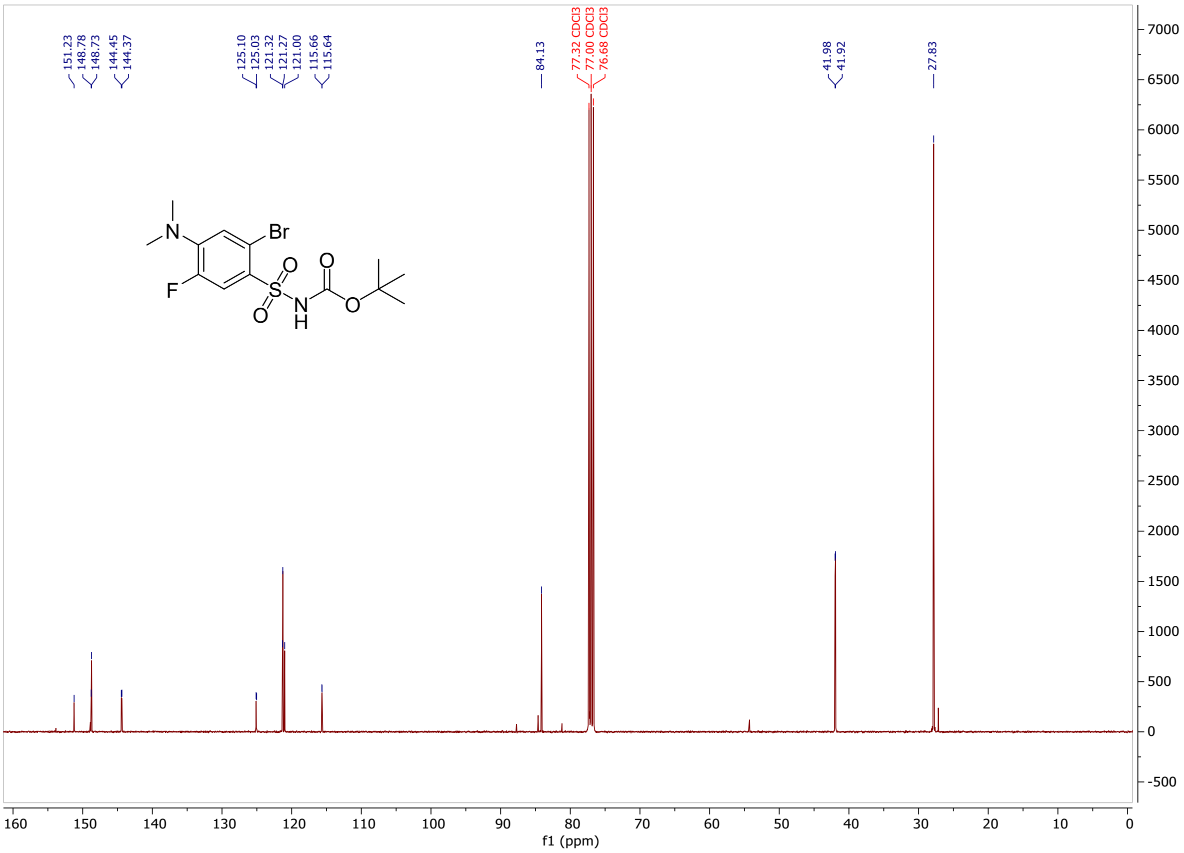

^{19}F-NMR (376 MHz, $CDCl_3$)

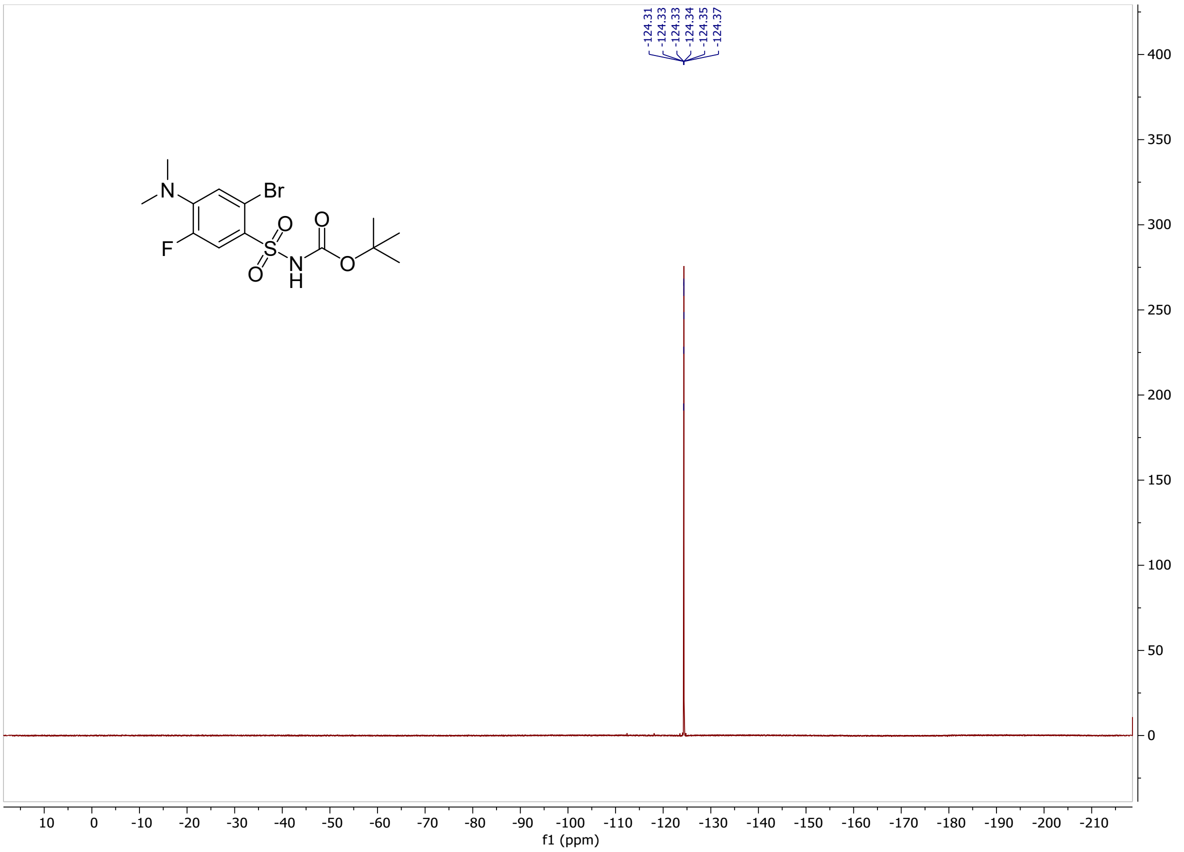

***tert*-Butyl ((1-methyl-1*H*-indol-3-yl)sulfonyl)carbamate (5a)**

^{1}H-NMR (400 MHz, CDCl$_3$)

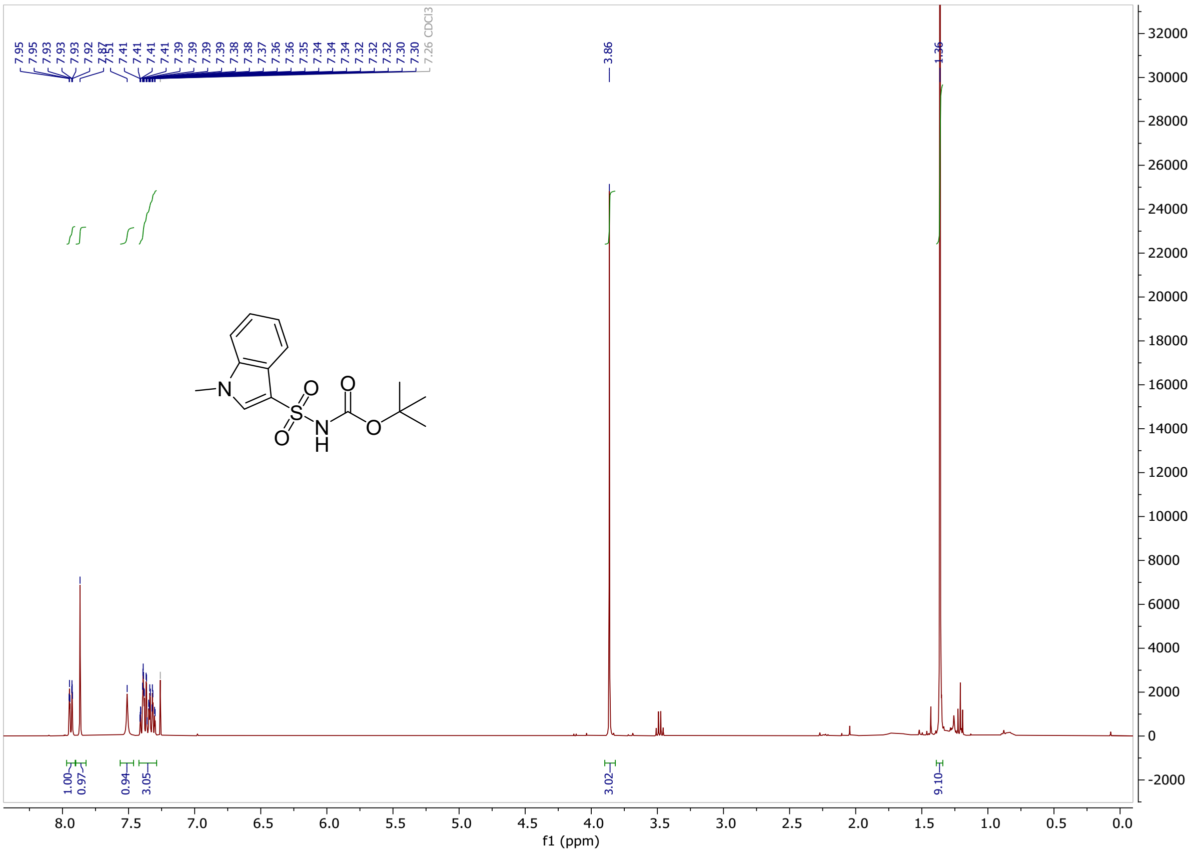

^{13}C-NMR (101 MHz, CDCl$_3$)

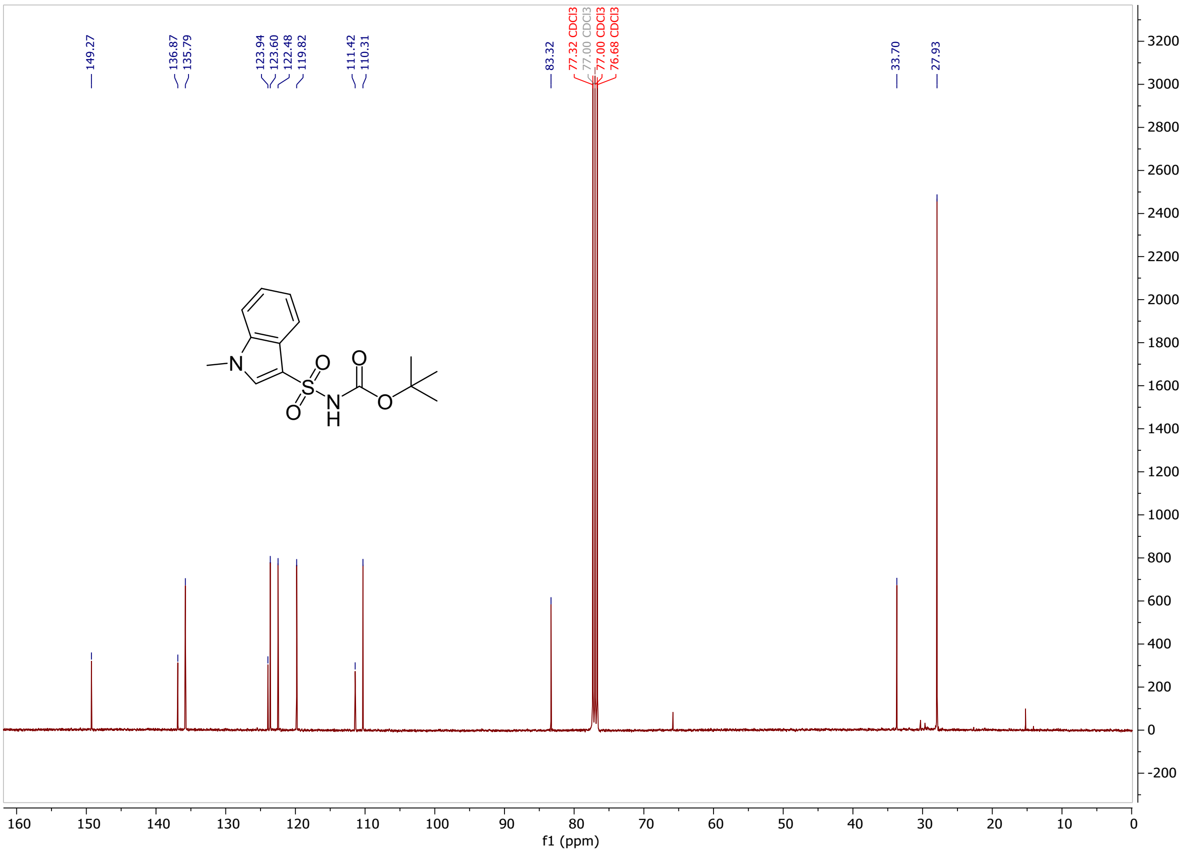

***tert*-Butyl ((1-(*tert*-butyldimethylsilyl)-1*H*-indol-3-yl)sulfonyl)carbamate (5b)**

^{1}H-NMR (400 MHz, $CDCl_3$)

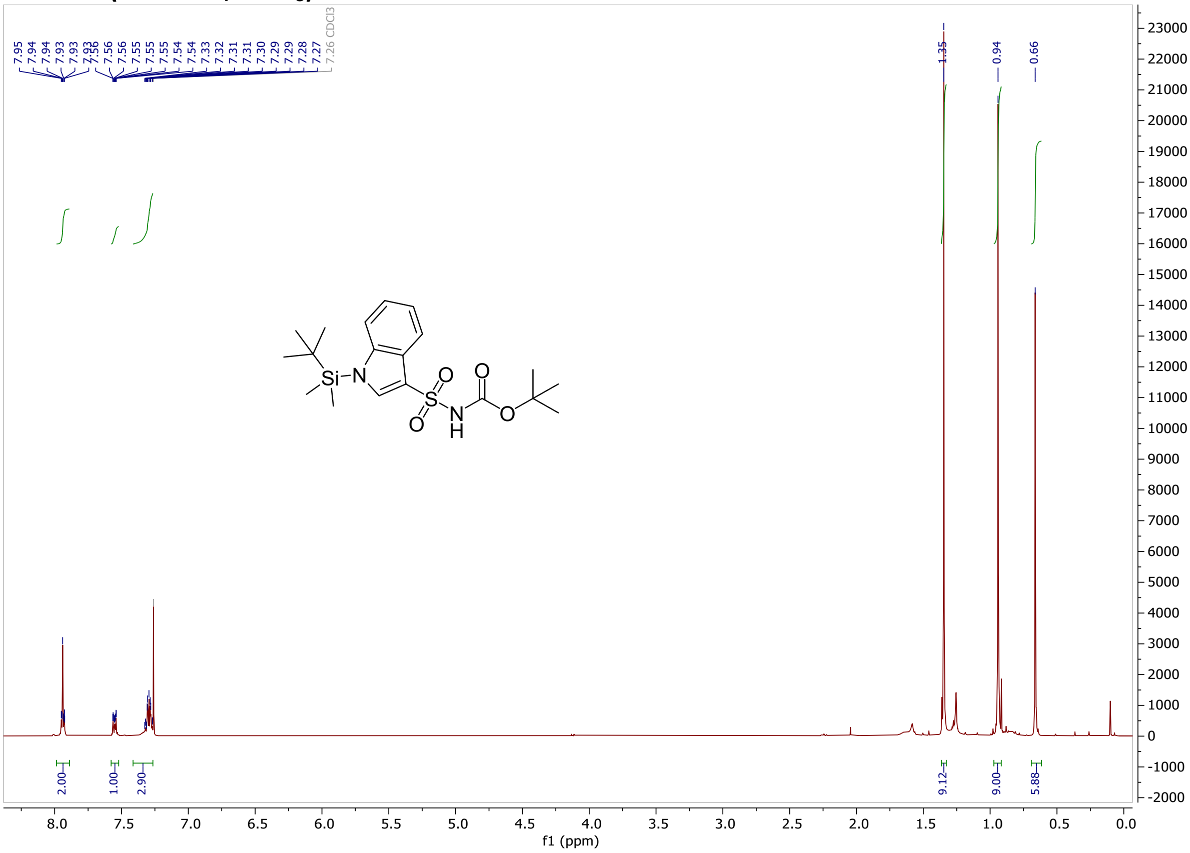

^{13}C-NMR (101 MHz, $CDCl_3$)

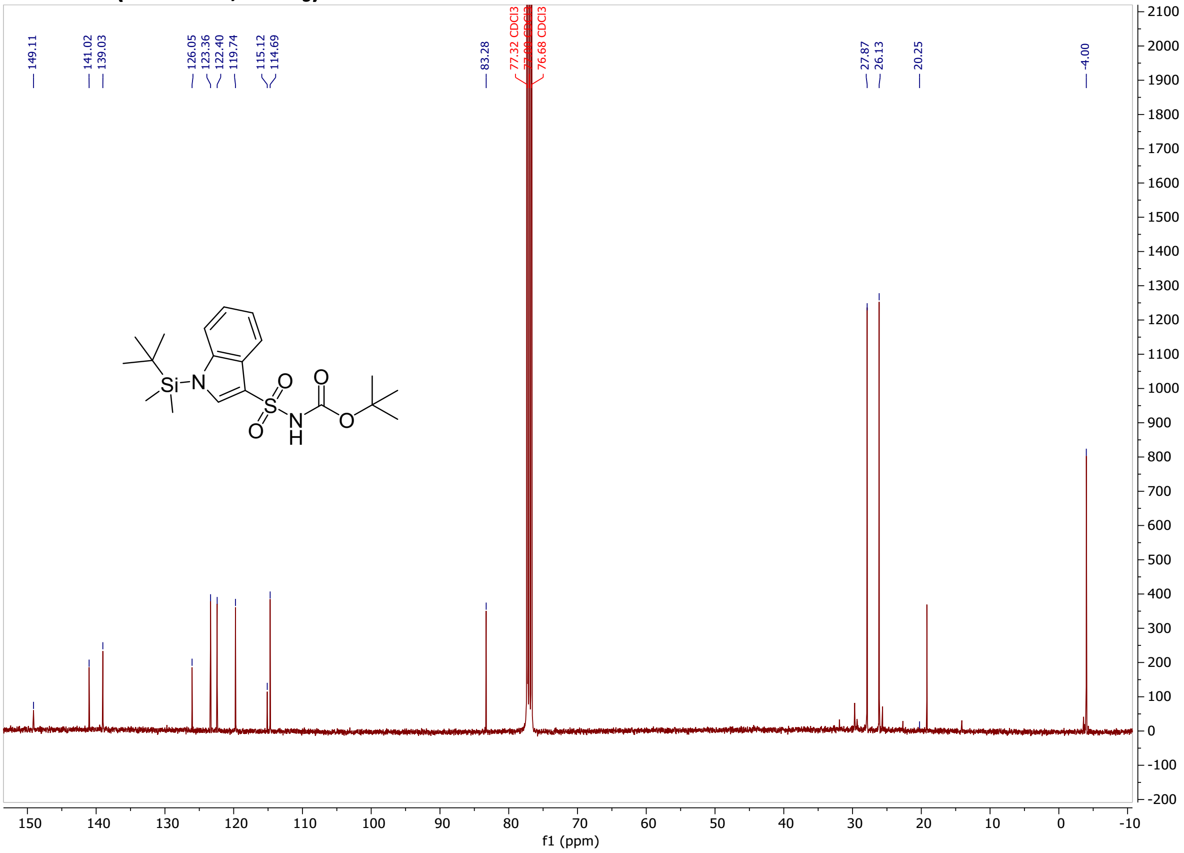

***tert*-Butyl 3-(*N*-(*tert*-butoxycarbonyl)sulfamoyl)-1*H*-indole-1-carboxylate (5c)**

^{1}H-NMR (400 MHz, $CDCl_3$)

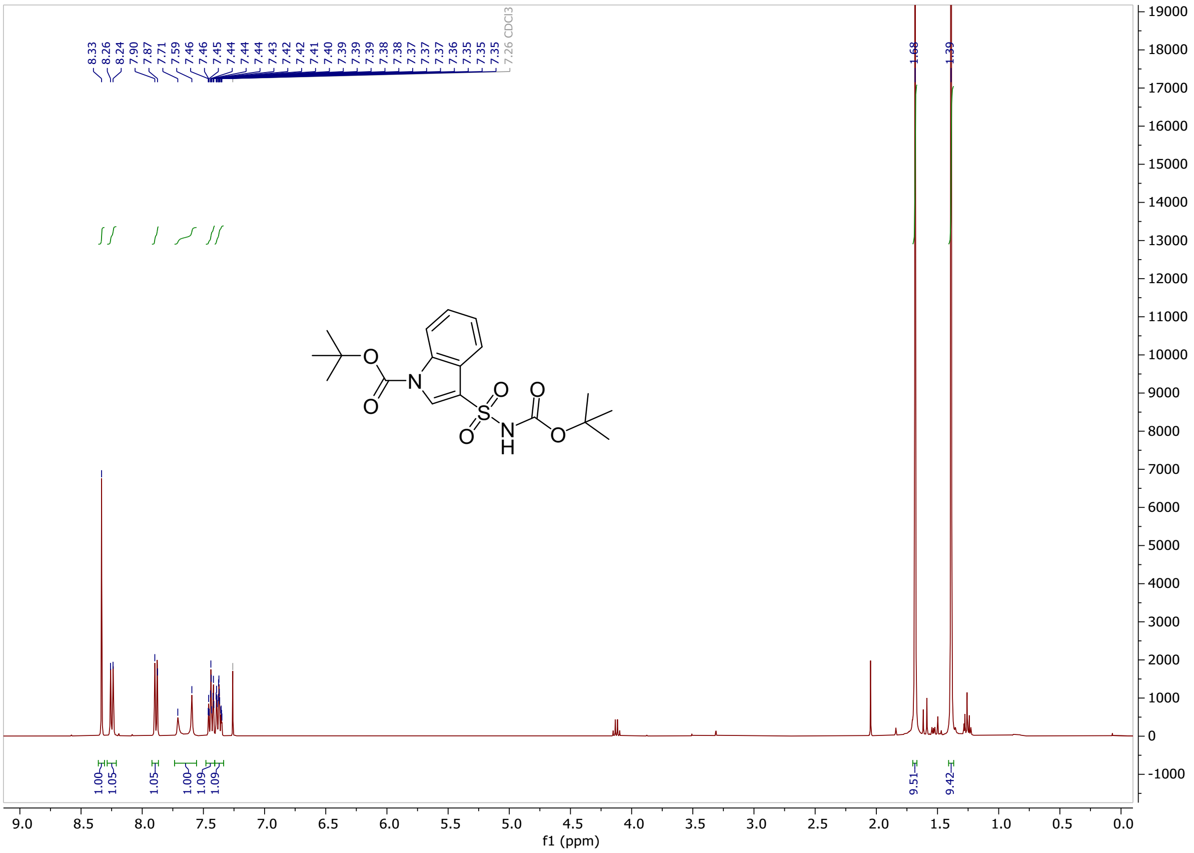

^{13}C-NMR (101 MHz, $CDCl_3$)

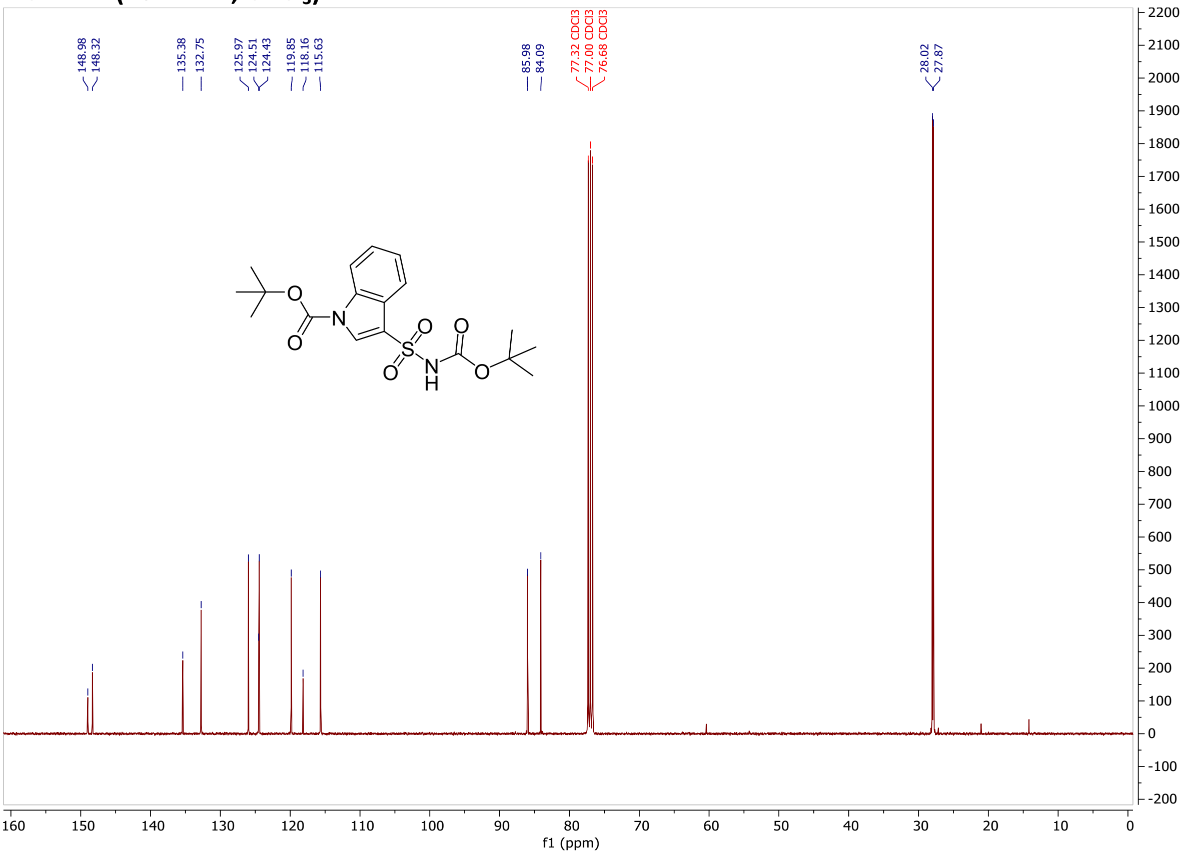

***tert*-Butyl 2-(*N*-(tert-butoxycarbonyl)sulfamoyl)-1*H*-pyrrole-1-carboxylate (5d)**

^{1}H-NMR (400 MHz, $CDCl_3$)

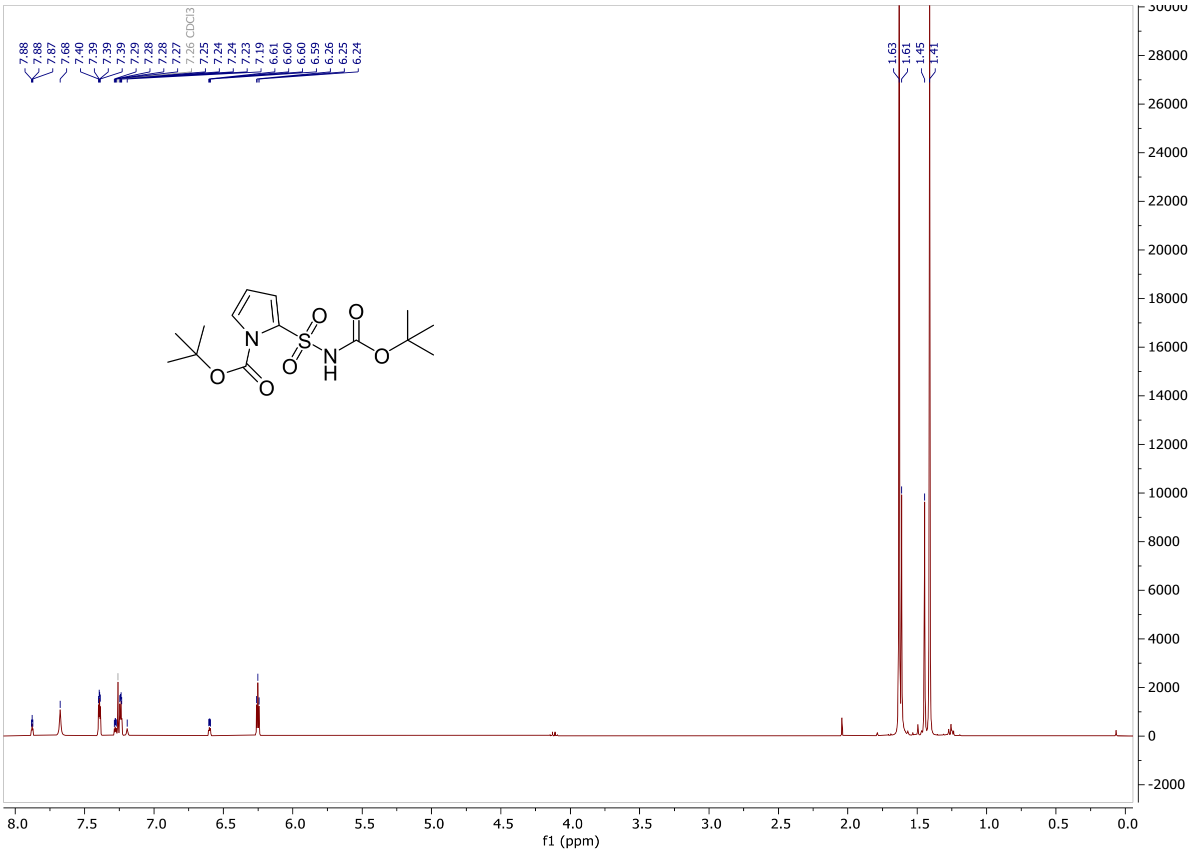

^{13}C-NMR (101 MHz, $CDCl_3$)

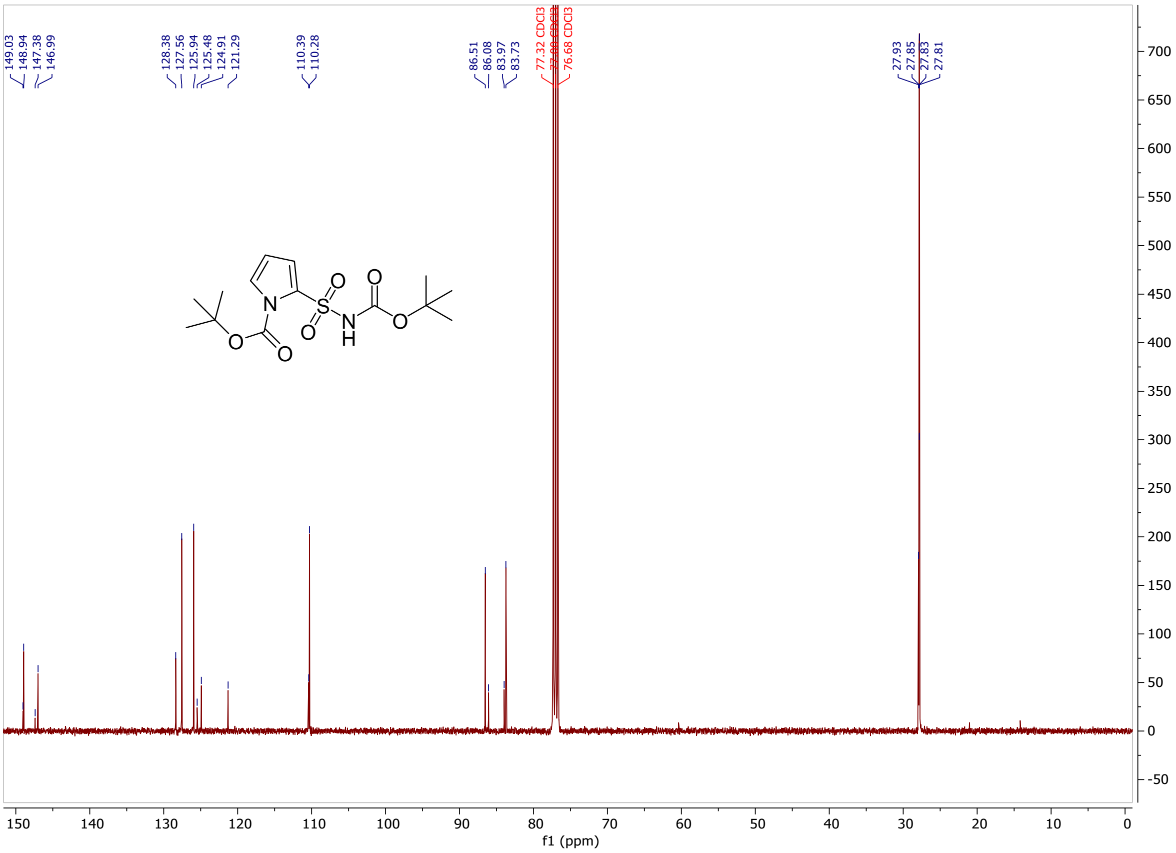

***tert*-Butyl ((5-methylfuran-2-yl)sulfonyl)carbamate (5e)**

^{1}H-NMR (400 MHz, $CDCl_3$)

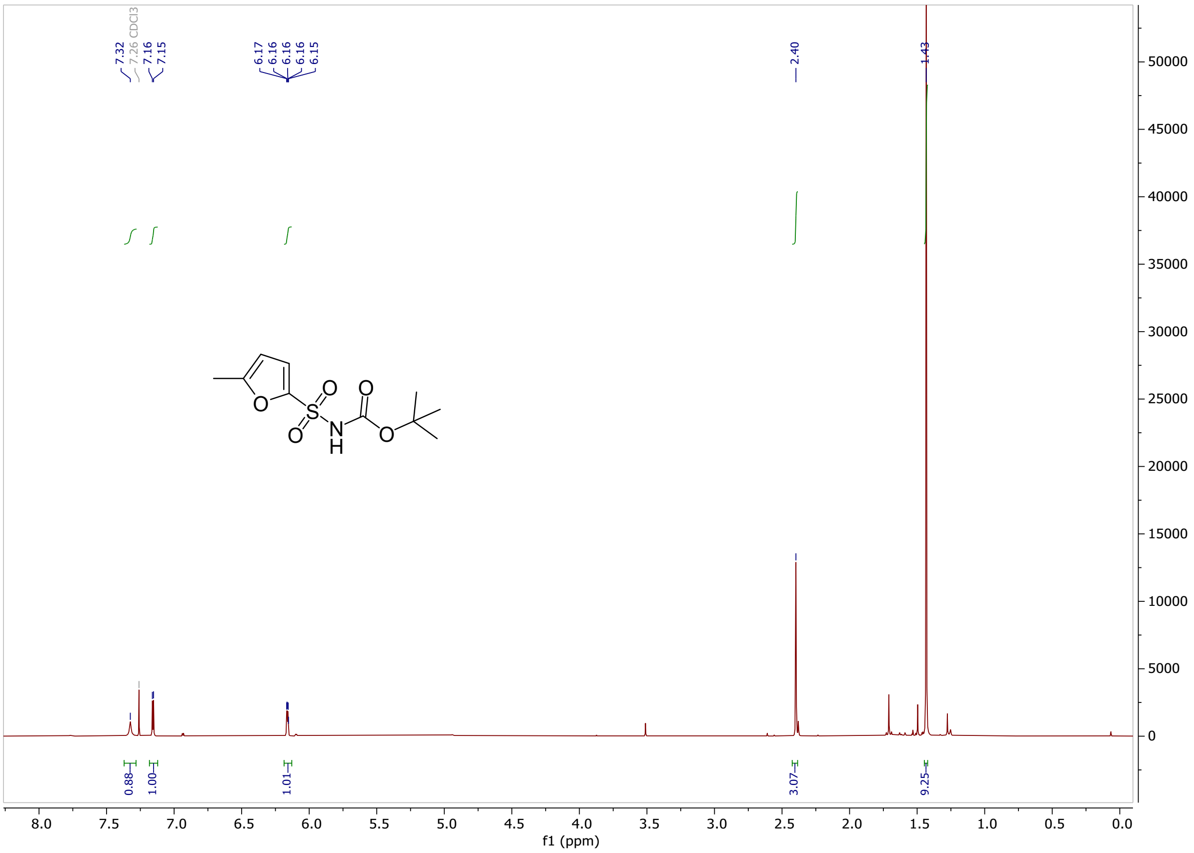

^{13}C-NMR (101 MHz, $CDCl_3$)

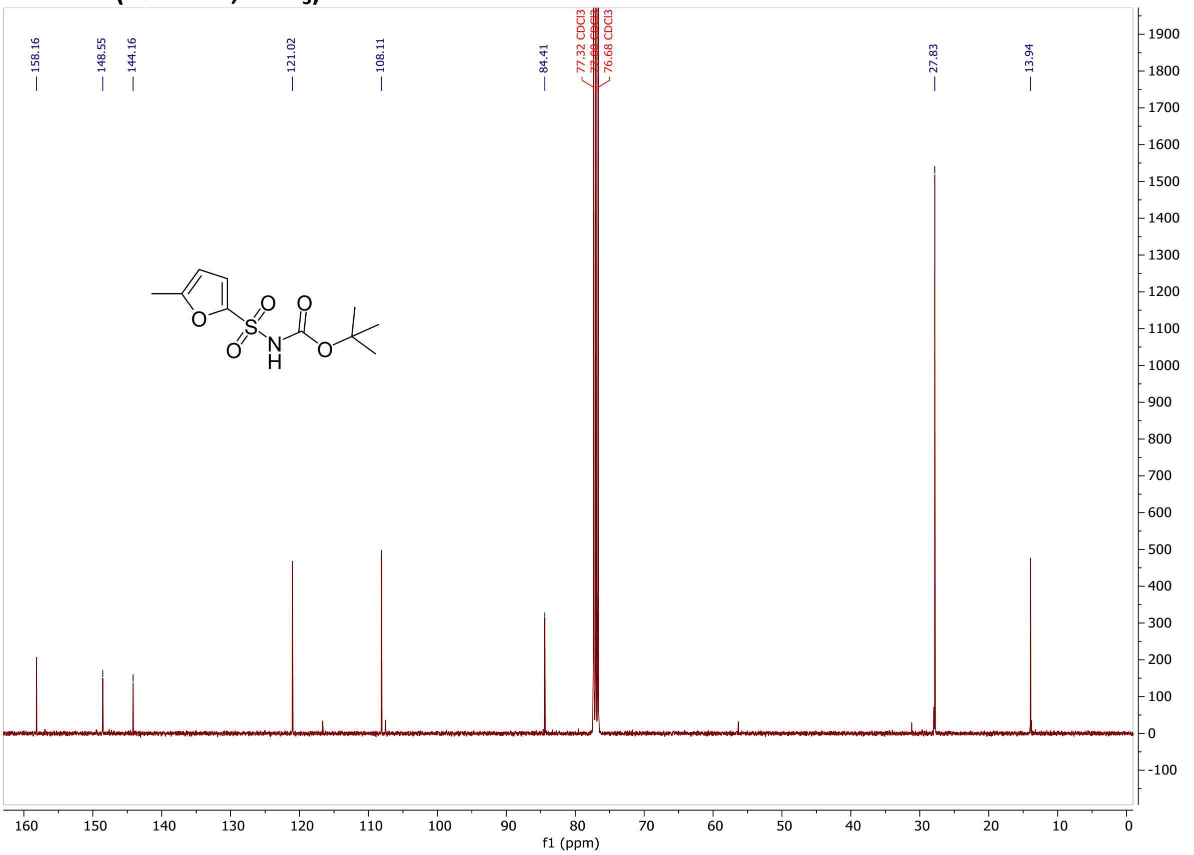

***tert*-Butyl (*E*)-((2-(dimethylamino)styryl)sulfonyl)carbamate (5f)**

^{1}H-NMR (400 MHz, CDCl$_3$)

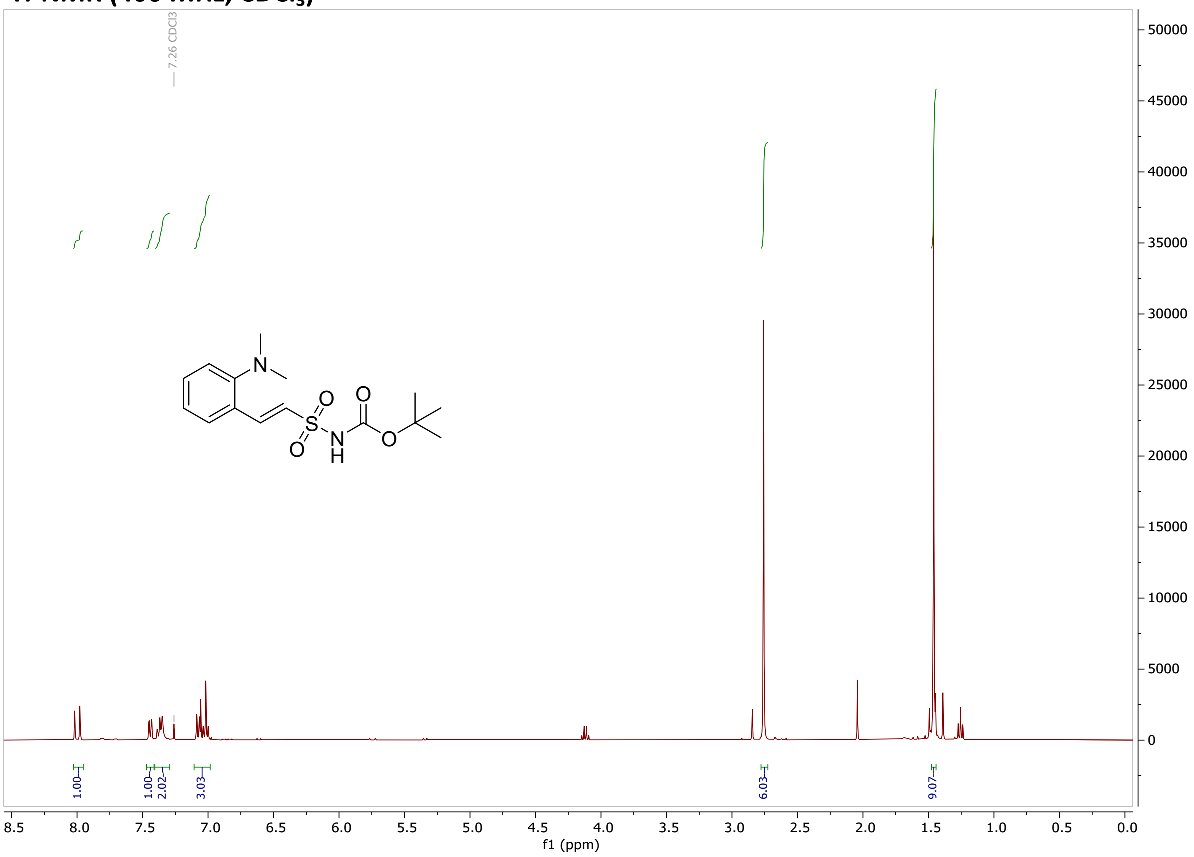

^{13}C-NMR (101 MHz, CDCl$_3$)

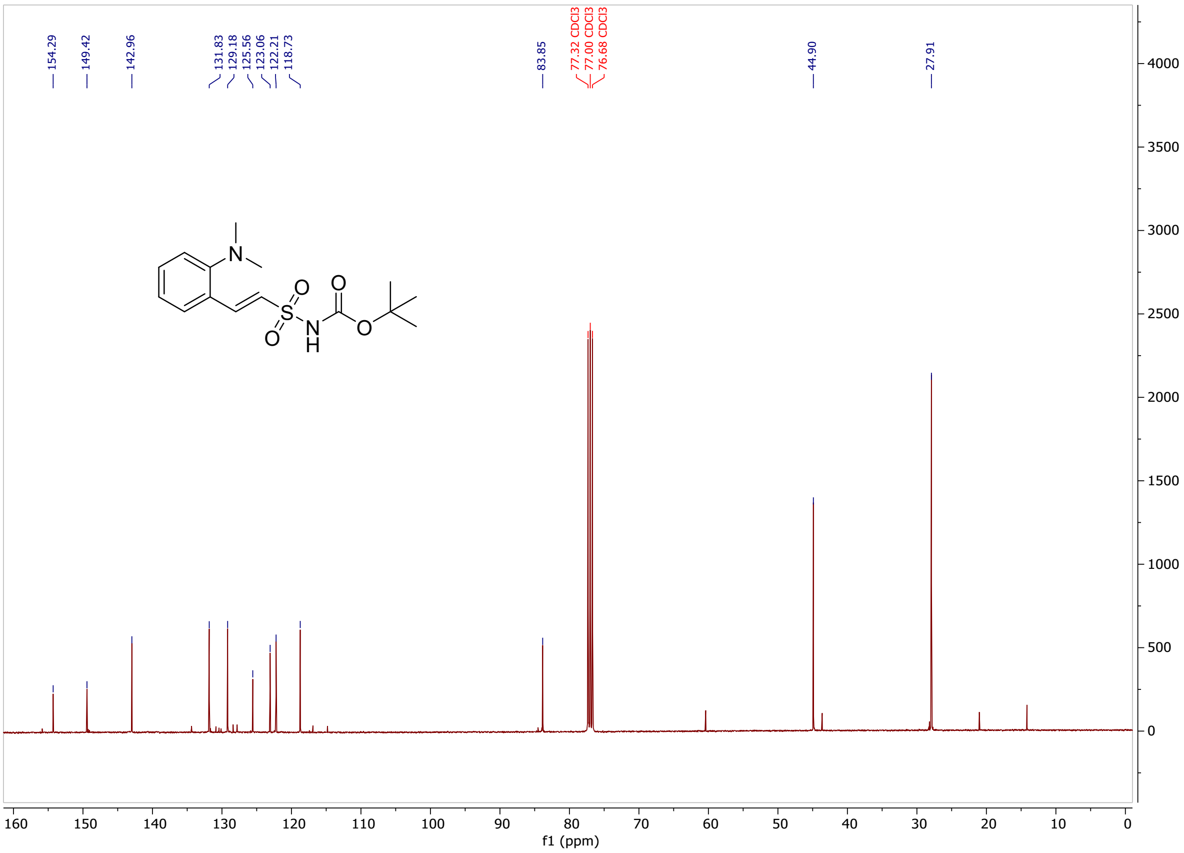

Bis(acetoxymethyl) 2,2'-((4-(*N*-(*tert*-butoxycarbonyl)sulfamoyl)phenyl)azanediyl)diacetate (8)
^{1}H-NMR (400 MHz, $CDCl_3$)

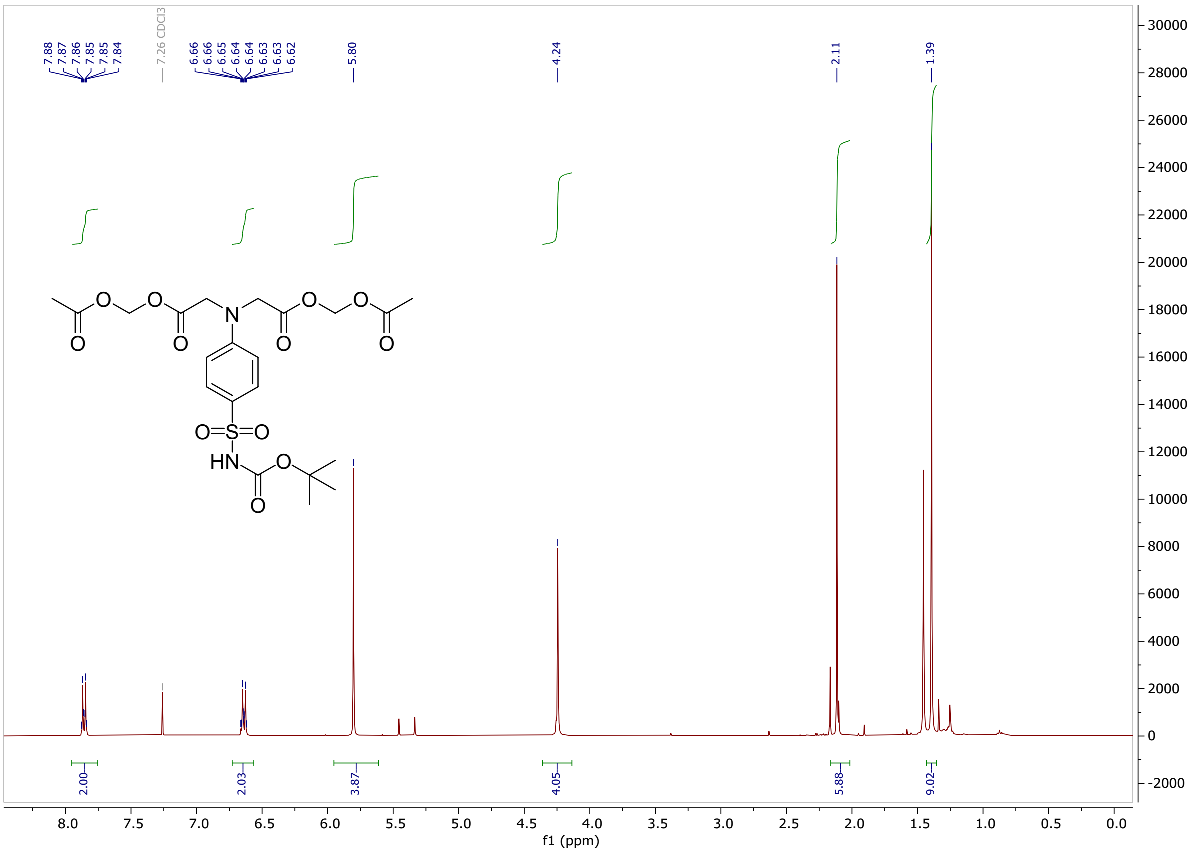

^{13}C-NMR (101 MHz, CDCl$_3$)

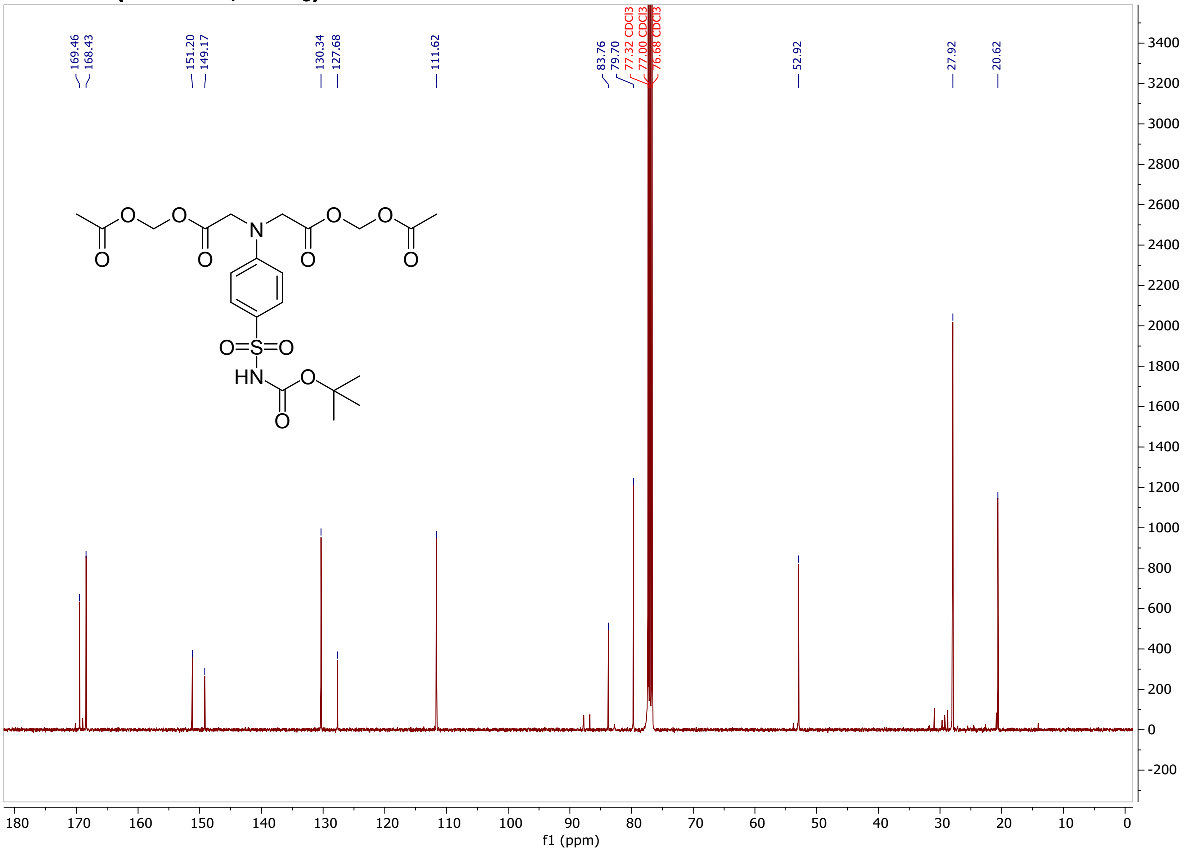

Bis(acetoxymethyl) 2,2'-((4-sulfamoylphenyl)azanediyl)diacetate (9)

^{1}H-NMR (400 MHz, $CDCl_3$)

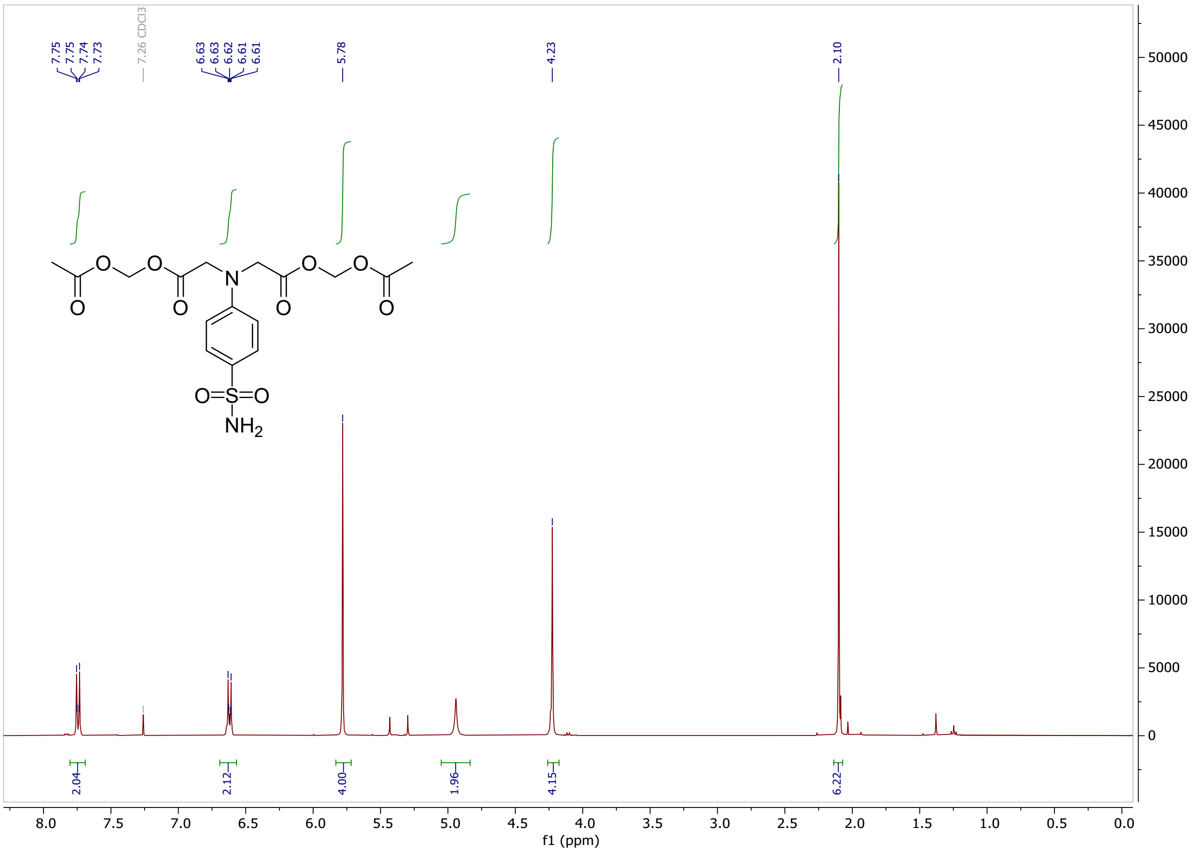

^{13}C-NMR (101 MHz, $CDCl_3$)

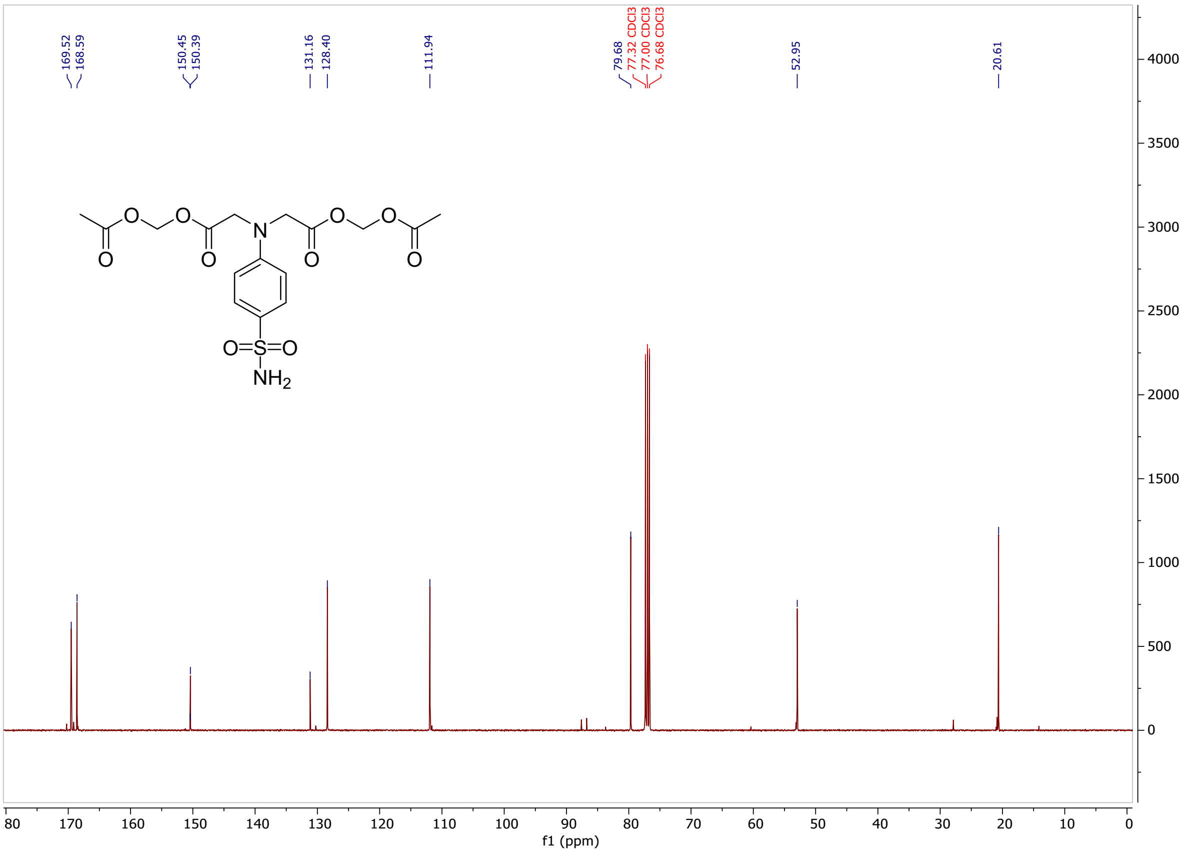

Bis(acetoxymethyl) 2,2'-((2-(2-(2-(bis(2-(acetoxymethoxy)-2-oxoethyl)amino)-5-sulfamoylphenoxy)ethoxy)phenyl)azanediyl)diacetate (11)
^{1}H-NMR (400 MHz, $CDCl_3$)

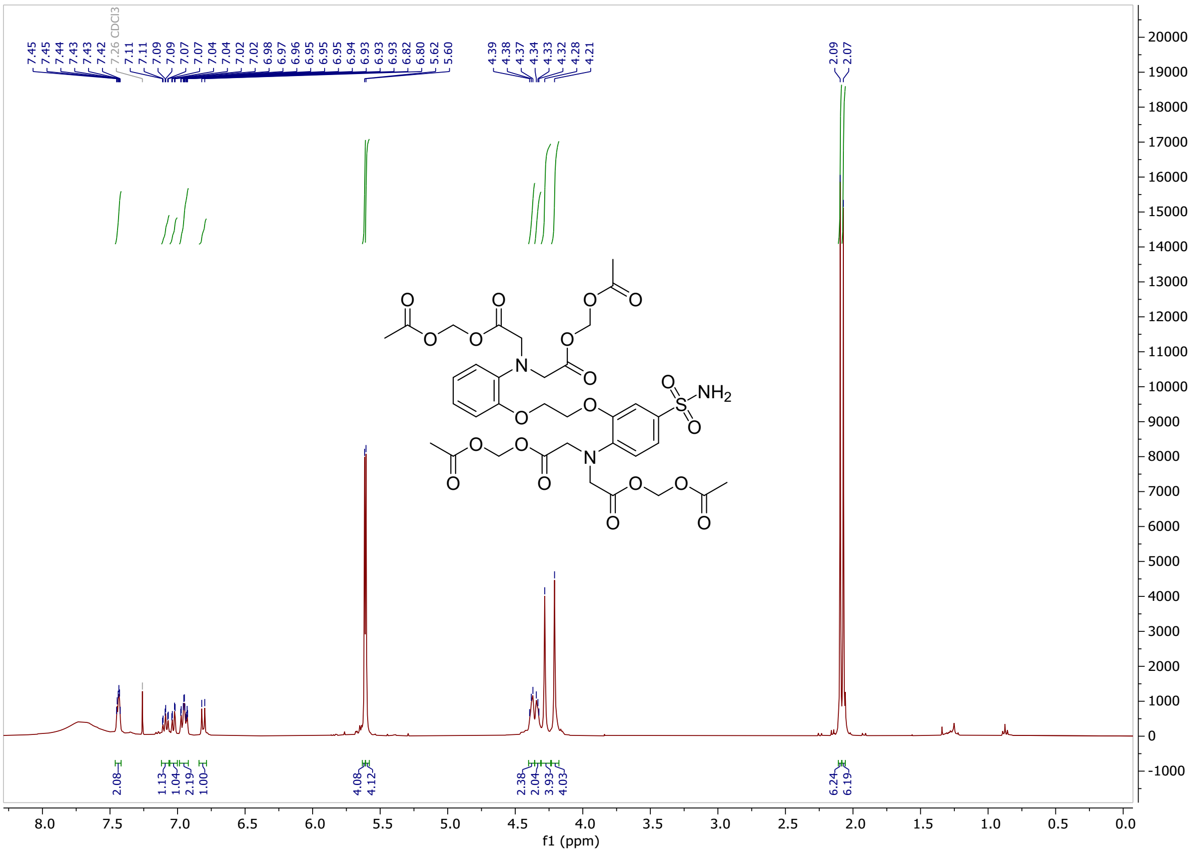

^{13}C-NMR (101 MHz, $CDCl_3$)

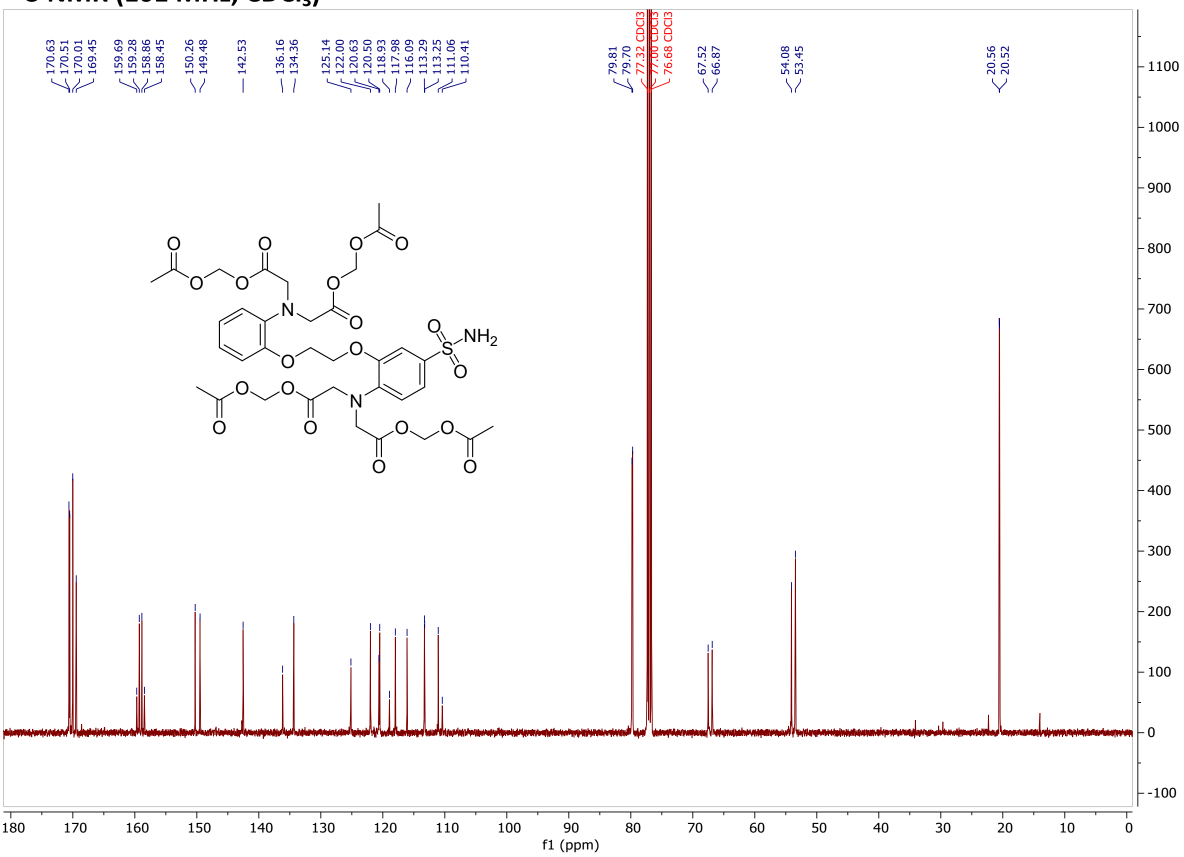

Bis(acetoxymethyl) 2,2'-((2-methoxyphenyl)azanediyl)diacetate (12)

^{1}H-NMR (400 MHz, $CDCl_3$)

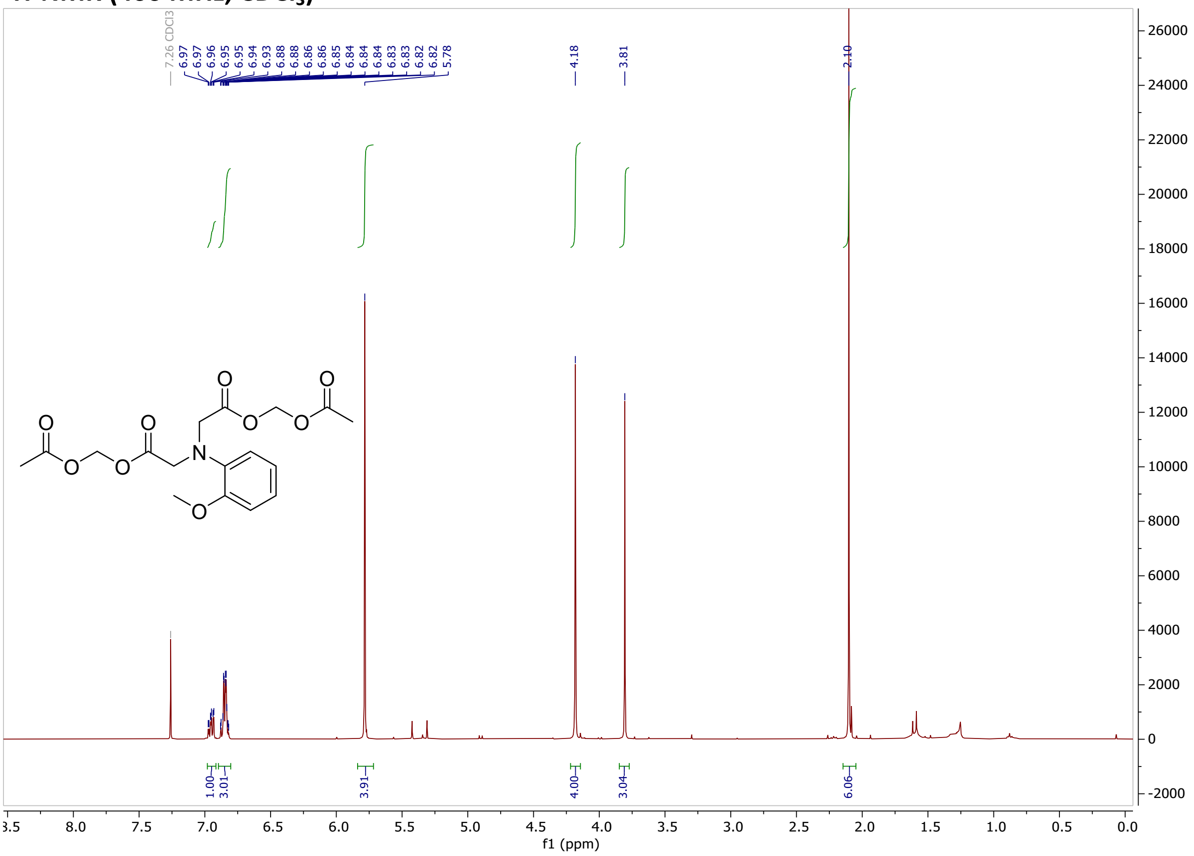

^{13}C-NMR (101 MHz, $CDCl_3$)

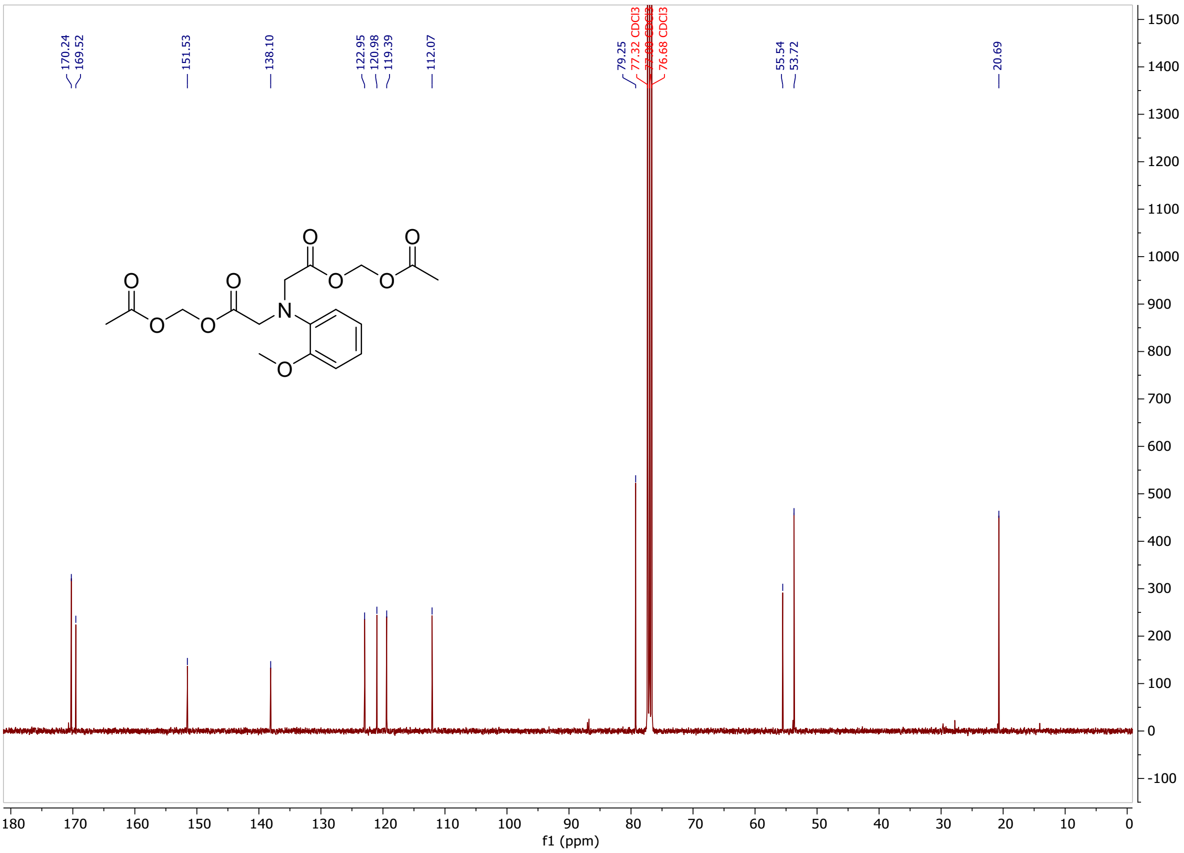

Bis(acetoxymethyl) 2,2'-((2-methoxy-4-sulfamoylphenyl)azanediyl)diacetate (13)

^{1}H-NMR (400 MHz, $CDCl_3$)

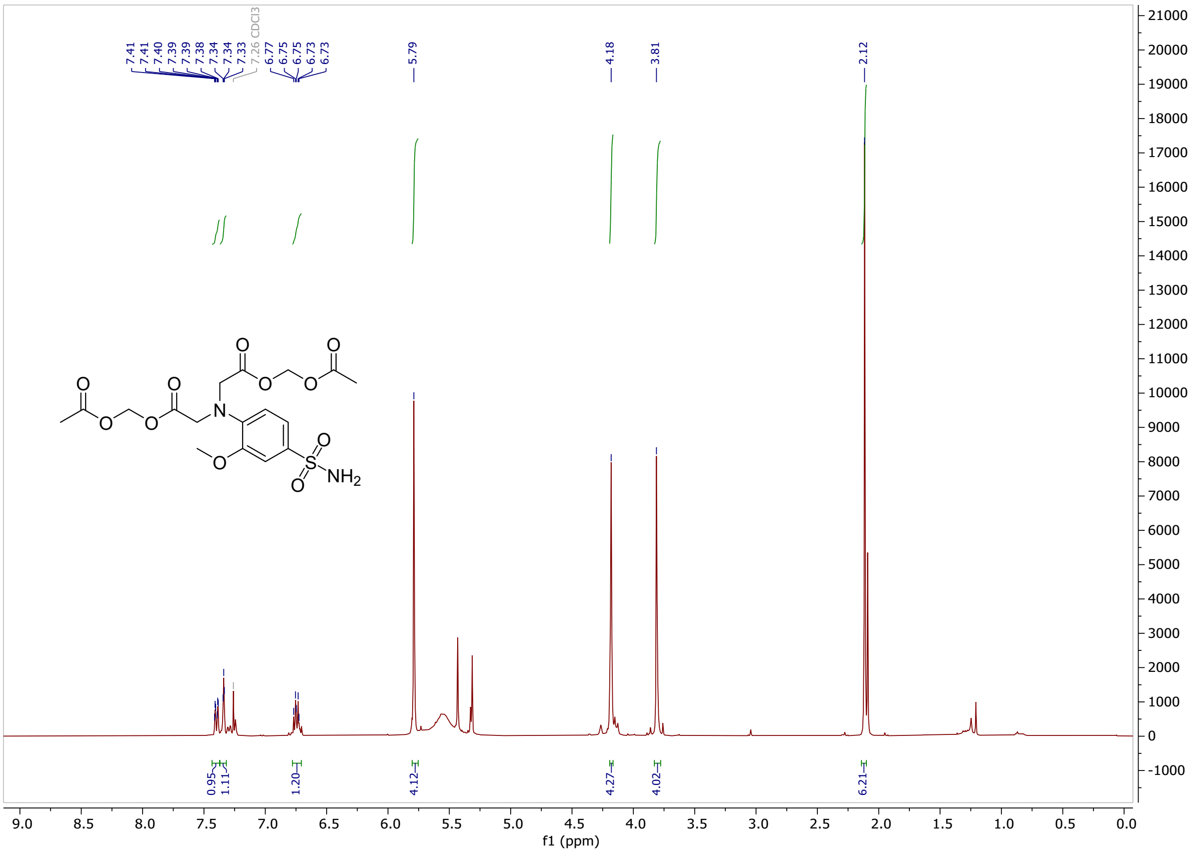

^{13}C-NMR (101 MHz, $CDCl_3$)

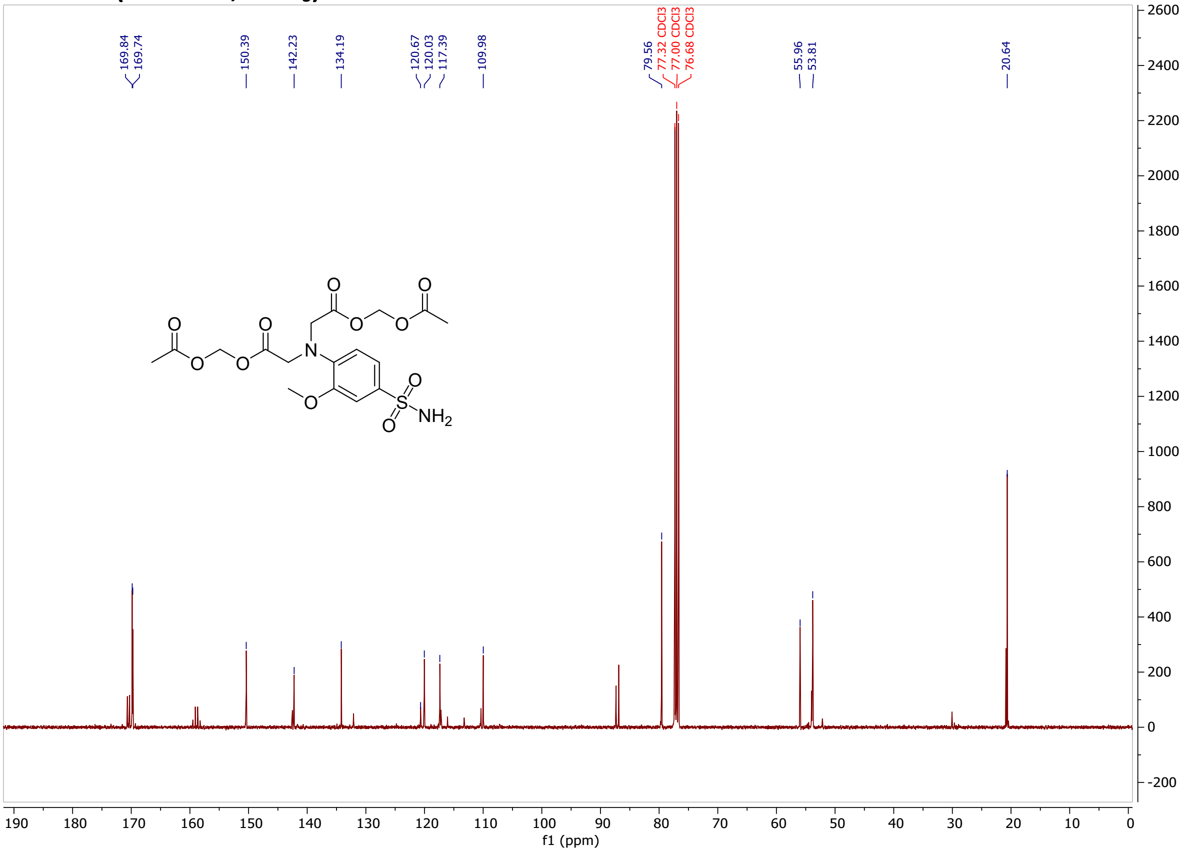

Bis(acetoxymethyl) 2,2'-((2-(2-(acetoxymethoxy)-2-oxoethoxy)phenyl)azanediyl)diacetate (14)

^{1}H-NMR (400 MHz, CDCl$_3$)

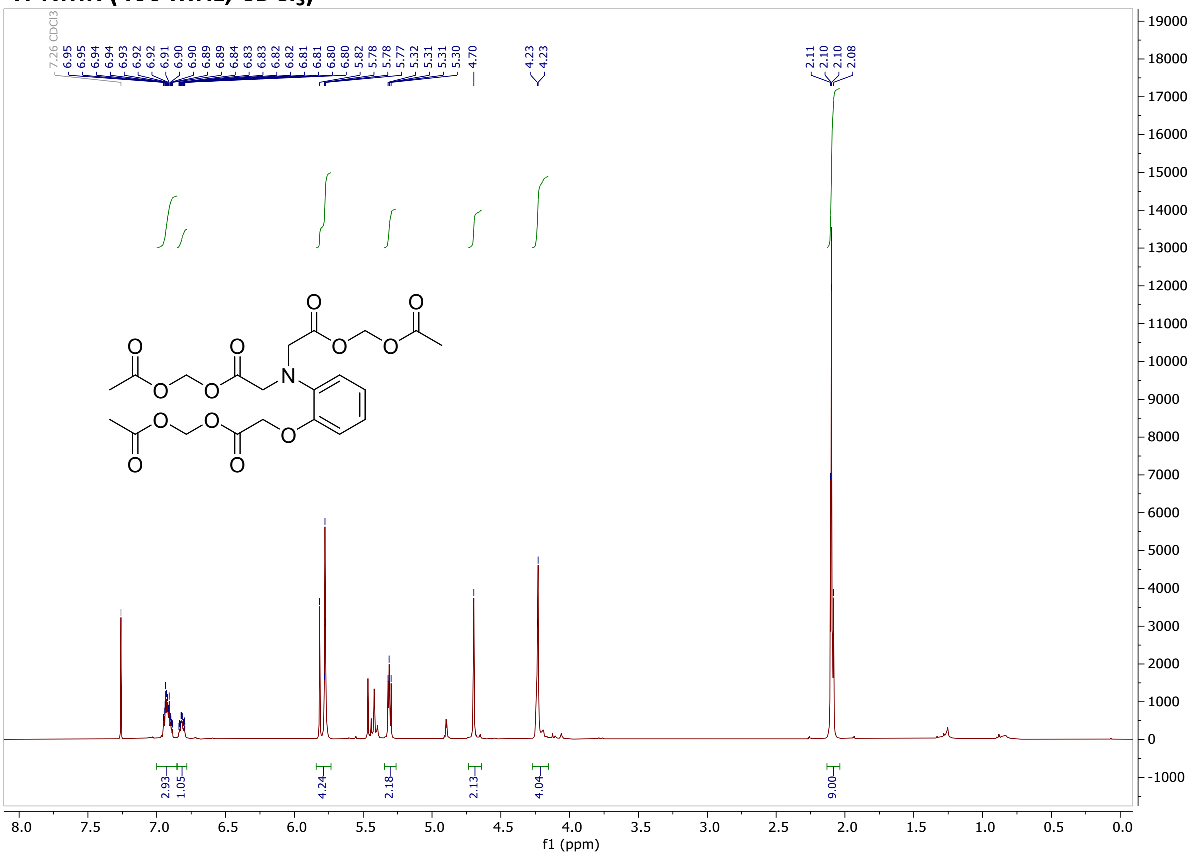

^{13}C-NMR (101 MHz, CDCl$_3$)

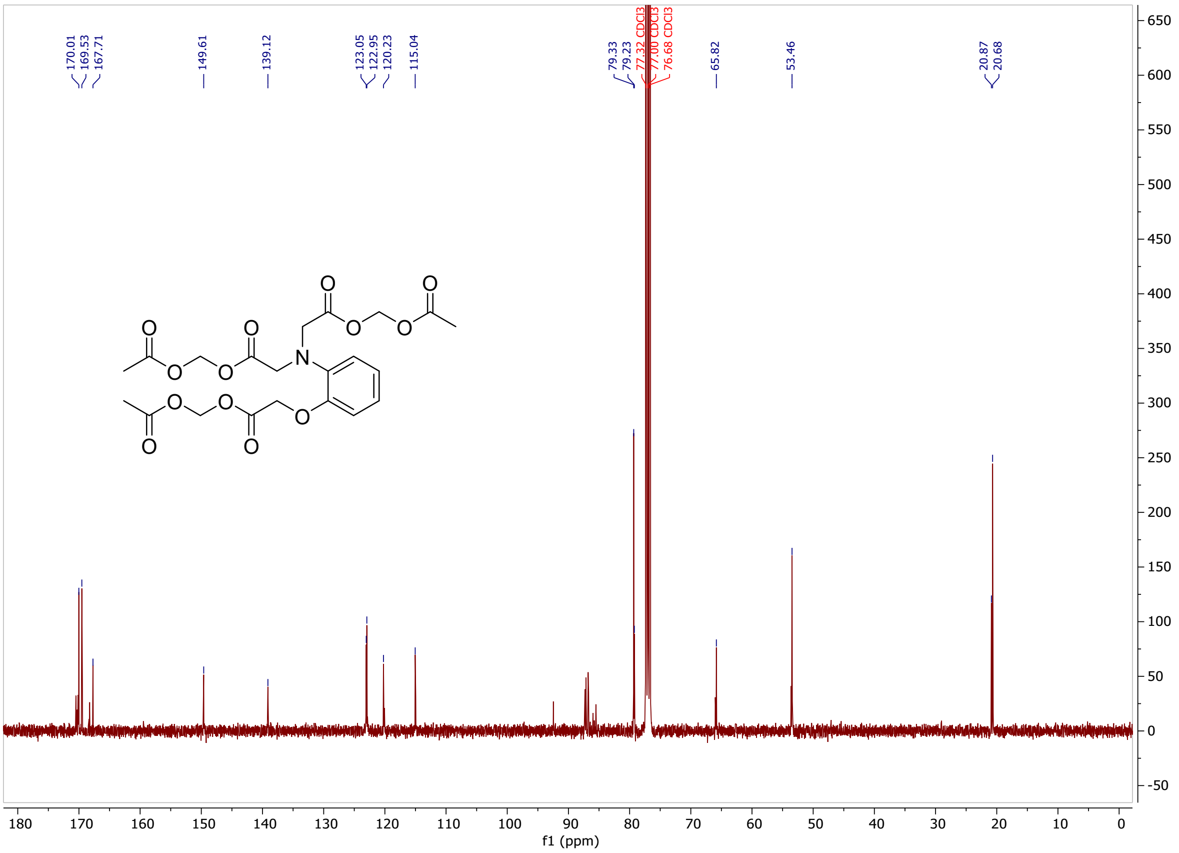

Bis(acetoxymethyl) 2,2'-((2-(2-(acetoxymethoxy)-2-oxoethoxy)-4-sulfamoylphenyl)azanediyl)diacetate (15)

^{1}H-NMR (400 MHz, $CDCl_3$)

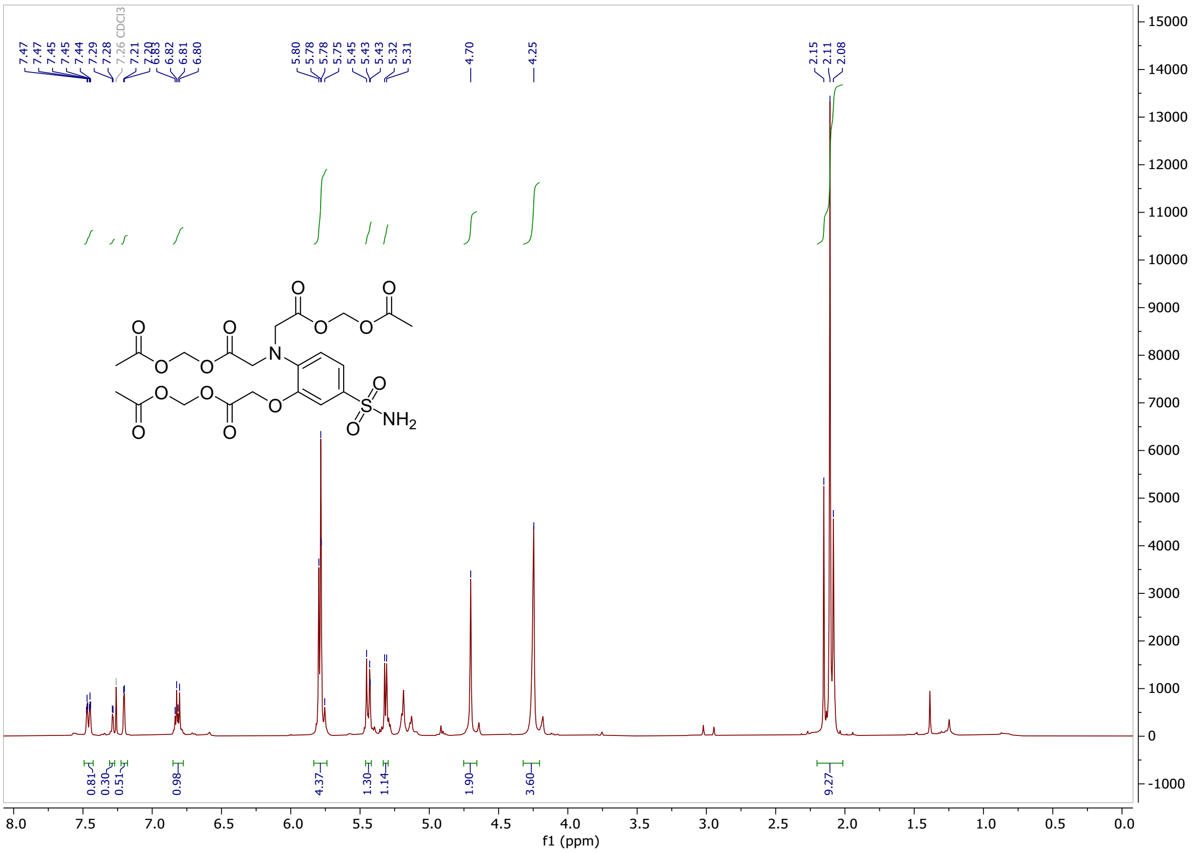

^{13}C-NMR (101 MHz, CDCl$_3$)

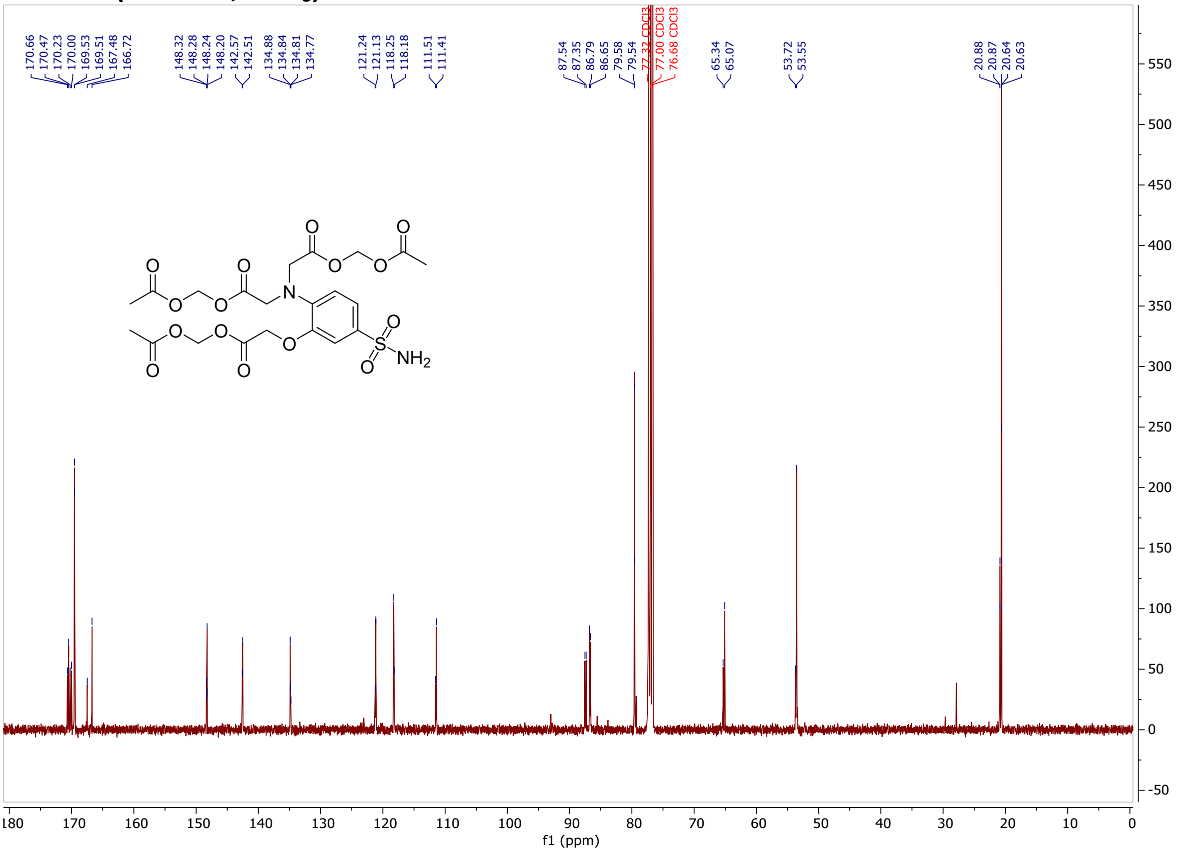

4-(1,4,7,10,13-Pentaoxa-16-azacyclooctadecan-16-yl)-3-(2-methoxyethoxy)benzenesulfonamide (2)

^{1}H-NMR (400 MHz, $CDCl_3$)

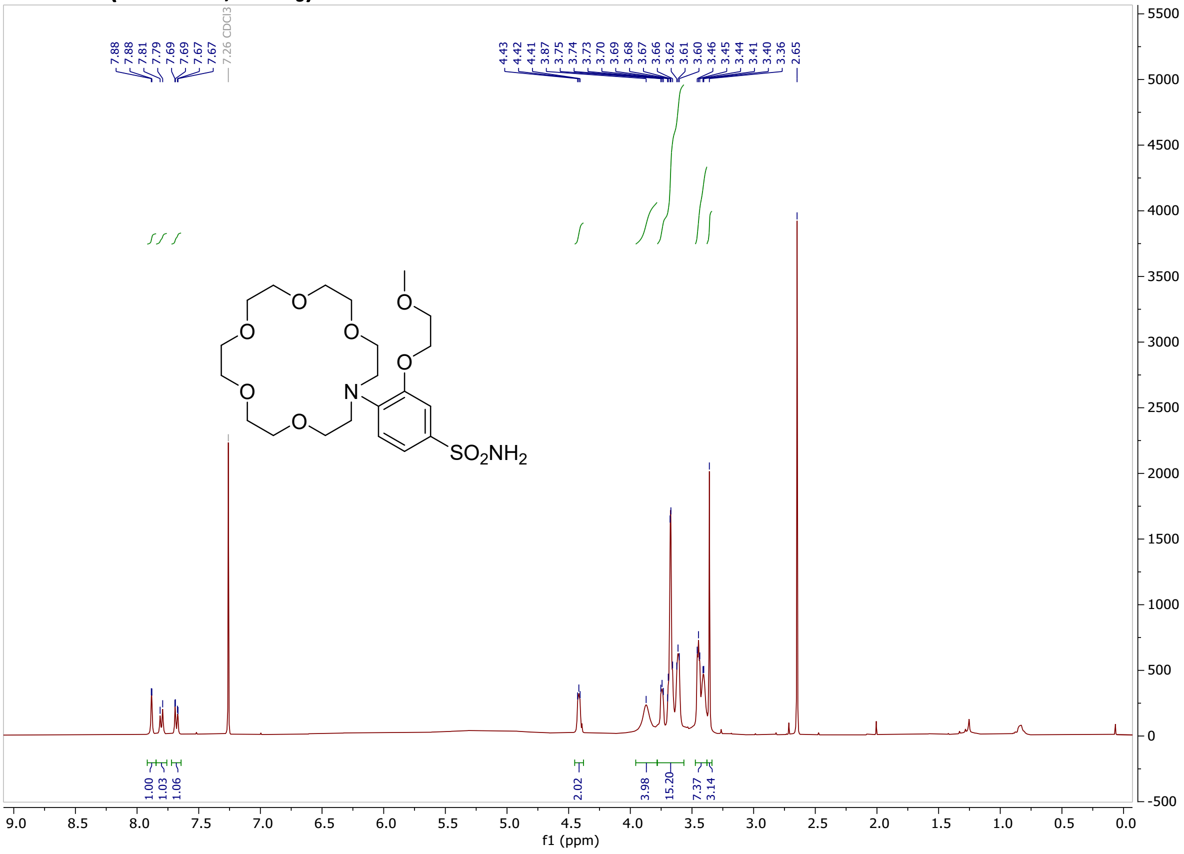

^{13}C-NMR (101 MHz, $CDCl_3$)

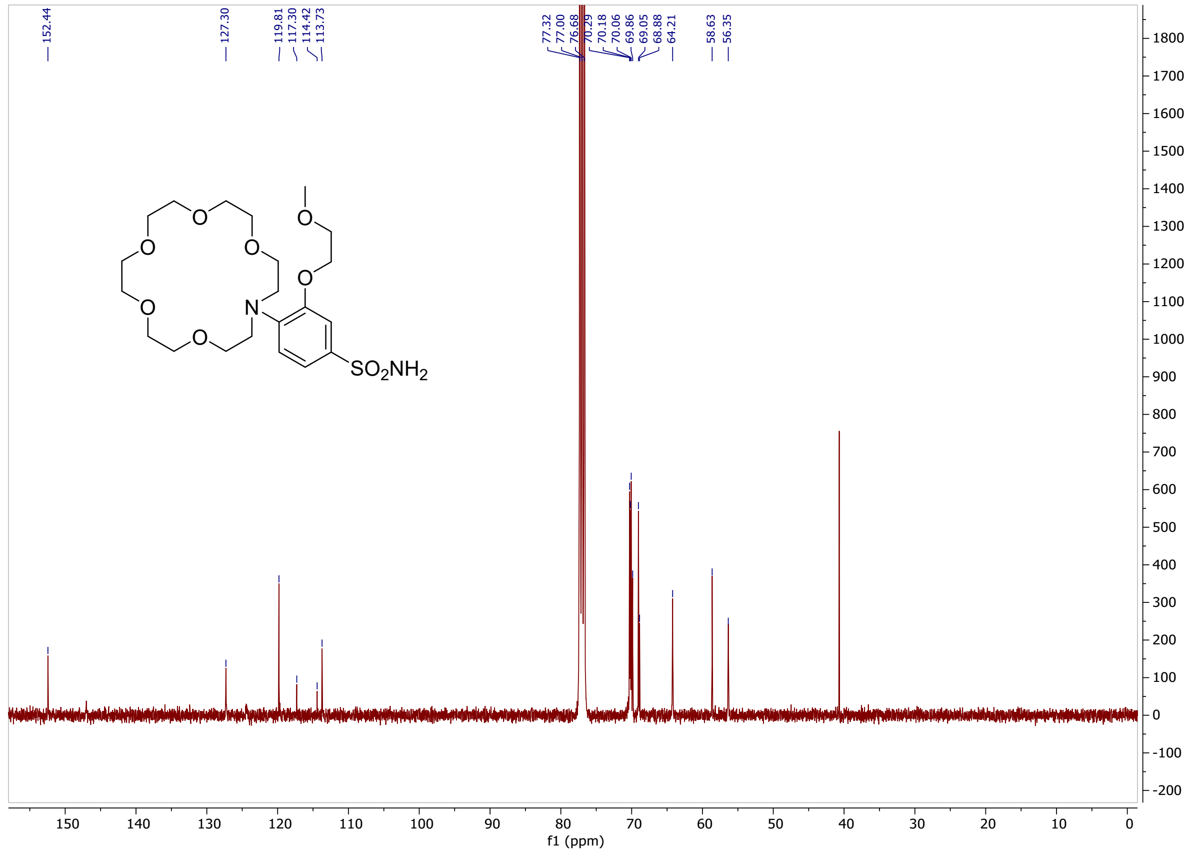

2-((4-(dimethylamino)phenyl)sulfonyl)-3-oxo-3',6'-bis((2,2,2-trifluoroethyl)amino)spiro[isoindoline-1,9'-xanthene]-6-carboxylic acid (18)

^{1}H-NMR (400 MHz, DMSO-d$_6$)

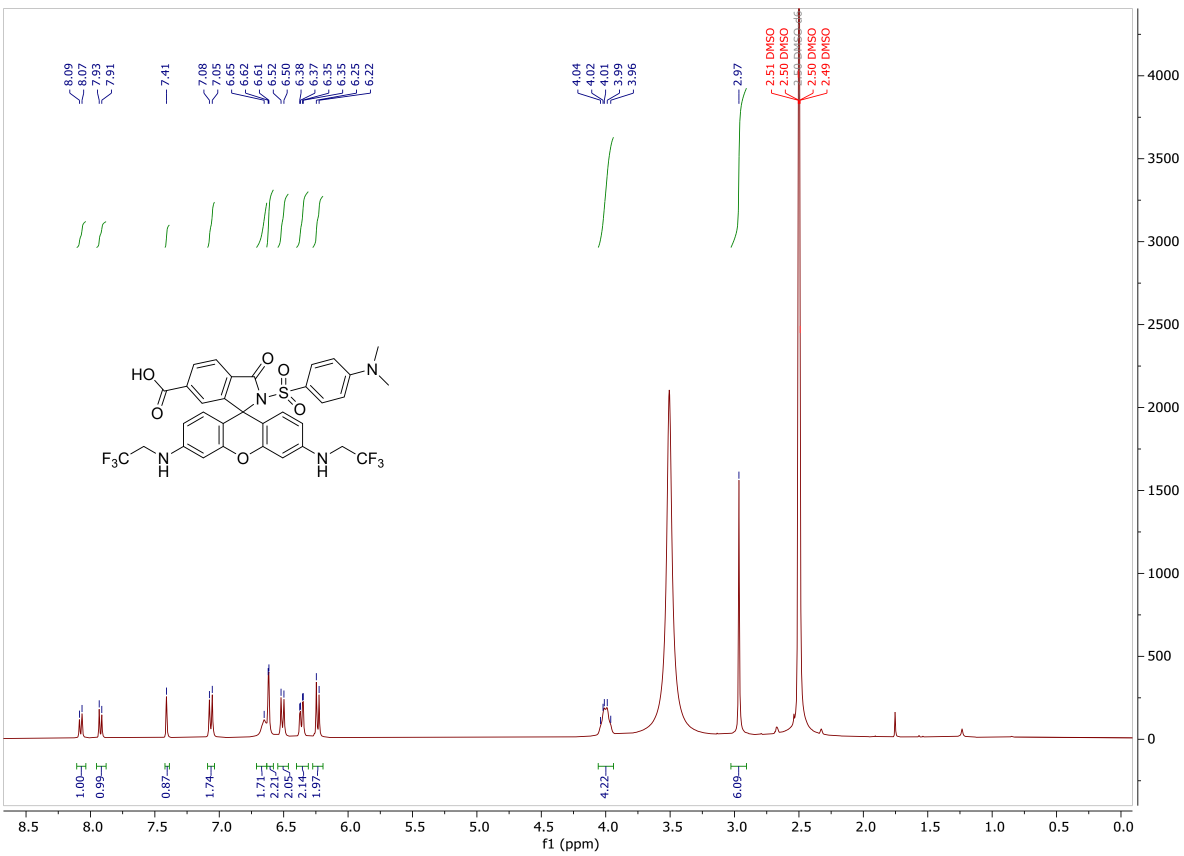

^{13}C-NMR (101 MHz, DMSO-d$_6$)

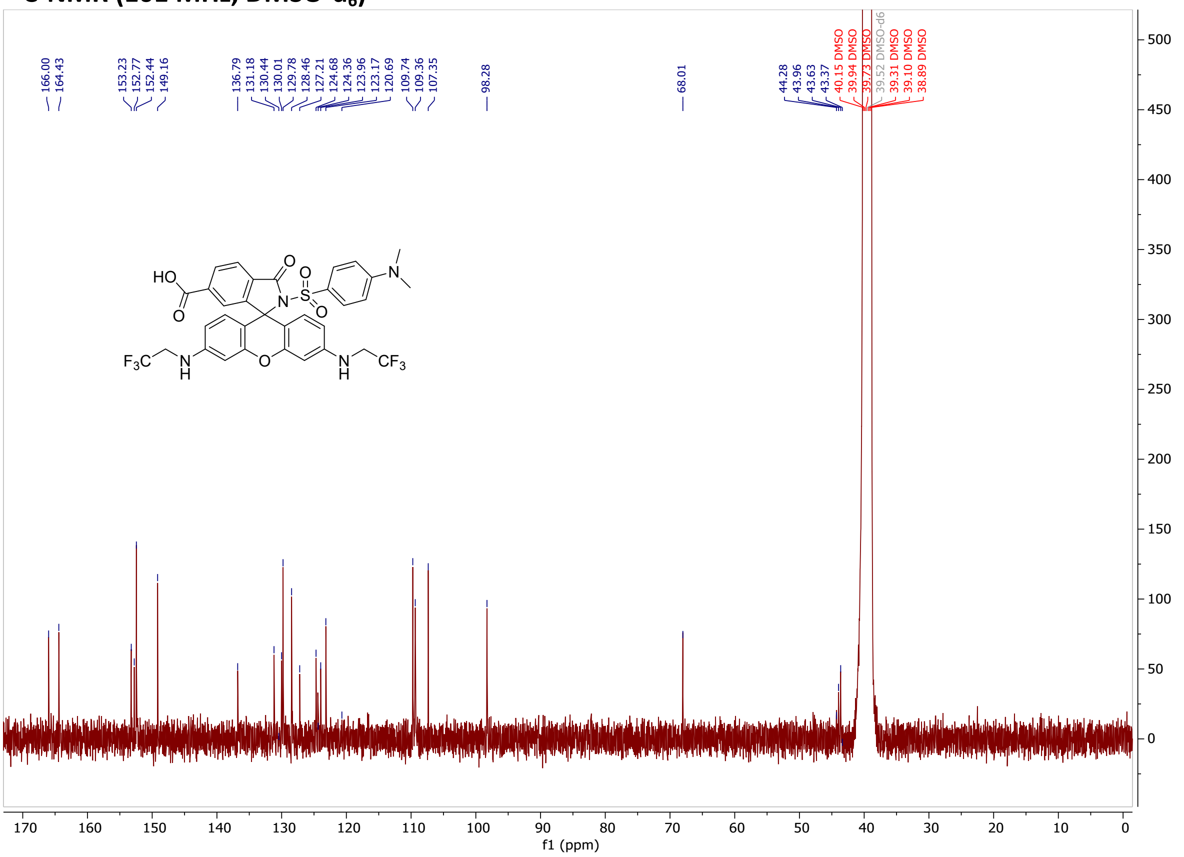

N-(2-(2-((6-Chlorohexyl)oxy)ethoxy)ethyl)-2-((4-(dimethylamino)phenyl)sulfonyl)-3-oxo-3',6'-bis((2,2,2-trifluoroethyl)amino)spiro[isoindoline-1,9'-xanthene]-6-carboxamide (20)

^{1}H-NMR (400 MHz, CD_3OD)

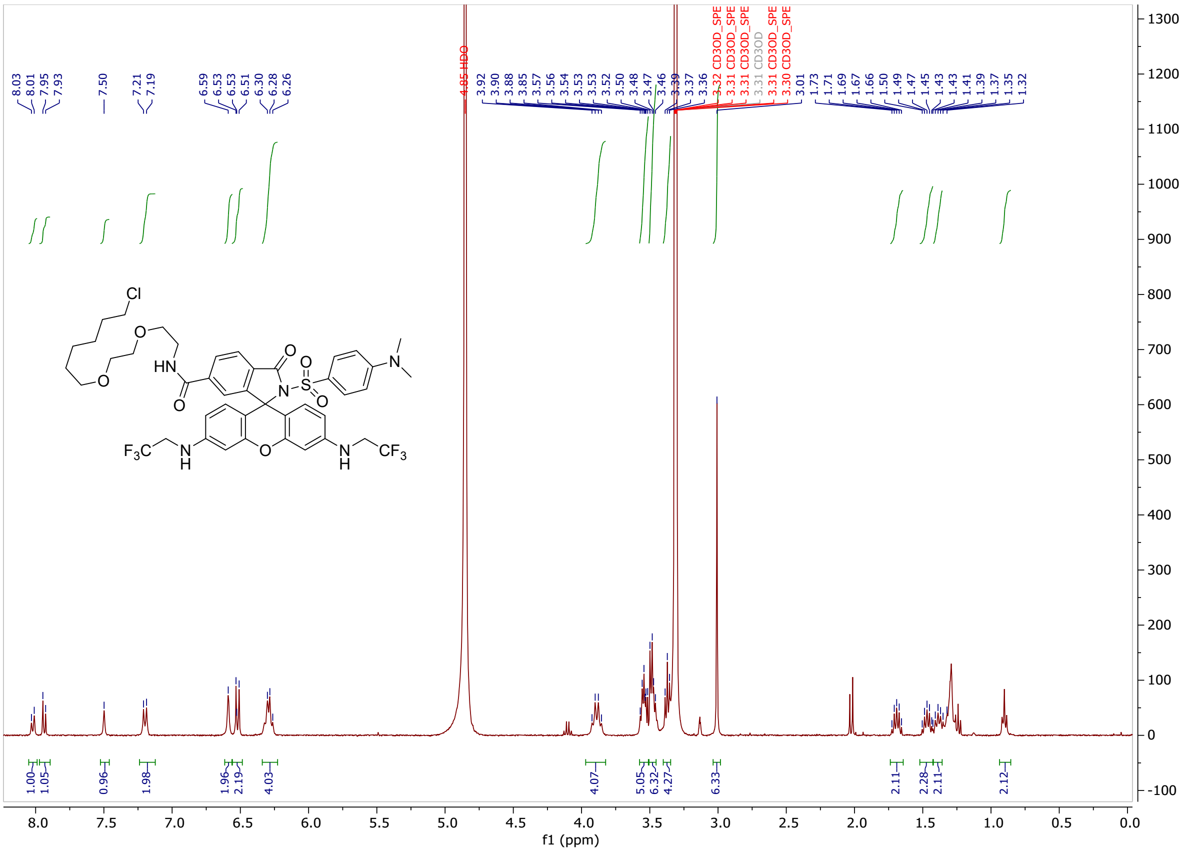

Benzyl ((4-(dimethylamino)phenyl)sulfonyl)carbamate (4a-Cbz)

^{1}H-NMR (400 MHz, $CDCl_3$)

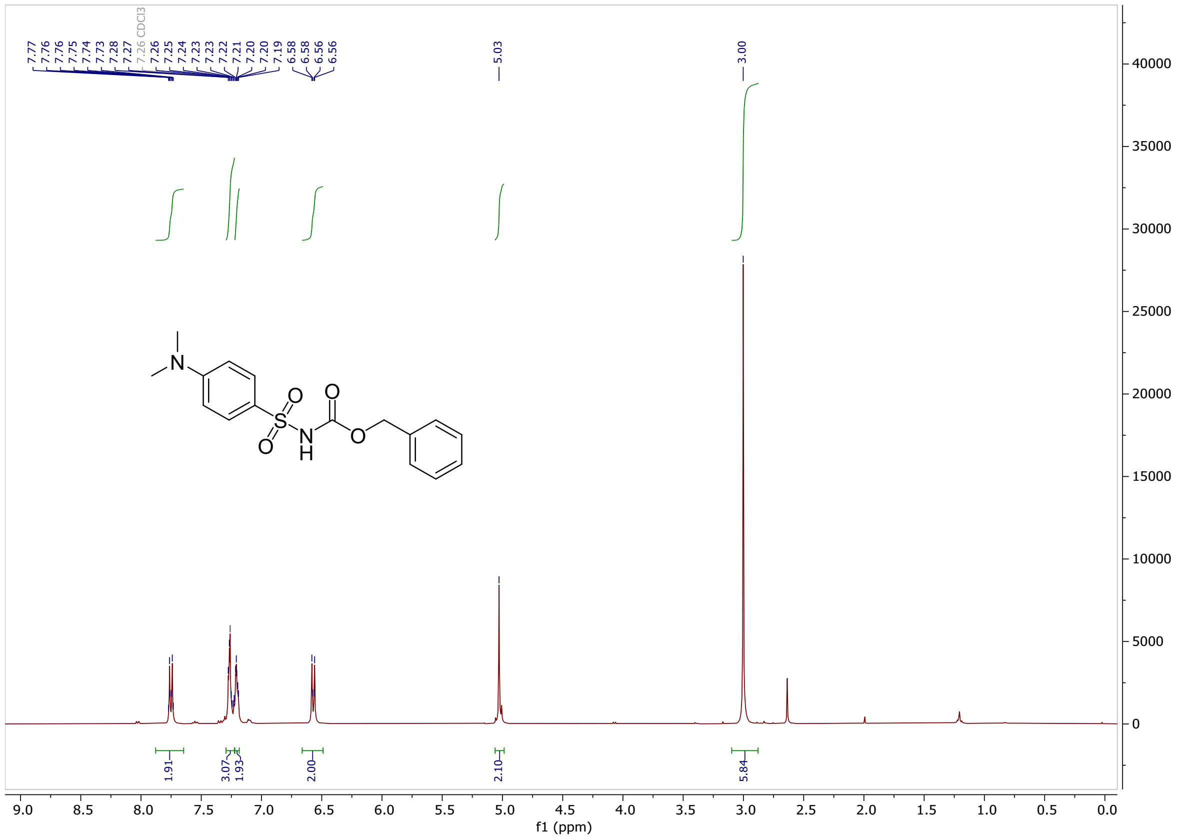

^{13}C-NMR (101 MHz, $CDCl_3$)

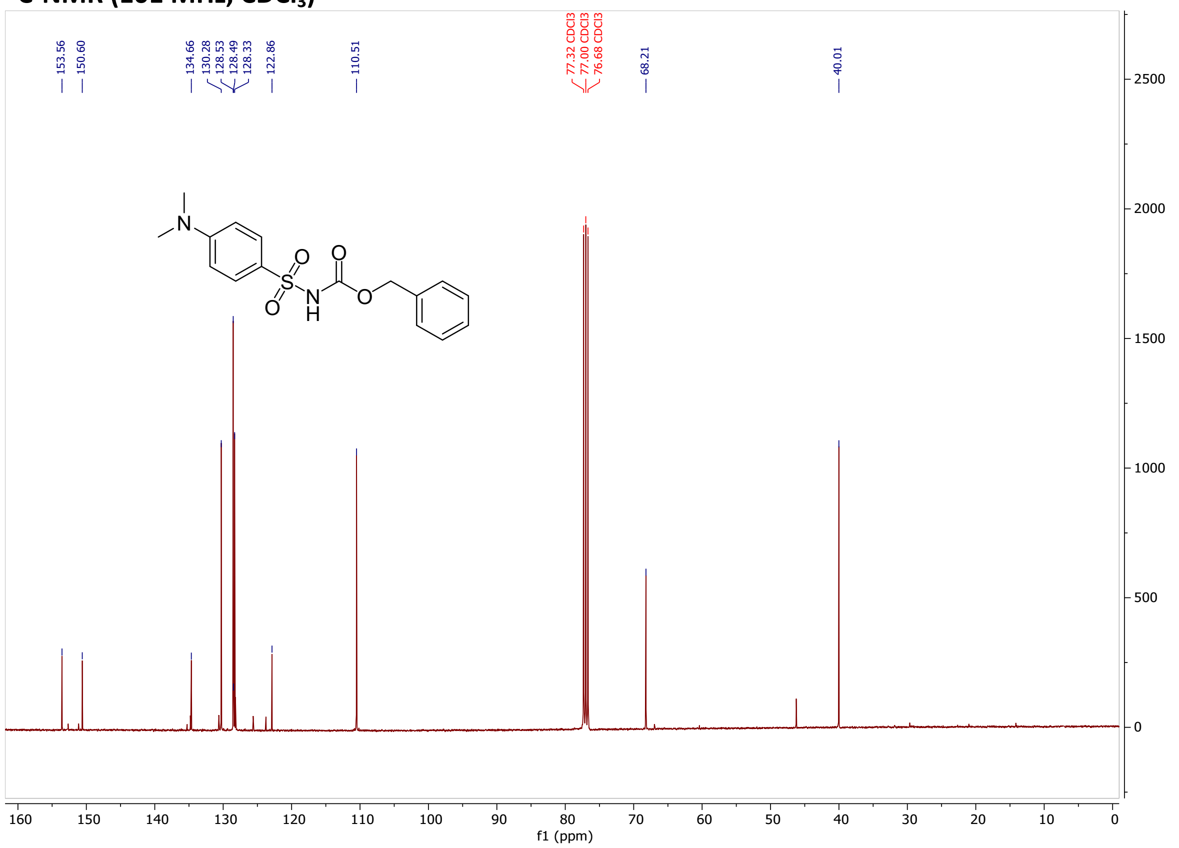

Allyl ((4-(dimethylamino)phenyl)sulfonyl)carbamate (4a-Alloc)

^{1}H-NMR (400 MHz, $CDCl_3$)

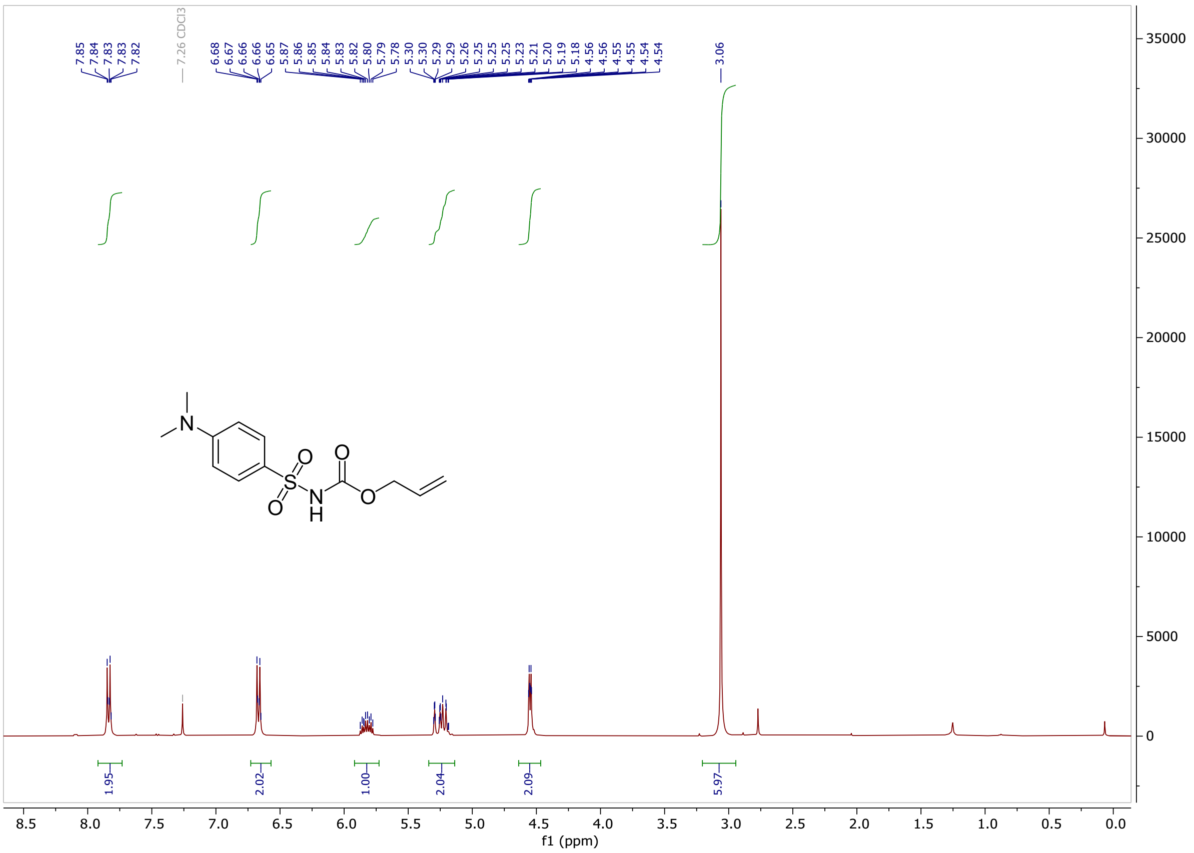

^{13}C-NMR (101 MHz, $CDCl_3$)

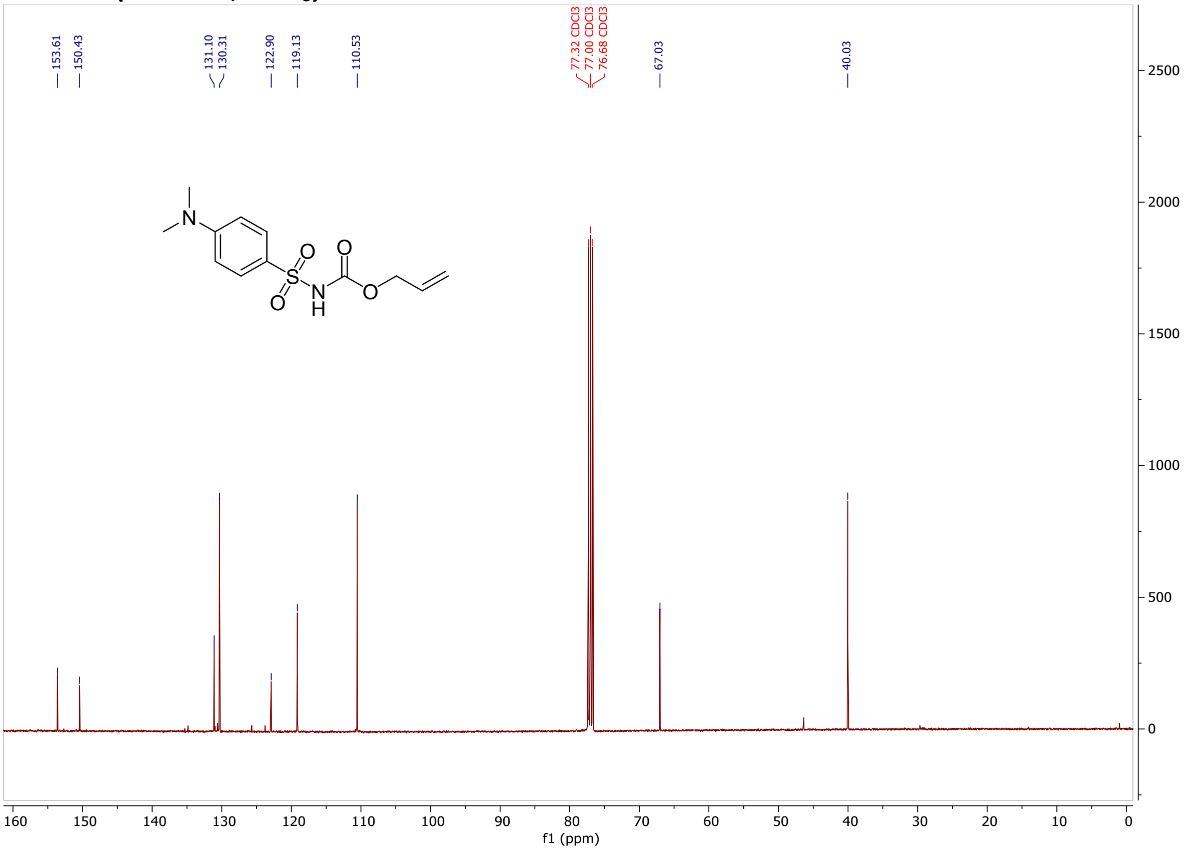

2,2,2-Trichloroethyl ((4-(dimethylamino)phenyl)sulfonyl)carbamate (4a-Troc)

^{1}H-NMR (400 MHz, $CDCl_3$)

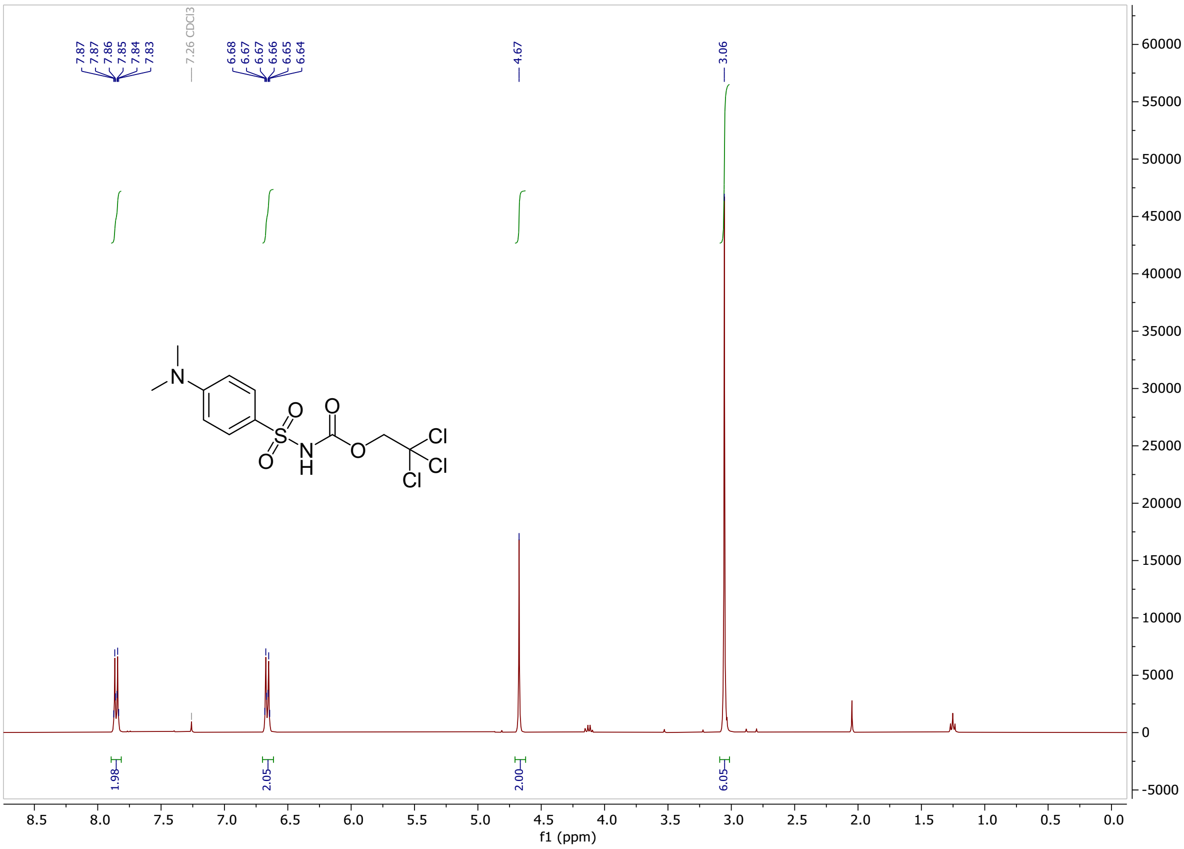

^{13}C-NMR (101 MHz, $CDCl_3$)

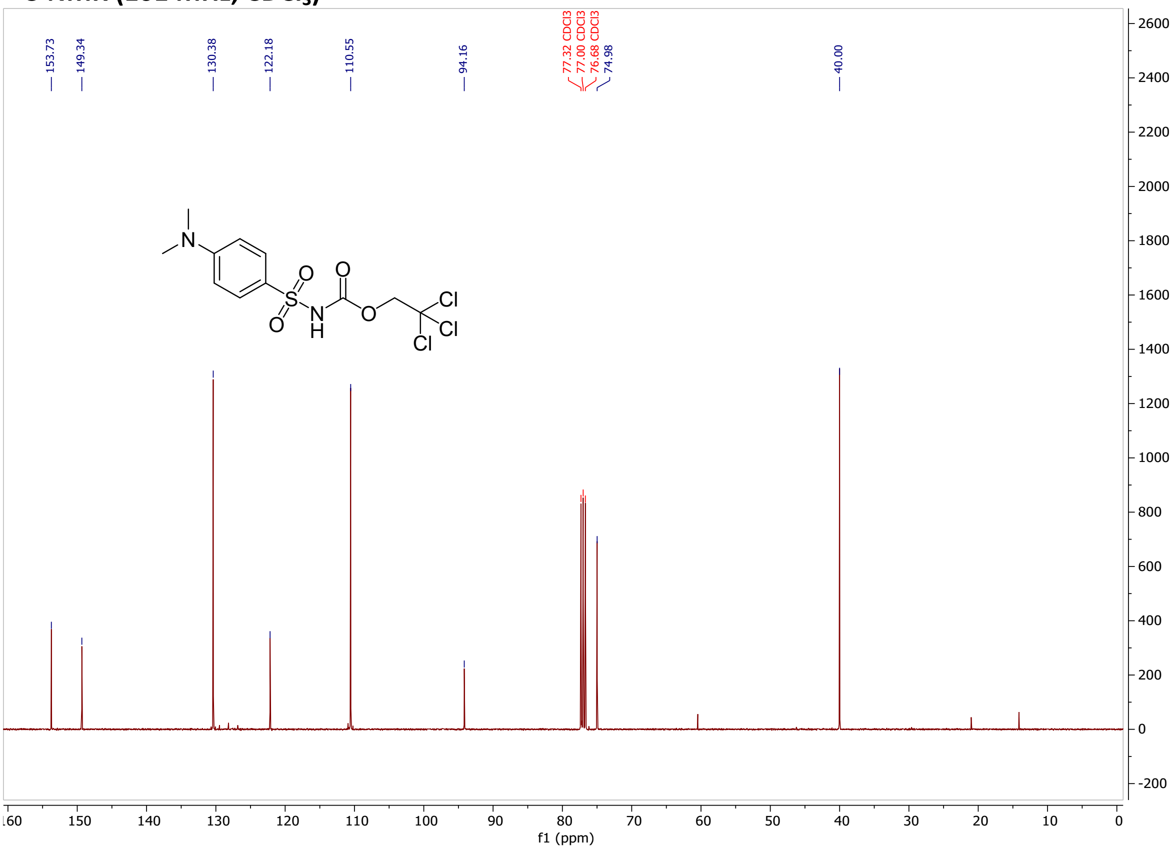

***N*-((4-(Dimethylamino)phenyl)sulfonyl)benzamide (4a-Bz)**
^{1}H-NMR (400 MHz, $CDCl_3$)

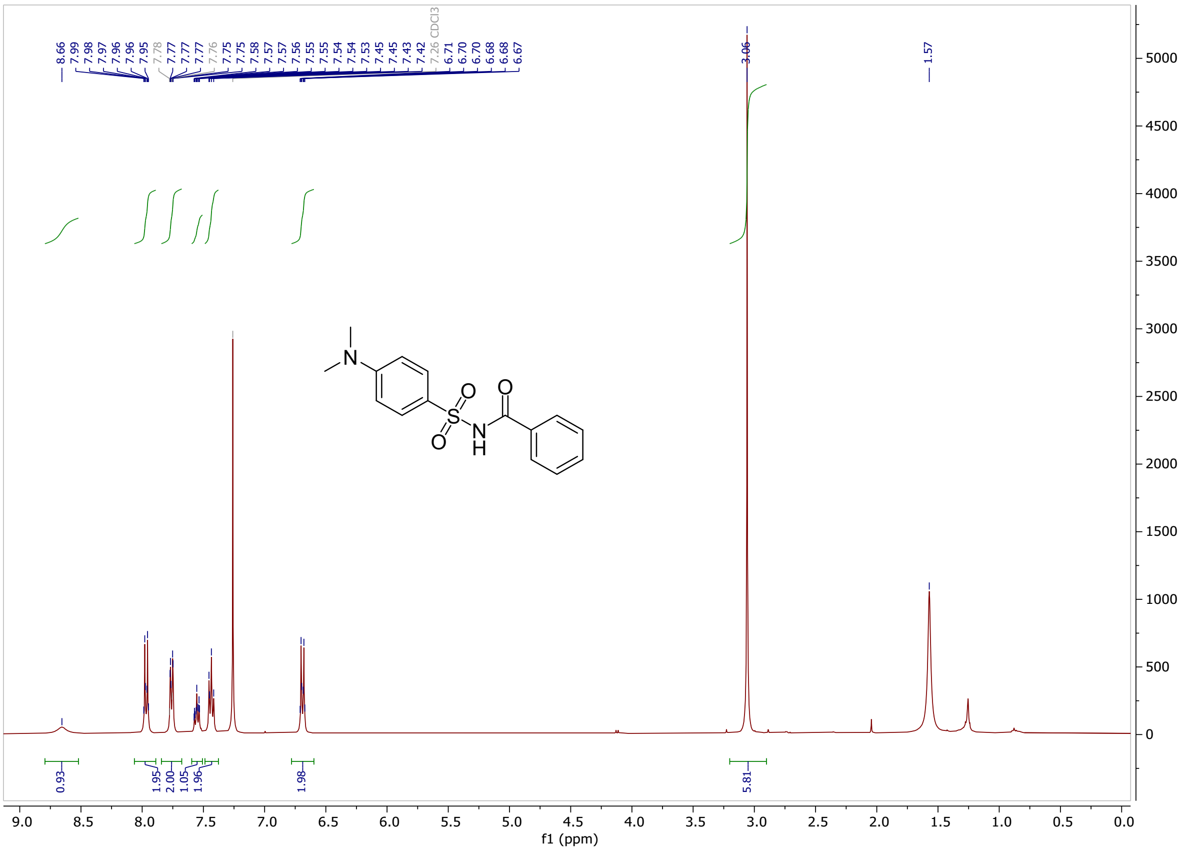

^{13}C-NMR (101 MHz, $CDCl_3$)

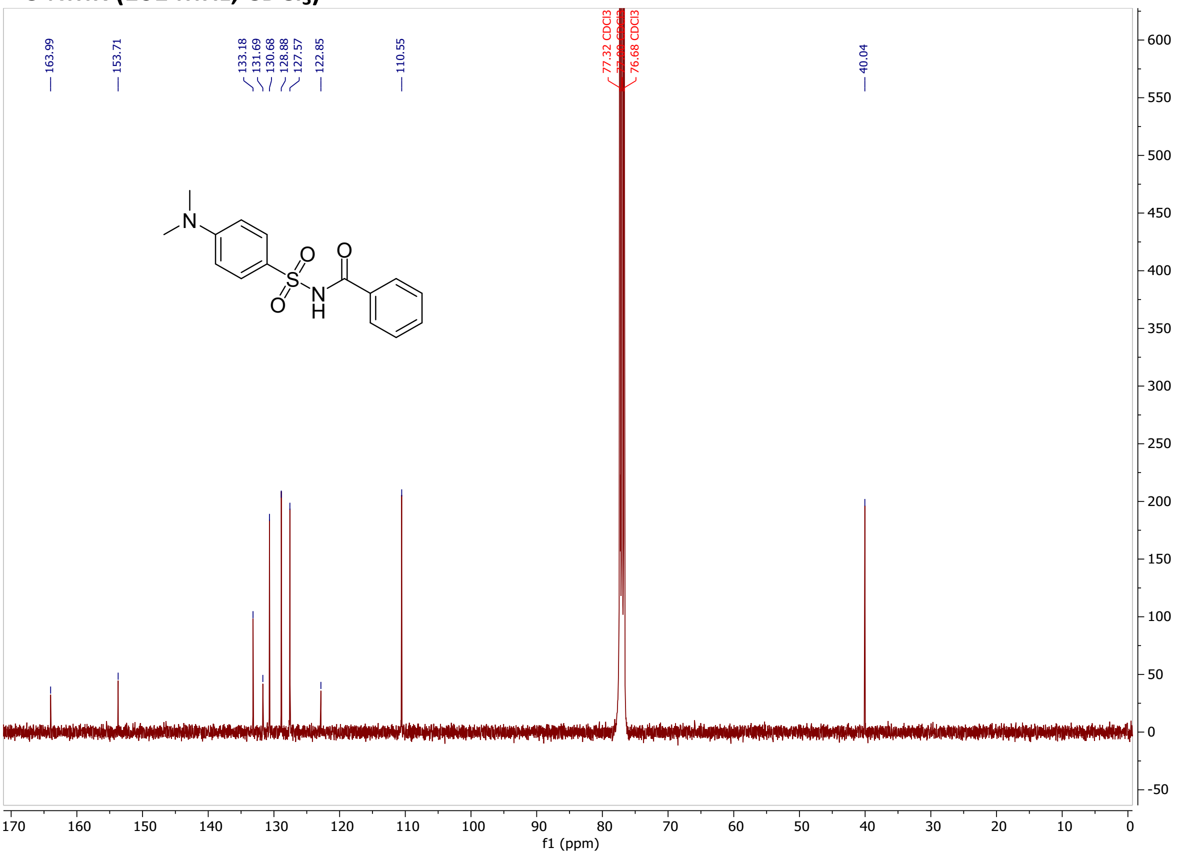

Supplement: SC-015-D4SC03075C-s001 [file SC-015-D4SC03075C-s001.pdf]
